# Association between anthropometric factors and meningioma risk: A systematic review and meta-analysis

**DOI:** 10.1371/journal.pone.0323461

**Published:** 2025-05-13

**Authors:** Chao Xu, Chuan Shao, Jing Wang, Xinmin Ding, Nan Wu

**Affiliations:** 1 Department of Neurosurgery, Chongqing General Hospital, Chongqing University, Chongqing, China; 2 Department of Plastic and Cosmetic Surgery, Xinqiao Hospital, Third Military Medical University, Chongqing, China; 3 Department of Neurosurgery, Shanxi Bethune Hospital, Shanxi Academy of Medical Sciences, Tongji Shanxi Hospital, Third Hospital of Shanxi Medical University, Taiyuan, China; Shaanxi Provincial People's Hospital, CHINA

## Abstract

**Background:**

Data regarding the association between anthropometric factors and meningioma risk are inconsistent. Our aim was to investigate the association of body mass index (BMI), height, waist to hip ratio (WHR), waist circumference, and meningioma risk through a comprehensive meta-analysis.

**Methods:**

An extensive review of literature was conducted in PubMed and Embase databases. Random-effects models were used to pool the study-specific relative risk estimates (RRs) and 95% confidence intervals (CIs). Moreover, we employed a dose-response meta-analysis with a one-stage robust error meta-regression (REMR) model.

**Results:**

We included nine prospective studies for four anthropometric factors listed above and meningioma risk. Compared with normal weight, both overweight (RR:1.11, 95% CI: 1.04, 1.19; *P* = 0.003, I^2^ = 0.0%) and obesity (RR: 1.38, 95% CI:1.16, 1.64; *P* < 0.001, I^2^ = 54.7%) were statistically significantly associated with meningioma risk. Dose-response analysis showed a nonlinear relationship between BMI and meningioma risk (*P* = 0.038). For height, a positive association was identified for men (RR:1.30, 95% CI:1.08, 1.56; *P* = 0.005, I^2^ = 0.0%) but not women (RR:1.13, 95% CI: 0.94,1.36; *P* = 0.186, I^2^ = 49.8%). Highest vs. lowest levels analyses also showed a positive association between meningioma risk and waist circumference (RR:1.89, 95% CI:1.34, 2.66; *P* < 0.001, I^2^ = 0.0%) and WHR (RR:1.40, 95% CI:1.00, 1.94; *P* = 0.048, I^2^ = 0.0%).

**Conclusion:**

Our meta-analysis indicates greater height (in men) and excess weight and body fat mass were associated with an increased risk of meningioma. Further prospective studies with particular attention to sex disparity and dose-response analysis are warranted to confirm our observation.

## Introduction

Meningiomas, arising from the arachnoid cap cells embedded in the arachnoid villi, are one of the most frequently diagnosed primary central nervous system (CNS) tumors, representing 41.7% of cases with an annual incidence rate of 10.15 per 100,000 population as documented in the 2024 CBTRUS Statistical Report (Central Brain Tumor Registry of the United States) [1]. The incidence of meningioma increases with age and demonstrates a strong female predominance, showing a female-to-male incidence ratio of 2.23 [1,2]. Understanding of the etiology of meningioma remains poor. To date, ionizing radiation exposure is the only environmental risk factor linked to the onset of meningioma [2].

Body size is associated with various hormonal, metabolic, and immunologic pathways that have been indicated or are suspected to play a role in cancer development [3]. Concerning meningioma, inconsistent results of anthropometric factors have been shown [4–28]. For example, higher height was significantly associated with an increased risk of meningioma in some [11] but not in other studies [4,8,10,12,13]. Similar findings were identified for body mass index (BMI) with meningioma risk. These disparities may be associated with the different sample sizes and other study designs. To provide solid evidence, several meta-analyses have been published [29–32]. Notably, retrospective case-control studies were included in previous meta-analyses; thus, their findings might have been biased. Moreover, several additional studies [11–13,15,23–28] on this issue have been conducted after the latest meta-analysis was published in 2016 [32]. Therefore, we performed an updated meta-analysis of prospective cohort studies to better understand the relationship between BMI and meningioma risk. To our knowledge, the evidence about other body measures and meningioma risk has not been comprehensively evaluated. In this meta-analysis, we also assessed the relationship between meningioma risk and height, waist circumference, and waist to hip ratio (WHR) for the first time.

## Materials and methods

### Reporting guideline

Our meta-analysis was reported in line with the guidelines outlined in the Preferred Reporting Items for Systematic Reviews and Meta-Analyses statement [33].

### Literature search

A comprehensive search of PubMed and Embase databases was conducted from inception through September 7, 2024. The search strategy included the following terms: body mass index, obesity, overweight, underweight, weight, adiposity, obese, anthropometry, height, BMI, waist circumference, hip circumference, waist to hip ratio, WHR, meningioma, central nervous system neoplasms, brain neoplasms, brain tumor, and brain cancer (detailed search strategy provided in S1 Table). No language restrictions were applied, but the search was limited to human studies. Additionally, reference lists of systematic reviews and meta-analyses were manually screened to identify supplementary publications. Study selection was performed by CX and independently verified by CS.

### Eligibility criteria

We included prospective cohort studies or case-control studies nested in prospective cohort studies that evaluated the relationship between meningioma risk and BMI, height, waist circumference, and WHR and provided adjusted relative risk (RR) estimates and 95% confidence intervals (CIs). In instances where multiple studies involved the same population, we prioritized the study with the longest follow-up period or largest sample size. Given the considerable bias, retrospective case-control, cross-sectional, and univariate analysis studies were excluded. Due to the substantial heterogeneity among brain tumors in terms of pathological characteristics and etiological origins, we excluded studies that analyzed brain tumors collectively or failed to specify histological subtypes (glioblastoma, meningioma, schwannoma, and other brain tumors). We also excluded those studies which reported all cases were spinal meningioma. Finally, we did not include those studies in which exposure was only assessed as a continuous variable (for example, per 10 cm).

### Data extraction and quality evaluation

Information regarding the first author, publication year, study location, sample size, follow-up years, exposure data, assessment method of exposure and outcome, the most adjusted RRs with 95% CIs, and confounders were extracted by XC and JW. Any discrepancies were settled by discussion with a third author (CS). Quality evaluation of the included studies was performed using the Newcastle-Ottawa Scale (https://www.ohri.ca/programs/clinical_epidemiology/oxford.asp, accessed on September 7, 2024). The scoring system comprises three components: selection (up to four stars), comparability (up to two stars), and outcome (up to three stars). In this study, several modifications were implemented to the guideline tool, including: (1) awarding a maximum of one star to studies reporting adjusted risk estimates, given that confounding factors represent a significant concern in observational research; and (2) granting one star to studies with a follow-up period exceeding the median or mean duration of 5 years or maximum duration of ten years. Moreover, a study awarded 7 stars or more was considered as a high-quality study.

### Statistical analysis

STATA software (version 15.0, STATA Corp., College Station, TX, USA) was used to perform all statistical analyses. Since the included studies had different study designs and settings, we used random-effects models by DerSimonian and Laird to pool the study-specific RRs with 95% CIs [34]. Heterogeneity was assessed using I^2^ statistics, with values categorized as low (25%), moderate (50%), or high (75%) [35]. Subgroup analysis was first performed by sex as the incidence of meningioma has a strong female predominance. Then, we performed a subgroup analysis by the method of exposure assessment (self-reported vs. measured) as there may be misclassification. Individuals, particularly the overweight or obese, may underreport the weight and overestimate the height. Publication bias was not assessed as less than ten studies were included.

In this study, our investigation first focused on comparing the highest vs. lowest levels of height, waist circumference, and WHR. BMI was initially assessed as a categorical variable. According to World Health Organization (WHO) guidelines, we divided BMI into four categories: underweight (less than 18.5 kg/m^2^), normal category (18.5 to less than 25 kg/m^2^), overweight (25 to less than 30 kg/m^2^), and obesity (greater than or equal to 30 kg/m^2^). For studies performed in Asian countries, the categories were defined as underweight (<18.5 kg/m^2^), normal category (18.5 to less than 23 kg/m^2^), overweight (23 to less than 27.5 kg/m^2^), and obese (greater than or equal to 27.5 kg/m^2^) [36,37]. When the standard cut-off point was not adopted, we matched study categories to the standard cut-off point. When an included study reported several risk estimates that fell into the same range representing overweight or obesity, we combined these RRs with the fixed-effect model before pooling them with other studies [38,39]. For example, the Iowa Women’s Health Study provided risk estimates for BMI categories of 30.0–34.9 kg/m² and 35.0 + kg/m² [7], both of which are classified as obesity (≥30 kg/m²) [7]. Initially, we combined these two risk estimates into a single risk estimate using a fixed-effect model, and subsequently combined it with risk estimates from other studies. Additionally, we employed a dose-response meta-analysis with a one-stage robust error meta-regression (REMR) model introduced by Doi [39,40]. This method requires reporting risk estimates and 95% CIs for at least two categories in each study. When the median or mean data of the BMI category was not shown, we used the midpoint of the range of the BMI category. When the highest or lowest BMI category is open, it was presumed that the width is the same as the interval between adjacent categories.

## Results

### Study selection

**Fig 1:**
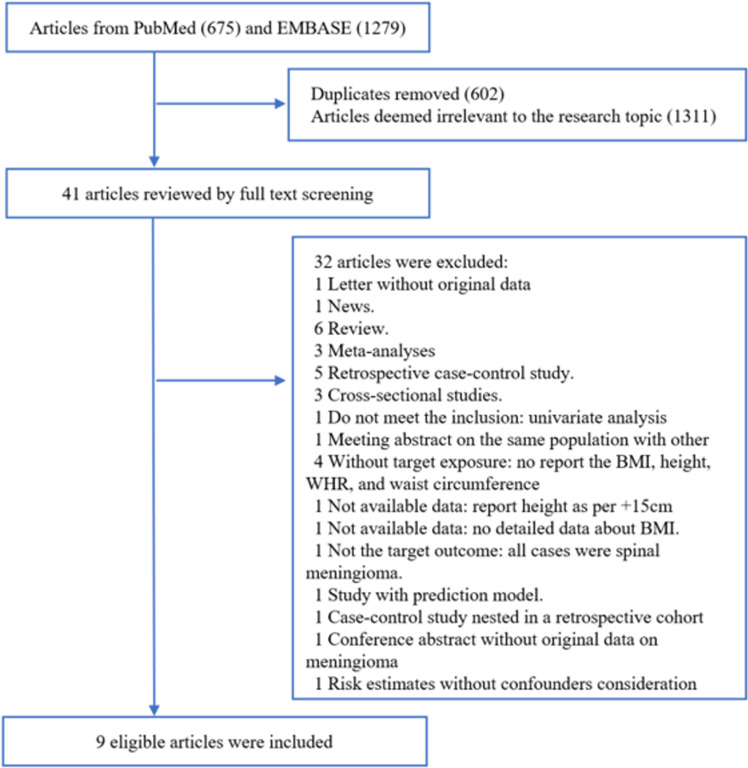
Flowchart of study selection in the meta-analysis.

To transparently illustrate the study selection process, Fig 1 outlines the complete workflow from initial database searches to final inclusion. Initially, we identified 1954 articles from PubMed and Embase databases. After using the EndNote “Find Duplicates” tool (Vision 21, Thomson Reuters, New York, NY, USA) and screening the titles and abstracts, we excluded 1913 studies: the duplicates (n = 602) and 1311 studies deemed irrelevant to the research topic. Of the remaining 41 articles selected for full-text review, 32 were excluded based on predefined eligibility criteria (detailed exclusion reasons provided in S2 Table). Finally, nine articles published from 2003 to 2020 were included in the current study [5–13].

### Study characteristics

To systematically assess heterogeneity across included studies, Table 1 and S3 Table summarizes key characteristics of the nine articles, including study design, population size, geographic distribution, case numbers, and other anthropometric factors, enabling readers to evaluate data representativeness and methodological variability. Overall, 8 cohorts [5–11,13] and 1 case-control study nested in a prospective cohort [12] involved 4,661,470 participants, and 5,609 meningiomas were diagnosed. These studies were performed in Japan [13], the USA [5,7,12], the United Kingdom [6,8], Denmark [8], Norway [8–11], Sweden [8,9], France [8], the Netherlands [8], Spain [8], Italy [8], Germany [8], and Greece [8]. The meningioma cases were ascertained by self-report or medical records (mainly using the International Classification of Diseases). Anthropometric factors, including BMI, height, waist circumference, and hip circumference, were assessed through self-report [5–7,12,13] or measurements [8–11]. A total of nine studies assessed the relationship between meningioma risk and BMI [5–13]. Most studies adopted the WHO guidelines to define overweight and obesity [6–8,10–13]. The reference categories of BMI among the included studies were not quite consistent. Specifically, 2 studies used normal weight (18.5 to less than 25 kg/m^2^ or 18.5–23 kg/m^2^ for Asians) [7,13], 3 used a range of normal weight (20 to less than 25 kg/m^2^) [8,10,11], 2 studies used both underweight and normal weight (<25 kg/m^2^) [6,12], one study used less than 22 kg/m^2^ [5], and one study used a mean BMI of 21.0 kg/m^2^ as reference level [9]. For height, waist circumference, and WHR, all included studies performed a “higher vs. lower’ comparison, and thus, the reference levels were different [6–8,10–13]. Except the Nurses’ Health Study cohort [5], all other studies awarded 7 or 8 stars are considered high-quality studies (S4 Table).

**Table 1:** Characteristics of the included studies in this meta-analysis.

Study	Country^a^	Study name	Study duration	Age at baseline	Sex	Cases/Cohort	Case classification criteria	Exposure Measurement
Jhawar et al. 2003 [5]	1	NHS	1976–1996/10 (mean)	30–55	Female	125/121,700	Self-reported and medical record	Self-reported
Benson et al. 2008 [6]	2	MWS	6.2 (mean)	50–65	Female	390/1,249,670	ICD-10, ICD-O-3	Self-reported
Johnson et al. 2011 [7]	1	IWHS	10.5 (mean)	65–84.6	Female	125/291,021	ICD-9	Self-reported
Michaud et al. 2011 [8]	2–11	EPIC	8.4 (mean)	35–70	Both	203/380,775	ICD-O-2	Measured
Edlinger et al. 2012 [9]	4,5	Me–Can	9.6 (median)	15–99	Both	348/418,578	ICD-7	Measured
Wiedmann et al. 2013 [10]	4	HUNT	23.5 (median)	≥20	Both	138/74,242	ICD-7, ICD-O-3	Measured
Wiedmann et al. 2017 [11]	4	NMRS	33.4 (median)	14–80	Both	3,335/1,855,333	ICD-7, ICD-O-3	Measured
Muskens et al. 2019 [12]	1	MEC	1993–2015	45–78	Both	894/167,226	ICD-9, 10	Self-reported
Ogawa et al. 2020 [13]	12	JPHC	18.1 (mean)	40–69	Both	51/102,925	ICD-O-3	Self-reported

NHS, Nurses’ Health Study cohort; MWS, Million Women Study cohort; IWHS, Iowa Women’s Health Study; EPIC, European Prospective Investigation into Cancer and Nutrition; Me-Can, Metabolic Syndrome and Cancer Project; HUNT Study, Nord–Trøndelag Health Study; NMRS, National Mass Radiography Service; MEC, Multiethnic Cohort; JPHC, Japan Public Health Centerebased Prospective Study; ICD, International Classification of Diseases; ICD-O, International Classification of Diseases-Oncology

^a^Studies were conducted in: (1) USA, (2) United Kingdom, (3) Denmark, (4) Norway, (5) Sweden, (6) France, (7) Netherlands, (8) Spain, (9) Italy, (10) Germany, (11) Greece, and (12) Japan.

### BMI and meningioma

**Fig 2:**
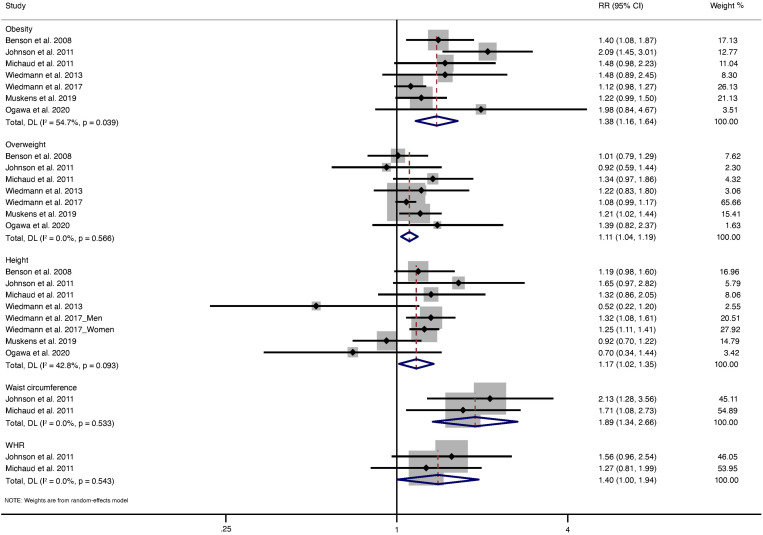
Forest plots for the relationship between meningioma risk and BMI, height, waist circumference, and WHR. BMI, body mass index; WHR, waist to hip ratio.

The relationship between BMI and meningioma risk was assessed in seven studies [6–8,10–13]. **Fig 2** shows the study-specific RRs and 95% CIs and pooled results for obesity and overweight. The pooled results showed that both obesity (RR: 1.38, 95% CI:1.16, 1.64; *P* < 0.001, I^2^ = 54.7%) and overweight (RR:1.11, 95% CI: 1.04, 1.19; *P* = 0.003, I^2^ = 0.0%) was associated with an increased risk of meningioma. Two studies used BMI of < 25 kg/m^2^ as the reference level [6,12], thus the reference level may include both underweight (<18.5 kg/m^2^) and normal weight (18.5 to less than 25 kg/m^2^). To maintain consistency, we further excluded two studies from sensitivity analysis. Correspondingly, the pooled results were not significantly changed (RR: 1.50, 95% CI:1.11, 2.03; *P* = 0.008, I^2^ = 68.1% for obesity; RR:1.11, 95% CI: 1.04, 1.19; *P* = 0.003, I^2^ = 0.0% for overweight). Subgroup analyses by sex and exposure assessment indicated no significant difference between these subgroups (**Table 2**).

**Table 2:** Summary results for the association between body mass index and meningioma risk.

Group	Overweight		Obesity		Height	
	Pooled RR with 95% CI and P	I^2^ test for Heterogeneity	Pooled RR with 95% CI and P	I^2^ test for Heterogeneity	Pooled RR with 95% CI and P	I^2^ test for Heterogeneity
All studies	1.11(1.04,1.19) 0.003	0.0%	1.38 (1.16, 1.64) <0.001	54.7%	1.07 (0.84,1.36) 0.580	46.6%
Sex						
Male	1.17(1.03,1.34) 0.019	0.0%	1.34 (1.04,1.72) 0.025	51.9%	1.30 (1.08,1.56) 0.005	0.0%
Female	1.09(1.00,1.17) 0.042	0.0%	1.35 (1.13,1.61) 0.001	0.0%	1.13 (0.94,1.36) 0.186	49.8%
Exposure assessment						
Self-reported	1.13(0.99,1.29) 0.061	0.0%	1.51 (1.16,1.95) 0.002	56.7%	1.09 (0.84,1.41) 0.533	47.3%
Measured	1.10(1.02,1.19) 0.019	0.0%	1.21 (1.01,1.45) 0.038	20.5%	0.89 (0.36,2.20) 0.804	72.8%

**Fig 3:**
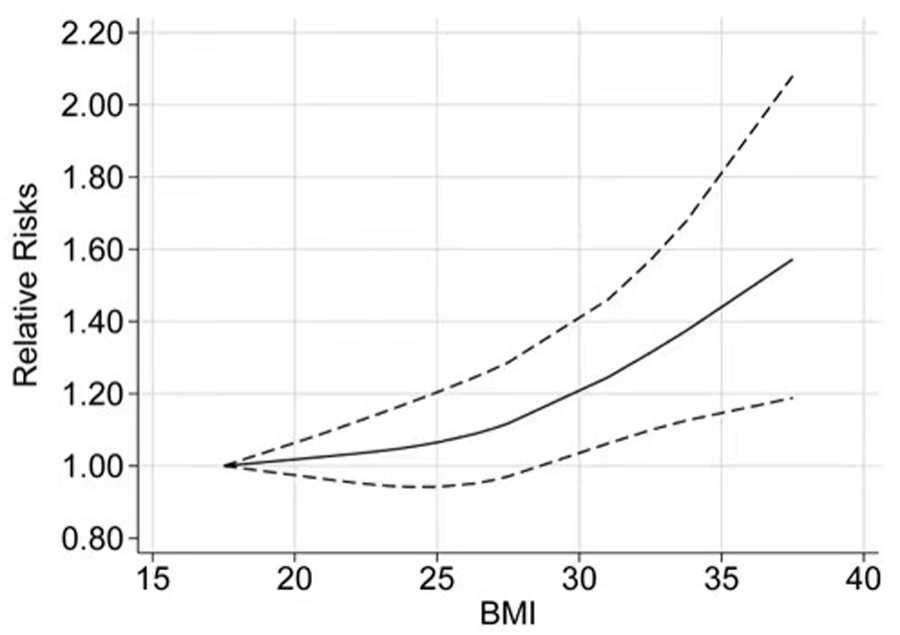
Dose-response relationships between BMI and meningioma risk. BMI, body mass index.

According to the REMR model, nine studies were included in the dose-response meta-analysis [5–13]. We found a nonlinear relationship between BMI and meningioma risk (**Fig 3**, *P* = 0.038).

### Height and meningioma

Seven studies have assessed the relationship between height and meningioma risk [6–8,10–13]. Notably, one of the seven included studies did not provide results for the overall population but provided a stratified result by sex [11]. We conducted a comparative analysis of the highest vs. lowest level of height (**Fig 2**). The pooled results showed no significant association between height and meningioma risk (RR:1.07, 95% CI:0.84, 1.36; *P* = 0.58, I^2^ = 46.6%). Subsequently, subgroup analysis by sex and the method of exposure assessment revealed a significant association between height and meningioma risk in men (RR:1.30, 95% CI:1.08, 1.56; *P* = 0.005, I^2^ = 0.0%) but not women (RR:1.13, 95% CI:0.94, 1.36; *P* = 0.186, I^2^ = 49.8%), self-reported (RR:1.09, 95% CI:0.84, 1.41; *P* = 0.533, I^2^ = 47.3%), or height-measured group (RR:0.89, 95% CI:0.36, 2.20; *P* = 0.804, I^2^ = 72.8%; **Table 2**).

### Waist circumference, WHR, and meningioma

Two studies have addressed the association between meningioma risk, waist circumference, and WHR [7,8]. In a comparison of highest versus lowest level of waist circumference and WHR, pooled results showed that greater waist circumference (RR:1.89, 95% CI:1.34,2.66; *P* < 0.001, I^2^ = 0.0%) and WHR (RR:1.40, 95% CI:1.00,1.94; *P* = 0.048, I^2^ = 0.0%) was associated with an increased risk of meningioma (**Fig 2**).

## Discussion

In the present meta-analysis of nine prospective studies, we found taller height (in men), increased BMI, waist circumference, and WHR were associated with an increased risk of meningioma. Dose-response meta-analysis showed a nonlinear relationship between BMI and meningioma risk.

Height has recently emerged as a critical anthropometric parameter in cancer risk research. The biological underpinnings of this relationship may lie in growth hormone and insulin-like growth factor (IGF-I and IGF-II) signaling during childhood and adolescence [41–43]. In prospective observational studies, the associations between elevated levels of these hormones and increased risks of prostate, breast, and colorectal cancers have been demonstrated [41]. Moreover, IGF1 signaling is established to upregulate oncogene expression and enhance tumor cell proliferation in vitro [41,44]. As for meningioma, existing studies demonstrate heterogeneous associations, with most reporting nonsignificant inverse or positive correlations [5–8,10,12–14] and a single outlier study suggesting contradictory findings [11]. After pooling these results, the overall finding showed no significant association between height and meningioma risk. However, subgroup analysis by sex identified a significant positive association in men but not women. Similarly, sex disparities in several cancer sites have been observed for height [45,46]. Notably, the apparent differences in height between men and women exist. Thus, using the same cut-off values of height for women and men may mask the genuine association, and further studies should do a separate analysis by sex and use the different cut-off values of height.

Waist circumference and WHR have recently gained significant considerable concerns as these two body size indicators reflect the body’s fat amount and the distribution of fat mass [47–49], and the significance of BMI in relation to body composition may differ depending on factors such as age, sex, and ethnicity [47]. For example, compared to white individuals, African Americans typically exhibit greater lean body mass and reduced levels of visceral fat. In contrast, Asians are generally characterized by lower lean body mass and higher amounts of visceral fat [47].To our knowledge, two prospective cohort studies assessed the relationship between waist circumference, WHR, and meningioma risk [7,8]. Both of them showed that greater waist circumference is associated with an increased risk of meningioma, with an HR of 2.13 (95% CI: 1.28–3.56) in the Iowa Women’s Health Study and an HR of 1.71 (1.08–2.73) in European Prospective Investigation into Cancer and Nutrition (EPIC) for Q4 vs. Q1 categories [7,8]. Additionally, a linear trend was also identified (*P* for trend = 0.01) [8]. Concerning WHR, a nonsignificant positive association was reported in previous studies [7,8]. Given that the power of a single study, we pooled data from the Iowa Women’s Health Study and EPIC study. The final results showed a significant positive association between meningioma risk and waist circumference and WHR in comparing the highest vs. lowest model. Moreover, we failed to perform a dose-response relationship between meningioma risk and waist circumference, and WHR due to the limited studies included in the current meta-analysis. Therefore, the relationship between meningioma risk and waist circumference, and WHR warrants further study.

Several meta-analyses and/or systematic reviews have addressed the relationship between BMI and meningioma risk [29–32]. The first two meta-analyses published in 2014 and 2015 found an increased risk of meningioma was associated with obesity but not overweight [29,30]. Another two meta-analyses published in 2015 and 2016 found that overweight and obesity were associated with an increased risk of meningioma [31,32]. Notably, retrospective case-control studies were included in all previous meta-analyses. Recall and selection bias are significant concerns. In the current study, our major findings are generally consistent with those of the latest meta-analysis published in 2016. However, the disparities between ours and previous studies are remarkable: (1) We only included prospective studies, and thus, our finding was less prone to recall and selection bias and reverse causation; (2) Our results were robust in subgroup analyses; (3) We adopted a one-stage REMR approach to perform a dose-response meta-analysis [40]; (4) We evaluated the association between meningioma risk and height, waist circumference, and WHR in this meta-analysis for the first time.

Multiple mechanisms have been proposed to explain the elevated meningioma risk in individuals with obesity. First, adipose tissue-driven estrogen excess may contribute to tumorigenesis [50–52], supported by evidence that sex steroid hormone receptors (e.g., estrogen receptors expressed in 48% of meningiomas [53]) mediate hormonal effects. In vitro studies confirm progesterone and estrogen stimulate meningioma cell proliferation [54,55], while epidemiologic data reveal a female predominance in incidence [1], collectively implicating estrogen in pathogenesis. Second, adiposity-associated insulin resistance and hyperinsulinemia may activate tumor-promoting IGF pathways [50–52]. This hypothesis was supported by the Norwegian prospective cohort data, which demonstrated an elevated meningioma risk in individuals with diabetes mellitus or glucose intolerance [56]. Additional mechanisms involve chronic inflammation, oxidative stress, adipocytokine dysregulation, ectopic fat-derived factors, microenvironmental perturbations, gut microbiome alterations, mechanical stress, and circadian/dietary disruptions [50–52,57,58].

Meningiomas account for 41.7% of all CNS tumors, presenting a significant public health priority due to their substantial disease burden. Notably, identifying modifiable risk factors for meningioma pathogenesis is critical to reducing this burden. In our study, elevated height (specifically in males), higher BMI, increased waist circumference, and elevated WHR demonstrated significant associations with meningioma risk. These findings indicate that anthropometric parameters—including height in males, BMI, waist circumference, and WHR—may serve as practical biomarkers for meningioma risk stratification. Clinicians should consider incorporating these indices into risk assessment frameworks, particularly for individuals with obesity or abnormal WHR. Furthermore, public health initiatives should prioritize population-level interventions targeting weight management and body fat reduction as primary preventive strategies.

Our meta-analysis also had some limitations. First, residual confounders’ effect on the association is still a significant concern, although all included studies have adjusted for various confounders. For example, excess weight or obesity may be associated with unhealthy lifestyles, such as less exercise, which was not adjusted in included studies. Second, we failed to perform a dose-response relationship between height, waist circumference, WHR, and meningioma risk as many studies did not provide detailed data about height categories, and only two studies were available for waist circumference and WHR. Third, the potential publication bias was not addressed due to the limited power of few included studies. Finally, the current meta-analysis incorporated only one study from Japan, with the remaining study populations exclusively derived from Western countries (United States and European nations). Given the limited geographical diversity of the included evidence, the external validity of these findings requires further confirmation in Asian populations and other ethnic groups.

## Conclusion

Based on the prospective evidence, this meta-analysis comprehensively evaluated the relationship between meningioma risk and several body size indicators and provided a more reliable estimation. In summary, excess weight (defined as BMI) and body fat mass (defined as waist circumference and WHR) were associated with an increased risk of meningioma. In particular, there was a nonlinear relationship between BMI and meningioma risk. Moreover, we found that greater height was associated with a risk of developing a meningioma in men, not women. Further prospective studies with particular attention to sex disparity and dose-response analysis are warranted to confirm our observation.

## Supporting information

S1 Table Database search.
(DOCX)

S2 Table Studies with exclusion reasons. (DOCX)

S3 Table Additional basic characteristic of included studies. (DOCX)

S4 Table Risk bias of included studies based on the Newcastle–Ottawa Scale. (DOCX)
