# Supplementary material for: Association between anthropometric factors and meningioma risk: A systematic review and meta-analysis
Source: PLoS One. 2025 May 13;20(5):e0323461. doi: 10.1371/journal.pone.0323461 (PMC12074524; doi:10.1371/journal.pone.0323461)
Supplement: S1 Table — Database search. (DOCX) [file pone.0323461.s001.docx]

**S1 Table Database search**

| PubMed |  |  |  |  | Embase |  |  |  |
| --- | --- | --- | --- | --- | --- | --- | --- | --- |
| Search | Query | Items found | Date |  | Search | Query | Items found | Date |
| #1 | Search body mass index[Title/Abstract] | 257,634 | 2024.09.07 |  | #1 | Search 'body mass index':ab,ti | 369,453 | 2024.09.07 |
| #2 | Search obesity[Title/Abstract] | 348,531 | 2024.09.07 |  | #2 | Search 'obesity':ab,ti | 493,001 | 2024.09.07 |
| #3 | Search overweight[Title/Abstract] | 95,341 | 2024.09.07 |  | #3 | Search 'overweight':ab,ti | 142,508 | 2024.09.07 |
| #4 | Search underweight[Title/Abstract] | 16,263 | 2024.09.07 |  | #4 | Search 'underweight':ab,ti | 24,856 | 2024.09.07 |
| #5 | Search weight[Title/Abstract] | 1,032,088 | 2024.09.07 |  | #5 | Search 'weight':ab,ti | 1,448,202 | 2024.09.07 |
| #6 | Search adiposity[Title/Abstract] | 32,868 | 2024.09.07 |  | #6 | Search 'adiposity':ab,ti | 45,605 | 2024.09.07 |
| #7 | Search obese[Title/Abstract] | 158,242 | 2024.09.07 |  | #7 | Search 'obese':ab,ti | 251,979 | 2024.09.07 |
| #8 | Search anthropometry[Title/Abstract] | 14,089 | 2024.09.07 |  | #8 | Search 'anthropometry':ab,ti | 16,053 | 2024.09.07 |
| #9 | Search height[Title/Abstract] | 208,125 | 2024.09.07 |  | #9 | Search 'height':ab,ti | 284,509 | 2024.09.07 |
| #10 | Search BMI[Title/Abstract] | 213,260 | 2024.09.07 |  | #10 | Search 'bmi':ab,ti | 444,793 | 2024.09.07 |
| #11 | Search waist circumference[Title/Abstract] | 37,046 | 2024.09.07 |  | #11 | Search 'waist circumference':ab,ti | 57,971 | 2024.09.07 |
| #12 | Search hip circumference[Title/Abstract] | 3,822 | 2024.09.07 |  | #12 | Search 'hip circumference':ab,ti | 5,969 | 2024.09.07 |
| #13 | Search waist to hip ratio[Title/Abstract] | 7,403 | 2024.09.07 |  | #13 | Search 'waist to hip ratio':ab,ti | 9,899 | 2024.09.07 |
| #14 | Search WHR[Title/Abstract] | 6,159 | 2024.09.07 |  | #14 | Search 'whr':ab,ti | 9,094 | 2024.09.07 |
| #15 | Search #1 OR #2 OR #3 OR #4 OR #5 OR #6 OR #7 OR #8 OR #9 OR #10 OR #11 OR #12 OR #13 OR #14 | 1,644,347 | 2024.09.07 |  | #15 | Search #1 OR #2 OR #3 OR #4 OR #5 OR #6 OR #7 OR #8 OR #9 OR #10 OR #11 OR #12 OR #13 OR #14 | 2,380,250 | 2024.09.07 |
| #16 | Search meningioma[Title/Abstract] | 20,085 | 2024.09.07 |  | #16 | Search 'meningioma':ab,ti | 25,497 | 2024.09.07 |
| #17 | Search brain cancer[Title/Abstract] | 5,328 | 2024.09.07 |  | #17 | Search 'brain cancer':ab,ti | 7,212 | 2024.09.07 |
| #18 | Search brain tumor[Title/Abstract] | 28,414 | 2024.09.07 |  | #18 | Search 'brain tumor':ab,ti | 39,308 | 2024.09.07 |
| #19 | Search brain neoplasms[Title/Abstract] | 6,048 | 2024.09.07 |  | #19 | Search 'brain neoplasms':ab,ti | 933 | 2024.09.07 |
| #20 | Search central nervous system neoplasms[Title/Abstract] | 554 | 2024.09.07 |  | #20 | Search 'central nervous system neoplasms':ab,ti | 447 | 2024.09.07 |
| #21 | Search #16 OR #17 OR #18 OR #19 OR #20 | 57,288 | 2024.09.07 |  | #21 | Search #16 OR #17 OR #18 OR #19 OR #20 | 70,534 | 2024.09.07 |
| #22 | Search #15 AND #21 | 932 | 2024.09.07 |  | #22 | Search #15 AND #21 | 1,564 | 2024.09.07 |
| #23 | Search #22: limited to Humans | 675 | 2024.09.07 |  | #23 | Search #22 AND [humans]/lim | 1,279 | 2024.09.07 |
