# Supplementary material for: Association between anthropometric factors and meningioma risk: A systematic review and meta-analysis
Source: PLoS One. 2025 May 13;20(5):e0323461. doi: 10.1371/journal.pone.0323461 (PMC12074524; doi:10.1371/journal.pone.0323461)
Supplement: S2 Table — (DOCX) [file pone.0323461.s002.docx]

**S2 Table. Studies excluded with reasons (N=1945).**

| **Number** | **Studies excluded as they were duplicates（N=602）** |
| --- | --- |
| 1 | da Silveira EF, Ferreira LM, Gehrcke M, Cruz L, Pedra NS, Ramos PT, et al. 2-(2-Methoxyphenyl)-3-((Piperidin-1-yl)ethyl)thiazolidin-4-One-Loaded Polymeric Nanocapsules: In Vitro Antiglioma Activity and In Vivo Toxicity Evaluation. Cellular and molecular neurobiology. 2019;39(6):783-97. |
| 2 | Li W, Liu J, Fu W, Zheng X, Ren L, Liu S, et al. 3-O-acetyl-11-keto-β-boswellic acid exerts anti-tumor effects in glioblastoma by arresting cell cycle at G2/M phase. Journal of Experimental and Clinical Cancer Research. 2018;37(1). doi: 10.1186/s13046-018-0805-4. |
| 3 | Poole N, Schwab J, Hageman J. A 9-year-old male with hypertension. Pediatric annals. 2015;44(1):e10-3. Epub 2015/01/27. doi: 10.3928/00904481-20151226-09. PubMed PMID: 25621628. |
| 4 | Johnson AH, Rodgers Phillips S, Rice M. Abnormal weight gain with fatigue and stress in early survivorship after childhood brain tumor diagnosis. Journal for specialists in pediatric nursing : JSPN. 2020;25(3):e12288. doi: 10.1111/jspn.12288. |
| 5 | Blumberg DL, Sklar CA, Wisoff J, David R. Abnormalities of water metabolism in children and adolescents following craniotomy for a brain tumor. Child's Nervous System. 1994;10(8):505-8. doi: 10.1007/BF00335072. |
| 6 | Yue Q, Shibata Y, Isobe T, Anno I, Kawamura H, Gong QY, et al. Absolute choline concentration measured by quantitative proton MR spectroscopy correlates with cell density in meningioma. Neuroradiology. 2009;51(1):61-7. doi: 10.1007/s00234-008-0461-z. |
| 7 | Yu J, Zheng J, Xu W, Weng J, Gao L, Tao L, et al. Accuracy of 18F-FDOPA Positron Emission Tomography and 18F-FET Positron Emission Tomography for Differentiating Radiation Necrosis from Brain Tumor Recurrence. World neurosurgery. 2018;114:e1211-e24. doi: 10.1016/j.wneu.2018.03.179. |
| 8 | Van Roessel IMAA, De Graaf JP, Biermasz NR, Charmandari E, Van Santen HM. Acquired Hypothalamic dysfunction in childhood: “What do patients need?” - an Endo-ERN survey. Hormone research in paediatrics. 2023;96:377. doi: 10.1159/000533803. |
| 9 | Ali BH, Khoee S, Mafakheri F, Sadri E, Mahabadi VP, Karimi MR, et al. Active targeted delivery of theranostic thermo/pH dual-responsive magnetic Janus nanoparticles functionalized with folic acid/fluorescein ligands for enhanced DOX combination therapy of rat glioblastoma. Journal of materials chemistry B. 2024;12(24):5957-73. |
| 10 | Losonczy H, Nagy Á, Tar A. [Actual questions about the prevention of venous thromboembolism in cancer patients receiving chemotherapy]. Orvosi hetilap. 2016;157(6):203-11. Epub 2016/04/28. doi: 10.1556/650.2016.30357. PubMed PMID: 27120721. |
| 11 | Conklin HM, Khan RB, Reddick WE, Helton S, Brown R, Howard SC, et al. Acute neurocognitive response to methylphenidate among survivors of childhood cancer: A randomized, double-blind, cross-over trial. Journal of pediatric psychology. 2007;32(9):1127-39. doi: 10.1093/jpepsy/jsm045. |
| 12 | Faienza MF, Delvecchio M, Indrio F, Francavilla R, Acquafredda A, Cavallo L. Acute pancreatitis in a girl with panhypopituitarism due to craniopharyngioma on growth hormone treatment. A combination of risk factors. Hormone research. 2009;71(6):372-5. doi: 10.1159/000223423. |
| 13 | Beckers D, Thomas M, Jamart J, Francois I, Maes M, Lebrethon MC, et al. Adult final height after GH therapy for irradiation-induced GH deficiency in childhood survivors of brain tumors: The Belgian experience. European journal of endocrinology. 2010;162(3):483-90. doi: 10.1530/EJE-09-0690. |
| 14 | Little RB, Nabors LB, Olson JJ, Thompson ZJ, Madden MH, LaRocca RV, et al. Age at attainment of adult height and risk of primary brain tumors. Cancer research. 2014;74(19). doi: 10.1158/1538-7445.AM2014-2164. |
| 15 | Denizot Y, De Armas R, Durand K, Robert S, Moreau JJ, Caire F, et al. Analysis of several PLA2 mRNA in human meningiomas. Mediators of inflammation. 2009;2009. doi: 10.1155/2009/689430. |
| 16 | Borghei-Razavi H, Raghavan A, Eguiluz-Melendez A, Joshi K, Fernandez-Miranda JC, Kshettry VR, et al. Anatomical Variations in the Location of Veins Draining into the Anterior Superior Sagittal Sinus: Implications for the Transbasal Approach. Operative Neurosurgery. 2020;18(6):668-75. |
| 17 | Tsai YT, Ko CY, Chang WC, Hsu TI. ANGPTL4 Induces TMZ Resistance of Glioblastoma by Promoting Cancer Stemness Enrichment via the EGFR/AKT/4E-BP1 Cascade. FASEB Journal. 2020;34(SUPPL 1). doi: 10.1096/fasebj.2020.34.s1.02019. |
| 18 | Tsai YT, Wu AC, Yang WB, Kao TJ, Chuang JY, Chang WC, et al. ANGPTL4 Induces TMZ Resistance of Glioblastoma by Promoting Cancer Stemness Enrichment via the EGFR/AKT/4E-BP1 Cascade. International journal of molecular sciences. 2019;20(22). |
| 19 | Tamminga RYJ, Kamps WA, Humphrey GB, Drayer NM. Anthropometric measurements at diagnosis of childhood cancer. Pediatric hematology and oncology. 1990;7(3):243-51. |
| 20 | Michaud DS, Bové G, Gallo V, Schlehofer B, Tjønneland A, Olsen A, et al. Anthropometric measures, physical activity, and risk of glioma and meningioma in a large prospective cohort study. Cancer Prevention Research. 2011;4(9):1385-92. doi: 10.1158/1940-6207.CAPR-11-0014. |
| 21 | Hawasli AH, Rubin JB, Tran DD, Adkins DR, Waheed S, Hullar TE, et al. Antiangiogenic agents for nonmalignant brain tumors. Journal of Neurological Surgery, Part B: Skull Base. 2013;74(3):136-41. doi: 10.1055/s-0033-1338262. |
| 22 | Mantia C, Zwicker JI. Anticoagulation in the Setting of Primary and Metastatic Brain Tumors. Cancer treatment and research. 2019;179:179-89. Epub 2019/07/19. doi: 10.1007/978-3-030-20315-3_12. PubMed PMID: 31317488. |
| 23 | McCutcheon IE, Flyvbjerg A, Hill H, Li J, Bennett WF, Scarlett JA, et al. Antitumor activity of the growth hormone receptor antagonist pegvisomant against human meningiomas in nude mice. Journal of neurosurgery. 2001;94(3):487-92. doi: 10.3171/jns.2001.94.3.0487. |
| 24 | Erdlenbruch B, Jendrossek V, Marx M, Hunold A, Eibl H, Lakomek M. Antitumor effects of erucylphosphocholine on brain tumor cells in vitro and in vivo. Anticancer research. 1998;18(4 A):2551-7. PubMed Central PMCID: Sigma(Germany). |
| 25 | Ferronato MJ, Alonso EN, Salomón DG, Fermento ME, Gandini NA, Quevedo MA, et al. Antitumoral effects of the alkynylphosphonate analogue of calcitriol EM1 on glioblastoma multiforme cells. Journal of Steroid Biochemistry and Molecular Biology. 2018;178:22-35. |
| 26 | Maravilla KR, Smith MP, Vymazal J, Goyal M, Herman M, Baima JJ, et al. Are there differences between macrocyclic gadolinium contrast agents for brain tumor imaging? Results of a multicenter intraindividual crossover comparison of gadobutrol with gadoteridol (The truth study). American Journal of Neuroradiology. 2015;36(1):14-23. |
| 27 | Gubernatorova E, Pavlova MG, Kaznacheeva T, Tselovalnikova T. Aseptic necrosis of femur in combination with secondary and adrenal insufficiency, primary hypogonadism in childhood medulloblastoma survivor. Osteoporosis International. 2016;27(SUPPL 1):S410-S1. doi: 10.1007/s00198-016-3530-x. |
| 28 | Ratnasinghe LD, Graubard BI, Kahle L, Tangrea JA, Taylor PR, Hawk E. Aspirin use and mortality from cancer in a prospective cohort study. Anticancer research. 2004;24(5 B):3177-84. |
| 29 | Bernardo BM, Orellana RC, Weisband YL, Hammar N, Walldius G, Malmstrom H, et al. Association between prediagnostic glucose, triglycerides, cholesterol and meningioma, and reverse causality. British journal of cancer. 2016;115(1):108-14. doi: 10.1038/bjc.2016.157. |
| 30 | Shahid S, Chaudary MA. Association of 25-hydroxyvitamin D with hematological profile and anthropometry in patients with glioma. Revista da Associacao Medica Brasileira. 2022;68(11):1547-52. doi: 10.1590/1806-9282.20220516. |
| 31 | Cole KL, Kazim SF, Thommen R, Alvarez-Crespo DJ, Vellek J, Conlon M, et al. Association of baseline frailty status and age with outcomes in patients undergoing intracranial meningioma surgery: Results of a nationwide analysis of 5818 patients from the National Surgical Quality Improvement Program (NSQIP) 2015–2019. European Journal of Surgical Oncology. 2022;48(7):1671-7. |
| 32 | Schneider CV, Schneider KM, Teumer A, Rudolph KL, Hartmann D, Rader DJ, et al. Association of Telomere Length with Risk of Disease and Mortality. JAMA internal medicine. 2022;182(3):291-300. doi: 10.1001/jamainternmed.2021.7804. |
| 33 | Shabo E, Wach J, Hamed M, Güresir Á, Weinhold L, Vatter H, et al. Asymptomatic Postoperative Cerebral Venous Sinus Thrombosis after Posterior Fossa Tumor Surgery: Incidence, Risk Factors, and Therapeutic Options. Neurosurgery. 2023;92(6):1171-6. doi: 10.1227/neu.0000000000002340. |
| 34 | Fròsina G, Profumo A, Marubbi D, Marcello D, Ravetti JL, Daga A. ATR kinase inhibitors NVP-BEZ235 and AZD6738 effectively penetrate the brain after systemic administration. Radiation Oncology. 2018;13(1). doi: 10.1186/s13014-018-1020-3. |
| 35 | Todo T, Adams EF, Fahlbusch R, Dingermann T, Werner H, Hinton DR. Autocrine growth stimulation of human meningioma cells by platelet- derived growth factor. Journal of neurosurgery. 1996;84(5):852-9. |
| 36 | Hervey-Jumper S, Li J, Lau D, Molinaro A, Perry D, Meng L, et al. Awake craniotomy to maximize glioma resection: Methods and technical nuances. Neuro-oncology. 2014;16:v135. doi: 10.1093/neuonc/nou263.6. |
| 37 | Hervey-Jumper SL, Li J, Lau D, Molinaro AM, Perry DW, Meng L, et al. Awake craniotomy to maximize glioma resection: Methods and technical nuances over a 27-year period. Journal of neurosurgery. 2015;123(2):325-39. doi: 10.3171/2014.10.JNS141520. |
| 38 | Pajecki D, Santo MA, Joaquim HD, Morita F, Riccioppo D, de Cleva R, et al. BARIATRIC SURGERY IN THE ELDERLY: RESULTS OF A MEAN FOLLOW-UP OF FIVE YEARS. Arquivos brasileiros de cirurgia digestiva : ABCD = Brazilian archives of digestive surgery. 2015;28:15-8. |
| 39 | Cappelli L, Uppendahl A, Gardner C, Khan M, Kayne A, Vemula S, et al. Baseline single institutional retrospective review of body mass index (BMI) as a prognostic indicator in patients with newly diagnosed glioblastoma (GBM). Journal of Clinical Neuroscience. 2024;127. |
| 40 | Vaneckova M, Herman M, Smith MP, Mechl M, Maravilla KR, Weichet J, et al. The benefits of high relaxivity for brain tumor imaging: Results of a multicenter intraindividual crossover comparison of gadobenate dimeglumine with gadoterate meglumine (the BENEFIT study). American Journal of Neuroradiology. 2015;36(9):1589-98. |
| 41 | Huang HW, Yan LM, Yang YL, He X, Sun XM, Wang YM, et al. Bi-frontal pneumocephalus is an independent risk factor for early postoperative agitation in adult patients admitted to intensive care unit after elective craniotomy for brain tumor: A prospective cohort study. PloS one. 2018;13(7). |
| 42 | Savitz DA, Ananth CV. Birth characteristics of childhood cancer cases, controls, and their siblings. Pediatric hematology and oncology. 1994;11(6):587-99. |
| 43 | Tettamanti G, Ljung R, Mathiesen T, Schwartzbaum J, Feychting M. Birth size characteristics and risk of brain tumors in early adulthood: Results from a swedish cohort study. Cancer Epidemiology Biomarkers and Prevention. 2016;25(4):678-85. doi: 10.1158/1055-9965.EPI-15-1096. |
| 44 | Harder T, Plagemann A, Harder A. Birth weight and subsequent risk of childhood primary brain tumors: A meta-analysis. American journal of epidemiology. 2008;168(4):366-73. doi: 10.1093/aje/kwn144. |
| 45 | Edlinger M, Strohmaier S, Jonsson H, Bjørge T, Manjer J, Borena WT, et al. Blood pressure and other metabolic syndrome factors and risk of brain tumour in the large population-based Me-Can cohort study. Journal of hypertension. 2012;30(2):290-6. doi: 10.1097/HJH.0b013e32834e9176. |
| 46 | Beccaria K, Sabbagh A, de Groot J, Canney M, Carpentier A, Heimberger AB. Blood-brain barrier opening with low intensity pulsed ultrasound for immune modulation and immune therapeutic delivery to CNS tumors. Journal of neuro-oncology. 2021;151(1):65-73. |
| 47 | Toyota P, Persad A, Liu E, Saini J, Zherebitskiy V, Auer R, et al. BMI as a Predictor of Recurrence in High-Grade Meningioma: A Single Center Retrospective Cohort Study. Journal of Neurological Surgery, Part B: Skull Base. 2024;85. doi: 10.1055/s-0044-1780240. |
| 48 | Boekhoff S, Peng J, Bison B, Eveslage M, Sowithayasakul P, Müller HL. Body Composition and Nuchal Skinfold Thickness in Pediatric Brain Tumor Patients. Pediatric Blood and Cancer. 2021;68(SUPPL 5). doi: 10.1002/pbc.29349. |
| 49 | Peng J, Boekhoff S, Eveslage M, Bison B, Sowithayasakul P, Muller HL. Body composition and nuchal skinfold thickness in pediatric brain tumor patients. Revista Argentina de Endocrinologia y Metabolismo. 2021;58(SUPPL 1):224. |
| 50 | Peng J, Boekhoff S, Eveslage M, Sowithayasakul P, Bison B, Muller HL. Body Composition and Nuchal Skinfold Thickness in Pediatric Brain Tumor Patients. Journal of the Endocrine Society. 2021;5:A624. doi: 10.1210/jendso/bvab048.1272. |
| 51 | Ogawa T, Sawada N, Iwasaki M, Budhathoki S, Yamaji T, Shimazu T, et al. Body mass index and height in relation to brain tumor risk in a Japanese population. Annals of epidemiology. 2020;51:1-6. doi: 10.1016/j.annepidem.2020.06.001. |
| 52 | Wiedmann M, Brunborg C, Lindemann K, Johannesen TB, Vatten L, Helseth E, et al. Body mass index and the risk of meningioma, glioma and schwannoma in a large prospective cohort study (The HUNT Study). British journal of cancer. 2013;109(1):289-94. doi: 10.1038/bjc.2013.304. |
| 53 | Garavaglia M, Mak T, Cusimano MD, Rigamonti A, Crescini C, McCredy V, et al. Body mass index as a risk factor for increased serum lactate during craniotomy. Minerva anestesiologica. 2013;79(10):1132-9. Epub 2013/05/24. PubMed PMID: 23698547. |
| 54 | van Roessel IMAA, van Schaik J, Meeteren AYNSV, Boot AM, der Grinten HLCV, Clement SC, et al. Body mass index at diagnosis of a childhood brain tumor; a reflection of hypothalamic-pituitary dysfunction or lifestyle? Supportive Care in Cancer. 2022;30(7):6093-102. |
| 55 | Liu Y, Hu H, Han Y, Li L, Li Z, Zhang L, et al. Body Mass Index Has a Nonlinear Association With Postoperative 30-Day Mortality in Patients Undergoing Craniotomy for Tumors in Men: An Analysis of Data From the ACS NSQIP Database. Frontiers in endocrinology. 2022;13. |
| 56 | Muskens IS, Wu AH, Porcel J, Cheng I, Le Marchand L, Wiemels JL, et al. Body mass index, comorbidities, and hormonal factors in relation to meningioma in an ethnically diverse population: The Multiethnic Cohort. Neuro-oncology. 2019;21(4):498-507. doi: 10.1093/neuonc/noz005. |
| 57 | Niedermaier T, Behrens G, Schmid D, Schlecht I, Fischer B, Leitzmann MF. Body mass index, physical activity, and risk of adult meningioma and glioma : A meta-analysis. Neurology. 2015;85(15):1342-50. doi: 10.1212/WNL.0000000000002020. |
| 58 | Da Silva AN, Heras-Herzig A, Schiff D. Bone health in patients with brain tumors. Surgical neurology. 2007;68(5):525-33. doi: 10.1016/j.surneu.2006.11.065. |
| 59 | Remes TM, Arikoski PM, Lähteenmäki PM, Arola MO, Pokka TML, Riikonen VP, et al. Bone mineral density is compromised in very long-term survivors of irradiated childhood brain tumor. Acta Oncologica. 2018;57(5):665-74. doi: 10.1080/0284186X.2018.1431401. |
| 60 | Al-Huniti A, Harshman LA, Novak M, Nopoulos P, Staber JM. Brain anomalies in children with severe factor VIII deficiency-a pilot study. Blood. 2019;134. doi: 10.1182/blood-2019-131804. |
| 61 | Bifulco M, Ranieri R, Ciaglia E. Brain cancer risk: the weight of obesity. Journal of neuro-oncology. 2017;131(2):421-2. doi: 10.1007/s11060-016-2315-6. |
| 62 | Timme M, Thomas C, Spille DC, Stummer W, Ebel H, Ewelt C, et al. Brain invasion in meningiomas: does surgical sampling impact specimen characteristics and histology? Neurosurgical review. 2020;43(2):793-800. doi: 10.1007/s10143-019-01125-0. |
| 63 | Selvi TK, Sumaiya Begum A, Poonkuzhali P, Aarthi R. Brain tumor classification for MRI images using dual-discriminator conditional generative adversarial network. Electromagnetic biology and medicine. 2024;43(1-2):81-94. |
| 64 | Lin L, Liao SC, Lee YJ, Tseng MC, Lee MB. Brain tumor presenting as anorexia nervosa in a 19-year-old man. Journal of the Formosan Medical Association. 2003;102(10):737-40. |
| 65 | Ranjbarzadeh R, Zarbakhsh P, Caputo A, Tirkolaee EB, Bendechache M. Brain tumor segmentation based on optimized convolutional neural network and improved chimp optimization algorithm. Computers in biology and medicine. 2024;168. doi: 10.1016/j.compbiomed.2023.107723. |
| 66 | Abecassis IJ, Smith T, Chandler JP. Brain tumors and the area postrema. Journal of Clinical Neuroscience. 2013;20(12):1795-7. doi: 10.1016/j.jocn.2013.01.028. |
| 67 | Abecassis IJ, Smith T, Chandler JP. Brain tumors and the area postrema. Journal of clinical neuroscience : official journal of the Neurosurgical Society of Australasia. 2013;20(12):1795-7. |
| 68 | Avliyakulov NK, Rajavel KS, Le KMT, Guo L, Mirsadraei L, Yong WH, et al. C-terminally truncated form of αb-crystallin is associated with IDH1 R132H mutation in anaplastic astrocytoma. Journal of neuro-oncology. 2014;117(1):53-65. doi: 10.1007/s11060-014-1371-z. |
| 69 | Jensen RL, Wurster RD. Calcium channel antagonists inhibit growth of subcutaneous xenograft meningiomas in nude mice. Surgical neurology. 2001;55(5):275-83. doi: 10.1016/S0090-3019(01)00444-X. |
| 70 | Kirman CR, Gargas ML, Marsh GM, Strother DE, Klaunig JE, Collins JJ, et al. Cancer dose--response assessment for acrylonitrile based upon rodent brain tumor incidence: use of epidemiologic, mechanistic, and pharmacokinetic support for nonlinearity. Regulatory toxicology and pharmacology : RTP. 2005;43(1):85-103. |
| 71 | Mulrooney DA, Yeazel MW, Kawashima T, Mertens AC, Mitby P, Stovall M, et al. Cardiac outcomes in a cohort of adult survivors of childhood and adolescent cancer: Retrospective analysis of the childhood cancer survivor study cohort. BMJ (Online). 2009;339(7736):34. doi: 10.1136/bmj.b4606. |
| 72 | Kayadjanian N, Hsu EA, Wood AM, Carson DS. Caregiver Burden and Its Relationship to Health-Related Quality of Life in Craniopharyngioma Survivors. Journal of Clinical Endocrinology and Metabolism. 2024;109(1):E76-E87. doi: 10.1210/clinem/dgad488. |
| 73 | Ikeda Y, Sakemi T, Shouno Y, Uchida M, Nagano Y, Kou T, et al. A case of polyarteritis nodosa who developed rapidly progressive glomerulonephritis and presented with a tumor-like shadow on head CT in the remission stage. Japanese Journal of Nephrology. 1994;36(9):1052-6. |
| 74 | Granbichler CA, Oberaigner W, Kuchukhidze G, Bauer G, Ndayisaba JP, Seppi K, et al. Cause-specific mortality in adult epilepsy patients from Tyrol, Austria: Hospital-based study. Journal of neurology. 2015;262(1):126-33. doi: 10.1007/s00415-014-7536-z. |
| 75 | Kim Y, Kumar S. CD44-mediated adhesion to hyaluronic acid contributes to mechanosensing and invasive motility. Molecular Cancer Research. 2014;12(10):1416-29. doi: 10.1158/1541-7786.MCR-13-0629. |
| 76 | Christensen K, Schrøder HD, Kristensen BW. CD133+ niches and single cells in glioblastoma have different phenotypes. Journal of neuro-oncology. 2011;104(1):129-43. doi: 10.1007/s11060-010-0488-y. |
| 77 | Yu W, Su Z, Wu Z, Mao X, Zheng W, Zeng Y. cDNA clone, prokaryotic expression and purification of human interleukin-13 receptor ά2 chain. Cancer Immunology, Immunotherapy. 2009;58(3):409-13. doi: 10.1007/s00262-008-0566-6. |
| 78 | Krysiak R, Marek B, Okopień B. Central precocious puberty. Endokrynologia Polska. 2008;59(6):530-40. |
| 79 | Han MJ, Kim SC, Joo CU, Kim SJ. Cerebral salt-wasting syndrome in a child with Wernicke encephalopathy treated with fludrocortisone therapy A case report. Medicine (United States). 2016;95(36). doi: 10.1097/MD.0000000000004393. |
| 80 | Kobayashi K, Suehiro M, Maihara T, Usami I, Kageyama Y, Okazaki S, et al. Cerebral Sinovenous Thrombosis and Subdural Hematoma as Treatment-Related Complications in Suprasellar Germ Cell Tumor Associated with Adipsic Diabetes Insipidus. Pediatric neurosurgery. 2019;54(4):288-92. |
| 81 | Yanardag H, Uygun S, Yumuk V, Caner M, Canbaz B. Cerebral tuberculosis mimicking intracranial tumour. Singapore medical journal. 2005;46(12):731-3. |
| 82 | Patel SI, Obeid H, Matti L, Ramakrishna H, Shamoun FE. Cerebral venous thrombosis: Current and newer anticoagulant treatment options. The neurologist. 2015;20(5):80-8. doi: 10.1097/NRL.0000000000000049. |
| 83 | Guntner AS, Peyrl A, Mayr L, Englinger B, Berger W, Slavc I, et al. Cerebrospinal fluid penetration of targeted therapeutics in pediatric brain tumor patients. Acta neuropathologica communications. 2020;8(1):78. doi: 10.1186/s40478-020-00953-2. |
| 84 | Iyengar V, Patell R, Zwicker J. Challenges in anticoagulation for patients with brain tumors. Best Practice and Research: Clinical Haematology. 2022;35(1). doi: 10.1016/j.beha.2022.101350. |
| 85 | Panagopoulou P, Sattar S, Aquilina K, Jan W, Jacques T, Slater O. Challenges in the Diagnosis of Medulloblastoma Recurrence at an Unusual Site in a Patient with Prader-Willi Syndrome. Journal of pediatric hematology/oncology. 2020;42(5):e381-e4. doi: 10.1097/MPH.0000000000001555. |
| 86 | Isobe T, Matsumura A, Anno I, Nagatomo Y, Yoshizawa T, Itai Y, et al. Changes in 1H-MRS in glioma patients before and after irradiation: The significance of quantitative analysis of choline-containing compounds. Neurological Surgery. 2003;31(2):167-72. |
| 87 | Brinksma A, Sulkers E, Kouwenberg D, Lelieveld O, Boot AM, Burgerhof JGM, et al. Changes in body size and body composition in survivors of childhood cancer: seven years follow-up of a prospective cohort study. Clinical nutrition (Edinburgh, Scotland). 2022;41(12):2778-85. |
| 88 | Woolf SH, Chapman DA, Buchanich JM, Bobby KJ, Zimmerman EB, Blackburn SM. Changes in midlife death rates across racial and ethnic groups in the United States: Systematic analysis of vital statistics. BMJ (Online). 2018;362. doi: 10.1136/bmj.k3096. |
| 89 | Yoshida K, Furuse M, Kaneoke Y, Sasou K, Motegi Y. [Changes in tissue T1 of brain tumor following GD-DTPA administration--differences in T1 time course and tissue-blood ratio by histological types]. No to shinkei = Brain and nerve. 1988;40(6):531-7. Epub 1988/06/01. PubMed PMID: 3224029. |
| 90 | Moriuchi S, Shimizu K, Miyao Y, Hayakawa T. Characterisation of a new mouse monoclonal antibody (ONS-M21) reactive with both medulloblastomas and gliomas. British journal of cancer. 1993;68(5):831-7. |
| 91 | Spirollari E, Vazquez S, Das A, Wang R, Ampie L, Carpenter AB, et al. Characteristics of Patients Selected for Surgical Treatment of Spinal Meningioma. World neurosurgery. 2022;165:e680-e8. doi: 10.1016/j.wneu.2022.06.121. |
| 92 | Nuñez-delMoral A, Brocos-Mosquera I, Vialou V, Callado LF, Erdozain AM. Characterization of Hevin (SPARCL1) Immunoreactivity in Postmortem Human Brain Homogenates. Neuroscience. 2021;467:91-109. doi: 10.1016/j.neuroscience.2021.05.017. |
| 93 | Motomura K, Sumita K, Chalise L, Nishikawa T, Tanahashi K, Ohka F, et al. Characterization of Intraoperative Motor Evoked Potential Monitoring for Surgery of the Pediatric Population with Brain Tumors. World neurosurgery. 2018;119:e1052-e9. doi: 10.1016/j.wneu.2018.08.039. |
| 94 | Motomura K, Sumita K, Chalise L, Nishikawa T, Tanahashi K, Ohka F, et al. Characterization of Intraoperative Motor Evoked Potential Monitoring for Surgery of the Pediatric Population with Brain Tumors. World neurosurgery. 2018;119:e1052-e9. doi: 10.1016/j.wneu.2018.08.039. |
| 95 | Shokhirev MN, Torosin NS, Kramer DJ, Johnson AA, Cuellar TL. CheekAge: a next-generation buccal epigenetic aging clock associated with lifestyle and health. GeroScience. 2024;46(3):3429-43. doi: 10.1007/s11357-024-01094-3. |
| 96 | Yifru S, Muluye D. Childhood cancer in Gondar University Hospital, Northwest Ethiopia. BMC research notes. 2015;8:474. doi: 10.1186/s13104-015-1440-1. |
| 97 | Holmes GE, Holmes FF, Baker AB, Hassanein RS. Childhood cancer survivors: Attained adult heights. Compared with sibling controls. Clinical pediatrics. 1990;29(5):268-72. |
| 98 | Magerman C, Boros E, Preziosi M, Lhoir S, Gilis N, De Witte O, et al. Childhood craniopharyngioma: a retrospective study of children followed in Hôpital Universitaire de Bruxelles. Frontiers in endocrinology. 2024;15. doi: 10.3389/fendo.2024.1297132. |
| 99 | Elowe-Gruau E, Beltrand J, Brauner R, Pinto G, Samara-Boustani D, Thalassinos C, et al. Childhood craniopharyngioma: Hypothalamus-sparing surgery decreases the risk of obesity. Journal of Clinical Endocrinology and Metabolism. 2013;98(6):2376-82. doi: 10.1210/jc.2012-3928. |
| 100 | Wermelt J, Ständer S, Pereira MP, Mannil M. Chronic brachioradial pruritus in cervical spine meningioma. Der Hautarzt; Zeitschrift fur Dermatologie, Venerologie, und verwandte Gebiete. 2022;73(6):485-7. doi: 10.1007/s00105-021-04897-z. |
| 101 | Gonzalez-Aponte MF, Damato AR, Trebucq LL, Simon T, Cárdenas-García SP, Cho K, et al. Circadian regulation of MGMT expression and promoter methylation underlies daily rhythms in TMZ sensitivity in glioblastoma. 2023. |
| 102 | Gonzalez-Aponte MF, Damato AR, Trebucq LL, Simon T, Cárdenas-García SP, Cho K, et al. Circadian regulation of MGMT expression and promoter methylation underlies daily rhythms in TMZ sensitivity in glioblastoma. Journal of neuro-oncology. 2024;166(3):419-30. doi: 10.1007/s11060-023-04535-9. |
| 103 | Wang Q, Zheng D, Li Y, Zhang Y, Sui R, Chen Y, et al. Circular RNA circ_0001588 sponges miR-211-5p to facilitate the progression of glioblastoma via up-regulating YY1 expression. Journal of Gene Medicine. 2021;23(10). doi: 10.1002/jgm.3371. |
| 104 | Liu F, Duan C, Han Y. Circular RNA hsa_circ_0000285 regulates the microRNA-599/G-protein subunit gamma 12 (miR-599/GNG12) axis to promote glioma progression. Journal of clinical laboratory analysis. 2022;36(3). doi: 10.1002/jcla.24207. |
| 105 | Carlotti Jr CG, Neder L, Colli BO, Dos Santos MB, Garcia AS, Elias Jr J, et al. Clear cell meningioma of the fourth ventricle. American Journal of Surgical Pathology. 2003;27(1):131-5. doi: 10.1097/00000478-200301000-00015. |
| 106 | Yuyama R, Mishima K, Fujimaki T, Suzuki I, Sasaki T, Ueno H, et al. Clinical experience of autologous blood transfusion and fibrin glue in neurosurgery. Neurological Surgery. 1998;26(8):685-90. |
| 107 | Van Iersel L, Xu J, Potter BS, Conklin HM, Zhang H, Chemaitilly W, et al. Clinical Importance of Free Thyroxine Concentration Decline after Radiotherapy for Pediatric and Adolescent Brain Tumors. Journal of Clinical Endocrinology and Metabolism. 2019;104(11):4998-5007. |
| 108 | Chotai S, Yan Y, Stewart T, Morone PJ. Clinical tool for prognostication of discharge outcomes following craniotomy for meningioma. Clinical neurology and neurosurgery. 2023;231. doi: 10.1016/j.clineuro.2023.107838. |
| 109 | Xie YG, Han FY, Peyrard M, Ruttledge MH, Fransson I, DeJong P, et al. Cloning of a novel, anonymous gene from a megabase-range YAC and cosmid contig in the neurofibromatosis type 2/meningioma region on human chromosome 22q12. Human molecular genetics. 1993;2(9):1361-8. |
| 110 | Arshad I, Jerez JV, Zahra T. Co-occurrence of Carotid Body Tumor, Meningioma and Schwannoma in a Symptomatic Patient, Incidental Findings Vs Genetic Syndrome; a case report. Journal of the Endocrine Society. 2022;6:A567-A8. doi: 10.1210/jendso/bvac150.1178. |
| 111 | Garg A, Patel P, Lignelli A, Baron E, Kazim M. Coincidental Optic Nerve Meningioma and Thyroid Eye Disease. Ophthalmic plastic and reconstructive surgery. 2015;31(4):e94-e5. doi: 10.1097/IOP.0000000000000113. |
| 112 | Liu TF, Cohen KA, Willingham MC, Tatter SB, Puri RK, Frankel AE. Combination fusion protein therapy of refractory brain tumors: Demonstration of efficacy in cell culture. Journal of neuro-oncology. 2003;65(1):77-85. doi: 10.1023/A:1026286214901. PubMed Central PMCID: Ligand(United States) |
| 113 | Syed S, Karambizi DI, Baker A, Groh DM, Toms SA. A Comparative Report on Intracranial Tumor-to-Tumor Metastasis and Collision Tumors. World neurosurgery. 2018;116:454-63.e2. doi: 10.1016/j.wneu.2018.04.109. |
| 114 | Iyengar V, Agrawal S, Chiasakul T, Tehranchi K, McNichol M, Carney B, et al. Comparison of Direct Oral Anticoagulants versus Low Molecular Weight Heparin in Primary and Metastatic Brain Cancers: A Meta-Analysis and Systematic Review. Research and Practice in Thrombosis and Haemostasis. 2023;7. doi: 10.1016/j.rpth.2023.100598. |
| 115 | Iyengar V, Agrawal S, Chiasakul T, Tehranchi K, McNichol M, Carney BJ, et al. Comparison of direct oral anticoagulants versus low-molecular-weight heparin in primary and metastatic brain cancers: a meta-analysis and systematic review. Journal of Thrombosis and Haemostasis. 2024;22(2):423-9. |
| 116 | Raygor KP, Than KD, Chou D, Mummaneni PV. Comparison of minimally invasive transspinous and open approaches for thoracolumbar intradural-extramedullary spinal tumors. Neurosurgical focus. 2015;39(2):E12. doi: 10.3171/2015.5.FOCUS15187. |
| 117 | Soldos P, Besenyi Z, Hideghéty K, Pávics L, Hegedűs Á, Rácz L, et al. Comparison of Shear Wave Elastography and Dynamometer Test in Muscle Tissue Characterization for Potential Medical and Sport Application. Pathology and Oncology Research. 2021;27. doi: 10.3389/pore.2021.1609798. |
| 118 | Hatzoglou V, Ulaner GA, Zhang Z, Beal K, Holodny AI, Young RJ. Comparison of the effectiveness of MRI perfusion and fluorine-18 FDG PET-CT for differentiating radiation injury from viable brain tumor: A preliminary retrospective analysis with pathologic correlation in all patients. Clinical imaging. 2013;37(3):451-7. |
| 119 | Quentin C, Charbonneau S, Moumdjian R, Lallo A, Bouthilier A, Fournier-Gosselin MP, et al. A comparison of two doses of mannitol on brain relaxation during supratentorial brain tumor craniotomy: A randomized trial. Anesthesia and analgesia. 2013;116(4):862-8. doi: 10.1213/ANE.0b013e318282dc70. |
| 120 | Sakai H, Yoshioka K, Yamagami K, Yamakita T, Hosoi M, Ishii T, et al. Complete adrenocorticotropin deficiency after radiation therapy for brain tumor with a normal growth hormone reserve. Internal Medicine. 2002;41(6):453-7. doi: 10.2169/internalmedicine.41.453. |
| 121 | Brosnan C, Razis P. Complications of treatment: Pulmonary embolism following craniotomy for meningioma. Journal of neurosurgical anesthesiology. 1999;11(2):119-23. doi: 10.1097/00008506-199904000-00008. |
| 122 | Fukui T, Suehiro S, Shibata T, Hirai H, Aoyama T. Concomitant pulmonary embolectomy and excision of a left atrial myxoma. Journal of cardiac surgery. 2002;17(5):408-9. Epub 2003/03/13. doi: 10.1111/j.1540-8191.2001.tb01169.x. PubMed PMID: 12630540. |
| 123 | Whitson WJ, Valdes PA, Harris BT, Paulsen KD, Roberts DW. Confocal microscopy for the histological fluorescence pattern of a recurrent atypical meningioma: Case report. Neurosurgery. 2011;68(6):E1768-E72. doi: 10.1227/NEU.0b013e318217163c. PubMed Central PMCID: Dusa(United States). |
| 124 | Kuo YC, Chao IW. Conjugation of melanotransferrin antibody on solid lipid nanoparticles for mediating brain cancer malignancy. Biotechnology progress. 2016;32(2):480-90. doi: 10.1002/btpr.2214. |
| 125 | Lim U, Subar AF, Mouw T, Hartge P, Morton LM, Stolzenberg-Solomon R, et al. Consumption of aspartame-containing beverages and incidence of hematopoietic and brain malignancies. Cancer Epidemiology Biomarkers and Prevention. 2006;15(9):1654-9. doi: 10.1158/1055-9965.EPI-06-0203. |
| 126 | Hawighorst H, Debus J, Schreiber W, Knopp MV, Engenhart-Cabillic R, Essig M, et al. Contrast-enhanced magnetization transfer imaging: Improvement of brain tumor conspicuity and delineation for radiosurgical target -volume definition. Radiotherapy and Oncology. 1997;43(3):261-7. |
| 127 | Zincircioglu SB, Kaplan MA, Isikdogan A, Cil T, Karadayi B, Dirier A, et al. Contribution of low-molecular weight heparin addition to concomitant chemoradiotherapy in the treatment of glioblastoma multiforme. Journal of BUON. 2012;17(1):124-7. |
| 128 | Oishi T, Sameshima T, Totsuka T, Yamasaki T, Koizumi S, Namba H. Cosmetic and Neuroprotective Placement of Custom-Made Ultra-High-Molecular-Weight Polyethylene Cranial Plate (SKULPIO) in Single-Step Surgery: Technical Note and Case Report. World neurosurgery. 2019;130:187-91. |
| 129 | Emfietzoglou R, Spyrou N, Mantzoros CS, Dalamaga M. Could the endocrine disruptor bisphenol-A be implicated in the pathogenesis of oral and oropharyngeal cancer? Metabolic considerations and future directions. Metabolism: clinical and experimental. 2019;91:61-9. |
| 130 | Lönnemark O, Ryttlefors M, Sundblom J. Cranioplasty in Brain Tumor Surgery: A Single-Center Retrospective Study Investigating Cranioplasty Failure and Tumor Recurrence. World neurosurgery. 2023;170:e313-e23. doi: 10.1016/j.wneu.2022.11.010. |
| 131 | Menezes AH. Craniovertebral junction neoplasms in the pediatric population. Child's Nervous System. 2008;24(10):1173-86. doi: 10.1007/s00381-008-0598-4. |
| 132 | Gezer E, Cantürk Z, Selek A, Çetinarslan B, Tarkun İ, Sözen M, et al. Cushing's disease due to a pituitary adenoma as a component of collision tumor: A case report and review of the literature. Journal of medical case reports. 2020;14(1). doi: 10.1186/s13256-020-02382-0. |
| 133 | Li Q, Wang J, Zhou Y. Data augmentation method based on the Gaussian kernel density for glioma diagnosis with Raman spectroscopy. Analytical methods : advancing methods and applications. 2023;15(15):1861-9. doi: 10.1039/d3ay00188a. |
| 134 | De A, Tiwari M, Grisan E, Chowdhury AS. A Deep Graph Cut Model For 3D Brain Tumor Segmentation. Annual International Conference of the IEEE Engineering in Medicine and Biology Society IEEE Engineering in Medicine and Biology Society Annual International Conference. 2022;2022:2105-9. |
| 135 | Hirao M, Oka N, Hirashima Y, Horie Y, Takaku A. Deep sylvian meningioma: case report and review of the literature. No shinkei geka Neurological surgery. 1986;14(12):1471-8. |
| 136 | Chaichana KL, Pendleton C, Jackson C, Martinez-Gutierrez JC, Diaz-Stransky A, Aguayo J, et al. Deep venous thrombosis and pulmonary embolisms in adult patients undergoing craniotomy for brain tumors. Neurological research. 2013;35(2):206-11. doi: 10.1179/1743132812Y.0000000126. |
| 137 | Levy ML, Granville RC, Hart D, Meltzer H. Deep venous thrombosis in children and adolescents. Journal of neurosurgery. 2004;101(1 Suppl):32-7. |
| 138 | Grzmil M, Hemmings BA. Deregulated signalling networks in human brain tumours. Biochimica et Biophysica Acta - Proteins and Proteomics. 2010;1804(3):476-83. doi: 10.1016/j.bbapap.2009.10.018. |
| 139 | Orthmann A, Zeisig R, Fichtmer I. Design and development of nanocarrier for efficient drug delivery into the brain. Journal of Cancer Research and Clinical Oncology. 2012;138:15. doi: 10.1007/s00432-011-1144-4. |
| 140 | Orthmann A, Zeisig R, Fichtner I. Design and development of nanocarrier for efficient drug delivery into the brain. European Journal of Cancer, Supplement. 2010;8(7):136. doi: 10.1016/S1359-6349(10)72136-4. |
| 141 | Dahlberg S, Nilsson CU, Kander T, Schött U. Detection of subclinical vitamin K deficiency in neurosurgery with PIVKA-II. Scandinavian journal of clinical and laboratory investigation. 2017;77(4):267-74. doi: 10.1080/00365513.2017.1303190. |
| 142 | Dahlberg S, Nilsson CU, Kander T, Schött U. Detection of subclinical vitamin K deficiency in neurosurgery with PIVKA-II. Scandinavian journal of clinical and laboratory investigation. 2017;77(4):267-74. doi: 10.1080/00365513.2017.1303190. |
| 143 | Forsyth PAJ, Evernden B, Law V, Kenchappa R, Puskas J, Ryzhova E, et al. Detection, molecular profiling and culture of CSF-CTCs in leptomeningeal disease (LMDz) in melanoma. Journal of Clinical Oncology. 2018;36(15). doi: 10.1200/jco.2018.36.15_suppl.e21543. |
| 144 | Fleming CAK, Viani K, Murphy A, Mosby T, Arora B, Schoeman J, et al. The development, testing, and preliminary feasibility of an adaptable pediatric oncology nutrition algorithm for low-middle income countries. Indian journal of cancer. 2015;52(2):225-8. doi: 10.4103/0019-509X.175834. |
| 145 | Seliger C, Meier CR, Becker C, Jick SS, Proescholdt M, Bogdahn U, et al. Diabetes, use of metformin, and the risk of meningioma. PloS one. 2017;12(7). doi: 10.1371/journal.pone.0181089. |
| 146 | Cornelli L, Van Paemel R, Ferro dos Santos MR, Roelandt S, Willems L, Vandersteene J, et al. Diagnosis of pediatric central nervous system tumors using methylation profiling of cfDNA from cerebrospinal fluid. Clinical epigenetics. 2024;16(1). doi: 10.1186/s13148-024-01696-w. |
| 147 | Guo WD, Linet MS, Chow WH, Li JY, Blot WJ. Diet and serum markers in relation to primary brain tumor risk in China. Nutrition and cancer. 1994;22(2):143-50. |
| 148 | Philipova T, Baryawno N, Hartmann W, Pietsch T, Druid H, Johnsen JI, et al. Differential forms of p53 in medulloblastoma primary tumors, cell lines and xenografts. International journal of oncology. 2011;38(3):843-9. doi: 10.3892/ijo.2010.884. |
| 149 | Barnett PA, Roman-Goldstein S, Ramsey F, McCormick CI, Sexton G, Szumowski J, et al. Differential permeability and quantitative MR imaging of a human lung carcinoma brain xenograft in the nude rat. American Journal of Pathology. 1995;146(2):436-49. |
| 150 | Neuwelt EA, Barnett PA, McCormick CI, Remsen LG, Kroll RA, Sexton G. Differential permeability of a human brain tumor xenograft in the nude rat: Impact of tumor size and method of administration on optimizing delivery of biologically diverse agents. Clinical Cancer Research. 1998;4(6):1549-55. |
| 151 | Bricaire C, Plu-Bureau G. Difficult contraceptions. Revue du Praticien. 2008;58(1):55-8+61-4. |
| 152 | Senthil Kumar S, Sengupta S, Zhu X, Mishra DK, Phoenix T, Dyer L, et al. Diffuse Intrinsic Pontine Glioma Cells Are Vulnerable to Mitotic Abnormalities Associated with BMI-1 Modulation. Molecular cancer research : MCR. 2020;18(11):1711-23. |
| 153 | Lee A, Oley F, Lo M, Fong R, McGann M, Saunders I, et al. Direct oral anticoagulants (DOACs) vs. low molecular weight heparins (LMWH) for venous thromboembolism (VTE) in patients with primary brain tumors or secondary brain metastases. Blood. 2020;136(SUPPL 1):6. d |
| 154 | Dubinski D, Won SY, Miesbach W, Keil F, Behmanesh B, Baumgarten P, et al. Direct oral anticoagulants for therapeutic anticoagulation in postoperative pulmonary embolism after meningioma resection. Journal of Clinical Neuroscience. 2020;81:265-9. doi: 10.1016/j.jocn.2020.09.059. |
| 155 | Lee A, Oley F, Lo M, Fong R, McGann M, Saunders I, et al. Direct oral anticoagulants or low-molecular-weight heparins for venous thromboembolism in patients with brain tumors. Thrombosis research. 2021;208:148-55. doi: 10.1016/j.thromres.2021.10.023. |
| 156 | Yu W, Kherani J, Tham D, Zhao L, Li P, Eshaghpour A, et al. Direct Oral Anticoagulants vs Low Molecular Weight Heparin in the Management of Patients with Primary or Metastatic Brain Tumours: A Systematic Review and Meta-Analysis. Research and Practice in Thrombosis and Haemostasis. 2023;7. doi: 10.1016/j.rpth.2023.100594. |
| 157 | Nikishina VB, Lazarenko VA, Petrash EA, Ahmetzyanova AI. Disturbance of body image in patients with meningiomas of parieto-occipital localization. Zhurnal Nevrologii i Psihiatrii imeni SS Korsakova. 2016;116(12):20-4. doi: 10.17116/jnevro201611612120-24. |
| 158 | Quan J, Ma C. DNMT1-mediated regulating on FBXO32 promotes the progression of glioma cells through the regulation of SKP1 activity. Environmental toxicology. 2024;39(2):783-93. doi: 10.1002/tox.23976. |
| 159 | Olivi A, Grossman SA, Tatter S, Barker F, Judy K, Olsen J, et al. Dose escalation of carmustine in surgically implanted polymers in patients with recurrent malignant glioma: A New Approaches to Brain Tumor Therapy CNS Consortium Trial. Journal of Clinical Oncology. 2003;21(9):1845-9. |
| 160 | Gadoue SM, Toomeh D, Schultze BE, Schulte RW. A dose-volume constraint (DVC) projection-based algorithm for IMPT inverse planning optimization. Medical physics. 2022;49(4):2699-708. Epub 2022/02/02. doi: 10.1002/mp.15504. PubMed PMID: 35103982. |
| 161 | Schmiegelow M, Lassen S, Weber L, Poulsen HS, Hertz H, Müller J. Dosimetry and growth hormone deficiency following cranial irradiation of childhood brain tumors. Medical and pediatric oncology. 1999;33(6):564-71. doi: 10.1002/(SICI)1096-911X(199912)33:6<564::AID-MPO8>3.0.CO;2-N. |
| 162 | Tatsuno S, Hata Y, Tada S. Double-dose Gd-DTPA: detectability of intraparenchymal brain metastasis. Nihon Igaku Hoshasen Gakkai zasshi Nippon acta radiologica. 1996;56(12):855-9. |
| 163 | Shen C, Wang X, Zheng Z, Gao C, Chen X, Zhao S, et al. Doxorubicin and indocyanine green loaded superparamagnetic iron oxide nanoparticles with PEGylated phospholipid coating for magnetic resonance with fluorescence imaging and chemotherapy of glioma. International journal of nanomedicine. 2019;14:101-17. |
| 164 | Nakagomi T, Takakura K. [Dynamic computed tomography of brain tumor]. No to shinkei = Brain and nerve. 1984;36(10):1031-40. Epub 1984/10/01. PubMed PMID: 6518125. |
| 165 | Nakagomi T, Segawa H, Tanaka H. Dynamic computed tomography of the brain. Neurosurgical review. 1985;8(1):15-25. |
| 166 | Caldeira LL, Yun SD, da Silva NA, Filss C, Shah NJ. Dynamic susceptibility contrast parametric imaging using accelerated dual-contrast echo planar imaging with keyhole. Journal of Magnetic Resonance Imaging. 2019;50(2):628-40. doi: 10.1002/jmri.26639. PubMed Central PMCID: Siemens(Germany). |
| 167 | Ramanauskienė E, Labanauskas L, Verkauskienė R, Sileikienė R. Early development of endocrine and metabolic consequences after treatment of central nervous system tumors in children. Medicina (Kaunas, Lithuania). 2014;50(5):275-80. doi: 10.1016/j.medici.2014.10.006. |
| 168 | Sescu D, Chansiriwongs A, Minta KJ, Vasudevan J, Kaliaperumal C. Early Preventive Strategies and CNS Meningioma – Is This Feasible? A Comprehensive Review of the Literature. World neurosurgery. 2023;180:123-33. doi: 10.1016/j.wneu.2023.09.075. |
| 169 | Siviero-Miachon AA, Monteiro CM, Pires LV, Rozalem AC, Silva NS, Petrilli AS, et al. Early traits of metabolic syndrome in pediatric post-cancer survivors: outcomes in adolescents and young adults treated for childhood medulloblastoma. Arquivos brasileiros de endocrinologia e metabologia. 2011;55(8):653-60. |
| 170 | Ozen Y, Ugur M, Ozbek IC, Yalcinkaya E. Early-Term Outcomes of Pulmonary Embolism Response Team. Phlebology. 2022;37(2):139-40. doi: 10.1177/02683555221110363. |
| 171 | Hansen JA, Stancel HH, Klesges LM, Tyc VL, Hinds PS, Wu S, et al. Eating behavior and BMI in adolescent survivors of brain tumor and acute lymphoblastic leukemia. Journal of pediatric oncology nursing : official journal of the Association of Pediatric Oncology Nurses. 2014;31(1):41-50. d |
| 172 | Bishop CJ, Ketola TM, Tzeng SY, Sunshine JC, Urtti A, Lemmetyinen H, et al. The effect and role of carbon atoms in Poly(β-amino ester)s for DNA binding and gene delivery. Journal of the American Chemical Society. 2013;135(18):6951-7. doi: 10.1021/ja4002376. |
| 173 | Izumoto S, Arita N, Ushio Y, Hayakawa T, Ohnishi T, Taki T, et al. Effect of MX-2, a morpholino anthracycline derivative, against human and rat glioma cells and experimental leptomeningeal tumors in rats. Gan to kagaku ryoho Cancer & chemotherapy. 1988;15(5):1765-9. |
| 174 | Cohen A, Noxon V, Dhamane A, Bruette R, Shah S, Hines DM, et al. Effectiveness and safety of anticoagulants among venous thromboembolism cancer patients with and without brain cancer. Thrombosis research. 2023;226:117-26. doi: 10.1016/j.thromres.2023.04.009. |
| 175 | Bruening R, Berchtenbreiter C, Holzknecht N, Essig M, Wu RH, Simmons A, et al. Effects of three different doses of a bolus injection of gadodiamide: assessment of regional cerebral blood volume maps in a blinded reader study. AJNR American journal of neuroradiology. 2000;21(9):1603-10. |
| 176 | Palmer JD, Francis JL, Pickard JD, Iannotti F. The efficacy and safety of aprotinin for hemostasis during intracranial surgery. Journal of neurosurgery. 2003;98(6):1208-16. doi: 10.3171/jns.2003.98.6.1208. PubMed Central PMCID: Bayer(United Kingdom). |
| 177 | Miller AB, Green LM. Electric and magnetic fields at power frequencies. Chronic diseases in Canada. 2010;29 Suppl 1:69-83. |
| 178 | Schneider M, Borger V, Grigutsch D, Güresir Á, Potthoff AL, Velten M, et al. Elevated body mass index facilitates early postoperative complications after surgery for intracranial meningioma. Neurosurgical review. 2021;44(2):1023-9. doi: 10.1007/s10143-020-01281-8. |
| 179 | van Schaik J, van Roessel IMAA, Bos ID, Claashen-van der Grinten HL, Clement SC, van Iersel L, et al. Elevated IGF-1 concentrations in children with low grade glioma: A descriptive analysis in a retrospective national cohort. Journal of neuroendocrinology. 2023;35(8). doi: 10.1111/jne.13317. |
| 180 | Kapoor-Narula U, Lenka N. Elucidating the Anti-Tumorigenic Efficacy of Oltipraz, a Dithiolethione, in Glioblastoma. Cells. 2022;11(19). doi: 10.3390/cells11193057. |
| 181 | Morrison LC, Tatari N, Werbowetski-Ogilvie TE. Embryonic Stem Cell Models of Human Brain Tumors. Methods in molecular biology (Clifton, NJ). 2019;1869:127-42. Epub 2018/10/17. doi: 10.1007/978-1-4939-8805-1_12. PubMed PMID: 30324520. |
| 182 | Shalitin S, Gal M, Goshen Y, Cohen I, Yaniv I, Phillip M. Endocrine outcome in long-term survivors of childhood brain tumors. Hormone research in paediatrics. 2011;76(2):113-22. doi: 10.1159/000327584. |
| 183 | Schildkraut JM, Calvocoressi L, Wang F, Wrensch M, Bondy ML, Wiemels JL, et al. Endogenous and exogenous hormone exposure and the risk of meningioma in men: Clinical article. Journal of neurosurgery. 2014;120(4):820-6. doi: 10.3171/2013.12.JNS131170. |
| 184 | Obacz J, Avril T, Le Reste PJ, Urra H, Quillien V, Hetz C, et al. Endoplasmic reticulum proteostasis in glioblastoma - From molecular mechanisms to therapeutic perspectives. Science signaling. 2017;10(470). doi: 10.1126/scisignal.aal2323. |
| 185 | Fan CH, Wang TW, Hsieh YK, Wang CF, Gao Z, Kim A, et al. Enhancing Boron Uptake in Brain Glioma by a Boron-Polymer/Microbubble Complex with Focused Ultrasound. ACS applied materials & interfaces. 2019;11(12):11144-56. doi: 10.1021/acsami.8b22468. |
| 186 | Walsh KM. Epidemiology of meningiomas. Handbook of clinical neurology. 2020;169:3-15. doi: 10.1016/B978-0-12-804280-9.00001-9. |
| 187 | Loiseau H, Huchet A, Rué M, Cowppli-Bony A, Baldi I. Epidemiology of primary brain tumor. Revue neurologique. 2009;165(8-9):650-70. doi: 10.1016/j.neurol.2009.04.002. |
| 188 | Falanga A, Russo L. Epidemiology, risk and outcomes of venous thromboembolism in cancer. Hamostaseologie. 2012;32(2):115-25. doi: 10.5482/ha-1170. |
| 189 | Kamson D, Yuen C, Saavedra LC, Vogel TJ, Merrell R, Walker M. Estrogen hormone replacement therapy in incidental meningioma-a growth rate analysis. Neuro-oncology. 2019;21:vi139. doi: 10.1093/neuonc/noz175.585. |
| 190 | Ida S. Evaluation and treatment of gastroesophageal reflux in infants and children. Nippon rinsho Japanese journal of clinical medicine. 2004;62(8):1553-8. |
| 191 | Lv PC, Roy J, Putt KS, Low PS. Evaluation of a Carbonic Anhydrase IX-Targeted Near-Infrared Dye for Fluorescence-Guided Surgery of Hypoxic Tumors. Molecular pharmaceutics. 2016;13(5):1618-25. doi: 10.1021/acs.molpharmaceut.6b00065. |
| 192 | Rakhshani N, Jeffery AS, Schulte F, Barrera M, Atenafu EG, Hamilton JK. Evaluation of a comprehensive care clinic model for children with brain tumor and risk for hypothalamic obesity. Obesity. 2010;18(9):1768-74. doi: 10.1038/oby.2009.491. |
| 193 | Sapkota MR, Yang Z, Zhu D, Zhang Y, Yuan T, Gao J, et al. Evaluation of Epidemiologic Factors, Radiographic Features, and Pathologic Findings for Predicting Peritumoral Brain Edema in Meningiomas. Journal of Magnetic Resonance Imaging. 2020;52(1):174-82. doi: 10.1002/jmri.27046. |
| 194 | Antle SD, Ho AK, Kalyan-Raman UP. Evidence for a calmodulin inhibitory substance(s) isolated from human meningiomas. Journal of neuro-oncology. 1993;16(3):201-9. Epub 1993/06/01. doi: 10.1007/bf01057034. PubMed PMID: 8301344. |
| 195 | Pareja-Peña F, Burgos-Molina AM, Sendra-Portero F, Ruiz-Gómez MJ. Evidences of the (400 MHz - 3 GHz) radiofrequency electromagnetic field influence on brain tumor induction. International journal of environmental health research. 2022;32(1):121-30. |
| 196 | Conger A, Zhao F, Wang X, Eisenberg A, Griffiths C, Esposito F, et al. Evolution of the graded repair of CSF leaks and skull base defects in endonasal endoscopic tumor surgery: Trends in repair failure and meningitis rates in 509 patients. Journal of neurosurgery. 2019;130(3):861-75. |
| 197 | Lakshmi A, Alagarsamy M, Anbarasa Pandian A, Paramathi Mani D. Evolutionary gravitational neocognitron neural network optimized with marine predators optimization algorithm for MRI brain tumor classification. Electromagnetic biology and medicine. 2024;43(1-2):1-18. |
| 198 | Patel AV, Patel KS, Teras LR. Excess body fatness and cancer risk: a summary of the epidemiologic evidence. Surgery for Obesity and Related Diseases. 2023;19(7):742-5. doi: 10.1016/j.soard.2023.01.025. |
| 199 | Scherübl H. [Excess Body Weight and Cancer Risk]. Deutsche medizinische Wochenschrift (1946). 2020;145(14):1006-14. Epub 2020/07/16. doi: 10.1055/a-1126-3903. PubMed PMID: 32668472. |
| 200 | Excess Weight Tied to Eight More Cancers. Cancer discovery. 2016;6(10):1072-3. doi: 10.1158/2159-8290.CD-NB2016-111. |
| 201 | Claus EB, Calvocoressi L, Bondy ML, Wrensch M, Wiemels JL, Schildkraut JM. Exogenous hormone use, reproductive factors, and risk of intracranial meningioma in females. Journal of neurosurgery. 2013;118(3):649-56. doi: 10.3171/2012.9.JNS12811. |
| 202 | Van Schaik J, Begijn DGA, Van Iersel L, Vergeer Y, Hoving EW, Peeters B, et al. Experiences with Glucagon-Like Peptide-1 Receptor Agonist in Children with Acquired Hypothalamic Obesity. Obesity facts. 2020;13(4):361-70. doi: 10.1159/000509302. |
| 203 | Verhoeff JJ, Stalpers LJ, Coumou AW, Koedooder K, Lavini C, Van Noorden CJ, et al. Experimental iodine-125 seed irradiation of intracerebral brain tumors in nude mice. Radiation oncology (London, England). 2007;2:38. doi: 10.1186/1748-717X-2-38. |
| 204 | Di Iorgi N, Morana G, Cappa M, D’Incerti L, Garrè ML, Grossi A, et al. Expert Opinion on the Management of Growth Hormone Deficiency in Brain Tumor Survivors: Results From an Italian Survey. Frontiers in endocrinology. 2022;13. doi: 10.3389/fendo.2022.920482. |
| 205 | Libermann TA, Razon N, Bartal AD. Expression of epidermal growth factor receptors in human brain tumors. Cancer research. 1984;44(2):753-60. |
| 206 | Chernov AN, Kim AV, Skliar SS, Fedorov EV, Tsapieva AN, Filatenkova TA, et al. Expression of molecular markers and synergistic anticancer effects of chemotherapy with antimicrobial peptides on glioblastoma cells. Cancer chemotherapy and pharmacology. 2024;93(5):455-69. |
| 207 | Amiri A, Mousakhani-Ganjeh A, Amiri Z, Guo YG, Pratap Singh A, Esmaeilzadeh Kenari R. Fabrication of cumin loaded-chitosan particles: Characterized by molecular, morphological, thermal, antioxidant and anticancer properties as well as its utilization in food system. Food chemistry. 2020;310. |
| 208 | Cerhan JH, Butts AM, Syrjanen JA, Aakre JA, Brown PD, Petersen RC, et al. Factors Associated With Meningioma Detected in a Population-Based Sample. Mayo Clinic proceedings. 2019;94(2):254-61. doi: 10.1016/j.mayocp.2018.07.026. |
| 209 | Shimazaki S, Kazukawa I, Mori K, Kihara M, Minagawa M. Factors predicting endocrine late effects in childhood cancer survivors from a Japanese hospital. Endocrine journal. 2020;67(2):131-40. doi: 10.1507/endocrj.EJ19-0228. |
| 210 | Isobe N, Ikawa F, Tominaga A, Kuroki K, Sadatomo T, Mizoue T, et al. Factors Related to Frailty Associated with Clinical Deterioration After Meningioma Surgery in the Elderly. World neurosurgery. 2018;119:e167-e73. doi: 10.1016/j.wneu.2018.07.080. |
| 211 | Isobe N, Ikawa F, Tominaga A, Kuroki K, Sadatomo T, Mizoue T, et al. Factors Related to Frailty Associated with Clinical Deterioration After Meningioma Surgery in the Elderly. World neurosurgery. 2018;119:e167-e73. doi: 10.1016/j.wneu.2018.07.080. |
| 212 | Lee V, Smith TW, Arikan M, Zhang L, Çataltepe O, Çataltepe S. Fatty Acid-binding Protein 4 Expression in Tumor Cells as a Potential Marker for Anaplastic Meningiomas. Applied immunohistochemistry & molecular morphology : AIMM. 2021;29(2):e10-e6. |
| 213 | Mansell M, Hill R, Johnson D, Hamby T, Hines A. Favorable Parental Perception of Proactive Nutrition Intervention in High-risk Pediatric Brain Tumor Population and Impact on Nutrition Outcomes. Journal of pediatric hematology/oncology. 2023;45(1):E103-E8. doi: 10.1097/MPH.0000000000002541. |
| 214 | Ishihara T, Nogami K, Takeshita Y, Ochi S, Shima M. Fibrinolytic abnormality associated with progression of pediatric solid tumor. Pediatrics International. 2018;60(6):540-6. doi: 10.1111/ped.13546. |
| 215 | Gurney JG, Ness KK, Stovall M, Wolden S, Punyko JA, Neglia JP, et al. Final Height and Body Mass Index among Adult Survivors of Childhood Brain Cancer: Childhood Cancer Survivor Study. Journal of Clinical Endocrinology and Metabolism. 2003;88(10):4731-9. doi: 10.1210/jc.2003-030784. |
| 216 | Chae HW, Park YS, Kim DS, Kwon AR, Kim HS, Kim DH. Final height and insulin-like growth factor-1 in children with medulloblastoma treated with growth hormone. Child's Nervous System. 2013;29(10):1859-63. doi: 10.1007/s00381-013-2124-6. |
| 217 | Furuhata H, Araki K, Ogawa T. Financial Estimation of the Uncertainty in Medicine Using Present Value of Medical Fees and a Mortality Risk Prediction Model: a Retrospective Cohort Study. Journal of medical systems. 2021;45(11). doi: 10.1007/s10916-021-01775-y. |
| 218 | Voelter-Mahlknecht S, Ho AD, Mahlknecht U. FISH-mapping and genomic organization of the NAD-dependent histone deacetylase gene, Sirtuin 2 (Sirt2). International journal of oncology. 2005;27(5):1187-96. |
| 219 | Preusser M, Plumer S, Dirnberger E, Hainfellner JA, Mannhalter C. Fixation of brain tumor biopsy specimens with RCL2 results in well-preserved histomorphology, immunohistochemistry and nucleic acids. Brain Pathology. 2010;20(6):1010-20. doi: 10.1111/j.1750-3639.2010.00400.x. |
| 220 | Tsao GJ, Tsang MW, Mobley BC, Cheng WW. Foramen magnum meningioma: Dysphagia of atypical etiology. Journal of general internal medicine. 2008;23(2):206-9. doi: 10.1007/s11606-007-0474-z. |
| 221 | Sastry RA, Pertsch NJ, Tang O, Shao B, Toms SA, Weil RJ. Frailty and outcomes after craniotomy for brain tumor. Journal of Clinical Neuroscience. 2020;81:95-100. doi: 10.1016/j.jocn.2020.09.002. |
| 222 | Harland TA, Wang M, Gunaydin D, Fringuello A, Freeman J, Hosokawa PW, et al. Frailty as a Predictor of Neurosurgical Outcomes in Brain Tumor Patients. World neurosurgery. 2020;133:e813-e8. doi: 10.1016/j.wneu.2019.10.010. |
| 223 | Weber RG, Hoischen A, Ehrler M, Zipper P, Kaulich K, Blaschke B, et al. Frequent loss of chromosome 9, homozygous CDKN2A/p14ARF/CDKN2B deletion and low TSC1 mRNA expression in pleomorphic xanthoastrocytomas. Oncogene. 2007;26(7):1088-97. doi: 10.1038/sj.onc.1209851. |
| 224 | Kilinc F, Setzer M, Marquardt G, Keil F, Dubinski D, Bruder M, et al. Functional outcome and morbidity after microsurgical resection of spinal meningiomas. Neurosurgical focus. 2021;50(5):E20. doi: 10.3171/2021.2.FOCUS201116. |
| 225 | Piaskowski S, Rieske P, Szybka M, Wozniak K, Bednarek A, Płuciennik E, et al. GADD45A and EPB41 as tumor suppressor genes in meningioma pathogenesis. Cancer genetics and cytogenetics. 2005;162(1):63-7. doi: 10.1016/j.cancergencyto.2005.02.009. |
| 226 | Parizel PM, Degryse HR, Gheuens J, Martin JJ, Van Vyve M, De La Porte C, et al. Gadolinium-DOTA enhanced MR imaging of intracranial lesions. Journal of computer assisted tomography. 1989;13(3):378-85. PubMed Central PMCID: Guerbet(France). |
| 227 | Bird CR, Drayer BP, Medina M, Rekate HL, Flom RA, Hodak JA. Gd-DTPA-enhanced MR imaging in pediatric patients after brain tumor resection. Radiology. 1988;169(1):123-6. doi: 10.1148/radiology.169.1.3420247. PubMed Central PMCID: Berlex(United States). |
| 228 | Calastri MCJ, Rodrigues N, Hatori G, Gregório ML, Brancati C, Zanovelo EM, et al. Genetic variants related to angiogenesis and apoptosis in patients with glioma. Arquivos de neuro-psiquiatria. 2018;76(6):393-8. Epub 2018/07/05. doi: 10.1590/0004-282x20180051. PubMed PMID: 29972422. |
| 229 | Kuijten RR, Bunin GR, Nass CC, Meadows AT. Gestational and familial risk factors for childhood astrocytoma: Results of a case-control study. Cancer research. 1990;50(9):2608-12. |
| 230 | Wolf S, Riess A, Landscheidt JF, Lumenta CB, Friederich P, Schürer L. Global end-diastolic volume acquired by transpulmonary thermodilution depends on age and gender in awake and spontaneously breathing patients. Critical care (London, England). 2009;13(6):R202. |
| 231 | Wang L, Xu R, Kaelber DC, Berger NA. Glucagon-Like Peptide 1 Receptor Agonists and 13 Obesity-Associated Cancers in Patients with Type 2 Diabetes. JAMA network open. 2024;7(7):e2421305. doi: 10.1001/jamanetworkopen.2024.21305. |
| 232 | Jasper BW, Conklin HM, Lawford J, Morris EB, Howard SC, Wu S, et al. Growth effects of methylphenidate among childhood cancer survivors: A 12-month case-matched open-label study. Pediatric Blood and Cancer. 2009;52(1):39-43. doi: 10.1002/pbc.21770. |
| 233 | Clarson CL, Del Maestro RF. Growth failure after treatment of pediatric brain tumors. Pediatrics. 1999;103(3):E37. |
| 234 | Sodero G, Agresti P, Triarico S, Romano A, Mastrangelo S, Attinà G, et al. Growth hormone replacement therapy in pediatric brain tumor survivors. Minerva pediatrics. 2022;74(3):340-8. doi: 10.23736/S2724-5276.22.06799-4. |
| 235 | Sano T, Yamasaki R, Saito H, Hirose T, Kudo E, Kameyama K, et al. Growth hormone-releasing hormone (GHRH)-secreting pancreatic tumor in a patient with multiple endocrine neoplasia type I. American Journal of Surgical Pathology. 1987;11(10):810-9. doi: 10.1097/00000478-198710000-00008. |
| 236 | Lin MF, Kawachi MH, Stallcup MR, Grunberg SM, Lin FF. Growth inhibition of androgen-insensitive human prostate carcinoma cells by a 19-norsteroid derivative agent, mifepristone. The Prostate. 1995;26(4):194-204. PubMed Central PMCID: Roussel Uclaf(France) |
| 237 | Luo Z, Jia Z, Yuan Z, Peng J. HDC-Net: Hierarchical Decoupled Convolution Network for Brain Tumor Segmentation. IEEE journal of biomedical and health informatics. 2021;25(3):737-45. doi: 10.1109/JBHI.2020.2998146. |
| 238 | Crom DB, Smith D, Xiong Z, Onar A, Hudson MM, Merchant TE, et al. Health status in long-term survivors of pediatric craniopharyngiomas. Journal of Neuroscience Nursing. 2010;42(6):323-8. doi: 10.1097/JNN.0b013e3181f8a59d. |
| 239 | Yust-Katz S, Amiel A, Shkara RA, Siegal T, Twig G. Height and the risk of meningioma. Neuro-oncology. 2019;21:vi74. doi: 10.1093/neuonc/noz175.302. |
| 240 | Ben-Zion Berliner M, Katz LH, Derazne E, Levine H, Keinan-Boker L, Benouaich-Amiel A, et al. Height as a risk factor in meningioma: A study of 2 million Israeli adolescents. BMC cancer. 2020;20(1). doi: 10.1186/s12885-020-07292-4. |
| 241 | Sterzing F, Schubert K, Sroka-Perez G, Kalz J, Debus J, Herfarth K. Helical tomotherapy: Experiences of the first 150 patients in Heidelberg. Strahlentherapie und Onkologie. 2008;184(1):8-14. doi: 10.1007/s00066-008-1778-6. |
| 242 | Spínola A, Ferreira S, Amado F, Lopes S, Roncon S. Hematopoietic progenitor cells collection in pediatric patients with brain tumor. Journal of clinical apheresis. 2016;31(1):22-8. doi: 10.1002/jca.21398. PubMed Central PMCID: Amgen(United States). |
| 243 | Schnoor R, Maas SLN, Broekman MLD. Heparin in malignant glioma: review of preclinical studies and clinical results. Journal of neuro-oncology. 2015;124(2):151-6. doi: 10.1007/s11060-015-1826-x. |
| 244 | Liu H, Liu N, Cheng Y, Jin W, Zhang P, Wang X, et al. Hexokinase 2 (HK2), the tumor promoter in glioma, is downregulated by miR-218/Bmi1 pathway. PloS one. 2017;12(12). doi: 10.1371/journal.pone.0189353. |
| 245 | Kwon OI, Lee MB, Jahng GH. High frequency conductivity decomposition by solving physically constraint underdetermined inverse problem in human brain. Scientific reports. 2023;13(1):3273. doi: 10.1038/s41598-023-30344-1. |
| 246 | Ronsley R, Rassekh SR, Fleming A, Empringham B, Jennings W, Portwine C, et al. High molecular weight adiponectin levels are inversely associated with adiposity in pediatric brain tumor survivors. Scientific reports. 2020;10(1):18606. doi: 10.1038/s41598-020-75638-w. |
| 247 | González Briceño LG, Kariyawasam D, Samara-Boustani D, Giani E, Beltrand J, Bolle S, et al. High Prevalence of Early Endocrine Disorders After Childhood Brain Tumors in a Large Cohort. Journal of Clinical Endocrinology and Metabolism. 2022;107(5):E2156-E66. doi: 10.1210/clinem/dgab893. |
| 248 | van Schaik J, van Roessel I, Schouten-van Meeteren N, van Iersel L, Clement SC, Boot AM, et al. High Prevalence of Weight Gain in Childhood Brain Tumor Survivors and Its Association With Hypothalamic-Pituitary Dysfunction. Journal of clinical oncology : official journal of the American Society of Clinical Oncology. 2021;39(11):1264-73. |
| 249 | Tao C, Gu D, Huang R, Zhou L, Hu Z, Chen Y, et al. Hippocampus segmentation after brain tumor resection via postoperative region synthesis. BMC medical imaging. 2023;23(1):142. doi: 10.1186/s12880-023-01087-2. |
| 250 | Zeng W, Kwan Law BY, Wai Wong VK, Bik Chan DS, Fai Mok SW, Ying Gao JJ, et al. HM30181A, a potent P-glycoprotein inhibitor, potentiates the absorption and in vivo antitumor efficacy of paclitaxel in an orthotopic brain tumor model. Cancer biology & medicine. 2020;17(4):986-1001. |
| 251 | Wang X, Duan X, Yang G, Zhang X, Deng L, Zheng H, et al. Honokiol crosses BBB and BCSFB, and inhibits brain tumor growth in rat 9l intracerebral gliosarcoma model and human U251 xenograft glioma model. PloS one. 2011;6(4). doi: 10.1371/journal.pone.0018490. |
| 252 | Wolf S, Riess A, Landscheidt JF, Lumenta CB, Schürer L, Friederich P. How to perform indexing of extravascular lung water: A validation study. Critical care medicine. 2013;41(4):990-8. doi: 10.1097/CCM.0b013e318275cd75. |
| 253 | Yu Z, Li X, Li J, Chen W, Tang Z, Geng D. HSA-net with a novel CAD pipeline boosts both clinical brain tumor MR image classification and segmentation. Computers in biology and medicine. 2024;170. doi: 10.1016/j.compbiomed.2024.108039. |
| 254 | Neal JH, Cotman CW, Olson JJ, Westphal M. Human brain tumor cyst fluid is mitogenic for primary astrocytes in culture. Neurosurgery. 1993;32(1):95-9. |
| 255 | Stromberg K, Hudgins WR, Dorman LS. Human brain tumor-associated urinary high molecular weight transforming growth factor: A high molecular weight form of epidermal growth factor. Cancer research. 1987;47(4):1190-6. |
| 256 | Rajaraman P. Hunting for the causes of meningioma--obesity is a suspect. Cancer prevention research (Philadelphia, Pa). 2011;4(9):1353-5. Epub 2011/09/07. doi: 10.1158/1940-6207.Capr-11-0360. PubMed PMID: 21893497; PubMed Central PMCID: PMC3170083. |
| 257 | Dube B, Pandey A, Joshi G, Sawant K. Hydrophobically modified polyethylenimine-based ternary complexes for targeting brain tumor: stability, in vitro and in vivo studies. Artificial Cells, Nanomedicine and Biotechnology. 2017;45(8):1685-98. doi: 10.1080/21691401.2017.1282497. |
| 258 | Schrell UM, Rittig MG, Anders M, Kiesewetter F, Marschalek R, Koch UH, et al. Hydroxyurea for treatment of unresectable and recurrent meningiomas. I. Inhibition of primary human meningioma cells in culture and in meningioma transplants by induction of the apoptotic pathway. Journal of neurosurgery. 1997;86(5):845-52. |
| 259 | Gelbart M, Nahum E, Gelbart M, Kaplan E, Kadmon G, Kershenovich A, et al. Hyperlactatemia in children following brain tumor resection: prevalence, associated factors, and clinical significance. Child's Nervous System. 2022;38(4):739-45. doi: 10.1007/s00381-021-05424-0. |
| 260 | Sahmoun AE, Case LD, Chavour S, Kareem S, Schwartz GG. Hypertension and risk of brain metastasis from small cell lung cancer: A retrospective follow-up study. Anticancer research. 2004;24(5 B):3115-20. |
| 261 | Hochberg I, Hochberg Z. Hypothalamic obesity. Endocrine development. 2010;17:185-96. Epub 2009/12/04. doi: 10.1159/000262539. PubMed PMID: 19955767. |
| 262 | Quigg TC, Haddad NG, Buchsbaum JC, Shih CS. Hypothalamic obesity syndrome: Rare presentation of CNS+ B-cell lymphoblastic lymphoma. Pediatric Blood and Cancer. 2012;59(5):930-3. doi: 10.1002/pbc.24058. |
| 263 | Giustozzi M, Proietti G, Becattini C, Roila F, Agnelli G, Mandalà M. ICH in primary or metastatic brain cancer patients with or without anticoagulant treatment: a systematic review and meta-analysis. Blood advances. 2022;6(16):4873-83. doi: 10.1182/bloodadvances.2022008086. |
| 264 | Mathew M, Salahuddin A, Mathew NR, Nandhagopal R. Idiopathic intracranial hypertension presenting as postpartum headache. Neurosciences. 2016;21(1):52-5. doi: 10.17712/nsj.2016.1.20150304. PubMed Central PMCID: Pfizer(Canada) |
| 265 | Hajnzić TF, Kastelan M, Lukac J, Hajnzić T. Immunocompetent cells and lymphocyte reactivity to mitogens in levamisole-treated brain tumor children. Pediatric hematology and oncology. 1999;16(4):335-40. Epub 1999/07/17. doi: 10.1080/088800199277164. PubMed PMID: 10407870. |
| 266 | Basu PS, Majhi R, Ghosh S, Ghosh B, Batabyal SK. Immunodiagnosis of the primary brain tumor (glioma) by the endogenous lectin. Clinica Chimica Acta. 2002;317(1-2):177-80. doi: 10.1016/S0009-8981(01)00785-9. |
| 267 | Murakami M, Ushio Y, Morino Y, Ohta T, Matsukado Y. Immunohistochemical localization of apolipoprotein E in human glial neoplasms. Journal of Clinical Investigation. 1988;82(1):177-88. doi: 10.1172/JCI113568. |
| 268 | Strobel K, Simpson P, Donohoue PA, Firat S, Jogal S. Impact of Age at Diagnosis and Hypothalamic Involvement on Body Mass Index Z-Score Change in Pediatric Brain Tumor Survivors. Hormone research in paediatrics. 2016;85(6):389-95. doi: 10.1159/000445890. |
| 269 | Strobel K, Simpson P, Donohoue P, Firat S, Jogal S. Impact of age at diagnosis on obesity in pediatric brain tumor survivors. Neuro-oncology. 2014;16:v49. doi: 10.1093/neuonc/nou243.17. |
| 270 | Strobel K, Simpson P, Donohoue P, Firat S, Jogal S. Impact of age at diagnosis on obesity in pediatric brain tumor survivors. Neuro-oncology. 2014;16:i111. doi: 10.1093/neuonc/nou079. |
| 271 | Boviatsis EJ, Bouras TI, Kouyialis AT, Themistocleous MS, Sakas DE. Impact of age on complications and outcome in meningioma surgery. Surgical neurology. 2007;68(4):407-11. doi: 10.1016/j.surneu.2006.11.071. |
| 272 | Gheorghiu A, Brunborg C, Johannesen TB, Helseth E, Zwart JA, Wiedmann MKH. The impact of body mass index and height on risk for primary tumours of the spinal cord, spinal meninges, spinal and peripheral nerves in 1.7 million norwegian women and men: a prospective cohort study. Acta Oncologica. 2022;61(1):1-6. |
| 273 | Popovic V, Pekic S, Golubicic I, Doknic M, Dieguez C, Casanueva FF. The impact of cranial irradiation on GH responsiveness to GHRH plus GH-releasing peptide-6. Journal of Clinical Endocrinology and Metabolism. 2002;87(5):2095-9. |
| 274 | Karhade AV, Fandino L, Gupta S, Cote DJ, Iorgulescu JB, Broekman ML, et al. Impact of operative length on post-operative complications in meningioma surgery: a NSQIP analysis. Journal of neuro-oncology. 2017;131(1):59-67. doi: 10.1007/s11060-016-2262-2. |
| 275 | Norman S, Ramos A, Giantini Larsen AM, Bander E, Goldberg J, Parker W, et al. Impact of the COVID-19 pandemic on neuro-oncology outcomes. Journal of neuro-oncology. 2021;154(3):375-81. doi: 10.1007/s11060-021-03838-z. |
| 276 | Colao A, Cuocolo A, Di Somma C, Cerbone G, Della Morte AM, Nicolai E, et al. Impaired cardiac performance in elderly patients with growth hormone deficiency. Journal of Clinical Endocrinology and Metabolism. 1999;84(11):3950-5. |
| 277 | Jung IH, Yoo J, Roh TH, Park HH, Hong CK. Importance of Sufficient Petrosectomy in an Anterior Petrosal Approach: Relightening of the Kawase Pyramid. World neurosurgery. 2021;153:e11-e9. doi: 10.1016/j.wneu.2021.05.017. |
| 278 | Gleeson HK, Stoeter R, Ogilvy-Stuart AL, Gattamaneni HR, Brennan BM, Shalet SM. Improvements in final height over 25 years in growth hormone (GH) -deficient childhood survivors of brain tumors receiving GH replacement. Journal of Clinical Endocrinology and Metabolism. 2003;88(8):3682-9. |
| 279 | Manjunath C, Mahurkar N. In vitro cytotoxicity of cardamom oil, lemon oil, and jasmine oil on human skin, gastric, and brain cancer cell line. Journal of cancer research and therapeutics. 2021;17(1):62-8. doi: 10.4103/jcrt.JCRT_915_17. PubMed Central PMCID: Sigma Aldrich(United States). |
| 280 | Brady ML, Grondin R, Zhang Z, Pomerleau F, Powell D, Huettl P, et al. In-vitro and in-vivo performance studies of a porous infusion catheter designed for intraparenchymal delivery of therapeutic agents of varying size. Journal of neuroscience methods. 2022;378. doi: 10.1016/j.jneumeth.2022.109643. |
| 281 | Nakano F, Matsubara T, Ishigaki T, Hatazaki S, Mouri G, Nakatsuka Y, et al. Incidence and risk factor of deep venous thrombosis in patients undergoing craniotomy for brain tumors: A Japanese single-center, retrospective study. Thrombosis research. 2018;165:95-100. doi: 10.1016/j.thromres.2018.03.016. |
| 282 | Shew M, Kavookjian H, Dahlstrom K, Muelleman T, Lin J, Camarata P, et al. Incidence and Risk Factors for Sigmoid Venous Thrombosis Following CPA Tumor Resection. Otology & neurotology : official publication of the American Otological Society, American Neurotology Society [and] European Academy of Otology and Neurotology. 2018;39(5):e376-e80. |
| 283 | Hoefnagel D, Kwee LE, Van Putten EHP, Kros JM, Dirven CMF, Dammers R. The incidence of postoperative thromboembolic complications following surgical resection of intracranial meningioma. A retrospective study of a large single center patient cohort. Clinical neurology and neurosurgery. 2014;123:150-4. |
| 284 | Klauschie J, Rose SR. Incidence of short stature in children with hydrocephalus. Journal of Pediatric Endocrinology and Metabolism. 1996;9(2):181-7. |
| 285 | Forsgren L, Nystrom L. An incident case-referent study of epileptic seizures in adults. Epilepsy research. 1990;6(1):66-81. doi: 10.1016/0920-1211(90)90010-S. |
| 286 | Aghi MK, Eskandar EN, Carter BS, Curry WT, Jr., Barker FG, 2nd. Increased prevalence of obesity and obesity-related postoperative complications in male meningioma patients. Clinical neurosurgery. 2007;54:236-40. Epub 2008/05/29. PubMed PMID: 18504925. |
| 287 | Aghi MK, Eskandar EN, Carter BS, Curry WT, Jr., Barker FG, 2nd. Increased prevalence of obesity and obesity-related postoperative complications in male patients with meningiomas. Neurosurgery. 2007;61(4):754-60; discussion 60-1. |
| 288 | Aghi MK, Eskandar EN, Carter BS, Curry WT, Barker FG. Increased prevalence of obesity and obesity-related postoperative complications in male patients with meningiomas. Neurosurgery. 2007;61(4):754-60. doi: 10.1227/01.NEU.0000298903.63635.E3. |
| 289 | Cheng TJ, Chen TM, Chen CH, Lai YK. Induction of stress response and differential expression of 70 kDa stress proteins by sodium fluoride in HeLa and rat brain tumor 9L cells. Journal of cellular biochemistry. 1998;69(2):221-31. |
| 290 | Silbergeld DL, Ali-Osman F, Winn HR. Induction of transformational changes in normal endothelial cells by cultured human astrocytoma cells. Journal of neurosurgery. 1991;75(4):604-12. doi: 10.3171/jns.1991.75.4.0604. |
| 291 | Claes Y, Van Hemelrijck J, Van Gerven M, Arnout J, Vermylen J, Weidler B, et al. Influence of hydroxyethyl starch on coagulation in patients during the perioperative period. Anesthesia and analgesia. 1992;75(1):24-30. doi: 10.1213/00000539-199207000-00005. |
| 292 | Bi Y, Shen C, Li C, Liu Y, Gao D, Shi C, et al. Inhibition of autophagy induced by quercetin at a late stage enhances cytotoxic effects on glioma cells. Tumor Biology. 2016;37(3):3549-60. doi: 10.1007/s13277-015-4125-4.. |
| 293 | Oga M, Takenaga K, Sato Y, Nakajima H, Koshikawa N, Osato K, et al. Inhibition of metastatic brain tumor growth by intramuscular administration of the endostatin gene. International journal of oncology. 2003;23(1):73-9. |
| 294 | Lv H, Zhang X, Sharma J, Reddy MV, Reddy EP, Gallo JM. Integrated pharmacokinetic-driven approach to screen candidate anticancer drugs for brain tumor chemotherapy. The AAPS journal. 2013;15(1):250-7. Epub 2012/11/28. doi: 10.1208/s12248-012-9428-4. |
| 295 | Anselmino M, Marcantoni L, Agresta A, Chieffo E, Floris R, Racheli M, et al. Interventional cardiology and X-ray exposure of the head: overview of clinical evidence and practical implications. Journal of Cardiovascular Medicine. 2022;23(6):353-8. doi: 10.2459/JCM.0000000000001262. |
| 296 | Lum MA, Martin AJ, Alexander MD, McCoy DB, Cooke DL, Lillaney P, et al. Intra-arterial MR perfusion imaging of meningiomas: Comparison to digital subtraction angiography and intravenous mr perfusion imaging. PloS one. 2016;11(11). doi: 10.1371/journal.pone.0163554. |
| 297 | Peng B, Zhao X, Yang MS, Li LL. Intracellular transglutaminase-catalyzed polymerization and assembly for bioimaging of hypoxic neuroblastoma cells. Journal of materials chemistry B. 2019;7(37):5626-32. doi: 10.1039/c9tb01227c. |
| 298 | Leader A, Hamulyák EN, Carney BJ, Avrahami M, Knip JJ, Rozenblatt S, et al. Intracranial hemorrhage with direct oral anticoagulants in patients with brain metastases. Blood advances. 2020;4(24):6291-7. Epub 2020/12/23. doi: 10.1182/bloodadvances.2020003238. |
| 299 | Leader A, Hamulyák EN, Carney BJ, Avrahami M, Knip JJ, Rozenblatt S, et al. Intracranial hemorrhage with direct oral anticoagulants in patients with brain metastases. Blood advances. 2020;4(24):6291-7. doi: 10.1182/bloodadvances.2020003238. |
| 300 | Carney BJ, Uhlmann EJ, Puligandla M, Mantia C, Weber GM, Neuberg DS, et al. Intracranial hemorrhage with direct oral anticoagulants in patients with brain tumors. Journal of Thrombosis and Haemostasis. 2019;17(1):72-6. doi: 10.1111/jth.14336. |
| 301 | Kimura H, Nakagawa K, Sakaki S, Matsuoka K. Intracranial meningioma of an infant: a case report. No shinkei geka Neurological surgery. 1987;15(6):663-8. |
| 302 | Pompili A, Cacciani L, Cattani F, Caroli F, Crecco M, Mastrostefano R, et al. [Intracranial meningiomas in the elderly]. Minerva medica. 1997;88(6):229-36. Epub 1997/06/01. PubMed PMID: 9280865. |
| 303 | Kremer P, Fardanesh M, Ding R, Pritsch M, Zoubaa S, Frei E. Intraoperative fluorescence staining of malignant brain tumors using 5-aminofluorescein-labeled albumin. Neurosurgery. 2009;64(3 Suppl):ons53-60; discussion ons- |
| 304 | Arai T, Tani S, Isoshima A, Nagashima H, Joki T, Takahashi-Fujigasaki J, et al. Intraoperative photodynamic diagnosis for spinal ependymoma using 5-aminolevulinic acid: Technical note. Neurological Surgery. 2006;34(8):811-7. |
| 305 | Joó GJ, Reiniger L, Papp C, Csaba A, Komáromy H, Rigó J, Jr. Intrauterine diagnosis and pathology of fetal choroid plexus carcinoma--a case study. Pathology, research and practice. 2014;210(12):1156-9. Epub 2014/07/19. doi: 10.1016/j.prp.2014.01.009. PubMed PMID: 25034609. |
| 306 | Theakstone AG, Brennan PM, Jenkinson MD, Goodacre R, Baker MJ. Investigating centrifugal filtration of serum-based FTIR spectroscopy for the stratification of brain tumours. PloS one. 2023;18(2 February). doi: 10.1371/journal.pone.0279669. |
| 307 | Hatlen RR, Rajagopalan P. Investigating Trans-differentiation of Glioblastoma Cells in an in Vitro 3D Model of the Perivascular Niche. ACS Biomaterials Science and Engineering. 2023;9(6):3445-61. doi: 10.1021/acsbiomaterials.2c01310. |
| 308 | Kobata T, Maeda Y, Monden T, Morimoto M, Oomori K. Investigation of Radioactivity Concentration of the Normal Brain Region for the Phantom Experiment in Brain Tumor PET Imaging. Nihon Hoshasen Gijutsu Gakkai zasshi. 2019;75(8):771-6. doi: 10.6009/jjrt.2019_JSRT_75.8.771. |
| 309 | Kayali H, Onguru O, Erdogan E, Sirin S, Timurkaynak E. Isolated intracranial Rosai-Dorfman disease mimicking meningioma. Clinical neuropathology. 2004;23(5):204-8. |
| 310 | da Silveira EF, Chassot JM, Teixeira FC, Azambuja JH, Debom G, Beira FT, et al. Ketoprofen-loaded polymeric nanocapsules selectively inhibit cancer cell growth in vitro and in preclinical model of glioblastoma multiforme. Investigational new drugs. 2013;31(6):1424-35. |
| 311 | Sobstyl M, Nagańska E, Glinka P, Wierzba-Bobrowicz T, Acewicz A, Kuls-Oszmaniec A. Large haemorrhage within glioblastoma mimicking haemorrhagic stroke and coexistance of meningioma: a case of collision tumours. Folia neuropathologica. 2023;61(4):433-41. doi: 10.5114/fn.2023.131640. |
| 312 | Jussila MP, Remes T, Anttonen J, Harila-Saari A, Niinimäki J, Pokka T, et al. Late vertebral side effects in long-term survivors of irradiated childhood brain tumor. PloS one. 2018;13(12). doi: 10.1371/journal.pone.0209193. |
| 313 | Cai J, He Z, Zheng Z, Xu Q, Hu C, Huo M. Learning global dependencies based on hierarchical full connection for brain tumor segmentation. Computer methods and programs in biomedicine. 2022;221. doi: 10.1016/j.cmpb.2022.106925. |
| 314 | Bikis C, Tzanavari T, Alexandraki KI, Theocharis S. Leptin Involvement in Primary Brain and Pituitary Tumors: Therapeutic Potential, Prognostic Value, and Proposed Diagnostic Application. Hormones and Cancer. 2018;9(3):144-55. doi: 10.1007/s12672-018-0327-8. |
| 315 | Benson VS, Pirie K, Green J, Casabonne D, Beral V. Lifestyle factors and primary glioma and meningioma tumours in the Million Women Study cohort. British journal of cancer. 2008;99(1):185-90. doi: 10.1038/sj.bjc.6604445. |
| 316 | Yu X, Wu Y, Bai Y, Han H, Chen L, Gao H, et al. A lightweight 3D UNet model for glioma grading. Physics in medicine and biology. 2022;67(15). doi: 10.1088/1361-6560/ac7d33. |
| 317 | Blijdorp K, van den Heuvel-Eibrink M, Pieters R, Boot A, Sluimer J, van der Lelij AJ, et al. The limited screening value of insulin-like growth factor-I as a marker for alterations in body composition in very long-term adult survivors of childhood cancer. Pediatric blood & cancer. 2012;59(4):711-6. |
| 318 | Blijdorp K, van den Heuvel-Eibrink M, Pieters R, Boot A, Sluimer J, van der Lelij AJ, et al. The limited screening value of insulin-like growth factor-i as a marker for alterations in body composition in very long-term adult survivors of childhood cancer. Pediatric Blood and Cancer. 2012;59(4):711-6. |
| 319 | Dimeco F, Li KW, Tyler BM, Wolf AS, Brem H, Olivi A. Local delivery of mitoxantrone for the treatment of malignant brain tumors in rats. Journal of neurosurgery. 2002;97(5):1173-8. doi: 10.3171/jns.2002.97.5.1173. PubMed Central PMCID: Sigma Aldrich(United States). |
| 320 | Heikens J, Ubbink MC, van der Pal HP, Bakker PJ, Fliers E, Smilde TJ, et al. Long term survivors of childhood brain cancer have an increased risk for cardiovascular disease. Cancer. 2000;88(9):2116-21. Epub 2000/05/17. PubMed PMID: 10813724. |
| 321 | Wójcik M, Dolezal-Ołtarzewska K, Kumorowicz-Czoch M, Kalicka-Kasperczyk A, Januś D, Zygmunt-Górska A, et al. [Long-term endocrine complications after brain tumor treatment--own experience]. Przegla̧d lekarski. 2010;67(11):1132-5. |
| 322 | Rutter MM, Rose SR. Long-term endocrine sequelae of childhood cancer. Current opinion in pediatrics. 2007;19(4):480-7. doi: 10.1097/MOP.0b013e3282058b56. |
| 323 | Jørgensen JT, Johansen MS, Ravnskjær L, Andersen KK, Bräuner EV, Loft S, et al. Long-term exposure to ambient air pollution and incidence of brain tumours: The Danish Nurse Cohort. Neurotoxicology. 2016;55:122-30. doi: 10.1016/j.neuro.2016.06.003. |
| 324 | Cochrane AM, Cheung C, Rangan K, Freyer D, Nahata L, Dhall G, et al. Long-term follow-up of endocrine function among young children with newly diagnosed malignant central nervous system tumors treated with irradiation-avoiding regimens. Pediatric Blood and Cancer. 2017;64(11). doi: 10.1002/pbc.26616. |
| 325 | Cochrane A, Imam S, Hiramanek R, Cheung C, Rangan K, Castillo E, et al. Long-term follow-up of endocrine function among young children with newly-diagnosed malignant central nervous system (CNS) tumors treated with irradiation-avoiding regimens: The Children's Hospital Los Angeles (CHLA) experience. Neuro-oncology. 2014;16:v46. doi: 10.1093/neuonc/nou243.3. |
| 326 | Schultes B, Ernst B. Long-term outcomes after gastric bypass surgery in patients with craniopharyngioma-related hypothalamic obesity-Three cases with 7, 8, and 14 years follow-up. Obesity research & clinical practice. 2023;17(4):361-5. |
| 327 | Hu Q, Zhao J, Xu J, Zhou X, Shui Y, Shen L, et al. Long-Term Relief of Cerebral Radiation Necrosis Treated with Low-Dose Bevacizumab-a Report of 2 Cases. Oncology research and treatment. 2017;40(3):133-7. doi: 10.1159/000455868. |
| 328 | Sato J, Shimamura N, Naraoka M, Terui K, Asano K, Itou E, et al. Long-term tumor-free survival case of congenital embryonal tumor with various pathological components. Child's Nervous System. 2013;29(6):921-6. doi: 10.1007/s00381-013-2052-5. |
| 329 | Johnson MD, Woodard A, Okediji EJ, Toms SA, Allen GS. Lovastatin is a potent inhibitor of meningioma cell proliferation: Evidence for inhibition of a mitogen associated protein kinase. Journal of neuro-oncology. 2002;56(2):133-42. doi: 10.1023/A:1014588214966. |
| 330 | Haj-Hosseini N, Richter JC, Hallbeck M, Wårdell K. Low dose 5-aminolevulinic acid: Implications in spectroscopic measurements during brain tumor surgery. Photodiagnosis and photodynamic therapy. 2015;12(2):209-14. Epub 2015/03/31. doi: 10.1016/j.pdpdt.2015.03.004. PubMed PMID: 25818546. |
| 331 | Mohr SB, Gorham ED, Garland CF, Grant WB, Garland FC. Low ultraviolet B and increased risk of brain cancer: An ecological study of 175 countries. Neuroepidemiology. 2010;35(4):281-90. doi: 10.1159/000314350. |
| 332 | Mareninov S, De Jesus J, Sanchez DE, Kay AB, Wilson RW, Babic I, et al. Lyophilized brain tumor specimens can be used for histologic, nucleic acid, and protein analyses after 1 year of room temperature storage. Journal of neuro-oncology. 2013;113(3):365-73. doi: 10.1007/s11060-013-1135-1. |
| 333 | Cho HR, Kim DH, Kim D, Doble P, Bishop D, Hare D, et al. Malignant Glioma: MR imaging by using 5-aminolevulinic acid in an animal model. Radiology. 2014;272(3):720-30. doi: 10.1148/radiol.14131459. |
| 334 | Knovich MA, Lesser GJ. The management of thromboembolic disease in patients with central nervous system malignancies. Current treatment options in oncology. 2004;5(6):511-7. doi: 10.1007/s11864-004-0039-x. |
| 335 | Pham HN, Goldberg RJ, Pham LQ, Nguyen HL, Pham DA, Mai LTT, et al. Maternal and Perinatal Factors Associated With Childhood Brain Tumors: A Case-Control Study in Vietnam. Cancer control : journal of the Moffitt Cancer Center. 2024;31. doi: 10.1177/10732748241258602. |
| 336 | Wu DM, Hong XW, Wen X, Han XR, Wang S, Wang YJ, et al. MCL1 gene silencing promotes senescence and apoptosis of glioma cells via inhibition of the PI3K/Akt signaling pathway. IUBMB life. 2019;71(1):81-92. doi: 10.1002/iub.1944. |
| 337 | Pruitt AA. Medical management of patients with brain tumors. Current Treatment Options in Neurology. 2011;13(4):413-26. doi: 10.1007/s11940-011-0132-y. |
| 338 | Wen PY, Schiff D, Kesari S, Drappatz J, Gigas DC, Doherty L. Medical management of patients with brain tumors. Journal of neuro-oncology. 2006;80(3):313-32. doi: 10.1007/s11060-006-9193-2. |
| 339 | Almanza-Aguilera E, Cano A, Gil-Lespinard M, Burguera N, Zamora-Ros R, Agudo A, et al. Mediterranean diet and olive oil, microbiota, and obesity-related cancers. From mechanisms to prevention. Seminars in cancer biology. 2023;95:103-19. doi: 10.1016/j.semcancer.2023.08.001. |
| 340 | Tabori U, Sung L, Hukin J, Laperriere N, Crooks B, Carret AS, et al. Medulloblastoma in the second decade of life: A specific group with respect to toxicity and management: A Canadian pediatric brain tumor consortium study. Cancer. 2005;103(9):1874-80. doi: 10.1002/cncr.21003. |
| 341 | Takahashi H, Cornish AJ, Sud A, Law PJ, Disney-Hogg L, Calvocoressi L, et al. Mendelian randomization provides support for obesity as a risk factor for meningioma. Scientific reports. 2019;9(1):309. doi: 10.1038/s41598-018-36186-6. |
| 342 | Chang CC, Chang YY, Lui CC, Huang CC, Liu JS. Meningeal hemangiopericytoma with delayed multiple distant metastases. Journal of the Chinese Medical Association. 2004;67(10):527-32. |
| 343 | Wahab M, Al-Azzawi F. Meningioma and hormonal influences. Climacteric : the journal of the International Menopause Society. 2003;6(4):285-92. doi: 10.1080/cmt.6.4.285.292. |
| 344 | Nakamura Y, Shimizu T, Ohigashi Y, Itou N, Ishikawa Y. Meningioma arising in Werner syndrome confirmed by mutation analysis. Journal of Clinical Neuroscience. 2005;12(4):503-6. doi: 10.1016/j.jocn.2003.12.022. |
| 345 | Du Z, Brewster R, Merrill PH, Chmielecki J, Francis J, Aizer A, et al. Meningioma transcription factors link cell lineage with systemic metabolic cues. Neuro-oncology. 2018;20(10):1331-43. doi: 10.1093/neuonc/noy057. |
| 346 | Rath SR, Long TM, Bear NL, Miles GCP, Bullock AM, Gottardo NG, et al. Metabolic and Psychological Impact of a Pragmatic Exercise Intervention Program in Adolescent and Young Adult Survivors of Pediatric Cancer-Related Cerebral Insult. Journal of adolescent and young adult oncology. 2018;7(3):349-57. doi: 10.1089/jayao.2017.0105. |
| 347 | Papalia H, Rochette E, Pereira B, Merlin E, Kanold J, Duché P. Metabolic response to exercise in childhood brain tumor survivors: A pilot controlled study. Pediatric Blood and Cancer. 2020;67(2). doi: 10.1002/pbc.28053. |
| 348 | Di Iorgi N, Morsellino V, Salem SD, Gallizia A, Ceroni F, Pistorio A, et al. Metabolic syndrome (MS) and cardiovascular risk (CV) in peripubertal and adolescent childhood cancer survivors (CCS). Hormone research in paediatrics. 2017;88:340-1. doi: 10.1159/000481424. |
| 349 | Nia AM, Branch DW, Maynard K, Frank T, Zavlin D, Patterson JT, et al. Metabolic Syndrome Associated with Increased Rates of Medical Complications After Intracranial Tumor Resection. World neurosurgery. 2019;126:e1055-e62. doi: 10.1016/j.wneu.2019.03.046. |
| 350 | Seliger C, Meier CR, Becker C, Jick SS, Proescholdt M, Bogdahn U, et al. Metabolic syndrome in relation to risk of meningioma. Oncotarget. 2017;8(2):2284-92. doi: 10.18632/oncotarget.13667. |
| 351 | Horne VE, Bielamowicz K, Nguyen J, Hilsenbeck S, Lindsay H, Sonabend R, et al. Methylphenidate improves weight control in childhood brain tumor survivors with hypothalamic obesity. Pediatric Blood and Cancer. 2020;67(7). doi: 10.1002/pbc.28379. |
| 352 | Pinheiro PS, Callahan KE, Stern MC, de Vries E. Migration from Mexico to the United States: A high-speed cancer transition. International journal of cancer. 2018;142(3):477-88. doi: 10.1002/ijc.31068. |
| 353 | Landrigan PJ, Raps H, Cropper M, Bald C, Brunner M, Canonizado EM, et al. The Minderoo-Monaco Commission on Plastics and Human Health. Annals of global health. 2023;89(1). doi: 10.5334/aogh.4056. |
| 354 | Samanic C, Teer J, Thompson Z, Creed J, Fridley B, Nabors LB, et al. Mitochondrial dna sequence variation and meningioma. Neuro-oncology. 2021;23(SUPPL 6):vi88. doi: 10.1093/neuonc/noab196.345. |
| 355 | Samanic CM, Teer JK, Thompson ZJ, Creed JH, Mokhtari S, Fridley BL, et al. Mitochondrial DNA sequence variation and risk of meningioma. Journal of neuro-oncology. 2021;155(3):319-24. doi: 10.1007/s11060-021-03878-5. |
| 356 | Espinosa I, D'Angelo E, Corominas M, Gonzalez A, Prat J. Mixed endometrial carcinomas with a "low-grade serous"-like component: a clinicopathologic, immunohistochemical, and molecular genetic study. Human pathology. 2018;71:65-73. |
| 357 | Seipel K, Messerli C, Wiedemann G, Bacher U, Pabst T. MN1, FOXP1 and hsa-miR-181a-5p as prognostic markers in acute myeloid leukemia patients treated with intensive induction chemotherapy and autologous stem cell transplantation. Leukemia research. 2020;89. doi: 10.1016/j.leukres.2020.106296. |
| 358 | Yoshikawa MH, Rabelo NN, Telles JPM, Figueiredo EG. Modifiable risk factors for glioblastoma: a systematic review and meta-analysis. Neurosurgical review. 2023;46(1). doi: 10.1007/s10143-023-02051-y. |
| 359 | Noda S, El-Jawahri A, Patel D, Lautenschlaeger T, Siedow M, Chakravarti A. Molecular Advances of Brain Tumors in Radiation Oncology. Seminars in radiation oncology. 2009;19(3):171-8. doi: 10.1016/j.semradonc.2009.02.005. PubMed Central PMCID: Imclone(United States) |
| 360 | Nishihara R, VanderWeele TJ, Shibuya K, Mittleman MA, Wang M, Field AE, et al. Molecular pathological epidemiology gives clues to paradoxical findings. European journal of epidemiology. 2015;30(10):1129-35. doi: 10.1007/s10654-015-0088-4. |
| 361 | Tsai SP, Wendt JK, Cardarelli KM, Fraser AE. A mortality and morbidity study of refinery and petrochemical employees in Louisiana. Occupational and environmental medicine. 2003;60(9):627-33. doi: 10.1136/oem.60.9.627. |
| 362 | Held P, Fellner C, Fellner F, Seitz J, Graf S, Hilbert M, et al. MRI of inner ear and facial nerve pathology using 3D MP-RAGE and 3D CISS sequences. British Journal of Radiology. 1997;70(JUNE):558-66. doi: 10.1259/bjr.70.834.9227246. |
| 363 | Chen W, Zhou W, Zhu L, Cao Y, Gu H, Yu B. MTDCNet: A 3D multi-threading dilated convolutional network for brain tumor automatic segmentation. Journal of biomedical informatics. 2022;133. doi: 10.1016/j.jbi.2022.104173. |
| 364 | Pachow D, Andrae N, Kliese N, Angenstein F, Stork O, Wilisch-Neumann A, et al. MTORC1 inhibitors suppress meningioma growth in mouse models. Clinical Cancer Research. 2013;19(5):1180-9. doi: 10.1158/1078-0432.CCR-12-1904. |
| 365 | Li W, Wu C, Yao Y, Dong B, Wei Z, Lv X, et al. MUC4 modulates human glioblastoma cell proliferation and invasion by upregulating EGFR expression. Neuroscience letters. 2014;566:82-7. doi: 10.1016/j.neulet.2014.02.033. |
| 366 | Kolakshyapati M, Ikawa F, Abiko M, Mitsuhara T, Kinoshita Y, Takeda M, et al. Multivariate risk factor analysis and literature review of postoperative deterioration in Karnofsky Performance Scale score in elderly patients with skull base meningioma. Neurosurgical focus. 2018;44(4):E14. doi: 10.3171/2018.1.FOCUS17730. |
| 367 | Zahedi M, Hizomi Arani R, Tohidi M, Haghighi S, Mehrpour M, Hadaegh F. Nasopharyngeal B-cell lymphoma with pan-hypopituitarism and oculomotor nerve palsy: a case report and review of the literature. BMC endocrine disorders. 2020;20(1). doi: 10.1186/s12902-020-00644-y. |
| 368 | Wang P, Zheng SY, Jiang RL, Wu HD, Li YA, Lu JL, et al. Necroptosis signaling and mitochondrial dysfunction cross-talking facilitate cell death mediated by chelerythrine in glioma. Free Radical Biology and Medicine. 2023;202:76-96. doi: 10.1016/j.freeradbiomed.2023.03.021. |
| 369 | Bhatti P, Doody DR, McKean-Cowdin R, Mueller BA. Neonatal vitamin D and childhood brain tumor risk. International journal of cancer. 2015;136(10):2481-5. doi: 10.1002/ijc.29291. |
| 370 | Ouafik L, Sauze S, Boudouresque F, Chinot O, Delfino C, Fina F, et al. Neutralization of adrenomedullin inhibits the growth of human glioblastoma cell lines in vitro and suppresses tumor xenograft growth in vivo. American Journal of Pathology. 2002;160(4):1279-92. |
| 371 | Sathornsumetee S, Rich JN. New approaches to primary brain tumor treatment. Anti-cancer drugs. 2006;17(9):1003-16. doi: 10.1097/01.cad.0000231473.00030.1f. PubMed Central PMCID: Antisense Pharma(Germany) |
| 372 | Wetterau L, Cohen P. New paradigms for growth hormone therapy in children. Hormone research. 2000;53(SUPPL. 3):31-6. doi: 10.1159/000023530. |
| 373 | Sathornsumetee S, Rich JN. New treatment strategies for malignant gliomas. Expert review of anticancer therapy. 2006;6(7):1087-104. doi: 10.1586/14737140.6.7.1087. PubMed Central PMCID: Astra Zeneca(United States) |
| 374 | Pugh MJ, Knoefel JE, Mortensen EM, Amuan ME, Berlowitz DR, Van Cott AC. New-onset epilepsy risk factors in older veterans. Journal of the American Geriatrics Society. 2009;57(2):237-42. Epub 2009/02/12. doi: 10.1111/j.1532-5415.2008.02124.x. PubMed PMID: 19207140. |
| 375 | Dinkhuysen JJ, Contreras C, Cipullo R, Finger MA, Rossi J, Manrique R, et al. Non Working Beating Heart: a new strategy of myocardial protection during heart transplant. Revista brasileira de cirurgia cardiovascular : orgao oficial da Sociedade Brasileira de Cirurgia Cardiovascular. 2011;26(4):630-4. |
| 376 | Dinkhuysen JJ, Contreras C, Cipullo R, Finger MA, Rossi J, Manrique R, et al. Non Working Beating Heart: A new strategy of myocardial protection during heart transplant. Brazilian Journal of Cardiovascular Surgery. 2011;26(4):630-4. doi: 10.5935/1678-9741.20110055. |
| 377 | Lakomkin N, Hadjipanayis C. Non-routine discharge disposition is associated with post-discharge complications and 30-day readmissions following craniotomy for brain tumor resection. Neuro-oncology. 2017;19:vi109. doi: 10.1093/neuonc/nox168. |
| 378 | Lakomkin N, Hadjipanayis CG. Non-routine discharge disposition is associated with post-discharge complications and 30-day readmissions following craniotomy for brain tumor resection. Journal of neuro-oncology. 2018;136(3):595-604. doi: 10.1007/s11060-017-2689-0. |
| 379 | Kato Y, Holm DA, Okollie B, Artemov D. Noninvasive detection of temozolomide in brain tumor xenografts by magnetic resonance spectroscopy. Neuro-oncology. 2010;12(1):71-9. doi: 10.1093/neuonc/nop006. PubMed Central PMCID: Bayer(Antigua and Barbuda). |
| 380 | Gravina GL, Mancini A, Colapietro A, Vitale F, Vetuschi A, Pompili S, et al. The novel CXCR4 antagonist, PRX177561, reduces tumor cell proliferation and accelerates cancer stem cell differentiation in glioblastoma preclinical models. Tumor Biology. 2017;39(6). |
| 381 | Jiang M, Zhai F, Kong J. A novel deep learning model DDU-net using edge features to enhance brain tumor segmentation on MR images. Artificial intelligence in medicine. 2021;121. doi: 10.1016/j.artmed.2021.102180. |
| 382 | Heckel D, Comtesse N, Brass N, Blin N, Zang KD, Meese E. Novel immunogenic antigen homologous to hyaluronidase in meningioma. Human molecular genetics. 1998;7(12):1859-72. doi: 10.1093/hmg/7.12.1859. |
| 383 | Kuo YH, Edgar MA, Luther N, Schwartz TH. Novel low-grade glioneuronal neoplasm presenting in an octogenarian: Case report and review of the literature. Clinical neurology and neurosurgery. 2006;108(4):426-32. doi: 10.1016/j.clineuro.2005.05.006. |
| 384 | Huq S, Khalafallah AM, Ruiz-Cardozo MA, Botros D, Oliveira LAP, Dux H, et al. A novel radiographic marker of sarcopenia with prognostic value in glioblastoma. Clinical neurology and neurosurgery. 2021;207. doi: 10.1016/j.clineuro.2021.106782. |
| 385 | Morone PJ, Stewart TG, Zuckermen SL, Dewan MC, Mistry A, Agazzi S, et al. A novel risk stratification tool to predict hospital length of stay after surgery for meningioma. Journal of Neurological Surgery, Part B: Skull Base. 2018;79. doi: 10.1055/s-0038-1633425. |
| 386 | Patni N, Alves C, Von Schnurbein J, Wabitsch M, Tannin G, Rakheja D, et al. A novel syndrome of generalized lipodystrophy associated with pilocytic astrocytoma. Journal of Clinical Endocrinology and Metabolism. 2015;100(10):3603-6. doi: 10.1210/jc.2015-2476. |
| 387 | Patni N, Alves C, von Schnurbein J, Wabitsch M, Tannin G, Rakheja D, et al. A novel syndrome of generalized lipodystrophy associated with pilocytic astrocytoma. 2015. p. 3603-6. |
| 388 | Lee EJ, Kim JH, Park ES, Kim YH, Lee JK, Hong SH, et al. A novel weighted scoring system for estimating the risk of rapid growth in untreated intracranial meningiomas. Journal of neurosurgery. 2017;127(5):971-80. doi: 10.3171/2016.9.JNS161669. |
| 389 | Peng J, Boekhoff S, Eveslage M, Bison B, Sowithayasakul P, Friedrich C, et al. Nuchal Skinfold Thickness in Pediatric Brain Tumor Patients. Frontiers in endocrinology. 2021;12:772856. Epub 2022/01/04. doi: 10.3389/fendo.2021.772856. |
| 390 | Peng J, Boekhoff S, Eveslage M, Bison B, Sowithayasakul P, Friedrich C, et al. Nuchal Skinfold Thickness in Pediatric Brain Tumor Patients. Frontiers in endocrinology. 2021;12. doi: 10.3389/fendo.2021.772856. |
| 391 | Runco D, Mertens A, Wasilewski-Masker K. Nutritional outcomes in pediatric oncology patients less than 3 years of age. Journal of Clinical and Translational Science. 2018:84. doi: 10.1017/cts.2018.293. |
| 392 | Dadashi-noshahr Y, Behzad A, Ahmadizadeh SN, Amirjani S, Vahdat Shariatpanahi Z. Nutritional status and clinical outcomes of brain tumor surgery in pediatric intensive care unit: A prospective cohort study. Clinical nutrition ESPEN. 2024;61:302-7. doi: 10.1016/j.clnesp.2024.03.029. |
| 393 | Prasad M, Arora B, Chinnaswamy G, Vora T, Narula G, Banavali S, et al. Nutritional status in survivors of childhood cancer: Experience from Tata Memorial Hospital, Mumbai. Indian journal of cancer. 2015;52(2):219-23. doi: 10.4103/0019-509X.175814. |
| 394 | Rhines LD, Sampath P, Dolan ME, Tyler BM, Brem H, Weingart J. O6-benzylguanine potentiates the antitumor effect of locally delivered carmustine against an intracranial rat glioma. Cancer research. 2000;60(22):6307-10. |
| 395 | Avgerinos KI, Spyrou N, Mantzoros CS, Dalamaga M. Obesity and cancer risk: Emerging biological mechanisms and perspectives. Metabolism: clinical and experimental. 2019;92:121-35. doi: 10.1016/j.metabol.2018.11.001. |
| 396 | Colditz GA, Peterson LL. Obesity and Cancer: Evidence, Impact, and Future Directions. Clinical chemistry. 2018;64(1):154-62. Epub 2017/10/19. doi: 10.1373/clinchem.2017.277376. PubMed PMID: 29038151. |
| 397 | Khazanchi R, Nandoliya KR, Shahin MN, Rae AI, Chaliparambil RK, Bowden SG, et al. Obesity and meningioma: a US population-based study paired with analysis of a multi-institutional cohort. Journal of neurosurgery. 2024;140(6):1558-67. doi: 10.3171/2023.11.JNS23732. |
| 398 | Pietilä S, Mäkipernaa A, Sievänen H, Koivisto AM, Wigren T, Lenko HL. Obesity and metabolic changes are common in young childhood brain tumor survivors. Pediatric Blood and Cancer. 2009;52(7):853-9. doi: 10.1002/pbc.21936. |
| 399 | Sergentanis TN, Tsivgoulis G, Perlepe C, Ntanasis-Stathopoulos I, Tzanninis IG, Sergentanis IN, et al. Obesity and risk for brain/CNS tumors, gliomas and meningiomas: A meta-analysis. PloS one. 2015;10(9). doi: 10.1371/journal.pone.0136974. |
| 400 | Zou Y, Pitchumoni CS. Obesity, obesities and gastrointestinal cancers. Disease-a-Month. 2023;69(12). doi: 10.1016/j.disamonth.2023.101592. |
| 401 | Vajtai I, von Gunten M, Fung C, Brekenfeld C, Kappeler A, Reinert MM. Oncocytic ependymoma: A new morphological variant of high-grade ependymal neoplasm composed of mitochondrion-rich epithelioid cells. Pathology Research and Practice. 2011;207(1):49-54. doi: 10.1016/j.prp.2010.05.006. |
| 402 | McHayle A, Pertsch NJ, Toms SA, Weil RJ. Operative duration and early outcomes in patients having a supratentorial craniotomy for brain tumor: A propensity matched analysis. Journal of Clinical Neuroscience. 2021;92:207-14. doi: 10.1016/j.jocn.2021.08.005. |
| 403 | Samples JR, Robertson DM, Taylor JZ, Waller RR. Optic nerve meningioma. Ophthalmology. 1983;90(12):1591-4. doi: 10.1016/S0161-6420(83)34346-3. |
| 404 | Rey JW, Heister P, Wirges U, Nadalin S, Breuer R, Niehues T. Organ donor with unclear primary brain tumor, a contraindication for transplantation? Case report of a one year old child. Klinische Padiatrie. 2009;221(6):390-2. doi: 10.1055/s-0029-1239528. |
| 405 | Mazzatenta D, Zoli M, Guaraldi F, Ambrosi F, Faustini Fustini M, Pasquini E, et al. Outcome of Endoscopic Endonasal Surgery in Pediatric Craniopharyngiomas. World neurosurgery. 2020;134:e277-e88. doi: 10.1016/j.wneu.2019.10.039. |
| 406 | Bekelis K, Bakhoum SF, Desai A, MacKenzie TA, Roberts DW. Outcome prediction in intracranial tumor surgery: The National Surgical Quality Improvement Program 2005-2010. Journal of neuro-oncology. 2013;113(1):57-64. doi: 10.1007/s11060-013-1089-3. |
| 407 | Soto JM, Nguyen AV, van Zyl JS, Huang JH. Outcomes After Supratentorial Craniotomy for Primary Malignant Brain Tumor Resection in Adult Patients: A National Surgical Quality Improvement Program Analysis. World neurosurgery. 2023;175:e780-e9. doi: 10.1016/j.wneu.2023.04.020. |
| 408 | Chai-Adisaksopha C, Linkins LA, Alkindi SY, Cheah M, Crowther MA, Iorio A. Outcomes of low-molecular-weight heparin treatment for venous thromboembolism in patients with primary and metastatic brain tumours. Thrombosis and haemostasis. 2017;117(3):589-94. doi: 10.1160/TH16-09-0680. |
| 409 | Chai-Adisaksopha C, Linkins LA, SY AL, Cheah M, Crowther MA, Iorio A. Outcomes of low-molecular-weight heparin treatment for venous thromboembolism in patients with primary and metastatic brain tumours. Thrombosis and haemostasis. 2017;117(3):589-94 |
| 410 | Chiasakul T, Redd R, Patell R, Khan AM, McCarthy EP, Neuberg D, et al. Overall survival with warfarin vs. low-molecular-weight heparin in cancer-associated thrombosis. Journal of Thrombosis and Haemostasis. 2021;19(11):2825-34. doi: 10.1111/jth.15519. |
| 411 | Wiedmann MKH, Brunborg C, Di Ieva A, Lindemann K, Johannesen TB, Vatten L, et al. Overweight, obesity and height as risk factors for meningioma, glioma, pituitary adenoma and nerve sheath tumor: a large population-based prospective cohort study. Acta Oncologica. 2017;56(10):1302-9. |
| 412 | Shao C, Bai LP, Qi ZY, Hui GZ, Wang Z. Overweight, obesity and meningioma risk: A meta-analysis. PloS one. 2014;9(2). doi: 10.1371/journal.pone.0090167. |
| 413 | Nagahama A, Yashiro M, Miki Y, Morisako H, Uda T, Goto T, et al. P53 expression is a useful predictive marker for recurrence of meningioma. Cancer research. 2019;79(13). doi: 10.1158/1538-7445.4901. |
| 414 | Li PC, Chen SY, Xiangfei D, Mao C, Wu CH, Shih JC. PAMs inhibits monoamine oxidase a activity and reduces glioma tumor growth, a potential adjuvant treatment for glioma. BMC complementary medicine and therapies. 2020;20(1). doi: 10.1186/s12906-020-03041-z. |
| 415 | Lee AG, Wall M. Papilledema: Are we any nearer to a consensus on pathogenesis and treatment? Current neurology and neuroscience reports. 2012;12(3):334-9. doi: 10.1007/s11910-012-0257-8. PubMed Central PMCID: Duramed(United States) |
| 416 | Smulevich VB, Solionova LG, Belyakova SV. Parental occupation and other factors and cancer risk in children: I. Study methodology and non-occupational factors. International journal of cancer. 1999;83(6):712-7. doi: 10.1002/(SICI)1097-0215(19991210)83:6<712::AID-IJC2>3.0.CO;2-D. |
| 417 | Thomsen H, Steffensen E, Larsson EM. Perfusion MRI (dynamic susceptibility contrast imaging) with different measurement approaches for the evaluation of blood flow and blood volume in human gliomas. Acta radiologica (Stockholm, Sweden : 1987). 2012;53(1):95-101. doi: 10.1258/ar.2011.110242. |
| 418 | Georgakis MK, Dessypris N, Papadakis V, Tragiannidis A, Bouka E, Hatzipantelis E, et al. Perinatal and early life risk factors for childhood brain tumors: Is instrument-assisted delivery associated with higher risk? Cancer epidemiology. 2019;59:178-84. doi: 10.1016/j.canep.2019.01.017. |
| 419 | Crump C, Sundquist J, Sieh W, Winkleby MA, Sundquist K. Perinatal and familial risk factors for brain tumors in childhood through young adulthood. Cancer research. 2015;75(3):576-83. doi: 10.1158/0008-5472.CAN-14-2285. |
| 420 | Wilhelmy F, Hantsche A, Wende T, Kasper J, Reuschel V, Frydrychowicz C, et al. Perioperative anticoagulation in patients with intracranial meningioma: No increased risk of intracranial hemorrhage? PloS one. 2020;15(9 September 2020). doi: 10.1371/journal.pone.0238387. |
| 421 | Shimony N, Amit U, Minz B, Grossman R, Dany MA, Gonen L, et al. Perioperative pregabalin for reducing pain, analgesic consumption, and anxiety and enhancing sleep quality in elective neurosurgical patients: A prospective, randomized, double-blind, and controlled clinical study. Journal of neurosurgery. 2016;125(6):1513-22. |
| 422 | Ruggieri F, Gemma M, Calvi MR, Nicelli E, Agarossi A, Beretta L. Perioperative serum brain natriuretic peptide and cardiac troponin in elective intracranial surgery. Neurocritical care. 2012;17(3):395-400. doi: 10.1007/s12028-012-9684-2. |
| 423 | Salmaggi A, Simonetti G, Trevisan E, Beecher D, Carapella CM, Dimeco F, et al. Perioperative thromboprophylaxis in patients with craniotomy for brain tumours: A systematic review. Journal of neuro-oncology. 2013;113(2):293-303. doi: 10.1007/s11060-013-1115-5. |
| 424 | Neuwelt EA, Specht HD, Hill SA. Permeability of human brain tumor to 99mTc-gluco-heptonate and 99mTc-albumin. Implications for monoclonal antibody therapy. Journal of neurosurgery. 1986;65(2):194-8. Epub 1986/08/01. doi: 10.3171/jns.1986.65.2.0194. PubMed PMID: 3723177. |
| 425 | Chang SM, Kuhn JG, Robins I, Schold SC, Spence AM, Berger MS, et al. Phase II study of phenylacetate in patients with recurrent malignant glioma: A North American brain tumor consortium report. Journal of Clinical Oncology. 1999;17(3):984-90. doi: 10.1200/jco.1999.17.3.984. |
| 426 | Affronti ML, Jackman JG, McSherry F, Herndon JE, Massey EC, Lipp E, et al. Phase II Study to Evaluate the Efficacy and Safety of Rilotumumab and Bevacizumab in Subjects with Recurrent Malignant Glioma. The oncologist. 2018;23(8):889-e98. doi: 10.1634/theoncologist.2018-0149. PubMed Central PMCID: Amgen |
| 427 | Hu J, Ljubimova JY, Inoue S, Konda B, Patil R, Ding H, et al. Phosphodiesterase type 5 inhibitors increase herceptin transport and treatment efficacy in mouse metastatic brain tumor models. PloS one. 2010;5(4). doi: 10.1371/journal.pone.0010108. PubMed Central PMCID: Genentech(United States) |
| 428 | Sharif T, Martell E, Dai C, Ghassemi-Rad MS, Lee K, Singh SK, et al. Phosphoglycerate dehydrogenase inhibition induces p-mTOR-independent autophagy and promotes multilineage differentiation in embryonal carcinoma stem-like cells. Cell Death and Disease. 2018;9(10). doi: 10.1038/s41419-018-0997-8. |
| 429 | Thawani JP, Amirshaghaghi A, Yan L, Stein JM, Liu J, Tsourkas A. Photoacoustic-Guided Surgery with Indocyanine Green-Coated Superparamagnetic Iron Oxide Nanoparticle Clusters. Small (Weinheim an der Bergstrasse, Germany). 2017;13(37). doi: 10.1002/smll.201701300. |
| 430 | Lewandowski M, Gwoździński K. Photoprotective and radioprotective properties of nitroxides and their application in magnetic resonance imaging. Postepy higieny i medycyny doswiadczalnej (Online). 2016;70:1101-11. |
| 431 | Friedenreich CM, Ryder-Burbidge C, McNeil J. Physical activity, obesity and sedentary behavior in cancer etiology: epidemiologic evidence and biologic mechanisms. Molecular oncology. 2021;15(3):790-800. doi: 10.1002/1878-0261.12772. |
| 432 | Portnow J, Badie B, Liu X, Frankel P, Mi S, Chen M, et al. A pilot microdialysis study in brain tumor patients to assess changes in intracerebral cytokine levels after craniotomy and in response to treatment with a targeted anti-cancer agent. Journal of neuro-oncology. 2014;118(1):169-77. doi: 10.1007/s11060-014-1415-4. |
| 433 | Howell JC, Rose SR. Pituitary disease in pediatric brain tumor survivors. Expert Review of Endocrinology and Metabolism. 2019;14(4):283-91. doi: 10.1080/17446651.2019.1620599. |
| 434 | Grzywotz A, Li Y, Unger N, Kiewert C, Chmielewski WX, Sure U, et al. Pituitary enlargement in patients with cerebrospinal fluid drainage due to ventricular shunt insertion: know the condition and do not mistake for adenoma. Pituitary. 2023;26(1):164-70. doi: 10.1007/s11102-022-01296-y. |
| 435 | Komarowska M, Chrzanowski R, Tylicka M, Rutkowski R, Mariak Z, Zelazowska-Rutkowska B, et al. Plasma concentration of Bisphenol A and leptin in patients with meningioma and glioma: A pilot study. Advances in medical sciences. 2022;67(2):229-33. doi: 10.1016/j.advms.2022.04.002. |
| 436 | Sawaya R, Highsmith R. Plasminogen activator activity and molecular weight patterns in human brain tumors. Journal of neurosurgery. 1988;68(1):73-9. doi: 10.3171/jns.1988.68.1.0073. |
| 437 | Lapierre V, Mahé C, Aupérin A, Stambouli F, Oubouzar N, Tramalloni D, et al. Platelet transfusion containing ABO-incompatible plasma and hepatic veno-occlusive disease after hematopoietic transplantation in young children. Transplantation. 2005;80(3):314-9. doi: 10.1097/01.tp.0000167758.63247.f4. |
| 438 | Redgate ES, Boggs S, Grudziak A, Deutsch M. Polyamines in brain tumor therapy. Journal of neuro-oncology. 1995;25(2):167-79. doi: 10.1007/BF01057761. |
| 439 | Mandrell B, Crabtree V, Smith M, Wise M, West N, Indelicato D, et al. Polysomnographyand multiple sleep latency test findings in children with craniopharyngioma prior to proton therapy. Pediatric Blood and Cancer. 2014;61:S240-S1. doi: 10.1002/pbc.25314. |
| 440 | Han K, Peyret T, Marchand M, Quartino A, Gosselin NH, Girish S, et al. Population pharmacokinetics of bevacizumab in cancer patients with external validation. Cancer chemotherapy and pharmacology. 2016;78(2):341-51. doi: 10.1007/s00280-016-3079-6. |
| 441 | Prisco D, Tufano A, Cenci C, Pignatelli P, Santilli F, Di Minno G, et al. Position paper of the Italian Society of Internal Medicine (SIMI) on prophylaxis and treatment of venous thromboembolism in patients with cancer. Internal and emergency medicine. 2019;14(1):21-38. doi: 10.1007/s11739-018-1956-1. |
| 442 | Nishiguchi T, Iwakiri T, Hayasaki K, Ohsawa M, Yoneda T, Mitsuhashi Y, et al. Post-embolisation susceptibility changes in giant meningiomas: Multiparametric histogram analysis using non-contrast-enhanced susceptibility-weighted PRESTO, diffusion-weighted and perfusion-weighted imaging. European radiology. 2013;23(2):551-61. |
| 443 | Mott JD, Thomas CL, Rosenbach MT, Takahara K, Greenspan DS, Banda MJ. Post-translational proteolytic processing of procollagen C-terminal proteinase enhancer releases a metalloproteinase inhibitor. Journal of Biological Chemistry. 2000;275(2):1384-90. doi: 10.1074/jbc.275.2.1384. |
| 444 | Behari S, Tyagi I, Banerji D, Kumar V, Jaiswal AK, Phadke RV, et al. Postauricular, transpetrous, presigmoid approach for extensive skull base tumors in the petroclival region: The successes and the travails. Acta neurochirurgica. 2010;152(10):1633-45. doi: 10.1007/s00701-010-0701-y. |
| 445 | Nguyen MP, Morshed RA, Cheung SW, Theodosopoulos PV, McDermott MW. Postoperative Complications and Neurological Deficits after Petroclival Region Meningioma Resection: A Case Series. Operative Neurosurgery. 2023;25(3):251-9. doi: 10.1227/ons.0000000000000791. |
| 446 | Castro-Ribeiro ML, Castro VIB, Vieira de Castro J, Pires RA, Reis RL, Costa BM, et al. The Potential of the Fibronectin Inhibitor Arg-Gly-Asp-Ser in the Development of Therapies for Glioblastoma. International journal of molecular sciences. 2024;25(9). doi: 10.3390/ijms25094910. |
| 447 | Miccoli L, Poirson-Bichat F, Sureau F, Bras Gonçalves R, Bourgeois Y, Dutrillaux B, et al. Potentiation of lonidamine and diazepam, two agents acting on mitochondria, in human glioblastoma treatment. Journal of the National Cancer Institute. 1998;90(18):1400-6. |
| 448 | Ranjan S, Leung D, Ghiaseddin AP, Taylor JW, Lobbous M, Dhawan A, et al. Practical guidance for direct oral anticoagulant use in the treatment of venous thromboembolism in primary and metastatic brain tumor patients. Cancer. 2024;130(9):1577-89. doi: 10.1002/cncr.35220. |
| 449 | Anttonen J, Remes T, Arikoski P, Lähteenmäki P, Arola M, Harila-Saari A, et al. Pre- And postdiagnosis growth failure, adult short stature, and untreated growth hormone deficiency in radiotherapy-treated long-term survivors of childhood brain tumor. PloS one. 2022;17(9 September). doi: 10.1371/journal.pone.0274274. |
| 450 | Helseth A, Tretli S. Pre-morbid height and weight as risk factors for development of central nervous system neoplasms. Neuroepidemiology. 1989;8(6):277-82. doi: 10.1159/000110195. |
| 451 | Zhang X, Lv H, Zhou Q, Elkholi R, Chipuk JE, Reddy MV, et al. Preclinical pharmacological evaluation of a novel multiple kinase inhibitor, ON123300, in brain tumor models. Molecular cancer therapeutics. 2014;13(5):1105-16.. |
| 452 | Rumshisky A, Ghassemi M, Naumann T, Szolovits P, Castro VM, McCoy TH, et al. Predicting early psychiatric readmission with natural language processing of narrative discharge summaries. Translational psychiatry. 2016;6(10). doi: 10.1038/TP.2015.182. |
| 453 | Muhlestein WE, Akagi DS, Davies JM, Chambless LB. Predicting Inpatient Length of Stay after Brain Tumor Surgery: Developing Machine Learning Ensembles to Improve Predictive Performance. Clinical neurosurgery. 2019;85(3):384-93. doi: 10.1093/neuros/nyy343. |
| 454 | Chen J, Xue Y, Ren L, Lv K, Du P, Cheng H, et al. Predicting meningioma grades and pathologic marker expression via deep learning. European radiology. 2024;34(5):2997-3008. doi: 10.1007/s00330-023-10258-2. |
| 455 | Hua C, Wu S, Chemaitilly W, Lukose RC, Merchant TE. Predicting the probability of abnormal stimulated growth hormone response in children after radiotherapy for brain tumors. International Journal of Radiation Oncology Biology Physics. 2012;84(4):990-5. doi: 10.1016/j.ijrobp.2012.01.049. |
| 456 | Tohidinezhad F, Zegers CML, Vaassen F, Dijkstra J, Anten M, Van Elmpt W, et al. Predicting the risk of neurocognitive decline after brain irradiation in adult patients with a primary brain tumor. Neuro-oncology. 2024;26(8):1467-78. doi: 10.1093/neuonc/noae035. |
| 457 | Gorenstein L, Shrot S, Ben-Ami M, Stern E, Yalon M, Hoffmann C, et al. Predictive factors for radiation-induced pituitary damage in pediatric patients with brain tumors. Radiotherapy and Oncology. 2024;196. doi: 10.1016/j.radonc.2024.110268. |
| 458 | Truong TH, Prokopishyn NL, Luu H, Guilcher GMT, Lewis VA. Predictive factors for successful peripheral blood stem cell mobilization and collection in children. Journal of clinical apheresis. 2019;34(5):598-606. doi: 10.1002/jca.21738. |
| 459 | Baucher G, Troude L, Al-Shabibi T, Avinens V, Fernandes S, Roche PH. Predictive factors of the postoperative proptosis recovery in surgery of spheno-orbital meningiomas. Acta neurochirurgica. 2024;166(1). doi: 10.1007/s00701-024-06053-7. |
| 460 | Naderi N, Moriyama D, Lin J, Sazgar M, Sen-Gupta I, Mnatsakanyan L. Predictors of in-hospital mortality in status epilepticus; Data from the nationwide inpatient sample database, 2008-2012. Epilepsy Currents. 2015;15:369. |
| 461 | Schneider B, Pülhorn H, Röhrig B, Rainov NG. Predisposing conditions and risk factors for development of symptomatic meningioma in adults. Cancer detection and prevention. 2005;29(5):440-7. doi: 10.1016/j.cdp.2005.07.002. |
| 462 | Mueller BA, Chow EJ, Kamineni A, Daling JR, Fraser A, Wiggins CL, et al. Pregnancy outcomes in female childhood and adolescent cancer survivors: A linked cancer-birth registry analysis. Archives of Pediatrics and Adolescent Medicine. 2009;163(10):879-86. doi: 10.1001/archpediatrics.2009.112. |
| 463 | Kim JH, Yoon HK, Lee HC, Park HP, Park CK, Dho YS, et al. Preoperative 5-aminolevulinic acid administration for brain tumor surgery is associated with an increase in postoperative liver enzymes: a retrospective cohort study. Acta neurochirurgica. 2019;161(11):2289-98. |
| 464 | Shi S, Cheng J, Chen H, Zhang Y, Zhao Y, Wang B. Preoperative and intraoperative predictors of deep venous thrombosis in adult patients undergoing craniotomy for brain tumors: A Chinese single-center, retrospective study. Thrombosis research. 2020;196:245-50. doi: 10.1016/j.thromres.2020.09.005. |
| 465 | Thommen R, Kazim SF, Rumalla K, Kassicieh AJ, Kalakoti P, Schmidt MH, et al. Preoperative frailty measured by risk analysis index predicts complications and poor discharge outcomes after Brain Tumor Resection in a large multi-center analysis. Journal of neuro-oncology. 2022;160(2):285-97. |
| 466 | Jeong YI, Song JG, Kang SS, Ryu HH, Lee YH, Choi C, et al. Preparation of poly(DL-lactide-co-glycolide) microspheres encapsulating all-trans retinoic acid. International journal of pharmaceutics. 2003;259(1-2):79-91. doi: 10.1016/S0378-5173(03)00207-2. |
| 467 | Lefranc F, Rynkowski M, DeWitte O, Kiss R. Present and potential future adjuvant issues in high-grade astrocytic glioma treatment. Advances and technical standards in neurosurgery. 2009;34:3-35. |
| 468 | Cutura N, Soldo V. [Preterm delivery in a patient with frontal lobe brain tumor]. Vojnosanitetski pregled. 2009;66(10):830-2. Epub 2009/11/27. doi: 10.2298/vsp0910830c. PubMed PMID: 19938763. |
| 469 | Lebbink CA, Ringers TP, Schouten-van Meeteren AYN, van Iersel L, Clement SC, Boot AM, et al. Prevalence and risk factors of hypothalamic-pituitary dysfunction in infant and toddler childhood brain tumor survivors. European journal of endocrinology. 2021;185(4):597-606. doi: 10.1530/EJE-21-0137. |
| 470 | Rowe L, Vera E, Acquaye A, Crandon S, Shah V, Bryla C, et al. The prevalence of altered body image in patients with primary brain tumors: an understudied population. Journal of neuro-oncology. 2020;147(2):397-404. doi: 10.1007/s11060-020-03433-8. |
| 471 | Pluimakers VG, van Atteveld JE, de Winter DTC, Bolier M, Fiocco M, Nievelstein R, et al. Prevalence, risk factors, and optimal way to determine overweight, obesity, and morbid obesity in the first Dutch cohort of 2338 long-term survivors of childhood cancer: a DCCSS-LATER study. European journal of endocrinology. 2023;189(5):495-507. |
| 472 | Mumoli N, Barco S, Cei M, Giorgi-Pierfranceschi M, Campanini M, Fontanella A, et al. Prevention and treatment of venous thromboembolism in patients with solid brain neoplasms: results of a survey among Italian physicians. Internal and emergency medicine. 2017;12(4):437-43. |
| 473 | Rozumenko VD. Prevention of postoperative venous thromboembolism in patient with brain tumor. Klinichna khirurhiia / Ministerstvo okhorony zdorov'ia Ukraïny, Naukove tovarystvo khirurhiv Ukraïny. 2003(8):50-3. |
| 474 | Hu MX, Liu JL, Chen XB, Xu AQ, Shu SR, Wang CH, et al. [Primary culture of human malignant meningioma cells and its intracranial orthotopic transplantation in nude mice]. Nan fang yi ke da xue xue bao = Journal of Southern Medical University. 2018;38(3):340-5. |
| 475 | Li XL, Ren J, Niu RN, Jiang X, Xu GH, Zhou P, et al. Primary intracranial leiomyosarcoma in an immunocompetent patient: Case report with emphasis on imaging features. Medicine (United States). 2019;98(17). doi: 10.1097/MD.0000000000015269. |
| 476 | Marinelli JP, Modzeski MC, Lane JI, Van Gompel JJ, Stokken JK, Thanarajasingam G, et al. Primary Skull Base Lymphoma: Manifestations and Clinical Outcomes of a Great Imitator. Otolaryngology - Head and Neck Surgery (United States). 2018;159(4):643-9. doi: 10.1177/0194599818773994. |
| 477 | Chotai NC, Tang PH, Gan BK, Lim CC. Primitive neuroectodermal tumour metastases mimicking neurofibromatosis type 2. Singapore medical journal. 2010;51(6):e98-102. Epub 2010/07/27. PubMed PMID: 20658100. |
| 478 | Griend JPV, Linnebur SA, Bainbridge JL. Probable levetiracetam-associated depression in the elderly: Two case reports. American Journal Geriatric Pharmacotherapy. 2009;7(5):281-4. doi: 10.1016/j.amjopharm.2009.10.002. |
| 479 | Oettel M, Mukhopadhyay AK. Progesterone: The forgotten hormone in men? The aging male : the official journal of the International Society for the Study of the Aging Male. 2004;7(3):236-57. doi: 10.1080/13685530400004199. |
| 480 | AlKhoshi AM, AlZahrani AA, Shawli FS, AlJabri AA, AlAnsari AH, Alshuqayfi K, et al. Prognostic Factors Affecting Postsurgical Outcomes of Adult Patients with Intracranial Meningioma: A Retrospective Study. World neurosurgery. 2023;180:e281-e7. doi: 10.1016/j.wneu.2023.09.055. |
| 481 | Li B, Gao B, Zhu HJ, Luwor RB, Lu J, Zhang L, et al. The Prognostic Value of Preoperative Inflammatory Markers for Pathological Grading of Glioma Patients. Technology in Cancer Research and Treatment. 2024;23. doi: 10.1177/15330338241273160. |
| 482 | Hahn BM, Schrell UMH, Sauer R, Fahlbusch R, Ganslandt O, Grabenbauer GG. Prolonged oral hydroxyurea and concurrent 3d-conformal radiation in patients with progressive or recurrent meningioma: Results of a pilot study. Journal of neuro-oncology. 2005;74(2):157-65. |
| 483 | Brandt B, Németh M, Berta G, Szünstein M, Heffer M, Rauch TA, et al. A Promising Way to Overcome Temozolomide Resistance through Inhibition of Protein Neddylation in Glioblastoma Cell Lines. International journal of molecular sciences. 2023;24(9). doi: 10.3390/ijms24097929. |
| 484 | van de Vijfeijken S, Münker T, de Jager N, Vandertop WP, Becking AG, Kleverlaan CJ. Properties of an In Vivo Fractured Poly(Methyl Methacrylate) Cranioplasty After 15 Years. World neurosurgery. 2019;123:e60-e8. Epub 2018/11/18. doi: 10.1016/j.wneu.2018.11.026. PubMed PMID: 30447447. |
| 485 | Moussa WM, Mohamed MA. Prophylactic use of anticoagulation and hemodilution for the prevention of venous thromboembolic events following meningioma surgery. Clinical neurology and neurosurgery. 2016;144:1-6. Epub 2016/03/08. doi: 10.1016/j.clineuro.2016.02.040. PubMed PMID: 26945875. |
| 486 | Hamilton MG, Hull RD, Pinco GF. Prophylaxis of venous thromboembolism in brain tumor patients. Journal of neuro-oncology. 1994;22(2):111-26. doi: 10.1007/BF01052887. |
| 487 | Saha BN, Ray N, Greiner R, Murtha A, Zhang H. Quick detection of brain tumors and edemas: A bounding box method using symmetry. Computerized Medical Imaging and Graphics. 2012;36(2):95-107. doi: 10.1016/j.compmedimag.2011.06.001. |
| 488 | Kuon E, Birkel J, Schmitt M, Dahm JB. Radiation exposure benefit of a lead cap in invasive cardiology. Heart (British Cardiac Society). 2003;89(10):1205-10. doi: 10.1136/heart.89.10.1205. |
| 489 | Pedachenko EG, Velibekov RA. Radiation-induced intracranial meningiomas. Likars'ka sprava / Ministerstvo okhorony zdorov'ia Ukraïny. 1998(4):118-20. |
| 490 | Barth RF, Yang W, Coderre JA. Rat brain tumor models to assess the efficacy of boron neutron capture therapy: A critical evaluation. Journal of neuro-oncology. 2003;62(1):61-74. doi: 10.1023/A:1023239021135. |
| 491 | Bellavance MA, Blanchette M, Fortin D. Recent advances in blood-brain barrier disruption as a CNS delivery strategy. AAPS Journal. 2008;10(1):166-77. doi: 10.1208/s12248-008-9018-7. |
| 492 | Young G, Toretsky JA, Campbell AB, Eskenazi AE. Recognition of common childhood malignancies. American family physician. 2000;61(7):2144-54. |
| 493 | Eisenring CV, Neidert MC, Sabanés Bové D, Held L, Sarnthein J, Krayenbühl N. Reduction of thromboembolic events in meningioma surgery: a cohort study of 724 consecutive patients. PloS one. 2013;8(11):e79170. |
| 494 | Ebrahimi Zade A, Shahabi Haghighi S, Soltani M. Reinforcement learning for optimal scheduling of Glioblastoma treatment with Temozolomide. Computer methods and programs in biomedicine. 2020;193. doi: 10.1016/j.cmpb.2020.105443. |
| 495 | Shahrestani MA, Saneei P, Shayanfar M, Mohammad-Shirazi M, Sharifi G, Sadeghi O, et al. The relationship between rice consumption and glioma: a case-control study in adults. Scientific reports. 2021;11(1):6073. doi: 10.1038/s41598-021-85562-2. |
| 496 | Duan B, Hu X, Zhao H, Qin J, Luo J. The relationship between urinary bisphenol A levels and meningioma in Chinese adults. International journal of clinical oncology. 2013;18(3):492-7. doi: 10.1007/s10147-012-0408-6. |
| 497 | Sarkar S, Throckmorton W, Bingham R, Msaouel P, Genovese G, Slopis J, et al. Renal Cell Carcinoma Unclassified with Medullary Phenotype in a Patient with Neurofibromatosis Type 2. Current Oncology. 2023;30(3):3355-65. doi: 10.3390/curroncol30030255. |
| 498 | Kurita N, Kawaguchi M, Nakahashi K, Sakamoto N, Horiuchi T, Takahashi M, et al. Retrospective Analysis of Postoperative Nausea and Vomiting after Craniotomy. Japanese Journal of Anesthesiology. 2004;53(2):150-5. |
| 499 | De Tommasi C, Cusimano MD. Rhabdomyolysis after neurosurgery: A review and a framework for prevention. Neurosurgical review. 2013;36(2):195-203. doi: 10.1007/s10143-012-0423-0. |
| 500 | Khan R, Merchant T, Sadighi Z, Crabtree V, Bello M, Sykes A, et al. Risk factors for hypersomnia/narcolepsy and response to therapy in survivors of childhood brain tumors. Neurology. 2017;88(16). |
| 501 | Wang C, Li P. Risk factors for intraoperative blood loss in resection of intracranial meningioma: Analysis of 530 cases. PloS one. 2023;18(9 September). doi: 10.1371/journal.pone.0291171. |
| 502 | Johnson DR, Olson JE, Vierkant RA, Hammack JE, Wang AH, Folsom AR, et al. Risk factors for meningioma in post-menopausal women: Results from the iowa women's health study. Neuro-oncology. 2010;12:iv27. doi: 10.1093/neuonc/noq116. |
| 503 | Johnson DR, Olson JE, Vierkant RA, Hammack JE, Wang AH, Folsom AR, et al. Risk factors for meningioma in postmenopausal women: Results from the Iowa Women's Health Study. Neuro-oncology. 2011;13(9):1011-9. doi: 10.1093/neuonc/nor081. |
| 504 | Delacourt L, Allodji R, Chappat J, Haddy N, El-Fayech C, Demoor-Goldschmidt C, et al. Risk factors for obesity in adulthood among survivors of childhood cancer. Obesity. 2023;31(7):1942-52. doi: 10.1002/oby.23784. |
| 505 | Ivan ME, Iorgulescu JB, El-Sayed I, McDermott MW, Parsa AT, Pletcher SD, et al. Risk factors for postoperative cerebrospinal fluid leak and meningitis after expanded endoscopic endonasal surgery. Journal of clinical neuroscience : official journal of the Neurosurgical Society of Australasia. 2015;22(1):48-54. |
| 506 | Gallus S, Foschi R, Talamini R, Altieri A, Negri E, Franceschi S, et al. Risk Factors for Prostate Cancer in Men Aged Less Than 60 Years: A Case-Control Study from Italy. Urology. 2007;70(6):1121-6. doi: 10.1016/j.urology.2007.07.020. |
| 507 | Lustig RH, Post SR, Srivannaboon K, Rose SR, Danish RK, Burghen GA, et al. Risk factors for the development of obesity in children surviving brain tumors. Journal of Clinical Endocrinology and Metabolism. 2003;88(2):611-6. doi: 10.1210/jc.2002-021180. |
| 508 | Zhao X, Wang L, Wei N, Zhang J, Ma W, Zhao H, et al. Risk factors of health care-associated infection in elderly patients: a retrospective cohort study performed at a tertiary hospital in China. BMC geriatrics. 2019;19(1):193. doi: 10.1186/s12877-019-1208-x. |
| 509 | Sangtongjaraskul S, Sae-Phua V, Amornfa J, Tuchinda L. Risk factors of intraoperative blood transfusion in pediatric craniotomy for intracranial tumor resection: a 10-year analysis. Journal of Neurosurgery: Pediatrics. 2023;32(1):115-23. doi: 10.3171/2023.2.PEDS22535. |
| 510 | Moseeva MB, Azizova TV, Bannikova MV. Risk of central nervous system tumour incidence in a cohort of workers chronically exposed to ionising radiation. Radiation and environmental biophysics. 2024;63(1):17-26. doi: 10.1007/s00411-023-01054-z. |
| 511 | Yang J, He Z, Li M, Hong T, Ouyang T. Risk of intracranial hemorrhage with direct oral anticoagulation versus low molecular weight heparin in the treatment of brain tumor-associated venous thromboembolism: A meta-analysis. Journal of Stroke and Cerebrovascular Diseases. 2023;32(8). doi: 10.1016/j.jstrokecerebrovasdis.2023.107243. |
| 512 | Hartnett KP, Ward KC, Kramer MR, Lash TL, Mertens AC, Spencer JB, et al. The risk of preterm birth and growth restriction in pregnancy after cancer. International journal of cancer. 2017;141(11):2187-96. doi: 10.1002/ijc.30914. |
| 513 | Heuch JM, Heuch I, Akslen LA, Kvåle G. Risk of primary childhood brain tumors related to birth characteristics: A Norwegian prospective study. International journal of cancer. 1998;77(4):498-503. doi: 10.1002/(SICI)1097-0215(19980812)77:4<498::AID-IJC4>3.0.CO;2-P. |
| 514 | Costanzo R, Simonetta I, Musso S, Benigno UE, Cusimano LM, Giovannini EA, et al. Role of Mediterranean diet in the development and recurrence of meningiomas: a narrative review. Neurosurgical review. 2023;46(1). doi: 10.1007/s10143-023-02128-8. |
| 515 | Abdolmohammadi J, Faeghi F, Arefan D, Zali A, Haghighatkhah H, Amiri J. The Role of Single Voxel MR Spectroscopy, T2 Relaxation Time and Apparent Diffusion Coefficient in Determining the Cellularity of Brain Tumors by MATLAB Software. Asian Pacific journal of cancer prevention : APJCP. 2018;19(10):2891-5. |
| 516 | Sriram R, Ali-Osman F. S1-nuclease enhancement of the ethidium bromide binding assay of drug-induced DNA interstrand crosslinking in human brain tumor cells. Analytical biochemistry. 1990;187(2):345-8. Epub 1990/06/01. doi: 10.1016/0003-2697(90)90467-n. PubMed PMID: 2200310. |
| 517 | Rao MG, Ladner TR, Shuman WH, Feng R, Fifi JT, De Leacy RA, et al. Safety and efficacy of preoperative embolization of meningioma. Journal of Neurological Surgery Part B: Skull Base. 2021;82(SUPPL 2). doi: 10.1055/s-0041-1725344. |
| 518 | Abuzzahab M, Scimia C, Lekaditis S, Machus B, Kearns S, Roth CL. Safety and Efficacy of Setmelanotide in Patients With Hypothalamic Obesity: Phase 2 Trial Design. Obesity facts. 2022;15:273. doi: 10.1159/000524649. |
| 519 | Constantini S, Kanner A, Friedman A, Shoshan Y, Israel Z, Ashkenazi E, et al. Safety of perioperative minidose heparin in patients undergoing brain tumor surgery: A prospective, randomized, double-blind study. Journal of neurosurgery. 2001;94(6):918-21. doi: 10.3171/jns.2001.94.6.0918. |
| 520 | Marcus CL, Trescher WH, Halbower AC, Lutz J. Secondary narcolepsy in children with brain tumors. Sleep. 2002;25(4):435-9. |
| 521 | Todo T, Adams EF, Rafferty B, Fahlbusch R, Dingermann T, Werner H. Secretion of interleukin-6 by human meningioma cells: Possible autocrine inhibitory regulation of neoplastic cell growth. Journal of neurosurgery. 1994;81(3):394-401. doi: 10.3171/jns.1994.81.3.0394. |
| 522 | Jhawar BS, Fuchs CS, Colditz GA, Stampfer MJ. Sex steroid hormone exposures and risk for meningioma. Journal of neurosurgery. 2003;99(5):848-53. doi: 10.3171/jns.2003.99.5.0848. |
| 523 | Bjørnerud A, Vatnehol SAS, Larsson C, Due-Tønnessen P, Hol PK, Groote IR. Signal enhancement of the dentate nucleus at unenhanced MR imaging after very high cumulative doses of the macrocyclic gadolinium-based contrast agent gadobutrol: An observational study. Radiology. 2017;285(2):434-44. |
| 524 | Lin KW, Liao A, Qutub AA. Simulation predicts IGFBP2-HIF1α interaction drives glioblastoma growth. PLoS computational biology. 2015;11(4). doi: 10.1371/journal.pcbi.1004169. |
| 525 | Califaretti E, Di Gregorio S, Zotta M, Passera R, Lesca A, Pellerino A, et al. A single center 18F-DOPA PET/CT experience in patients with primary brain tumors after treatment: preliminary results. Clinical and Translational Imaging. 2022;10(SUPPL 1):S47-S8. doi: 10.1007/s40336-022-00492-x. |
| 526 | Brimeyer C, Adams L, Zhu L, Srivastava DK, Wise M, Hudson MM, et al. Sleep complaints in survivors of pediatric brain tumors. Supportive Care in Cancer. 2016;24(1):23-31. doi: 10.1007/s00520-015-2713-x. |
| 527 | Nolan VG, Gapstur R, Gross CR, Desain LA, Neglia JP, Gajjar A, et al. Sleep disturbances in adult survivors of childhood brain tumors. Quality of life research : an international journal of quality of life aspects of treatment, care and rehabilitation. 2013;22(4):781-9. doi: 10.1007/s11136-012-0208-5. |
| 528 | Terada T. Small cell carcinoma of the brain without extracranial involvement by serial CT, MRI and PET. International journal of clinical and experimental pathology. 2010;3(3):323-7. |
| 529 | Markowitz D, Aamodt WW, Hamedani AG. Social Determinants of Health in Idiopathic Intracranial Hypertension. Journal of Neuro-Ophthalmology. 2024;44(3):346-9. doi: 10.1097/WNO.0000000000002073. |
| 530 | Pedro MT, Eissler A, Scheuerle A, Schmidberger J, Kratzer W, Wirtz CR, et al. Sodium Fluorescein as Intraoperative Visualization Tool During Peripheral Nerve Biopsies. World neurosurgery. 2020;133:e513-e21. doi: 10.1016/j.wneu.2019.09.081. |
| 531 | Chornaya V, Lyannaya O. Some physicochemical properties of cathepsin H from human meningioma. Experimental oncology. 2004;26(4):278-81. |
| 532 | Chornaya V, Lyannaya O. Some physicochemical properties of cathepsin H from human meningioma. Eksperimentalnaya Onkologiya. 2004;26(4):278-81. |
| 533 | Shahi MH, Rey JA, Castresana JS. The sonic hedgehog-GLI1 signaling pathway in brain tumor development. Expert opinion on therapeutic targets. 2012;16(12):1227-38. doi: 10.1517/14728222.2012.720975. |
| 534 | Prah MA, Al-Gizawiy MM, Mueller WM, Cochran EJ, Hoffmann RG, Connelly JM, et al. Spatial discrimination of glioblastoma and treatment effect with histologically-validated perfusion and diffusion magnetic resonance imaging metrics. Journal of neuro-oncology. 2018;136(1):13-21. |
| 535 | Preston-Martin S, Monroe K, Lee PJ, Bernstein L, Kelsey J, Henderson S, et al. Spinal meningiomas in women in Los Angeles county: Investigation of an etiological hypothesis. Cancer Epidemiology Biomarkers and Prevention. 1995;4(4):333-9. |
| 536 | Frati A, Pesce A, Toccaceli G, Fraschetti F, Caruso R, Raco A. Spinal Meningiomas Prognostic Evaluation Score (SPES): predicting the neurological outcomes in spinal meningioma surgery. Neurosurgical review. 2019;42(1):115-25. doi: 10.1007/s10143-018-0961-1. |
| 537 | Gans MS, Byrne SF, Glaser JS. Standardized A-scan echography in optic nerve disease. Archives of Ophthalmology. 1987;105(9):1232-6. |
| 538 | Komolafe MA, Sunmonu TA, Oke O. Stroke-like syndrome in a middle aged Nigerian woman with metastatic brain cancer. West African journal of medicine. 2009;28(4):266-9. |
| 539 | Tzeng SY, Green JJ. Subtle Changes to Polymer Structure and Degradation Mechanism Enable Highly Effective Nanoparticles for siRNA and DNA Delivery to Human Brain Cancer. Advanced healthcare materials. 2013;2(3):468-80. doi: 10.1002/adhm.201200257. |
| 540 | Goto T, Tanioka Y, Sakai T, Matsumoto I, Kakinoki K, Tanaka T, et al. Successful islet transplantation from a single pancreas harvested from a young, low-BMI, non-heart-beating cadaver. Transplantation proceedings. 2005;37(8):3430-2. doi: 10.1016/j.transproceed.2005.09.041. |
| 541 | Liao YM, Yeh CJ, Shu HL, Lin PC, Chang TT, Chiou SS. Successful large-volume leukapheresis for hematopoietic stem cell collection in a very-low-weight brain tumor infant with coagulopathy. Pediatrics and neonatology. 2013;54(3):211-3. doi: 10.1016/j.pedneo.2013.03.007. |
| 542 | Ifteni P, Correll CU, Burtea V, Kane JM, Manu P. Sudden unexpected death in schizophrenia: Autopsy findings in psychiatric inpatients. Schizophrenia research. 2014;155(1-3):72-6. doi: 10.1016/j.schres.2014.03.011. |
| 543 | Cao J, Sun P, Gu C, Wang H, Qu Y, Zhang H, et al. Surgical Implications and Radiologic Classification for the Upward Bulging of the Planum Sphenoidale in Patients With Anterior Skull Base Meningiomas Involving the Tuberculum Sellae Area. The Journal of craniofacial surgery. 2023;34(2):467-70. |
| 544 | Wertz D, Boveroux P, Péters P, Lenelle J, Franssen C. Surgical resection of a sphenoid wing meningioma in a patient with Glanzmann thrombasthenia. Acta anaesthesiologica Belgica. 2011;62(2):83-6. |
| 545 | Craven M, Crowley JH, Chiang L, Kline C, Malbari F, Hocking MC, et al. A Survey of Patient-Relevant Outcomes in Pediatric Craniopharyngioma: Focus on Hypothalamic Obesity. Frontiers in endocrinology. 2022;13. doi: 10.3389/fendo.2022.876770. |
| 546 | Khan AM, Chiasakul T, Redd R, Patell R, McCarthy EP, Neuberg D, et al. Survival outcomes with warfarin compared with direct oral anticoagulants in cancer-associated venous thromboembolism in the United States: A population-based cohort study. PLoS medicine. 2022;19(5). doi: 10.1371/journal.pmed.1004012. |
| 547 | Forsell P, Hellers G. The Swedish adjustable gastric banding (SAGB) for morbid obesity: 9 year experience and a 4-year follow-up of patients operated with a new adjustable band. Obesity surgery. 1997;7(4):345-51. doi: 10.1381/096089297765555601. |
| 548 | Macartney G, Stacey D, Harrison MB, VanDenKerkhof E. Symptoms, coping, and quality of life in pediatric brain tumor survivors: a qualitative study. Oncology nursing forum. 2014;41(4):390-8. doi: 10.1188/14.ONF.390-398. |
| 549 | Algattas H, Damania D, DeAndrea-Lazarus I, Kimmell KT, Marko NF, Walter KA, et al. Systematic review of safety and cost-effectiveness of venous thromboembolism prophylaxis strategies in patients undergoing craniotomy for brain tumor. Neurosurgery. 2018;82(2):142-54. doi: 10.1093/neuros/nyx156. |
| 550 | Algattas H, Damania D, Deandrea-Lazarus I, Kimmell KT, Marko NF, Walter KA, et al. Systematic Review of Safety and Cost-Effectiveness of Venous Thromboembolism Prophylaxis Strategies in Patients Undergoing Craniotomy for Brain Tumor. Clinical neurosurgery. 2018;82(2):142-54. |
| 551 | Sharif T, Dai C, Martell E, Ghassemi-Rad MS, Hanes MR, Murphy PJ, et al. TAP73 modifies metabolism and positively regulates growth of cancer stem–like cells in a redox-sensitive manner. Clinical Cancer Research. 2019;25(6):2001-17. doi: 10.1158/1078-0432.CCR-17-3177. |
| 552 | Guo P, Ma J, Li S, Guo Z, Adams AL, Gallo JM. Targeted delivery of a peripheral benzodiazepine receptor ligand-gemcitabine conjugate to brain tumors in a xenograft model. Cancer chemotherapy and pharmacology. 2001;48(2):169-76. doi: 10.1007/s002800100284. |
| 553 | Sathornsumetee S, Reardon DA. Targeting multiple kinases in glioblastoma multiforme. Expert opinion on investigational drugs. 2009;18(3):277-92. doi: 10.1517/13543780802692603. PubMed Central PMCID: Adnexus Therapesutics(United States) |
| 554 | Kong Y, Ai C, Dong F, Xia X, Zhao X, Yang C, et al. Targeting of BMI-1 with PTC-209 inhibits glioblastoma development. Cell cycle (Georgetown, Tex). 2018;17(10):1199-211. doi: 10.1080/15384101.2018.1469872. PubMed Central PMCID: Selleck. |
| 555 | Fourniols T, Randolph LD, Staub A, Vanvarenberg K, Leprince JG, Préat V, et al. Temozolomide-loaded photopolymerizable PEG-DMA-based hydrogel for the treatment of glioblastoma. Journal of Controlled Release. 2015;210:95-104. doi: 10.1016/j.jconrel.2015.05.272. |
| 556 | Senders JT, Muskens IS, Cote DJ, Goldhaber NH, Dawood HY, Gormley WB, et al. Thirty-day outcomes after craniotomy for primary malignant brain tumors: A national surgical quality improvement program analysis. Clinical neurosurgery. 2018;83(6):1249-58. doi: 10.1093/neuros/nyy001. |
| 557 | Abdelazeem RM, Youssef D, El-Azab J, Hassab-Elnaby S, Agour M. Three-dimensional visualization of brain tumor progression based accurate segmentation via comparative holographic projection. PloS one. 2020;15(7 July). doi: 10.1371/journal.pone.0236835. |
| 558 | Payen JF, Faillot T, Audibert G, Vergnes MC, Bosson JL, Lestienne B, et al. Thromboprophylaxis in neurosurgery and head trauma. Annales francaises d'anesthesie et de reanimation. 2005;24(8):921-7. doi: 10.1016/j.annfar.2005.05.012. |
| 559 | Jo JT, Schiff D, Perry JR. Thrombosis in brain tumors. Seminars in thrombosis and hemostasis. 2014;40(3):325-31. doi: 10.1055/s-0034-1370791. |
| 560 | Goh KY, Poon WS, Chan DT, Ip CP. Tissue plasminogen activator expression in meningiomas and glioblastomas. Clinical neurology and neurosurgery. 2005;107(4):296-300. Epub 2005/05/12. doi: 10.1016/j.clineuro.2004.09.010. PubMed PMID: 15885387. |
| 561 | Castellino SM, Tooze JA, Flowers L, Hill DF, McMullen KP, Shaw EG, et al. Toxicity and efficacy of the acetylcholinesterase (AChe) inhibitor donepezil in childhood brain tumor survivors: A pilot study. Pediatric Blood and Cancer. 2012;59(3):540-7. doi: 10.1002/pbc.24078. |
| 562 | Mashiko T, Oguma H, Konno T, Gomi A, Yamaguchi T, Nagayama R, et al. Training of Intra-Axial Brain Tumor Resection Using a Self-Made Simple Device with Agar and Gelatin. World neurosurgery. 2018;109:e298-e304. doi: 10.1016/j.wneu.2017.09.162. |
| 563 | Fisher RS, Rausch JR, Ferrante AC, Prussien KV, Olshefski RS, Vannatta KA, et al. Trajectories of health behaviors across early childhood cancer survivorship. Psycho-oncology. 2019;28(1):68-75. doi: 10.1002/pon.4911. |
| 564 | Nitta T, Sato K, Okumura K. Transforming growth factor (TGF)-β like activity of intracranial meningioma and its effect on cell growth. Journal of the neurological sciences. 1991;101(1):19-23. doi: 10.1016/0022-510X(91)90014-X. PubMed Central PMCID: Collaborative Research. |
| 565 | Kanno H, Kuwabara T, Yasumitsu H, Umeda M. Transforming growth factors in urine from patients with primary brain tumors. Journal of neurosurgery. 1988;68(5):775-80. doi: 10.3171/jns.1988.68.5.0775. |
| 566 | Li X, Fang X, Yang G, Su S, Zhu L, Yu Z. TransU²-Net: An Effective Medical Image Segmentation Framework Based on Transformer and U²-Net. IEEE journal of translational engineering in health and medicine. 2023;11:441-50. doi: 10.1109/JTEHM.2023.3289990. |
| 567 | Jansson S, Khorram-Manesh A, Nilsson O, Kölby L, Tisell LE, Wängberg B, et al. Treatment of bilateral pheochromocytoma and adrenal medullary hyperplasia. 2006. p. 429-35. |
| 568 | Nukolova N, Baklaushev V, Khalansky A, Yusubalieva G, Sandalova T, Kabanov A, et al. Treatment of brain tumor by targeted cisplatin-loaded nanogels in rats. FEBS Journal. 2013;280:313-4. doi: 10.1111/febs.12340. |
| 569 | Veerasarn K, Veerasarn V. Treatment of brain tumors in Thailand from 2005 to 2014: Data from the national health security office. Journal of the Medical Association of Thailand. 2016;99:S74-S81. |
| 570 | Becattini C, Di Nisio M, Franco L, Lee A, Agnelli G, Mandalà M. Treatment of venous thromboembolism in cancer patients: The dark side of the moon. Cancer treatment reviews. 2021;96. doi: 10.1016/j.ctrv.2021.102190. |
| 571 | Villena-Suarez JR, Vicente W, Taxa L, Cuéllar L, Nuñez-Butrón MT, Villegas V, et al. Tuberculosis that mimics cancer: Cases referred to the national institute of neoplastic diseases, Lima-Peru. Revista peruana de medicina experimental y salud publica. 2018;35(1):77-83. |
| 572 | Satgé D, Nishi M, Sirvent N, Vekemans M, Chenard MP, Barnes A. A tumor profile in Patau syndrome (trisomy 13). American Journal of Medical Genetics, Part A. 2017;173(8):2088-96. doi: 10.1002/ajmg.a.38294. |
| 573 | Marinău LD, Singer CE, Meşină C, Niculescu EC, Puiu I, Petrescu IO, et al. Two girl patients with medulloblastoma. Case reports. Romanian journal of morphology and embryology = Revue roumaine de morphologie et embryologie. 2017;58(3):1103-8. |
| 574 | Kawasaki S, Misawa H, Kaneda R, Shizuku T, Tamura Y, Kondo T, et al. Type 2 diabetes is complicated by sleep apnoea syndrome: focussing in comorbidities. Diabetologia. 2018;61:S514-S5. doi: 10.1007/s00125-018-4693-0. |
| 575 | Chambless LB, Parker SL, Hassam-Malani L, McGirt MJ, Thompson RC. Type 2 diabetes mellitus and obesity are independent risk factors for poor outcome in patients with high-grade glioma. Journal of neuro-oncology. 2012;106(2):383-9. doi: 10.1007/s11060-011-0676-4. |
| 576 | Skjøth-Rasmussen J, Bøgeskov L, Sehested A, Klausen C, Broholm H, Nysom K. The use of 5-ALA to assist complete removal of residual non-enhancing part of childhood medulloblastoma: a case report. Child's Nervous System. 2015;31(11):2173-7. doi: 10.1007/s00381-015-2762-y. |
| 577 | Hu W, Ye J, Wang J, Ma X, Zhang Z. Use of kilovoltage X-ray volume imaging in patient dose calculation for head-and-neck and partial brain radiation therapy. Radiation Oncology. 2010;5(1). doi: 10.1186/1748-717X-5-29. |
| 578 | Safaee M, Sun MZ, Oh T, Aghi MK, Berger MS, McDermott MW, et al. Use of thrombin-based hemostatic matrix during meningioma resection: A potential risk factor for perioperative thromboembolic events. Clinical neurology and neurosurgery. 2014;119:116-20. doi: 10.1016/j.clineuro.2014.01.021. |
| 579 | Bladowska J, Zimny A, Guziński M, Hałoń A, Tabakow P, Czyż M, et al. Usefulness of perfusion weighted magnetic resonance imaging with signal-intensity curves analysis in the differential diagnosis of sellar and parasellar tumors: preliminary report. European journal of radiology. 2013;82(8):1292-8. |
| 580 | Yue Q, Isobe T, Shibata Y, Kawamura H, Anno I, Matsumura A. Usefulness of quantitative proton MR spectroscopy in the differentiation of benign and malignant meningioma. Sheng wu yi xue gong cheng xue za zhi = Journal of biomedical engineering = Shengwu yixue gongchengxue zazhi. 2011;28(6):1103-9. |
| 581 | Jones LW, Guill B, Keir ST, Carter K, Friedman HS, Bigner DD, et al. Using the theory of planned behavior to understand the determinants of exercise intention in patients diagnosed with primary brain cancer. Psycho-oncology. 2007;16(3):232-40. doi: 10.1002/pon.1077. |
| 582 | Hazany S, Hesselink JR, Healy JF, Imbesi SG. Utilization of glutamate/creatine ratios for proton spectroscopic diagnosis of meningiomas. Neuroradiology. 2007;49(2):121-7. doi: 10.1007/s00234-006-0167-z. |
| 583 | Fahey FH, Kinahan PE, Doot RK, Kocak M, Thurston H, Poussaint TY. Variability in PET quantitation within a multicenter consortium. Medical physics. 2010;37(7):3660-6. doi: 10.1118/1.3455705. |
| 584 | Fahey FH, Kinahan PE, Doot RK, Kocak M, Thurston H, Poussaint TY. Variability in PET quantitation within a multicenter consortium. Medical physics. 2010;37(7):3660-6. |
| 585 | Sollmann N, Bulubas L, Tanigawa N, Zimmer C, Meyer B, Krieg SM. The variability of motor evoked potential latencies in neurosurgical motor mapping by preoperative navigated transcranial magnetic stimulation. BMC neuroscience. 2017;18(1). doi: 10.1186/s12868-016-0321-4. |
| 586 | Van Tellingen O, Boogerd W, Nooijen WJ, Beijnen JH. The vascular compartment hampers accurate determination of teniposide penetration into brain tumor tissue. Cancer chemotherapy and pharmacology. 1997;40(4):330-4. doi: 10.1007/s002800050665. |
| 587 | Diaz M, Schiff D. Vascular complications in patients with brain tumors. Current opinion in oncology. 2022;34(6):698-704. doi: 10.1097/CCO.0000000000000875. |
| 588 | Lasica N, Djilvesi D, Papic V, Karan M, Jelaca B, Golubovic J, et al. Venous thromboembolic and hemorrhagic events after meningioma surgery: A single-center retrospective cohort study of risk factors. PloS one. 2022;17(8 August). doi: 10.1371/journal.pone.0273189. |
| 589 | Senders JT, Goldhaber NH, Cote DJ, Muskens IS, Dawood HY, De Vos FYFL, et al. Venous thromboembolism and intracranial hemorrhage after craniotomy for primary malignant brain tumors: a National Surgical Quality Improvement Program analysis. Journal of neuro-oncology. 2018;136(1):135-45. |
| 590 | Hamilton MG, Hull RD, Pineo GF. Venous thromboembolism in neurosurgery and neurology patients: A review. Neurosurgery. 1994;34(2):280-96. |
| 591 | Cote DJ, Dubois HM, Karhade AV, Smith TR. Venous Thromboembolism in Patients Undergoing Craniotomy for Brain Tumors: A U.S. Nationwide Analysis. Seminars in thrombosis and hemostasis. 2016;42(8):870-6. doi: 10.1055/s-0036-1592306. |
| 592 | Chan AT, Atiemo A, Diran LK, Licholai GP, McLaren Black P, Creager MA, et al. Venous thromboembolism occurs frequently in patients undergoing brain tumor surgery despite prophylaxis. Journal of thrombosis and thrombolysis. 1999;8(2):139-42. |
| 593 | Sjåvik K, Bartek J, Solheim O, Ingebrigtsen T, Gulati S, Sagberg LM, et al. Venous Thromboembolism Prophylaxis in Meningioma Surgery: A Population-Based Comparative Effectiveness Study of Routine Mechanical Prophylaxis with or without Preoperative Low-Molecular-Weight Heparin. World neurosurgery. 2016;88:320-6. |
| 594 | Panagopoulos D, Karydakis P, Noutsos G, Themistocleous M. Venous Thromboembolism Risk and Thromboprophylaxis in Pediatric Neurosurgery and Spinal Injury: Current Trends and Literature Review. Seminars in thrombosis and hemostasis. 2022;48(3):318-22. doi: 10.1055/s-0041-1733959. |
| 595 | Hisada Y, Geddings JE, Ay C, Mackman N. Venous thrombosis and cancer: From mouse models to clinical trials. Journal of Thrombosis and Haemostasis. 2015;13(8):1372-82. doi: 10.1111/jth.13009. |
| 596 | Steele CB, Thomas CC, Henley SJ, Massetti GM, Galuska DA, Agurs-Collins T, et al. Vital Signs: Trends in Incidence of Cancers Associated with Overweight and Obesity - United States, 2005-2014. MMWR Morbidity and mortality weekly report. 2017;66(39):1052-8. doi: 10.15585/mmwr.mm6639e1. |
| 597 | Wang Z, Zeng S, Liu L, Zhong X, Cai L. Weak correlation between total psoas muscle area and sarcopenia index for children with brain tumor. Nutrition in Clinical Practice. 2023;38(4):838-49. doi: 10.1002/ncp.10996. |
| 598 | Lehman RAW, Krishnamurthy S, Berlin CM. Weight and height deficits in children with brain stem tumors. Clinical pediatrics. 2002;41(5):315-21. doi: 10.1177/000992280204100504. |
| 599 | Maschio M, Sperati F, Dinapoli L, Vidiri A, Fabi A, Pace A, et al. Weight of epilepsy in brain tumor patients. Journal of neuro-oncology. 2014;118(2):385-93. doi: 10.1007/s11060-014-1449-7. |
| 600 | Morales JS, Valenzuela PL, Herrera-Olivares AM, Rincón-Castanedo C, Martín-Ruiz A, Castillo-García A, et al. What are the effects of exercise training in childhood cancer survivors? A systematic review. Cancer and Metastasis Reviews. 2020;39(1):115-25. doi: 10.1007/s10555-020-09852-3. |
| 601 | Weichenthal S, Olaniyan T, Christidis T, Lavigne E, Hatzopoulou M, Van Ryswyk K, et al. Within-city Spatial Variations in Ambient Ultrafine Particle Concentrations and Incident Brain Tumors in Adults. Epidemiology (Cambridge, Mass). 2020;31(2):177-83. doi: 10.1097/EDE.0000000000001137. |
| 602 | Fotovati A, Abu-Ali S, Wang PS, Deleyrolle LP, Lee C, Triscott J, et al. YB-1 bridges neural stem cells and brain tumor-initiating cells via its roles in differentiation and cell growth. Cancer research. 2011;71(16):5569-78. doi: 10.1158/0008-5472.CAN-10-2805. |
|  | **Studies were excluded as they were not related this issue (N=1311).** |
| 1 | Zuccoli G, Marcello N, Pisanello A, Servadei F, Vaccaro S, Mukherjee P, et al. Metabolic management of glioblastoma multiforme using standard therapy together with a restricted ketogenic diet: Case Report. Nutrition and Metabolism. 2010;7. doi: 10.1186/1743-7075-7-33. |
| 2 | Zou Y, Pitchumoni CS. Obesity, obesities and gastrointestinal cancers. Disease-a-month : DM. 2023;69(12):101592. Epub 2023/06/13. doi: 10.1016/j.disamonth.2023.101592. PubMed PMID: 37308362. |
| 3 | Zjukovitsj D, Allard M, Djekic J. [Hyperostosis frontalis interna : etiology and differential diagnosis]. Revue medicale de Liege. 2022;77(12):701-5. Epub 2022/12/10. PubMed PMID: 36484746. |
| 4 | Zincircioglu SB, Kaplan MA, Isikdogan A, Cil T, Karadayi B, Dirier A, et al. Contribution of low-molecular weight heparin addition to concomitant chemoradiotherapy in the treatment of glioblastoma multiforme. Journal of BUON : official journal of the Balkan Union of Oncology. 2012;17(1):124-7. |
| 5 | Zielinski R, Rusin A, Madden T, Conrad C, Johansen M, Fokt I, et al. Development of orally bioavailable formulation of WP1066 and its evaluation in vivo. Cancer research. 2015;75(15). doi: 10.1158/1538-7445.AM2015-4540. |
| 6 | Zhu JJ, Pfannl R, Do-Dai D, Yao K, Mignano J, Wu JK, et al. Postmortem pathological correlation of increasing MRI T2/flair hyperintensity changes in glioblastoma patients after treatment with bevacizumab. Neuro-oncology. 2010;12:iv114. doi: 10.1093/neuonc/noq116. |
| 7 | Zhou P, Mansukhani MM, Toskic D, Scalia S, Lee L, Wong SW, et al. Next Generation Sequencing Identifies Light-Chain Amyloid (AL)-Related Igvl Genes in Patients with λ Monoclonal Gammopathy of Undetermined Significance (MGUS) or Smoldering Multiple Myeloma (SMM). Blood. 2022;140:10048-9. |
| 8 | Zhou J, Ye J, Liang Y, Zhao J, Wu Y, Luo S, et al. scSE-NL V-Net: A Brain Tumor Automatic Segmentation Method Based on Spatial and Channel “Squeeze-and-Excitation” Network With Non-local Block. Frontiers in Neuroscience. 2022;16. doi: 10.3389/fnins.2022.916818. |
| 9 | Zhou C, Ding C, Wang X, Lu Z, Tao D. One-pass Multi-task Networks with Cross-task Guided Attention for Brain Tumor Segmentation. IEEE transactions on image processing : a publication of the IEEE Signal Processing Society. 2020. doi: 10.1109/TIP.2020.2973510. |
| 10 | Zheng Y, Teo K, Nga VDW, Yeo TT, Lim MJR. Risk factors for 30-day postoperative surgical site hematoma requiring evacuation after resection of brain metastases. Clinical neurology and neurosurgery. 2023;226:107617. Epub 2023/02/09. doi: 10.1016/j.clineuro.2023.107617. PubMed PMID: 36753860. |
| 11 | Zheng F, Pang Y, Li L, Pang Y, Zhang J, Wang X, et al. Applications of nanobodies in brain diseases. Frontiers in immunology. 2022;13:978513. Epub 2022/11/26. doi: 10.3389/fimmu.2022.978513. PubMed PMID: 36426363; PubMed Central PMCID: PMC9679430. |
| 12 | Zhao X, Wang L, Wei N, Zhang J, Ma W, Zhao H, et al. Risk factors of health care-associated infection in elderly patients: a retrospective cohort study performed at a tertiary hospital in China. BMC geriatrics. 2019;19(1):193. Epub 2019/07/22. doi: 10.1186/s12877-019-1208-x. |
| 13 | Zhao L, Chen T, Tang X, Li S, Liang R, Wang Y. Medulloblastoma malignant biological behaviors are associated with HOTAIR/miR-483-3p/CDK4 axis. Annals of Translational Medicine. 2020;8(14). doi: 10.21037/atm-20-5006. |
| 14 | Zhang Y, Liu PX, Hou W. Modeling of glioma growth using modified reaction-diffusion equation on brain MR images. Computer methods and programs in biomedicine. 2022;227:107233. Epub 2022/11/15. doi: 10.1016/j.cmpb.2022.107233. PubMed PMID: 36375418. |
| 15 | Zhang X, Lv H, Zhou Q, Elkholi R, Chipuk JE, Reddy MVR, et al. Preclinical pharmacological evaluation of a novel multiple kinase inhibitor, ON123300, in brain tumor models. Molecular cancer therapeutics. 2014;13(5):1105-16. doi: 10.1158/1535-7163.MCT-13-0847. PubMed Central PMCID: LC. |
| 16 | Zhang J, Jiang Z, Liu D, Sun Q, Hou Y, Liu B. 3D asymmetric expectation-maximization attention network for brain tumor segmentation. NMR in biomedicine. 2022;35(5):e4657. Epub 2021/12/04. doi: 10.1002/nbm.4657. PubMed PMID: 34859922. |
| 17 | Zhang H, Peeters S, Vengerovich G, Antoury L, Wan Park K, Suh J, et al. Risk Factors Associated with Postoperative CSF Leak in Nonsellar Tumors. Journal of Neurological Surgery, Part B Skull Base. 2022;83(SUPPL 1). doi: 10.1055/s-0042-1743742. |
| 18 | Zhang B, Brantley K, Rosenberg S, Kirkner G, Collins L, Ruddy K, et al. Second primary non-breast cancers in young breast cancer survivors. Cancer research. 2024;84(9). doi: 10.1158/1538-7445.SABCS23-PO3-11-10. |
| 19 | Zeng W, Law BYK, Wong VKW, Chan DSB, Mok SWF, Gao JJY, et al. HM30181A, a potent P-glycoprotein inhibitor, potentiates the absorption and in vivo antitumor efficacy of paclitaxel in an orthotopic brain tumor model. Cancer Biology and Medicine. 2020;17(4):986-1001. |
| 20 | Zbijewski W. High-resolution extremity cone-beam CT prototype system. Medical physics. 2018;45(6):e539. doi: 10.1002/mp.12938. |
| 21 | Zambrano PO, Lovato C. Development of Rapidly Progressive Cushing's Disease Following Pituitary Apoplexy Several Years Prior. Journal of the Endocrine Society. 2019;3. doi: 10.1210/js.2019-MON-408. |
| 22 | Zahedi M, Hizomi Arani R, Tohidi M, Haghighi S, Mehrpour M, Hadaegh F. Nasopharyngeal B-cell lymphoma with pan-hypopituitarism and oculomotor nerve palsy: a case report and review of the literature. BMC endocrine disorders. 2020;20(1):163. |
| 23 | Yuyama R, Mishima K, Fujimaki T, Suzuki I, Sasaki T, Ueno H, et al. [Clinical experience of autologous blood transfusion and fibrin glue in neurosurgery]. No shinkei geka Neurological surgery. 1998;26(8):685-90. Epub 1998/09/23. PubMed PMID: 9743997. |
| 24 | Yue Q, Shibata Y, Isobe T, Anno I, Kawamura H, Gong QY, et al. Absolute choline concentration measured by quantitative proton MR spectroscopy correlates with cell density in meningioma. Neuroradiology. 2009;51(1):61-7. Epub 2008/11/13. doi: 10.1007/s00234-008-0461-z. PubMed PMID: 19002445. |
| 25 | Yue Q, Isobe T, Shibata Y, Kawamura H, Anno I, Matsumura A. Usefulness of quantitative proton MR spectroscopy in the differentiation of benign and malignant meningioma. Sheng wu yi xue gong cheng xue za zhi = Journal of biomedical engineering = Shengwu yixue gongchengxue zazhi. 2011;28(6):1103-9. |
| 26 | Yu Z, Li X, Li J, Chen W, Tang Z, Geng D. HSA-net with a novel CAD pipeline boosts both clinical brain tumor MR image classification and segmentation. Computers in biology and medicine. 2024;170:108039. Epub 2024/02/04. doi: 10.1016/j.compbiomed.2024.108039. PubMed PMID: 38308874. |
| 27 | Yu X, Wu Y, Bai Y, Han H, Chen L, Gao H, et al. A lightweight 3D UNet model for glioma grading. Physics in medicine and biology. 2022;67(15). Epub 2022/06/30. doi: 10.1088/1361-6560/ac7d33. PubMed PMID: 35767979. |
| 28 | Yu W, Su Z, Wu Z, Mao X, Zheng W, Zeng Y. cDNA clone, prokaryotic expression and purification of human interleukin-13 receptor [alpha]2 chain. Cancer immunology, immunotherapy : CII. 2009;58(3):409-13. doi: 10.1007/s00262-008-0566-6. |
| 29 | Yu MR, Yun MR, Cho HB, Choi HB, Dong G, Choi SJ, et al. P2.09-11 JIN-A04, Highly Effective and Brain-penetrant Tyrosine Kinase Inhibitor Targeting HER2 Exon20 Insertion Mutations in NSCLC. Journal of Thoracic Oncology. 2023;18(11):S335-S6. doi: 10.1016/j.jtho.2023.09.585. |
| 30 | Yu MK, Baichwal V, Jones J, Mather G, Carlson R. MPC-6827: Antitumor activity in an orthotopic brain model and in combination with bevacizumab. Neuro-oncology. 2009;11(5):592-3. doi: 10.1215/15228517-2009-034. |
| 31 | Yu L, Yu Z, Sun L, Zhu L, Geng D. A brain tumor computer-aided diagnosis method with automatic lesion segmentation and ensemble decision strategy. Frontiers in Medicine. 2023;10. doi: 10.3389/fmed.2023.1232496. |
| 32 | Yu J, Zheng J, Xu W, Weng J, Gao L, Tao L, et al. Accuracy of (18)F-FDOPA Positron Emission Tomography and (18)F-FET Positron Emission Tomography for Differentiating Radiation Necrosis from Brain Tumor Recurrence. World neurosurgery. 2018;114:e1211-e24. |
| 33 | Yu H, Chen M, Zhang J. Nutritional assessment for 891 patients with common cancer in a cancer hospital of southwest China. Journal of Clinical Oncology. 2024;42(16). |
| 34 | Youngerman BE, Kosty JA, Gerges MM, Tabaee A, Kacker A, Anand VK, et al. Acellular dermal matrix as an alternative to autologous fascia lata for skull base repair following extended endoscopic endonasal approaches. Acta neurochirurgica. 2020;162(4):863-73. Epub 2020/02/13. doi: 10.1007/s00701- |
| 35 | Young JS, Cho NW, Casey-Clyde T, Santos R, Seo K, Phillips J, et al. Convection-delivered Adenoviral Gene Therapy Reprograms the Immunosuppressive Glioblastoma Microenvironment National Brain Tumor Society (NBTS) Award. Journal of neurosurgery. 2022;136(5). |
| 36 | Young G, Toretsky JA, Campbell AB, Eskenazi AE. Recognition of common childhood malignancies. American family physician. 2000;61(7):2144-54. Epub 2000/04/25. PubMed PMID: 10779255. |
| 37 | Yoshikawa MH, Rabelo NN, Telles JPM, Figueiredo EG. Modifiable risk factors for glioblastoma: a systematic review and meta-analysis. Neurosurgical review. 2023;46(1):143. Epub 2023/06/21. doi: 10.1007/s10143-023-02051-y. PubMed PMID: 37340151. |
| 38 | Yoshida T, Nakao K, Chiba Y, Terada Y, Ogiwara A, Yoshii K, et al. Analysis of endocrinopathy as late effects in Childhood cancer survivors at a single institute in Japan. International Journal of Pediatric Endocrinology. 2017;2017. doi: 10.1186/s13633-017-0054-x. |
| 39 | Yoshida K, Furuse M, Kaneoke Y, Sasou K, Motegi Y. Changes in tissue T1 of brain tumor following Gd-DTPA administration - Differences in T1 time course and tissue-blood ratio by histological types. Brain and Nerve. 1988;40(6):531-7. |
| 40 | Yilmaz E, Mayadev A, Kobota K, Cambier Z, Schmidt C, Fisahn C, et al. Functional neurorehabilitation using the hybroid assistive limb (HAL): A first experience in the United States. European Spine Journal. 2018;27(11):2911-2. doi: 10.1007/s00586-018-5770-2. |
| 41 | Yilmaz E, Fisahn C, Mayadev A, Schmidt C, Kobata K, Cambier Z, et al. Can Exoskeletons help us dealing with the Global Burden of Disease? Results from the 1-Year Follow-Up of an ongoing prospective Pilot study using the HAL Exoskeleton. European Spine Journal. 2019;28:2669-70. |
| 42 | Yifru S, Muluye D. Childhood cancer in Gondar University Hospital, Northwest Ethiopia. BMC research notes. 2015;8:474. Epub 2015/09/26. doi: 10.1186/s13104-015-1440-1. PubMed PMID: 26404043; PubMed Central PMCID: PMC4582631. |
| 43 | Yerrabothala S, Gourley BL, Ford JC, Ahmed SR, Guerin SJ, Wishart HA, et al. Coagulation activation in brain neoplasms. Blood. 2017;130. |
| 44 | Yeh WL, Lu DY, Lee MJ, Fu WM. Leptin induces migration and invasion of glioma cells through MMP-13 production. Glia. 2009;57(4):454-64. Epub 2008/09/25. doi: 10.1002/glia.20773. PubMed PMID: 18814267. |
| 45 | Yedinak CG, Hopkins S, Williams J, Ibrahim A, Cetas JS, Fleseriu M. Medical therapy with pasireotide in recurrent Cushing's disease: Experience of patients treated for at least 1 year at a single center. Frontiers in endocrinology. 2017;8(FEB). doi: 10.3389/fendo.2017.00035. |
| 46 | Yavaş Abalı Z, Öztürk AP, Baş F, Poyrazoğlu Ş, Akcan N, Kebudi R, et al. Long-Term Endocrinologic Follow-Up of Children with Brain Tumors and Comparison of Growth Hormone Therapy Outcomes: A Single-Center Experience. Turkish Archives of Pediatrics. 2023;58(3):308-13. |
| 47 | Yaqub M, Jinchao F, Zia MS, Arshid K, Jia K, Rehman ZU, et al. State-of-the-art CNN optimizer for brain tumor segmentation in magnetic resonance images. Brain Sciences. 2020;10(7):1-19. doi: 10.3390/brainsci10070427. |
| 48 | Yannoutsos A, Cacciatore C, Jaouen S, Farge D, Frere C. Treatment of cancer-associated venous thromboembolism: A focus on special populations. Journal de medecine vasculaire. 2023;48(3-4):124-35. Epub 2023/11/02. doi: 10.1016/j.jdmv.2023.09.001. PubMed PMID: 37914457. |
| 49 | Yang W, Barth RF, Rotaru JH, Moeschberger ML, Joel DD, Nawrocky MM, et al. Enhanced survival of glioma bearing rats following boron neutron capture therapy with blood-brain barrier disruption and intracarotid injection of boronophenylalanine. Journal of neuro-oncology. 1997;33(1-2):59-70. |
| 50 | Yang SH. Risk factors associated with late hyperglycemia in brain tumor patients with postoperative administration of dexamethasone. Neuro-oncology. 2019;21:vi179. doi: 10.1093/neuonc/noz175.747. |
| 51 | Yang JU, Kim S, Lee KC, Lee YJ, Kim JY, Park JA. Development of Brain-Tumor-Targeted Benzothiazole-Based Boron Complex for Boron Neutron Capture Therapy. ACS Medicinal Chemistry Letters. 2022;13(10):1615-20. doi: 10.1021/acsmedchemlett.2c00284. |
| 52 | Yang J, He Z, Li M, Hong T, Ouyang T. Risk of intracranial hemorrhage with direct oral anticoagulation versus low molecular weight heparin in the treatment of brain tumor-associated venous thromboembolism: A meta-analysis. Journal of stroke and cerebrovascular diseases : the official journal of National Stroke Association. 2023;32(8):107243. |
| 53 | Yang A, Kim J, Lee JW, Yoo KH, Sung KW, Koo HH, et al. The impact of growth hormone treatment on height gain over 3 years and final height of childhood cancer survivors after hematopoietic SCT with craniospinal irradiation or total body irradiation. Hormone research in paediatrics. 2017;88:129-30. doi: 10.1159/000481424. |
| 54 | Yanardag H, Uygun S, Yumuk V, Caner M, Canbaz B. Cerebral tuberculosis mimicking intracranial tumour. Singapore medical journal. 2005;46(12):731-3. Epub 2005/11/26. PubMed PMID: 16308650. |
| 55 | Yan Y, Hui P, Lu Z, Wang R, Zhang B, Yu Z, et al. Application value of intraoperative microvascular Doppler ultrasound in neuroendoscopic transnasal resection of sellar tumors. Chinese Journal of Neurosurgery. 2022;38(2):117-22. doi: 10.3760/cma.j.cn112050-20210825-00420. |
| 56 | Yamashita S, Kaneda K, Han TH, Baker MT, Todd MM. Pharmacokinetic basis of mannitol administration after single bolus dose for the treatment of raised ICP. Journal of neurosurgical anesthesiology. 2009;21(4):385-6. doi: 10.1097/01.ana.0000358102.35410.08. |
| 57 | Yamashita K. [11C]-L-Methionine positron emission tomography/computed tomography in the management of children with brain tumors. Pediatric Radiology. 2011;41:S329. doi: 10.1007/s00247-011-2025-3. |
| 58 | Yaman A, Yücesan C, Isikay C, Yücemen N. Pseudotumor cerebri: A report of 24 patients with emphasis on the clinical manifestations and etiology. Neurologia Croatica. 2002;51(1-2):19-24+105-10. |
| 59 | Yamada M, Anzo M, Hasegawa Y. Evaluation of IGF-I levels in subjects whose GH secretion status was judged mainly by auxological data. Clinical Pediatric Endocrinology. 2003;12(2):87-92. doi: 10.1297/cpe.12.87. |
| 60 | Xu J, Ren X, Tran A, Shackleford GM, Erdreich-Epstein A. PID1, a new growth-inhibitory gene, sensitizes brain tumor cell lines to chemotherapy. Cancer research. 2014;74(19). doi: 10.1158/1538-7445.AM2014-3975. |
| 61 | Xing X, Yu L, Zhu L, Xing L, Liu L. Multi-Contrast MRI Acceleration with K-Space Progressive Learning and Image-Space Self-to-Peer Aggregation. 2024. p. S139-S40. |
| 62 | Xie YG, Han FY, Peyrard M, Ruttledge MH, Fransson I, DeJong P, et al. Cloning of a novel, anonymous gene from a megabase-range YAC and cosmid contig in the neurofibromatosis type 2/meningioma region on human chromosome 22q12. Human molecular genetics. 1993;2(9):1361-8. |
| 63 | Xie T, Ding YH, Sang CS, Lin ZX, Dong J, Fu XA. Vitexin enhances radiosensitivity of mouse subcutaneous xenograft glioma by affecting the miR-17-5p/miR-130b-3p/PTEN/HIF-1α pathway. Strahlentherapie und Onkologie : Organ der Deutschen Rontgengesellschaft [et al]. 2024;200(6):535-43. |
| 64 | Xie MY, Lin ZY, Sun XF, Feng JJ, Mai L, Wu CC, et al. Per- and polyfluoroalkyl substances (PFAS) exposure in plasma and their blood-brain barrier transmission efficiency-A pilot study. Environment international. 2024;187:108719. Epub 2024/05/09. doi: 10.1016/j.envint.2024.108719. |
| 65 | Xie MG, Wang XF, Qiao J, Zhou J, Guan YG, Liu CQ, et al. The long-term surgical outcomes of low-grade epilepsy-associated neuroepithelial tumors. Epilepsia open. 2022;7(4):697-709. Epub 2022/09/10. doi: 10.1002/epi4.12648. PubMed PMID: 36081402; PubMed Central PMCID: PMC9712488. |
| 66 | Wu S, Cooksey RM, Vega G, Klesse LJ, Bowers DC. Effects of hypothalamic radiation therapy exposure on cardiometabolic risk factors and bone density in survivors of childhood brain tumors. Journal of Clinical Oncology. 2015;33(15). |
| 67 | Wu DM, Hong XW, Wen X, Han XR, Wang S, Wang YJ, et al. MCL1 gene silencing promotes senescence and apoptosis of glioma cells via inhibition of the PI3K/Akt signaling pathway. IUBMB life. 2019;71(1):81-92. Epub 2018/10/09. doi: 10.1002/iub.1944. PubMed PMID: 30296359. |
| 68 | Woolf SH, Chapman DA, Buchanich JM, Bobby KJ, Zimmerman EB, Blackburn SM. Changes in midlife death rates across racial and ethnic groups in the United States: systematic analysis of vital statistics. BMJ (Clinical research ed). 2018;362:k3096. Epub 2018/08/17. doi: 10.1136/bmj.k3096. |
| 69 | Wolf S, Riess A, Landscheidt JF, Lumenta CB, Schürer L, Friederich P. How to perform indexing of extravascular lung water: a validation study. Critical care medicine. 2013;41(4):990-8. Epub 2013/02/08. doi: 10.1097/CCM.0b013e318275cd75. PubMed PMID: 23388510. |
| 70 | Wolf S, Riess A, Landscheidt JF, Lumenta CB, Friederich P, Schürer L. Global end-diastolic volume acquired by transpulmonary thermodilution depends on age and gender in awake and spontaneously breathing patients. Critical care (London, England). 2009;13(6):R202. |
| 71 | Wolf S, Riess A, Landscheidt J, Lumenta C, Schuerer L, Friederich P. How to perform indexing of extravascular lung water data. Critical Care. 2012;16:S88-S9. doi: 10.1186/cc10853. |
| 72 | Wolf S, Friederich P, Landscheidt J, Lumenta C, Schuerer L, Riess A. The pulmonary vascular permeability index PVPI is dependent on age and body height of a patient. Intensive care medicine. 2012;38:S196. doi: 10.1007/s00134-012-2683-0. |
| 73 | Wójcik M, Dolezal-Ołtarzewska K, Kumorowicz-Czoch M, Kalicka-Kasperczyk A, Januś D, Zygmunt-Górska A, et al. [Long-term endocrine complications after brain tumor treatment--own experience]. Przeglad lekarski. 2010;67(11):1132-5. Epub 2010/01/01. PubMed PMID: 21442963. |
| 74 | Winn M, Pauleck S, Viskochil R, Richardson S, Litchman M, Colman H, et al. Incident diabetes by obesity-related cancer status and cancer treatment type. Cancer research. 2023;83(7). doi: 10.1158/1538-7445.AM2023-741. |
| 75 | Williams EC, Wiranowska M, Toomey R, Alcantar N. Thermo-responsive double packaged system for localized drug delivery for cancer treatment. Cancer research. 2011;71(8). doi: 10.1158/1538-7445.AM2011-3229. |
| 76 | Wilhelmy F, Hantsche A, Wende T, Kasper J, Reuschel V, Frydrychowicz C, et al. Perioperative anticoagulation in patients with intracranial meningioma: No increased risk of intracranial hemorrhage? PloS one. 2020;15(9):e0238387. |
| 77 | Wiler JL, Price C, Fintak DR, Shields C. Leukocoria and irregular pupil. American Journal of Emergency Medicine. 2010;28(1):121.e5-.e8. doi: 10.1016/j.ajem.2009.05.013. |
| 78 | Whitson WJ, Valdes PA, Harris BT, Paulsen KD, Roberts DW. Confocal microscopy for the histological fluorescence pattern of a recurrent atypical meningioma: case report. Neurosurgery. 2011;68(6):E1768-72; discussion E72-3. |
| 79 | Wetterau L, Cohen P. New paradigms for growth hormone therapy in children. Hormone research. 2000;53 Suppl 3:31-6. Epub 2000/09/06. doi: 10.1159/000023530. PubMed PMID: 10971101. |
| 80 | Wertz D, Boveroux P, Péters P, Lenelle J, Franssen C. Surgical resection of a sphenoid wing meningioma in a patient with Glanzmann thrombasthenia. Acta anaesthesiologica Belgica. 2011;62(2):83-6. Epub 2011/09/17. PubMed PMID: 21919374. |
| 81 | Wermelt J, Ständer S, Pereira MP, Mannil M. [Chronic brachioradial pruritus in cervical spine meningioma]. Der Hautarzt; Zeitschrift fur Dermatologie, Venerologie, und verwandte Gebiete. 2022;73(6):485-7. Epub 2021/10/06. doi: 10.1007/s00105-021-04897-z. PubMed PMID: 34609536. |
| 82 | Wen PY, Schiff D, Kesari S, Drappatz J, Gigas DC, Doherty L. Medical management of patients with brain tumors. Journal of neuro-oncology. 2006;80(3):313-32. Epub 2006/06/30. doi: 10.1007/s11060-006-9193-2. PubMed PMID: 16807780. |
| 83 | Weichenthal S, Olaniyan T, Christidis T, Lavigne E, Hatzopoulou M, Van Ryswyk K, et al. Within-city Spatial Variations in Ambient Ultrafine Particle Concentrations and Incident Brain Tumors in Adults. Epidemiology (Cambridge, Mass). 2020;31(2):177-83. |
| 84 | Weber RG, Hoischen A, Ehrler M, Zipper P, Kaulich K, Blaschke B, et al. Frequent loss of chromosome 9, homozygous CDKN2A/p14(ARF)/CDKN2B deletion and low TSC1 mRNA expression in pleomorphic xanthoastrocytomas. Oncogene. 2007;26(7):1088-97. |
| 85 | Wang Z, Zhang Y, Guo Z. Advances in the research of the relationship between tumor and venous thromboembolism. Chinese Journal of Clinical Oncology. 2008;35(24):1429-31+33. |
| 86 | Wang Z, Zeng S, Liu L, Zhong X, Cai L. Weak correlation between total psoas muscle area and sarcopenia index for children with brain tumor. Nutrition in clinical practice : official publication of the American Society for Parenteral and Enteral Nutrition. 2023;38(4):838-49. |
| 87 | Wang Z, Li Y, Jones JB, Zhang Z, Mao H. Targeting Tumor-Associated M2-like Macrophages in a Patient-derived Xenograft Model of Glioblastoma Using Sub-5 nm Iron Oxide Nanoparticles. Molecular imaging and biology. 2022;24:S427-S8. doi: 10.1007/s11307-022-01794-2. |
| 88 | Wang Y, Liu X, Guan G, Zhao W, Zhuang M. A risk classification system with five-gene for survival prediction of glioblastoma patients. Frontiers in Neurology. 2019;10(JUL). doi: 10.3389/fneur.2019.00745. |
| 89 | Wang X, Duan X, Yang G, Zhang X, Deng L, Zheng H, et al. Honokiol crosses BBB and BCSFB, and inhibits brain tumor growth in rat 9L intracerebral gliosarcoma model and human U251 xenograft glioma model. PloS one. 2011;6(4):e18490. doi: 10.1371/journal.pone.0018490. |
| 90 | Wang W, Aafreen S, Terrilion C, Liu G, Ngen EJ. Magnetic resonance imaging of the retention of biohybrid extracellular vesicles in the brain for sustained drug release in the repair of radiotherapy-induced brain injury. Molecular imaging and biology. 2022;24:S133. doi: 10.1007/s11307-022-01794-2. |
| 91 | Wang R, Zhu Z, Li Z, Li D, Chen X, Zhang J. Clinical Translation of a Dual Integrin αvβ3 and Gastrin-Releasing Peptide Receptor Targeting PET tracer: 68Ga-DOTA-RM26-RGD. Journal of Nuclear Medicine. 2023;64. |
| 92 | Wang Q, Zheng D, Li Y, Zhang Y, Sui R, Chen Y, et al. Circular RNA circ_0001588 sponges miR-211-5p to facilitate the progression of glioblastoma via up-regulating YY1 expression. The journal of gene medicine. 2021;23(10):e3371. |
| 93 | Wang P, Zheng SY, Jiang RL, Wu HD, Li YA, Lu JL, et al. Necroptosis signaling and mitochondrial dysfunction cross-talking facilitate cell death mediated by chelerythrine in glioma. Free radical biology & medicine. 2023;202:76-96. |
| 94 | Wang L, Xu R, Kaelber DC, Berger NA. Glucagon-Like Peptide 1 Receptor Agonists and 13 Obesity-Associated Cancers in Patients With Type 2 Diabetes. JAMA network open. 2024;7(7):e2421305. Epub 2024/07/05. doi: 10.1001/jamanetworkopen.2024.21305. |
| 95 | Wang L, Liu Z, Balivada S, Pyle M, Cote GJ, Shi J, et al. Reprogramming towards glioma stem-like cells by cytokines. Cancer research. 2011;71(8). doi: 10.1158/1538-7445.AM2011-LB-98. |
| 96 | Wang KW, Valencia M, Banfield L, Chau R, Fleming A, Singh SK, et al. The effectiveness of interventions to treat obesity in survivors of childhood brain tumors: a systematic review protocol. Systematic reviews. 2016;5(1):101. |
| 97 | Wang KW, Fleming A, Singh SK, Banfield L, de Souza RJ, Thabane L, et al. Evaluating overweight and obesity prevalence in survivors of childhood brain tumors: a systematic review protocol. Systematic reviews. 2017;6(1):43. Epub 2017/03/04. doi: 10.1186/s13643-017-0439-1. |
| 98 | Wang KW, Fleming A, Burrow S, De Souza RJ, Thabane L, Samaan MC. Children with brain tumors have enhanced visceral adiposity compared to non-cancer controls: A preliminary analysis from the Canadian study of determinants of endometabolic health in children Study. Hormone research in paediatrics. 2016;86:523. doi: 10.1159/000449142. |
| 99 | Wang C, Li P. Risk factors for intraoperative blood loss in resection of intracranial meningioma: Analysis of 530 cases. PloS one. 2023;18(9):e0291171. Epub 2023/09/08. doi: 10.1371/journal.pone.0291171. PubMed PMID: 37682850; PubMed Central PMCID: PMC10490957. |
| 100 | Walsh KM, Zhang C, Calvocoressi L, Hansen HM, Berchuck A, Schildkraut JM, et al. Pleiotropic MLLT10 variation confers risk of meningioma and estrogen-mediated cancers. Neuro-Oncology Advances. 2022;4(1). doi: 10.1093/noajnl/vdac044. |
| 101 | Walsh KM. Epidemiology of meningiomas. Handbook of clinical neurology. 2020;169:3-15. Epub 2020/06/20. doi: 10.1016/b978-0-12-804280-9.00001-9. PubMed PMID: 32553297. |
| 102 | Walsh K, Zhang C, Calvocoressi L, Hansen H, Berchuck A, Schildkraut J, et al. Pleiotropic MLLT10 variation confers risk of meningioma, breast, and ovarian cancers. Neuro-oncology. 2019;21:vi142. doi: 10.1093/neuonc/noz175.594. |
| 103 | Von Hoff K, Herzog N, Ottensmeier H, Grabow D, Gerber NU, Friedrich C, et al. Long-term endocrinological impairments and quality of life of patients with medulloblastoma treated within the hit'91 study. Neuro-oncology. 2012;14:i132. doi: 10.1093/neuonc/nos106. |
| 104 | Von Ehrlich B. Polytopic painful syndromes of symptomatic soft tissue calcification following chronic magnesium depletion: Preventive options of an ignored association. Trace Elements and Electrolytes. 2023;40(2):95-6. doi: 10.5414/TEX01694. |
| 105 | Voelter-Mahlknecht S, Ho AD, Mahlknecht U. FISH-mapping and genomic organization of the NAD-dependent histone deacetylase gene, Sirtuin 2 (Sirt2). International journal of oncology. 2005;27(5):1187-96. Epub 2005/10/08. PubMed PMID: 16211212. |
| 106 | Vinchon-Petit S, Jarnet D, Jadaud E, Feuvret L, Garcion E, Menei P. External irradiation models for intracranial 9L glioma studies. Neuro-oncology. 2010;12:iv89. doi: 10.1093/neuonc/noq116. |
| 107 | Vimala M, Ranjith Kumar P. Real-time Multi Fractal Ensemble Analysis CNN Model for Optimizing Brain Tumor Classification and Survival Prediction Using SVM. Biomedical and Pharmacology Journal. 2023;16(1):305-18. doi: 10.13005/bpj/2612. |
| 108 | Villena-Suarez JR, Vicente W, Taxa L, Cuéllar L, Nuñez-Butrón MT, Villegas V, et al. [Tuberculosis That Mimics Cancer: Cases Referred to the National Institute of Neoplastic Diseases, Lima-Peru]. Revista peruana de medicina experimental y salud publica. 2018;35(1):77-83. |
| 109 | Viker K, Concilio SC, Clark SM, Robinson S, Dyer R, Gades N, et al. Preclinical Toxicology Assessment of Intracranial Administration of an Oncolytic Measles Virus Strain Armed with H. pylori Neutrophil-Activating Protein Prior to Phase I Trial in Glioblastoma Patients. Molecular Therapy. 2024;32(4):825. doi: 10.1016/j.ymthe.2024.04.020. |
| 110 | Vijaykumar M, Prasad N, Eshwar R, Ashok, Ramanna, Devi U, et al. An unusual case of dyspepsia. Indian Journal of Gastroenterology. 2012;31(1):A22. doi: 10.1007/s12664-012-0264-3. |
| 111 | Verhoeff JJ, Stalpers LJ, Coumou AW, Koedooder K, Lavini C, Van Noorden CJ, et al. Experimental iodine-125 seed irradiation of intracerebral brain tumors in nude mice. Radiation oncology (London, England). 2007;2:38. |
| 112 | Vengerovich G, Antoury L, Wan Park K, Heaney AP, Bergsneider M, Wang MB. Postoperative CSF leaks following endoscopic anterior skull base surgery: Risk factors and management. Journal of Neurological Surgery, Part B: Skull Base. 2019;80. doi: 10.1055/s-0039-1679426. |
| 113 | Veluru M, Anolik J. A Case of Cushing's Syndrome due to Primary Bilateral Macronodular Adrenal Hyperplasia. Journal of the Endocrine Society. 2022;6:A49-A50. doi: 10.1210/jendso/bvac150.102. |
| 114 | Vella R, Kanzaki L, Mudhar A, Chakraborty S. UNRAVELING THE GUT-BRAIN CONNECTION: INTERPLAY OF AUTONOMIC SYMPTOMS, ANXIETY AND NAUSEA IN CHRONIC NAUSEA SUFFERERS. Gastroenterology. 2024;166(5):S-776. doi: 10.1016/S0016-5085(24)02267-4. |
| 115 | Velasco-Hidalgo L, García-Guzmán AD, Rodríguez-Aguilar EA, Pinzón-Navarro BA, Guevara-Cruz M, Zapata-Tarrés M, et al. DEVELOPMENT OF NEW PREDCITIVE EQUATION FOR RESTING ENERGY EXPENDITURE IN PEDIATRIC PATIENTS WITH ONCOLOGY DIAGNOSIS. Pediatric Blood and Cancer. 2022;69:S389. doi: 10.1002/pbc.29952. |
| 116 | Velasco P, Clemente M, Lorite R, Ventura MC, Gros L, Sanchez de Toledo J, et al. The role of leptin in diencephalic syndrome. Pediatrics. 2014;133(1):e263-6. Epub 2013/12/25. doi: 10.1542/peds.2012-3196. PubMed PMID: 24366994. |
| 117 | Veerasarn K, Veerasarn V. Treatment of Brain Tumors in Thailand from 2005 to 2014: Data from the National Health Security Office. Journal of the Medical Association of Thailand = Chotmaihet thangphaet. 2016;99 Suppl 3:S74-81. Epub 2016/06/01. PubMed PMID: 29901347. |
| 118 | Vassantachart A, Cao Y, Gribble M, Guzman S, Ye JC, Hurth K, et al. Automatic Differentiation of Grade I and II Meningioma on Magnetic Resonance Image Using an Asymmetric Convolutional Neural Network. International Journal of Radiation Oncology Biology Physics. 2021;111(3):e561-e2. doi: 10.1016/j.ijrobp.2021.07.1520. |
| 119 | Varma VR, Rangarajan SV, Siu A, Rabinowitz MR, Nyquist GG, Evans JJ, et al. Bilayer button graft for endoscopic repair of high-flow cranial base defects. Journal of Neurological Surgery Part B: Skull Base. 2017;78. doi: 10.1055/s-0037-1600695. |
| 120 | Vaneckova M, Herman M, Smith MP, Mechl M, Maravilla KR, Weichet J, et al. The Benefits of High Relaxivity for Brain Tumor Imaging: Results of a Multicenter Intraindividual Crossover Comparison of Gadobenate Dimeglumine with Gadoterate Meglumine (The BENEFIT Study). AJNR American journal of neuroradiology. 2015;36(9):1589-98. |
| 121 | Vande Griend JP, Linnebur SA, Bainbridge JL. Probable levetiracetam-associated depression in the elderly: two case reports. The American journal of geriatric pharmacotherapy. 2009;7(5):281-4. Epub 2009/12/02. doi: 10.1016/j.amjopharm.2009.10.002. PubMed PMID: 19948304. |
| 122 | van Tellingen O, Boogerd W, Nooijen WJ, Beijnen JH. The vascular compartment hampers accurate determination of teniposide penetration into brain tumor tissue. Cancer chemotherapy and pharmacology. 1997;40(4):330-4. Epub 1997/01/01. doi: 10.1007/s002800050665. PubMed PMID: 9225951. |
| 123 | Van Schaik J, Welling M, De Groot C, Abawi O, Burghard M, Kleinendorst L, et al. Dextro-Amphetamine Treatment for Hypothalamic Obesity in Children Surviving Brain Tumors or Other Causes. Pediatric Blood and Cancer. 2021;68(SUPPL 5). doi: 10.1002/pbc.29349. |
| 124 | van Schaik J, van Roessel IMAA, Schouten-Van Meeteren NAYN, van Iersel L, Clement SC, Boot AM, et al. High Prevalence of Weight Gain in Childhood Brain Tumor Survivors and Its Association with Hypothalamic-Pituitary Dysfunction. Journal of Clinical Oncology. 2021;39(11):1264-73. |
| 125 | van Schaik J, van Roessel I, Bos ID, Claashen-van der Grinten HL, Clement SC, van Iersel L, et al. Elevated IGF-1 concentrations in children with low grade glioma: A descriptive analysis in a retrospective national cohort. Journal of neuroendocrinology. 2023;35(8):e13317. |
| 126 | Van Schaik J, Burghard M, Lequin MH, van Maren EA, van Dijk AM, Takken T, et al. Resting energy expenditure in children at risk of hypothalamic dysfunction. Endocrine Connections. 2022;11(8). doi: 10.1530/EC-22-0276. |
| 127 | Van Schaik J, Burghard M, Lequin M, Van Maren E, Van Dijk A, Takken T, et al. RESTING ENERGY EXPENDITURE CORRELATES WITH CLINICAL AND RADIOLOGICAL HYPOTHALAMIC DAMAGE IN CHILDREN SURVIVING A SUPRASELLAR BRAIN TUMOR. Supportive Care in Cancer. 2022;30:S139-S40. |
| 128 | Van Schaik J, Burghard M, Lequin M, Van Maren E, Van Dijk A, Takken T, et al. LOW RESTING ENERGY EXPENDITURE IS ASSOCIATED WITH CLINICAL AND RADIOLOGICAL HYPOTHALAMIC DAMAGE IN CHILDREN SURVIVING A SUPRASELLAR BRAIN TUMOR. Neuro-oncology. 2022;24:i10. |
| 129 | Van Schaik J, Burghard M, Lequin M, Van Dijk A, Takken T, Rehorst L, et al. Low Resting Energy Expenditure Correlates with Clinical and Radiological Hypothalamic Damage in Children Surviving A Brain Tumor. Pediatric Blood and Cancer. 2021;68(SUPPL 5). doi: 10.1002/pbc.29349. |
| 130 | van Schaik J, Begijn DGA, van Iersel L, Vergeer Y, Hoving EW, Peeters B, et al. Experiences with Glucagon-Like Peptide-1 Receptor Agonist in Children with Acquired Hypothalamic Obesity. Obesity facts. 2020;13(4):361-70. |
| 131 | van Santen HM, Denzer C, Müller HL. Could setmelanotide be the game-changer for acquired hypothalamic obesity? Frontiers in endocrinology. 2023;14:1307889. Epub 2024/01/19. doi: 10.3389/fendo.2023.1307889. PubMed PMID: 38239988; PubMed Central PMCID: PMC10794340. |
| 132 | van Roessel IMAA, de Graaf JP, Biermasz NR, Charmandari E, van Santen HM. Acquired hypothalamic dysfunction in childhood: ‘what do patients need?’ – an Endo-ERN survey. Endocrine Connections. 2023;12(10). doi: 10.1530/EC-23-0147. |
| 133 | van Roessel I, van Schaik J, Meeteren A, Boot AM, der Grinten HLC, Clement SC, et al. Body mass index at diagnosis of a childhood brain tumor; a reflection of hypothalamic-pituitary dysfunction or lifestyle? Supportive care in cancer : official journal of the Multinational Association of Supportive Care in Cancer. 2022;30(7):6093-102. |
| 134 | Van Raemdonck D, Dupont L, Coosemans W, Decaluwé H, De Leyn P, Lerut T, et al. Five hundred consecutive isolated lung transplantations; a single center experience. Interactive Cardiovascular and Thoracic Surgery. 2011;13:S52. doi: 10.1510/icvts.2011.0000S6. |
| 135 | Van Ommen CCN, Van Iersel L, Lequin MH, Clement SC, Janssens GOR, Boot AM, et al. MRI changes in time after cranial irradiation, and their relation with pituitary function in survivors of childhood medulloblastoma. Hormone research in paediatrics. 2018;90:526. doi: 10.1159/000492307. |
| 136 | Van Ommen C, Iersel L, Lequin M, Janssens G, Clement S, Boot A, et al. Radiological Alterations Of The Hypothalamic-pituitary Region After Craniospinal Irradiation For Medulloblastoma During Childhood. Journal of the Endocrine Society. 2019;3. doi: 10.1210/js.2019-MON-458. |
| 137 | van Iersel L, Xu J, Potter BS, Conklin HM, Zhang H, Chemaitilly W, et al. Clinical Importance of Free Thyroxine Concentration Decline After Radiotherapy for Pediatric and Adolescent Brain Tumors. The Journal of clinical endocrinology and metabolism. 2019;104(11):4998-5007. |
| 138 | Van Duyvenvoorde H, Kant S, Pereira A, Autar A, Oostdijk W, Breuning M, et al. A maternally transmitted novel variant in the IGF1R observed in two unrelated tall males suggests that IGF1R may be imprinted and that the variant is activating. Hormone research in paediatrics. 2011;76:102. doi: 10.1159/000334326. |
| 139 | van de Vijfeijken SECM, Münker TJAG, de Jager N, Vandertop WP, Becking AG, Kleverlaan CJ, et al. Properties of an In Vivo Fractured Poly(Methyl Methacrylate) Cranioplasty After 15 Years. World neurosurgery. 2019;123:e60-e8. doi: 10.1016/j.wneu.2018.11.026. |
| 140 | Vajtai I, von Gunten M, Fung C, Brekenfeld C, Kappeler A, Reinert MM. Oncocytic ependymoma: a new morphological variant of high-grade ependymal neoplasm composed of mitochondrion-rich epithelioid cells. Pathology, research and practice. 2011;207(1):49-54. |
| 141 | Utsuki S, Oka H, Abe K, Osawa S, Yamazaki T, Yasui Y, et al. Primary central nervous system lymphoma in acquired immune deficiency syndrome mimicking toxoplasmosis. Brain Tumor Pathology. 2011;28(1):83-7. doi: 10.1007/s10014-010-0001-5. |
| 142 | Uthoff H, Quesada R, Katzen B, Kreusch A, Staub D. Radioprotective light-weight caps in the interventional setting: A randomized controlled trial (PROTECT-trial). Vasa - European Journal of Vascular Medicine. 2015;44:40-1. doi: 10.1024/0301-1526/a000476. |
| 143 | Unteregger G, Zang KD, Issinger OG. Two-dimensional polyacrylamide gel electrophoresis of nuclear proteins in human meningiomas. Electrophoresis. 1983;4(4):303-11. |
| 144 | Unal S, Peker E. EVALUATION of CORPUS CALLOSUM, OPTIC and OLFACTORY NERVE SIZES with MRI in MS PATIENTS. Neuroradiology. 2023;65:S96. doi: 10.1007/s00234-023-03203-z. |
| 145 | Ünal S, Peker E. Evaluation of corpus callosum, optic and olfactory nerve sizes with MRI in MS patients. Neuroradiology. 2022;64(2):420. doi: 10.1007/s00234-021-02887-5. |
| 146 | Tzeng SY, Green JJ. Subtle changes to polymer structure and degradation mechanism enable highly effective nanoparticles for siRNA and DNA delivery to human brain cancer. Advanced healthcare materials. 2013;2(3):468-80. |
| 147 | Tuǧcu D, Şahin Ş, Bilici M, Tuna R, Karaman S, Ünüvar A, et al. A rare childhood tumor: High grade B cell gastric lymphoma. Pediatric Blood and Cancer. 2020;67(SUPPL 4). doi: 10.1002/pbc.28742. |
| 148 | Tsuyumu M, Hoshi Y, Fuse M, Suganuma Y, Oata M. [Regional cerebral blood flow in senile patients with meningiomas (author's transl)]. No shinkei geka Neurological surgery. 1975;3(1):37-42. Epub 1975/01/01. PubMed PMID: 1238912. |
| 149 | Tsuchiya K, Imai M, Tateishi H, Nitatori T, Shiokawa Y. Ct angiographic surface anatomic scanning of brain tumors can be performed with a contrast agent of 54 Ml using dose adjustment by the patient weight and a linearly decelerated injection rate. Neuroradiology. 2011;53:S23 |
| 150 | Tsuchiya K, Imai M, Tateishi H, Nitatori T. Consecutive acquisition of time-resolved contrast-enhanced mra and perfusion mr imaging of brain tumors with a total contrast dose of 16 ml. Neuroradiology. 2011;53:S39. doi: 10.1007/s00234-011-0914-7. |
| 151 | Tsao GJ, Tsang MW, Mobley BC, Cheng WW. Foramen magnum meningioma: Dysphagia of atypical etiology. Journal of general internal medicine. 2008;23(2):206-9. Epub 2007/12/18. doi: 10.1007/s11606-007-0474-z. PubMed PMID: 18080720; PubMed Central PMCID: PMC2359174. |
| 152 | Tsao AS, Song Z, Ho AL, Mehnert JM, Mitchell EP, Wright JJ, et al. Phase II study of vismodegib in patients with SMO or PTCH1 mutated tumors: Results from NCI-MATCH ECOG-ACRIN Trial (EAY131) Subprotocol T. Journal of Clinical Oncology. 2022;40(16). doi: 10.1200/JCO.2022.40.16_suppl.3010. |
| 153 | Tsai YT, Wu AC, Yang WB, Kao TJ, Chuang JY, Chang WC, et al. Angptl4 induces tmz resistance of glioblastoma by promoting cancer stemness enrichment via the egfr/akt/4e-bp1 cascade. International journal of molecular sciences. 2019;20(22). doi: 10.3390/ijms20225625. |
| 154 | Tsai YT, Chen YT, Hsu FT, Tsai JJ, Liu YC. Amentoflavone enhances regorafenib-induced apoptosis and -inhibited invasion via inactivating STAT3/NF-KB signaling pathway in Hepatocellular carcinoma. Cancer research. 2024;84(6). doi: 10.1158/1538-7445.AM2024-1102. |
| 155 | Tsai SP, Wendt JK, Cardarelli KM, Fraser AE. A mortality and morbidity study of refinery and petrochemical employees in Louisiana. Occupational and environmental medicine. 2003;60(9):627-33. |
| 156 | Truong TH, Prokopishyn NL, Luu H, Guilcher GMT, Lewis VA. Predictive factors for successful peripheral blood stem cell mobilization and collection in children. Journal of clinical apheresis. 2019;34(5):598-606. Epub 2019/08/08. doi: 10.1002/jca.21738. PubMed PMID: 31390093. |
| 157 | Trotta FI, Palmiero R, Soffietti R. A CASE OF BING-NEEL SYNDROME AS THE FIRST MANIFESTATION OF WALDENSTROM MACROGLOBULIEMIA. Neurological Sciences. 2022;43:S107. doi: 10.1007/s10072-022-06531-9. |
| 158 | Triscott J, Rose Pambid M, Dunn SE. Concise review: bullseye: targeting cancer stem cells to improve the treatment of gliomas by repurposing disulfiram. Stem cells (Dayton, Ohio). 2015;33(4):1042-6. Epub 2015/01/16. doi: 10.1002/stem.1956. PubMed PMID: 25588723. |
| 159 | Tripathi MK, Ojha SK, Amal H. Nitric oxide is a target by a combo-drug for glioblastoma treatment. Annals of Oncology. 2023;34:S403. doi: 10.1016/j.annonc.2023.09.1726. |
| 160 | Toyota P, Persad AR, Liu E, Saini J, Zherebitskiy V, Auer R, et al. BMI as a predictor of recurrence in high-grade meningioma: A single center retrospective cohort study. Canadian Journal of Neurological Sciences. 2024;51:S41-S2. doi: 10.1017/cjn.2024.198. |
| 161 | Tosun H, Demirtas A, Baydilli N, Sabur V, Akinsal EC, Demirci D, et al. The results of radical nephrectomy. European Urology, Supplements. 2013;12(4):e1359. doi: 10.1016/S1569-9056(13)62284-8. |
| 162 | Tolstikova O. Unilateral autoimmune ophthalmopathy as a first symptom in manifestation of autoimmune thyroiditis and systemic lupus erythematosus in an adolescent girl. Case report. Hormone research in paediatrics. 2021;94(SUPPL 1):406. doi: 10.1159/000518849. |
| 163 | Tohidinezhad F, Zegers CML, Vaassen F, Dijkstra J, Anten M, Van Elmpt W, et al. Predicting the risk of neurocognitive decline after brain irradiation in adult patients with a primary brain tumor. Neuro-oncology. 2024;26(8):1467-78. doi: 10.1093/neuonc/noae035. |
| 164 | Todo T, Adams EF, Rafferty B, Fahlbusch R, Dingermann T, Werner H. Secretion of interleukin-6 by human meningioma cells: possible autocrine inhibitory regulation of neoplastic cell growth. Journal of neurosurgery. 1994;81(3):394-401. doi: 10.3171/jns.1994.81.3.0394. |
| 165 | Todo T, Adams EF, Fahlbusch R, Dingermann T, Werner H. Autocrine growth stimulation of human meningioma cells by platelet-derived growth factor. Journal of neurosurgery. 1996;84(5):852-8; discussion 8-9. Epub 1996/05/01. doi: 10.3171/jns.1996.84.5.0852. PubMed PMID: 8622161. |
| 166 | Tobassum S, Tahir HM, Arshad M, Zahid MT, Ali S, Ahsan MM. Nature and applications of scorpion venom: an overview. Toxin Reviews. 2020;39(3):214-25. doi: 10.1080/15569543.2018.1530681. |
| 167 | Tiulpakov MA, Bezlepkina OB, Nagaeva EV, Azizian VN, Lapshina AM. A CLINICAL CASE and BRIEF LITERATURE REVIEW of ICENKO–CUSHING’S DISEASE in A PEDIATRIC PATIENT with ATYPICAL ONSET of the DISEASE. Problemy endokrinologii. 2022;68(4):92-101. |
| 168 | Tiulpakov MA, Bezlepkina OB, Nagaeva EV, Azizian VN, Lapshina AM. [A clinical Case and brief literature review of Icenko-Cushing's Disease in a pediatric patient with atypical onset of the disease]. Problemy endokrinologii. 2022;68(4):92-101. |
| 169 | Tirgar F, Azizi Z, Hosseindoost S, Hadjighassem M. Preclinical gene therapy in glioblastoma multiforme: Using olfactory ensheathing cells containing a suicide gene. Life Sciences. 2022;311. doi: 10.1016/j.lfs.2022.121132. |
| 170 | Ting H, Liu M. Multimodal Transformer of Incomplete MRI Data for Brain Tumor Segmentation. IEEE journal of biomedical and health informatics. 2024;28(1):89-99. doi: 10.1109/JBHI.2023.3286689. |
| 171 | Timmer M, Kauke M, Saf A, Krischek B, Goldbrunner R. Semiautomatic image segmentation based volume approximation of intracranial meningiomas. Neuro-oncology. 2018;20:iii321. doi: 10.1093/neuonc/noy139. |
| 172 | Timme M, Thomas C, Spille DC, Stummer W, Ebel H, Ewelt C, et al. Brain invasion in meningiomas: does surgical sampling impact specimen characteristics and histology? Neurosurgical review. 2020;43(2):793-800. Epub 2019/06/05. doi: 10.1007/s10143-019-01125-0. PubMed PMID: 31161444. |
| 173 | Thomsen H, Steffensen E, Larsson EM. Perfusion MRI (dynamic susceptibility contrast imaging) with different measurement approaches for the evaluation of blood flow and blood volume in human gliomas. Acta radiologica (Stockholm, Sweden : 1987). 2012;53(1):95-101. |
| 174 | Thompson CA, Novotny P, Sloan JA, Bartz A, Yost K. Association between patient-reported outcomes and physical activity measured on the apple watch in patients with hematological malignancies. Blood. 2017;130. |
| 175 | Thommen R, Kazim SF, Rumalla K, Kassicieh AJ, Kalokoti P, Schmidt MH, et al. Preoperative frailty measured by risk analysis index predicts complications and poor discharge outcomes after Brain Tumor Resection in a large multi-center analysis. Journal of neuro-oncology. 2022;160(2):285-97. |
| 176 | Thomas-Teinturier C, Oliver-Petit I, Pacquement H, Oberlin O, Munzer M, De Vathaire F. Influence of growth hormone therapy in the occurrence of second neoplasm in survivors of childhood cancer. Hormone research in paediatrics. 2017;88:61. doi: 10.1159/000481424. |
| 177 | Thomas R, Caravati EM, Plumb J. Two cases of pediatric iatrogenic sodium intoxication. Clinical Toxicology. 2013;51(7):633-4. doi: 10.3109/15563650.2013.817658. |
| 178 | Theriault BC, Pazniokas J, Adkoli AS, Cho EK, Rao N, Schmidt M, et al. Frailty predicts worse outcomes after intracranial meningioma surgery irrespective of existing prognostic factors. Neurosurgical focus. 2020;49(4):E16.. |
| 179 | Theakstone AG, Brennan PM, Jenkinson MD, Goodacre R, Baker MJ. Investigating centrifugal filtration of serum-based FTIR spectroscopy for the stratification of brain tumours. PloS one. 2023;18(2):e0279669. |
| 180 | Theakstone AG, Brennan PM, Jenkinson MD, Goodacre R, Baker MJ. Centrifugal filtration of serum for FTIR spectroscopy does not improve stratification of brain tumours. 2022. |
| 181 | Thawani JP, Amirshaghaghi A, Yan L, Stein JM, Liu J, Tsourkas A. Photoacoustic-Guided Surgery with Indocyanine Green-Coated Superparamagnetic Iron Oxide Nanoparticle Clusters. Small (Weinheim an der Bergstrasse, Germany). 2017;13(37). |
| 182 | Thao BP, Dung VC, Khanh NN, Ngoc CTB, Hoan NT. Clinical presentation and etiology of central precocious puberty in Vietnamese children. Hormone research in paediatrics. 2013;80:334. |
| 183 | Thai Hao Phan TH. A case of arrhythmogenic right ventricular cardiomyopathy. European Journal of Heart Failure. 2019;21:159. doi: 10.1002/ejhf.1488. |
| 184 | Tettamanti G, Ljung R, Mathiesen T, Schwartzbaum J, Feychting M. Birth Size Characteristics and Risk of Brain Tumors in Early Adulthood: Results from a Swedish Cohort Study. Cancer epidemiology, biomarkers & prevention : a publication of the American Association for Cancer Research, cosponsored by the American Society of Preventive Oncology. 2016;25(4):678-85. |
| 185 | Terzian CN, Gamba J, Senerchia A, Brandáo LR, Petrilli AS. A successful implementation of a pediatric thrombosis program to promote assistance to children, adolescent and young adults with cancer. Journal of Thrombosis and Haemostasis. 2013;11:619-20. |
| 186 | Terzi DS, Azginoglu N. In-Domain Transfer Learning Strategy for Tumor Detection on Brain MRI. Diagnostics. 2023;13(12). doi: 10.3390/diagnostics13122110. |
| 187 | Terada T. Small cell carcinoma of the brain without extracranial involvement by serial CT, MRI and PET. International journal of clinical and experimental pathology. 2010;3(3):323-7. Epub 2010/03/13. PubMed PMID: 20224732; PubMed Central PMCID: PMC2836511. |
| 188 | Terada T. Occult very small lung carcinoma with a solitary brain metastasis that is clinically diagnosed as cavernous hemangioma: A case report. Cases Journal. 2009;2(8). doi: 10.4076/1757-1626-2-7475. |
| 189 | Teber B, Elli M, Bayram N, Yaman Y, Ünal D, Doǧan MS, et al. Second Primer Tumor after Burkitt Lymphoma: Medullablastoma. Pediatric Blood and Cancer. 2021;68(SUPPL 5). doi: 10.1002/pbc.29349. |
| 190 | Tavakoli-Sabour S. MRI Negative Meningioma Causing Isolated Abducens Nerve Palsy. Journal of Neurological Surgery, Part B: Skull Base. 2024;85. doi: 10.1055/s-0044-1780197. |
| 191 | Tatsuno S, Hata Y, Tada S. [Double-dose Gd-DTPA: detectability of intraparenchymal brain metastasis]. Nihon Igaku Hoshasen Gakkai zasshi Nippon acta radiologica. 1996;56(12):855-9. Epub 1996/10/01. PubMed PMID: 8940816. |
| 192 | Tao C, Gu D, Huang R, Zhou L, Hu Z, Chen Y, et al. Hippocampus segmentation after brain tumor resection via postoperative region synthesis. BMC medical imaging. 2023;23(1):142. |
| 193 | Tanriverdi M, Mutluay FK, Cakir FB. The effects of fatigue on daily living activities and quality of life in children with brain tumor. Neuro-oncology. 2018;20:i161-i2. doi: 10.1093/neuonc/noy059. |
| 194 | Tankala S, Pavani G, Biswal B, Siddartha G, Sahu G, Subrahmanyam NB, et al. A novel depth search based light weight CAR network for the segmentation of brain tumour from MR images. Neuroscience Informatics. 2022;2(4). doi: 10.1016/j.neuri.2022.100105. |
| 195 | Tamminga RY, Kamps WA, Humphrey GB, Drayer NM. Anthropometric measurements at diagnosis of childhood cancer. Pediatric hematology and oncology. 1990;7(3):243-51. Epub 1990/01/01. doi: 10.3109/08880019009033399. PubMed PMID: 2206866. |
| 196 | Takken T, Timmons BW, Van Mil E, De Vroede M, Helders PJM. Feasibility of fitness testing in children treated for suprapituitary brain tumors: A pilot study. Rehabilitation Oncology. 2009;27(3):3-6. doi: 10.1097/01893697-200927030-00001. |
| 197 | Takamasu T, Wada M, Taguchi M, Goto H, Taniguchi H. Effects of intervention of nutrition project team for childhood cancer. Clinical Nutrition. 2016;35:S236-S7. |
| 198 | Taj-Aldean KAH. Role of diffusion–Weight MRI in differential diagnosis of cerebral cystic lesions: A prospective study. International Journal of Pharmaceutical and Clinical Research. 2017;9(1):1-5. |
| 199 | Taheri E, Raeeszadeh-Sarmazdeh M. Effect of TIMPs and Their Minimally Engineered Variants in Blocking Invasion and Migration of Brain Cancer Cells. 2024. |
| 200 | Tabouret E, Rousseau B, Barrie M, Thiebault L, Chinot O, Hulin A. Bevacizumab residual concentration for recurrent glioblastoma patients. Fundamental and Clinical Pharmacology. 2016;30:26. doi: 10.1111/fcp.12189. |
| 201 | Tabori U, Sung L, Hukin J, Laperriere N, Crooks B, Carret AS, et al. Medulloblastoma in the second decade of life: a specific group with respect to toxicity and management: a Canadian Pediatric Brain Tumor Consortium Study. Cancer. 2005;103(9):1874-80. |
| 202 | Syed S, Karambizi DI, Baker A, Groh DM, Toms SA. A Comparative Report on Intracranial Tumor-to-Tumor Metastasis and Collision Tumors. World neurosurgery. 2018;116:454-63.e2. Epub 2018/04/29. doi: 10.1016/j.wneu.2018.04.109. PubMed PMID: 29704691. |
| 203 | Sureshkumar M, Pandian A. Accessing the blood-brain barrier to treat brain disorders. Current Nanomedicine. 2019;9(3):198-209. doi: 10.2174/2468187309666190823154318. |
| 204 | Sun M, Uy B, Imbroane M, Peeters S, McCormick J, Zhang H, et al. Comparison of Changes in Quality of Life after Endoscopic Endonasal Approach for Craniopharyngiomas versus Anterior Skull Base Meningiomas. Journal of Neurological Surgery, Part B Skull Base. 2022;83(SUPPL 1). doi: 10.1055/s-0042-1743779. |
| 205 | Sukarova-Angelovska E, Kocova M, Angelkova N, Joseva J. Impaired growth hormone secretion associated with large hypothalamic hamartoma. Hormone research in paediatrics. 2016;86:437. doi: 10.1159/000449142. |
| 206 | Subramanian V, Krishnakumar B, Vijayalakshmi. Spontaneously regressing brainstem glioma in a child: A case report. Annals of Indian Academy of Neurology. 2017;20(SUPPL 2):S83. |
| 207 | Šturm D. Effects of fraxiparine therapy and D-dimer levels during treatment of deep vein thrombosis and thromboembolism in cancer patients. Libri Oncologici. 2004;32(1-3):33-8. |
| 208 | Stromberg K, Hudgins WR, Dorman LS, Henderson LE, Sowder RC, Sherrell BJ, et al. Human brain tumor-associated urinary high molecular weight transforming growth factor: a high molecular weight form of epidermal growth factor. Cancer research. 1987;47(4):1190-6. |
| 209 | Strobel K, Simpson P, Donohoue PA, Firat S, Jogal S. Impact of Age at Diagnosis and Hypothalamic Involvement on Body Mass Index Z-Score Change in Pediatric Brain Tumor Survivors. Hormone research in paediatrics. 2016;85(6):389-95. doi: 10.1159/000445890. PubMed PMID: 27255491. |
| 210 | Strobel K, Simpson P, Donohoue P, Firat S, Jogal S. Impact of age at diagnosis on obesity in pediatric brain tumor survivors. Pediatric Blood and Cancer. 2014;61:S184. doi: 10.1002/pbc.25314. |
| 211 | Stortz E, Turin C, Lillehei K, Catel C, Damek D, Wierman M. Mucosal-Associated Lymphoid Tissue Lymphoma: a Rare Condition in the Differential Diagnosis of a Parasellar Mass. Journal of the Endocrine Society. 2022;6:A501. doi: 10.1210/jendso/bvac150.1042. |
| 212 | Sterzing F, Schubert K, Sroka-Perez G, Kalz J, Debus J, Herfarth K. Helical tomotherapy. Experiences of the first 150 patients in Heidelberg. Strahlentherapie und Onkologie : Organ der Deutschen Rontgengesellschaft [et al]. 2008;184(1):8-14. |
| 213 | Stern E, Ami MB, Caspi S, Lurye M, Abebe-Campino G, Yalon M, et al. Growth characteristics and final height in survivors of childhood medulloblastoma. Hormone research in paediatrics. 2023;96:297. doi: 10.1159/000533803. |
| 214 | Steele CB, Thomas CC, Henley SJ, Massetti GM, Galuska DA, Agurs-Collins T, et al. Vital Signs: Trends in Incidence of Cancers Associated with Overweight and Obesity - United States, 2005-2014. MMWR Morbidity and mortality weekly report. 2017;66(39):1052-8. |
| 215 | Starr HJ. PSEUDOTUMOR CEREBRI. REPORT OF SIX CASES COMPARED WITH ONE CASE OF PSEUDOPAPILLEDEMA. The Medical annals of the District of Columbia. 1964;33:357-62. Epub 1964/08/01. PubMed PMID: 14180782. |
| 216 | Sriram R, Ali-Osman F. S1-nucleases enhancement of the ethidium bromide binding assay of drug-induced DNA interstrand crosslinking in human brain tumor cells. Analytical biochemistry. 1990;187(2):345-8. doi: 10.1016/0003-2697(90)90467-N. PubMed Central PMCID: Bristol(United States). |
| 217 | Spirollari E, Vazquez S, Das A, Wang R, Ampie L, Carpenter AB, et al. Characteristics of Patients Selected for Surgical Treatment of Spinal Meningioma. World neurosurgery. 2022;165:e680-e8. Epub 2022/07/03. doi: 10.1016/j.wneu.2022.06.121. PubMed PMID: 35779754. |
| 218 | Spínola A, Ferreira S, Amado F, Lopes S, Roncon S. Hematopoietic progenitor cells collection in pediatric patients with brain tumor. Journal of clinical apheresis. 2016;31(1):22-8. Epub 2015/04/09. doi: 10.1002/jca.21398. PubMed PMID: 25851934. |
| 219 | Spanakis M, Oraiopoulou ME, Tzamali E, Sakkalis V, Maris TG, Papadaki E, et al. An in silico estimation of the pharmacokinetic profile and the disposition of Gd-DTPA in brain tumor lesions of different vasculature through PBPK models. Neuro-oncology. 2014;16:ii84. doi: 10.1093/neuonc/nou174. |
| 220 | Soto JM, Nguyen AV, van Zyl JS, Huang JH. Outcomes After Supratentorial Craniotomy for Primary Malignant Brain Tumor Resection in Adult Patients: A National Surgical Quality Improvement Program Analysis. World neurosurgery. 2023;175:e780-e9. |
| 221 | Song K, Wang J, Chai X, Zhang YM, Yan CY, Zhou JB, et al. Meningioma associated with reactive hyperplasia and colonization of melanocytes. Chinese Journal of Contemporary Neurology and Neurosurgery. 2021;21(6):485-92. doi: 10.3969/j.issn.1672-6731.2021.06.011. |
| 222 | Son T, Oliva C, Ali MY, Flor S, Griguer C. Copper Toxicity and Chemoresistance in Malignant Brain Tumor. Free Radical Biology and Medicine. 2019;145:S77. doi: 10.1016/j.freeradbiomed.2019.10.204. |
| 223 | Soltani S, Shayanfar M, Benisi-Kohansal S, Mohammad-Shirazi M, Sharifi G, Djazayeri A, et al. Adherence to the MIND diet in relation to glioma: a case-control study. Nutritional neuroscience. 2022;25(4):771-8. Epub 2020/08/26. doi: 10.1080/1028415x.2020.1809876. PubMed PMID: 32840185. |
| 224 | Sollmann N, Bulubas L, Tanigawa N, Zimmer C, Meyer B, Krieg SM. The variability of motor evoked potential latencies in neurosurgical motor mapping by preoperative navigated transcranial magnetic stimulation. BMC neuroscience. 2017;18(1):5. |
| 225 | Soliman HM, ElBeheiry AA, Abdel-Kerim AA, Farhoud AH, Reda MI. Recurrent brain tumor versus radiation necrosis; can dynamic susceptibility contrast (DSC) perfusion magnetic resonance imaging differentiate? Egyptian Journal of Radiology and Nuclear Medicine. 2018;49(3):719-26. |
| 226 | Soldos P, Besenyi Z, Hideghéty K, Pávics L, Hegedűs Á, Rácz L, et al. Comparison of Shear Wave Elastography and Dynamometer Test in Muscle Tissue Characterization for Potential Medical and Sport Application. Pathology oncology research : POR. 2021;27:1609798. |
| 227 | Soldado Rodriguez L, Valverde Barea M, Ruiz Martínez GM. First psychotic episode and incidental meningioma. European Psychiatry. 2020;63:S539. doi: 10.1192/j.eurpsy.2020.6. |
| 228 | Sodero G, Agresti P, Triarico S, Romano A, Mastrangelo S, Attinà G, et al. Growth hormone replacement therapy in pediatric brain tumor survivors. Minerva pediatrics. 2022;74(3):340-8. Epub 2022/02/11. doi: 10.23736/s2724-5276.22.06799-4. PubMed PMID: 35142454. |
| 229 | Sobstyl M, Nagańska E, Glinka P, Wierzba-Bobrowicz T, Acewicz A, Kuls-Oszmaniec A. Large haemorrhage within glioblastoma mimicking haemorrhagic stroke and coexistance of meningioma: a case of collision tumours. Folia neuropathologica. 2023;61(4):433-41. |
| 230 | Snyder MC, Terry BN, Jaishankar GB. A bitter pillto swallow. Journal of Investigative Medicine. 2011;59(2):380. doi: 10.231/JIM.0b013e31820bab4c. |
| 231 | Snurdel M, Batista A, Kirkpatrick N, Almodovar C, Riedemann L, Peterson T, et al. Targeting placental growth factor/ neuropilin-1 pathway inhibits growth and spread of medulloblastoma. Cancer research. 2014;74(20). doi: 10.1158/1538-7445.PEDCAN-IA17. |
| 232 | Snuderl M, Batista A, Kirkpatrick N, De Almodovar CR, Riedemann L, Knevels E, et al. Placental growth factor/neuropilin 1 signaling is a therapeutic target in pediatric medulloblastoma. Neuro-oncology. 2013;15:i19-i20. doi: 10.1093/neuonc/not047. |
| 233 | Smulevich VB, Solionova LG, Belyakova SV. Parental occupation and other factors and cancer risk in children: I. Study methodology and non-occupational factors. International journal of cancer. 1999;83(6):712-7. |
| 234 | Skjøth-Rasmussen J, Bøgeskov L, Sehested A, Klausen C, Broholm H, Nysom K. The use of 5-ALA to assist complete removal of residual non-enhancing part of childhood medulloblastoma: a case report. Child's nervous system : ChNS : official journal of the International Society for Pediatric Neurosurgery. 2015;31(11):2173-7. |
| 235 | Sjåvik K, Bartek J, Jr., Solheim O, Ingebrigtsen T, Gulati S, Sagberg LM, et al. Venous Thromboembolism Prophylaxis in Meningioma Surgery: A Population-Based Comparative Effectiveness Study of Routine Mechanical Prophylaxis with or without Preoperative Low-Molecular-Weight Heparin. World neurosurgery. 2016;88:320-6.. |
| 236 | Siviero-Miachon AA, de Castro Monteiro CM, Pires LV, Rozalem AC, da Silva NS, Petrilli AS, et al. Early traits of metabolic syndrome in pediatric post-cancer survivors: Outcomes in adolescents and young adults treated for childhood medulloblastoma. Arquivos brasileiros de endocrinologia e metabologia. 2011;55(8):653-60. |
| 237 | Sitlinger A, Thompson DP, Deal MA, Garcia E, Stewart T, Guadalupe E, et al. Exercise and chronic lymphocytic leukemia (CLL) - Relationships among physical activity, fitness, & inflammation, and their impacts on CLL patients. Blood. 2018;132. doi: 10.1182/blood-2018-99-117736. |
| 238 | Sirianni RW, Saltzman WM, Carson RE. Development of dPET, a PET technique to measure the diffusion of drugs after direct delivery to the brain. Journal of Cerebral Blood Flow and Metabolism. 2009;29:S366-S7. doi: 10.1038/jcbfm.2009.150. |
| 239 | Singh B, Hadley S, Calimag A, Yasmeen T. Abstract #1407012: ACTH Producing Pituitary Adenoma Found After Transsphenoidal Resection of Meningioma. Endocrine Practice. 2023;29(5):S79. doi: 10.1016/j.eprac.2023.03.178. |
| 240 | Simonetti G, Gammone M, Bersani A, Garofalo R, Anghileri E, Botturi A, et al. OBSERVATIONAL, PROSPECTIVE MULTICENTER STUDY ON THE ROLE OF BODY MASS INDEX (BMI) IN THE DEVELOPMENT OF THROMBOTIC COMPLICATIONS IN CANCER PATIENTS WITH PICC: PRELIMINARY RESULTS. Neuro-oncology. 2022;24:ii55-ii6. doi: 10.1093/neuonc/noac174. |
| 241 | Silva V, Stahelin L, Barbato M, Sens M, Weber T. Could multiple acrochordons be a sign of glioblastoma multiforme? Journal of the American Academy of Dermatology. 2012;66(4):AB128. doi: 10.1016/j.jaad.2011.11.541. |
| 242 | Silbergeld DL, Ali-Osman F, Winn HR. Induction of transformational changes in normal endothelial cells by cultured human astrocytoma cells. Journal of neurosurgery. 1991;75(4):604-12. Epub 1991/10/01. doi: 10.3171/jns.1991.75.4.0604. PubMed PMID: 1885978. |
| 243 | Siekmann M, Lothes T, König R, Wirtz CR, Coburger J. Experimental study of sector and linear array ultrasound accuracy and the influence of navigated 3D-reconstruction as compared to MRI in a brain tumor model. International journal of computer assisted radiology and surgery. 2018;13(3):471-8. |
| 244 | Shoshan Y, Siegal T. Control of vasogenic edema in a brain tumor model: comparison between dexamethasone and superoxide dismutase. Neurosurgery. 1996;39(6):1206-13; discussion 13-4. Epub 1996/12/01. doi: 10.1097/00006123-199612000-00025. PubMed PMID: 8938776. |
| 245 | Shokhirev MN, Torosin NS, Kramer DJ, Johnson AA, Cuellar TL. CheekAge: a next-generation buccal epigenetic aging clock associated with lifestyle and health. GeroScience. 2024;46(3):3429-43. Epub 2024/03/05. doi: 10.1007/s11357-024-01094-3. |
| 246 | Shiuan EF, Sun L, Reynoso J, Li S, Li G, Kim J, et al. Immune landscape of resected brain metastases in patients treated with and without immune checkpoint blockade immunotherapy. Cancer research. 2023;83(2). doi: 10.1158/1538-7445.METASTASIS22-B032. |
| 247 | Shimony N, Amit U, Minz B, Grossman R, Dany MA, Gonen L, et al. Perioperative pregabalin for reducing pain, analgesic consumption, and anxiety and enhancing sleep quality in elective neurosurgical patients: a prospective, randomized, double-blind, and controlled clinical study. Journal of neurosurgery. 2016;125(6):1513-22. |
| 248 | Shimizu M, Shimizu A, Takamasu T, Goto H, Taniguchi H. Effectiveness of Nutrition Support Team Intervention in Pediatric Patients with Cancer. Journal of nutritional science and vitaminology. 2024;70(4):328-35. |
| 249 | Shimazaki S, Kazukawa I, Mori K, Kihara M, Minagawa M. Factors predicting endocrine late effects in childhood cancer survivors from a Japanese hospital. Endocrine journal. 2020;67(2):131-40. doi: 10.1507/endocrj.EJ19-0228. PubMed PMID: 31656261. |
| 250 | Shi S, Cheng J, Chen H, Zhang Y, Zhao Y, Wang B. Preoperative and intraoperative predictors of deep venous thrombosis in adult patients undergoing craniotomy for brain tumors: A Chinese single-center, retrospective study. Thrombosis research. 2020;196:245-50. |
| 251 | Shi J, Yu L, Cheng Q, Yang X, Cheng KT, Yan Z. MFTrans: Modality-Masked Fusion Transformer for Incomplete Multi-Modality Brain Tumor Segmentation. IEEE journal of biomedical and health informatics. 2024;28(1):379-90. doi: 10.1109/JBHI.2023.3326151. |
| 252 | Shew M, Kavookjian H, Dahlstrom K, Muelleman T, Lin J, Camarata P, et al. Incidence and Risk Factors for Sigmoid Venous Thrombosis Following CPA Tumor Resection. Otology & neurotology : official publication of the American Otological Society, American Neurotology Society [and] European Academy of Otology and Neurotology. 2018;39(5):e376-e80. |
| 253 | Sherwood PR, Price TJ, Weimer J, Ren D, Donovan HS, Given CW, et al. Neuro-oncology family caregivers are at risk for systemic inflammation. Journal of neuro-oncology. 2016;128(1):109-18. Epub 2016/02/26. doi: 10.1007/s11060-016-2083-3. |
| 254 | Shen V, Johnson C, Schenk J, Pathare J. The use of complementary and alternative medicine (CAM) in children with brain tumors. Pediatric Blood and Cancer. 2015;62:S105. doi: 10.1002/pbc.25540. |
| 255 | Shen C, Wang X, Zheng Z, Gao C, Chen X, Zhao S, et al. Doxorubicin and indocyanine green loaded superparamagnetic iron oxide nanoparticles with PEGylated phospholipid coating for magnetic resonance with fluorescence imaging and chemotherapy of glioma. International journal of nanomedicine. 2019;14:101-17. |
| 256 | Shelatkar T, Urvashi D, Shorfuzzaman M, Alsufyani A, Lakshmanna K. Diagnosis of Brain Tumor Using Light Weight Deep Learning Model with Fine-Tuning Approach. Computational and mathematical methods in medicine. 2022;2022:2858845. doi: 10.1155/2022/2858845. |
| 257 | Sharif T, Martell E, Dai C, Ghassemi-Rad MS, Lee K, Singh SK, et al. Phosphoglycerate dehydrogenase inhibition induces p-mTOR-independent autophagy and promotes multilineage differentiation in embryonal carcinoma stem-like cells. Cell death & disease. 2018;9(10):990. |
| 258 | Sharif T, Dai C, Martell E, Ghassemi-Rad MS, Hanes MR, Murphy PJ, et al. TAp73 Modifies Metabolism and Positively Regulates Growth of Cancer Stem-Like Cells in a Redox-Sensitive Manner. Clinical cancer research : an official journal of the American Association for Cancer Research. 2019;25(6):2001-17. |
| 259 | Shalitin S, Gal M, Goshen Y, Cohen I, Yaniv I, Phillip M. Endocrine outcome in long-term survivors of childhood brain tumors. Hormone research in paediatrics. 2011;76(2):113-22. Epub 2011/06/22. doi: 10.1159/000327584. PubMed PMID: 21691048. |
| 260 | Shahrestani MA, Saneei P, Shayanfar M, Mohammad-Shirazi M, Sharifi G, Sadeghi O, et al. The relationship between rice consumption and glioma: a case-control study in adults. Scientific reports. 2021;11(1):6073. |
| 261 | Shahid S, Chaudary MA. Association of 25-hydroxyvitamin D with hematological profile and anthropometry in patients with glioma. Revista da Associacao Medica Brasileira (1992). 2022;68(11):1547-52. |
| 262 | Shahi MH, Rey JA, Castresana JS. The sonic hedgehog-GLI1 signaling pathway in brain tumor development. Expert opinion on therapeutic targets. 2012;16(12):1227-38. Epub 2012/09/21. doi: 10.1517/14728222.2012.720975. PubMed PMID: 22992192. |
| 263 | Shah RJ, Patel J, Belany PA. A Case Of Papillary Craniopharyngioma WHO Grade 1 Turned Into Abscess. Journal of the Endocrine Society. 2023;7:A633-A4. doi: 10.1210/jendso/bvad114.1182. |
| 264 | Shabo E, Wach J, Hamed M, Güresir Á, Weinhold L, Vatter H, et al. Asymptomatic Postoperative Cerebral Venous Sinus Thrombosis After Posterior Fossa Tumor Surgery: Incidence, Risk Factors, and Therapeutic Options. Neurosurgery. 2023;92(6):1171-6. |
| 265 | Sescu D, Chansiriwongs A, Minta KJ, Vasudevan J, Kaliaperumal C. Early Preventive Strategies and CNS Meningioma - Is This Feasible? A Comprehensive Review of the Literature. World neurosurgery. 2023;180:123-33.. |
| 266 | Seo GH, Choi JH, Kim YM, Koh KN, Im HJ, Ra YS, et al. Long-term endocrine effects and trends in body mass index changes in patients with childhood-onset brain tumors. Journal of neuro-oncology. 2018;138(1):55-62. |
| 267 | Sengupta S, Nandi S, Hindi ES, Wainwright DA, Han Y, Lesniak MS. Short hairpin RNA-mediated fibronectin knockdown delays tumor growth in a mouse glioma model. Neoplasia (New York, NY). 2010;12(10):837-47. |
| 268 | Senders JT, Muskens IS, Cote DJ, Goldhaber NH, Dawood HY, Gormley WB, et al. Thirty-Day Outcomes After Craniotomy for Primary Malignant Brain Tumors: A National Surgical Quality Improvement Program Analysis. Neurosurgery. 2018;83(6):1249-59. |
| 269 | Senders JT, Goldhaber NH, Cote DJ, Muskens IS, Dawood HY, De Vos F, et al. Venous thromboembolism and intracranial hemorrhage after craniotomy for primary malignant brain tumors: a National Surgical Quality Improvement Program analysis. Journal of neuro-oncology. 2018;136(1):135-45. |
| 270 | Sen R, Benjamin CG, Golfinos JG, Sen C, Roland JT, Jethanamest D, et al. Post-operative sinus thrombosis in the setting of skull base and parasagittal surgery. Journal of Neurological Surgery Part B: Skull Base. 2017;78. doi: 10.1055/s-0037-1600693. |
| 271 | Selvi T K, Sumaiya Begum A, Poonkuzhali P, Aarthi R. Brain tumor classification for MRI images using dual-discriminator conditional generative adversarial network. Electromagnetic biology and medicine. 2024;43(1-2):81-94. doi: 10.1080/15368378.2024.2321352. |
| 272 | Sekine A. Characteristics of metastatic brain tumor in patients with lung adenocarcinoma with mutation of epidermal growth factor receptor gene. American Journal of Respiratory and Critical Care Medicine. 2010;181(1). |
| 273 | Seipel K, Messerli C, Wiedemann G, Bacher U, Pabst T. MN1, FOXP1 and hsa-miR-181a-5p as prognostic markers in acute myeloid leukemia patients treated with intensive induction chemotherapy and autologous stem cell transplantation. Leukemia research. 2020;89:106296. |
| 274 | Seidlits SK, Sohrabi A, Lefebvre A, Condro M, Kornblum HI, Digman MA. Effects Of Matrix Stiffness On Glioblastoma Metabolism. Tissue Engineering - Part A. 2022;28:85-6. doi: 10.1089/ten.tea.2022.29030.abstracts. |
| 275 | Seetha J, Raja SS. Brain tumor classification using Convolutional Neural Networks. Biomedical and Pharmacology Journal. 2018;11(3):1457-61. doi: 10.13005/bpj/1511. |
| 276 | Schulz C, Kunz U, Mauer UM. Unilateral microsurgical approaches for the resection of intradural spinal neoplasms. European Spine Journal. 2013;22(11):2599. doi: 10.1007/s00586-013-3050-8. |
| 277 | Schultes B, Ernst B. Long-term outcomes after gastric bypass surgery in patients with craniopharyngioma-related hypothalamic obesity—Three cases with 7, 8, and 14 years follow-up. Obesity Research and Clinical Practice. 2023;17(4):361-5. doi: 10.1016/j.orcp.2023.06.004. |
| 278 | Schrell UMH, Rittig MG, Anders M, Kiesewetter F, Marschalek R, Koch UH, et al. Hydroxyurea for treatment of unresectable and recurrent meningiomas. I. Inhibition of primary human meningioma cells in culture and in meningioma transplants by induction of the apoptotic pathway. Journal of neurosurgery. 1997;86(5):845-52. |
| 279 | Schnoor R, Maas SL, Broekman ML. Heparin in malignant glioma: review of preclinical studies and clinical results. Journal of neuro-oncology. 2015;124(2):151-6. Epub 2015/07/01. doi: 10.1007/s11060-015-1826-x. PubMed PMID: 26123362; PubMed Central PMCID: PMC4582077. |
| 280 | Schneider M, Borger V, Grigutsch D, Güresir Á, Potthoff AL, Velten M, et al. Elevated body mass index facilitates early postoperative complications after surgery for intracranial meningioma. Neurosurgical review. 2021;44(2):1023-9. |
| 281 | Schneider CV, Schneider KM, Teumer A, Rudolph KL, Hartmann D, Rader DJ, et al. Association of Telomere Length With Risk of Disease and Mortality. JAMA internal medicine. 2022;182(3):291-300. Epub 2022/01/19. doi: 10.1001/jamainternmed.2021.7804. |
| 282 | Schmiegelow M, Lassen S, Weber L, Poulsen HS, Hertz H, Müller J. Dosimetry and growth hormone deficiency following cranial irradiation of childhood brain tumors. Medical and pediatric oncology. 1999;33(6):564-71. |
| 283 | Schipmann S, Spille DC, Gallus M, Lohmann S, Schwake M, Warneke N, et al. Postoperative surveillance in cranial and spinal tumor neurosurgery: when is this warranted? Journal of neurosurgery. 2023;138(5):1188-98. Epub 2022/09/18. doi: 10.3171/2022.7.Jns22691. P |
| 284 | Sawaya R, Highsmith R. Plasminogen activator activity and molecular weight patterns in human brain tumors. Journal of neurosurgery. 1988;68(1):73-9. Epub 1988/01/01. doi: 10.3171/jns.1988.68.1.0073. PubMed PMID: 3335914. |
| 285 | Savitz DA, Ananth CV. Birth characteristics of childhood cancer cases, controls, and their siblings. Pediatric hematology and oncology. 1994;11(6):587-99. Epub 1994/11/01. doi: 10.3109/08880019409141806. PubMed PMID: 7857782. |
| 286 | Sato K, Takeuchi T, Hayashi N, Miyata H, Ogasawara T, Watanabe T, et al. Anesthetic management of a dental patient with panhypopituitarism. Journal of Japanese Dental Society of Anesthesiology. 1993;21(4):777-83. |
| 287 | Sato J, Shimamura N, Naraoka M, Terui K, Asano K, Itou E, et al. Long-term tumor-free survival case of congenital embryonal tumor with various pathological components. Child's nervous system : ChNS : official journal of the International Society for Pediatric Neurosurgery. 2013;29(6):921-6. |
| 288 | Sathornsumetee S, Rich JN. New treatment strategies for malignant gliomas. Expert review of anticancer therapy. 2006;6(7):1087-104. Epub 2006/07/13. doi: 10.1586/14737140.6.7.1087. PubMed PMID: 16831080. |
| 289 | Sathornsumetee S, Rich JN. New approaches to primary brain tumor treatment. Anti-cancer drugs. 2006;17(9):1003-16. Epub 2006/09/27. doi: 10.1097/01.cad.0000231473.00030.1f. PubMed PMID: 17001172. |
| 290 | Sathornsumetee S, Reardon DA. Targeting multiple kinases in glioblastoma multiforme. Expert opinion on investigational drugs. 2009;18(3):277-92. Epub 2009/02/27. doi: 10.1517/13543780802692603. PubMed PMID: 19243279. |
| 291 | Sathies Kumar T, Arun C, Ezhumalai P. An approach for brain tumor detection using optimal feature selection and optimized deep belief network. Biomedical Signal Processing and Control. 2022;73. doi: 10.1016/j.bspc.2021.103440. |
| 292 | Satgé D, Nishi M, Sirvent N, Vekemans M, Chenard MP, Barnes A. A tumor profile in Patau syndrome (trisomy 13). American journal of medical genetics Part A. 2017;173(8):2088-96. Epub 2017/05/26. doi: 10.1002/ajmg.a.38294. PubMed PMID: 28544599. |
| 293 | Sastry RA, Pertsch NJ, Tang O, Shao B, Toms SA, Weil RJ. Frailty and outcomes after craniotomy for brain tumor. Journal of clinical neuroscience : official journal of the Neurosurgical Society of Australasia. 2020;81:95-100. Epub 2020/11/24. doi: 10.1016/j.jocn.2020.09.002. PubMed PMID: 33222979. |
| 294 | Sasaki N, Ishii T, Yasuchika K, Kamimura R, Doi R, Uemoto S. Alpha-fetoprotein can be used as a pancreatic cancer stem cell marker. Journal of Hepatology. 2010;52:S348. doi: 10.1016/S0168-8278(10)60895-1. |
| 295 | Sarvaideo JL, Kristianto J, Connelly JM, Blank RD. Endothelin-1 driving osteosclerosis and meningiomatosis in intraosseous meningioma. Endocrine Reviews. 2017;38(3). |
| 296 | Sarkar S, Throckmorton W, Bingham R, Msaouel P, Genovese G, Slopis J, et al. Renal Cell Carcinoma Unclassified with Medullary Phenotype in a Patient with Neurofibromatosis Type 2. Current oncology (Toronto, Ont). 2023;30(3):3355-65. |
| 297 | Sapkota MR, Yang Z, Zhu D, Zhang Y, Yuan T, Gao J, et al. Evaluation of Epidemiologic Factors, Radiographic Features, and Pathologic Findings for Predicting Peritumoral Brain Edema in Meningiomas. Journal of magnetic resonance imaging : JMRI. 2020;52(1):174-82. |
| 298 | Santisukwongchote S, Coire C, Duncan E, Lubelsky S, Keith J. Subcutaneous implantation of a meningioma following abdominal subcutaneous preservation of a cranial bone flap. Journal of Neuropathology and Experimental Neurology. 2020;79(6):705. doi: 10.1093/jnen/nlaa036. |
| 299 | Santiago RB, Shah N, Ali A, Ranjan S. Safety of Direct Oral Anticoagulants as Compared to Low Molecular Weight Heparin in Treating Venous Thromboembolism in Patients with Primary Brain Tumors and Brain Metastases -A Review of Literature and Meta-analysis. Neurology. 2023;100(17). doi: 10.1212/WNL.0000000000203961. |
| 300 | Sano T, Yamasaki R, Saito H, Hirose T, Kudo E, Kameyama K, et al. Growth hormone-releasing hormone (GHRH)-secreting pancreatic tumor in a patient with multiple endocrine neoplasia type I. The American journal of surgical pathology. 1987;11(10):810-9. |
| 301 | Sangtongjaraskul S, Sae-Phua V, Amornfa J, Tuchinda L. Risk factors of intraoperative blood transfusion in pediatric craniotomy for intracranial tumor resection: a 10-year analysis. Journal of neurosurgery Pediatrics. 2023;32(1):115-23. |
| 302 | Sanghez V, Chen M, Li S, Chou TF, Iacovino M, Lin HJ, et al. Efficacy of Asparaginase Erwinia chrysanthemi With and Without Temozolomide Against Glioma Cells and Intracranial Mouse Medulloblastoma. Anticancer research. 2018;38(5):2627-34. |
| 303 | Sane S, Sinaei B, Golabi P, Talebi H, Rahmani N, Foruhar R, et al. The Neurologic Complications Associated with Anesthesia in Pediatrics Treated with Radiotherapy Under Anesthesia. Iranian Journal of Pediatrics. 2022;32(1). doi: 10.5812/IJP.116822. |
| 304 | Sandhiya B, Kanaga Suba Raja S, Shruthi K, Praveena Rachel Kamala S. Brain tumour segmentation and classification with reconstructed MRI using DCGAN. Biomedical Signal Processing and Control. 2024;92. doi: 10.1016/j.bspc.2024.106005. |
| 305 | Samuel AA, Tukel M. “MY EYE IS DOWN AND OUT” - UNUSUAL PRESENTATION OF HIGH-GRADE BURKITT LYMPHOMA AS AN OCULOMOTOR NERVE PALSY. Journal of general internal medicine. 2022;37:S548. doi: 10.1007/s11606-022-07653-8. |
| 306 | Samples JR, Robertson DM, Taylor JZ, Waller RR. Optic nerve meningioma. Ophthalmology. 1983;90(12):1591-4. Epub 1983/12/01. doi: 10.1016/s0161-6420(83)34346-3. PubMed PMID: 6677859. |
| 307 | Samanic CM, Teer JK, Thompson ZJ, Creed JH, Mokhtari S, Fridley BL, et al. Mitochondrial DNA sequence variation and risk of meningioma. Journal of neuro-oncology. 2021;155(3):319-24. Epub 2021/10/21. doi: 10.1007/s11060-021-03878-5. PubMed PMID: 34669147. |
| 308 | Samaan MC, Yousif M, Wang KW, Fleming A, Burrow S, Johnston D, et al. Adiposity and depressive symptoms in survivors of childhood brain tumors: A report from the Canadian study of the determinants of endometabolic health in children. Journal of the neurological sciences. 2017;381:746. doi: 10.1016/j.jns.2017.08.2105. |
| 309 | Samaan C, Duckworth J, Scheinemann K. Children surviving brain tumors have high rates of obesity independent of puberty. Neuro-oncology. 2012;14:i131. doi: 10.1093/neuonc/nos106. |
| 310 | Salmaggi A, Simonetti G, Trevisan E, Beecher D, Carapella CM, DiMeco F, et al. Perioperative thromboprophylaxis in patients with craniotomy for brain tumours: a systematic review. Journal of neuro-oncology. 2013;113(2):293-303. |
| 311 | Saldarriaga C, Lyssikatos C, Belyavskaya E, Keil M, Chittiboina P, Sinaii N, et al. Postoperative Diabetes Insipidus and Hyponatremia in Children after Transsphenoidal Surgery for Adrenocorticotropin Hormone and Growth Hormone Secreting Adenomas. The Journal of pediatrics. 2018;195:169-74.e1. |
| 312 | Sakai H, Yoshioka K, Yamagami K, Yamakita T, Hosoi M, Ishii T, et al. Complete adrenocorticotropin deficiency after radiation therapy for brain tumor with a normal growth hormone reserve. Internal medicine (Tokyo, Japan). 2002;41(6):453-7. |
| 313 | Sakai H, Kanki T, Fuse T, Nakamura N. Blunt injury on the craniectomized head. Report of two cases. Neurological Surgery. 1983;11(10):1093-6. |
| 314 | Sakai H, Kanki T, Fuse T, Nakamura N. [Head injuries in patients with pre-existing skull defects]. No shinkei geka Neurological surgery. 1983;11(10):1093-6. Epub 1983/10/01. PubMed PMID: 6646341. |
| 315 | Saito Y. The effects of hypothermia and steroid hormone on cerebral metabolism in cerebro-neurological diseases; experimental and clinical studies. Surgery. 1963;25(11):1121-32. |
| 316 | Saito N, Hirai N, Nagata T, Kushida N, Sato S, Hiramoto Y, et al. ANTI-TUMOR AROMATHERAPY FOR MALIGNANT BRAIN TUMORS USING VOLATILE COMPONENTS OF HIBA ESSENTIAL OIL. Neuro-oncology. 2022;24:vii217. doi: 10.1093/neuonc/noac209.833. |
| 317 | Said HM, Stein S, Hagemann C, Polat B, Staab A, Flentje M, et al. Sp1controlled gene regulation in brain tumours microenvironment. Cytopathology. 2011;22:182-3. doi: 10.1111/j.1365-2303.2011.00911.x. |
| 318 | Said HM, Polat B, Hagemann C, Staab A, Flentje M, Vordermark D. Comparative analysis of Sp1 gene regulation in an alternating oxygenating microenvironment of human brain tumors. FEBS Journal. 2010;277:134. doi: 10.1111/j.1742-4658.2010.07680.x. |
| 319 | Sahmoun AE, Case LD, Chavour S, Kareem S, Schwartz GG. Hypertension and risk of brain metastasis from small cell lung cancer: a retrospective follow-up study. Anticancer research. 2004;24(5b):3115-20. Epub 2004/10/30. PubMed PMID: 15510598. |
| 320 | Sahinturk F, Sonmez E, Borcek P, Altinors N. Does leptin have a role in the development of intracranial meningiomas? Ceska a Slovenska Neurologie a Neurochirurgie. 2019;82(2):166-70. doi: 10.14735/AMCSNN2019CSNN.EU1. |
| 321 | Saha BN, Ray N, Greiner R, Murtha A, Zhang H. Quick detection of brain tumors and edemas: a bounding box method using symmetry. Computerized medical imaging and graphics : the official journal of the Computerized Medical Imaging Society. 2012;36(2):95-107. |
| 322 | Safaee M, Sun MZ, Oh T, Aghi MK, Berger MS, McDermott MW, et al. Use of thrombin-based hemostatic matrix during meningioma resection: a potential risk factor for perioperative thromboembolic events. Clinical neurology and neurosurgery. 2014;119:116-20. |
| 323 | Rutter MM, Rose SR. Long-term endocrine sequelae of childhood cancer. Current opinion in pediatrics. 2007;19(4):480-7. Epub 2007/07/17. doi: 10.1097/MOP.0b013e3282058b56. PubMed PMID: 17630615. |
| 324 | Rutkowski R, Reszec J, Hermanowicz A, Chrzanowski R, Lyson T, Mariak Z, et al. Correlation of leptin receptor expression with BMI in differential grades of human meningiomas. Oncology Letters. 2016;11(4):2515-9. doi: 10.3892/ol.2016.4272. |
| 325 | Runco D, Wasilewski-Masker K, McCracken C, Mazewski C, Patterson B, Mertens A. Impact of tumor type and treatment intensity on weight-for-age through cancer therapy in children less than 3 years old. Pediatric Blood and Cancer. 2018;65:S199. doi: 10.1002/pbc.27057. |
| 326 | Runco D, Wasilewski-Masker K, Mazewski C, Patterson B, Mertens A. Risk factors for weight loss in infants and young children undergoing cancer treatment. Pediatric Blood and Cancer. 2019;66:S192-S3. doi: 10.1002/pbc.27713. |
| 327 | Runco D, Mertens A, Wasilewski-Masker K. Nutritional outcomes in pediatric oncology patients less than 3 years of age. Journal of Clinical and Translational Science. 2018;2:84. doi: 10.1017/cts.2018.260. |
| 328 | Rumshisky A, Ghassemi M, Naumann T, Szolovits P, Castro VM, McCoy TH, et al. Predicting early psychiatric readmission with natural language processing of narrative discharge summaries. Translational psychiatry. 2016;6(10):e921. |
| 329 | Ruggieri F, Gemma M, Calvi MR, Nicelli E, Agarossi A, Beretta L. Perioperative serum brain natriuretic peptide and cardiac troponin in elective intracranial surgery. Neurocritical care. 2012;17(3):395-400. Epub 2012/03/08. doi: 10.1007/s12028-012-9684-2. PubMed PMID: 22396190. |
| 330 | Ruckser R, Tatzreiter G, Pfisterer W, Oberhauser G, Hönigschnabel S, Aboul-Enein F, et al. Case report: Complete remission with second-line bevacizumab therapy in glioblastoma patient. Neuro-oncology. 2012;14:vi69. doi: 10.1093/neuonc/nos229. |
| 331 | Rozumenko VD. [Prevention of postoperative venous thromboembolism in patient with brain tumor]. Klinichna khirurhiia. 2003(8):50-3. Epub 2003/11/13. PubMed PMID: 14610797. |
| 332 | Rowe L, Vera E, Acquaye A, Crandon S, Shah V, Bryla C, et al. The prevalence of altered body image in patients with primary brain tumors: an understudied population. Journal of neuro-oncology. 2020;147(2):397-404. |
| 333 | Roth CL, Zenno A. Treatment of hypothalamic obesity in people with hypothalamic injury: new drugs are on the horizon. Frontiers in endocrinology. 2023;14:1256514. Epub 2023/10/02. doi: 10.3389/fendo.2023.1256514. PubMed PMID: 37780616; PubMed Central PMCID: PMC10533996. |
| 334 | Roth CL, Shoemaker AH, Gottschalk M, Miller JL, Yuan G, Chen E, et al. Interim Efficacy and Safety Analysis of Setmelanotide As a Novel Treatment for Hypothalamic Obesity. Annals of Nutrition and Metabolism. 2023;79(1):11-2. doi: 10.1159/000527413. |
| 335 | Roth C, Shoemaker A, Gottschalk M, Miller J, Yuan G, Malhotra S, et al. Impact of Setmelanotide Treatment on Reducing Hyperphagia in Pediatric and Adult Patients With Hypothalamic Obesity. Hormone research in paediatrics. 2023;96:260-2. doi: 10.1159/000531602. |
| 336 | Roth C, Shoemaker A, Gottschalk M, Miller J, Yuan G, Chen E, et al. Efficacy and Safety Analysis of Setmelanotide as a Novel Treatment for Hypothalamic Obesity. Obesity. 2022;30:125. doi: 10.1002/oby.23626. |
| 337 | Rossi EG, Pellegrini M, Diacciati S, Picchi M, Baldini E. Integrative approach with diet and complementary medicine in oncology: The experience in the hospital of lucca (Italy). Journal of Alternative and Complementary Medicine. 2014;20(5):A139-A40. doi: 10.1089/acm.2014.5373. |
| 338 | Ross BD, Mitchell SL, Merkle H, Garwood M. In vivo 31P and 2H NMR [corrected] studies of rat brain tumor pH and blood flow during acute hyperglycemia: differential effects between subcutaneous and intracerebral locations. Magnetic resonance in medicine. 1989;12(2):219-34. |
| 339 | Roskoski R, Jr. The ErbB/HER family of protein-tyrosine kinases and cancer. Pharmacological research. 2014;79:34-74. Epub 2013/11/26. doi: 10.1016/j.phrs.2013.11.002. PubMed PMID: 24269963. |
| 340 | Ronsley R, Rassekh SR, Fleming A, Empringham B, Jennings W, Portwine C, et al. High molecular weight adiponectin levels are inversely associated with adiposity in pediatric brain tumor survivors. Scientific reports. 2020;10(1):18606 |
| 341 | Romano A, Masino M, Rivetti S, Mastrangelo S, Attinà G, Maurizi P, et al. Anthropometric parameters as a tool for the prediction of metabolic and cardiovascular risk in childhood brain tumor survivors. Diabetology and Metabolic Syndrome. 2024;16(1). doi: 10.1186/s13098-024-01262-7. |
| 342 | Roka YB, Paudel G, Bidur KC, Munakomi S. The diencephalic syndrome of Russell: A case report. Journal of Nepal Paediatric Society. 2010;30(1):60-3. doi: 10.3126/jnps.v30i1.2464. |
| 343 | Rohin MAK, Ridzwan N, Jumli MN, Hadi NA, Hussin N, Zahary MN, et al. Screening Of bismillah leaf (Vernonia Amygdalina) extraction for antiproliferative activies in human glioblastoma brain cancer cell lines. Research Journal of Pharmaceutical, Biological and Chemical Sciences. 2016;7(2):1084-9. |
| 344 | Roessel I, Iersel L, Schouten-van Meeteren A, Clement S, Boot A, Claahsen-van der Grinten H, et al. Body Mass Index at Diagnosis and Hypothalamic-Pituitary Dysfunction during Follow-Up in Childhood Brain Tumor Survivors. Journal of the Endocrine Society. 2019;3. doi: 10.1210/js.2019-SAT-280. |
| 345 | Rodríguez-Aguilar EA, García-Guzmán AD, Guevara-Cruz M, Pinzón-Navarro BA, Velasco-Hidalgo L, Zapata-Tarrés M, et al. Development Of New Predcitive Equation For Resting Energy Expenditure In Pediatric Patients With Oncology Diagnosis. Clinical nutrition ESPEN. 2023;54:669. |
| 346 | Rocha NG, Gomez SA, Danielian S. Severe combined immunodeficiency. about a case. Journal of Clinical Immunology. 2017;37(1):S42. doi: 10.1007/s10875-017-0442-2. |
| 347 | Rizzo SM, Tavakol S, LindaBi W, Campia U, Schmaier A. Venous thromboembolism incidence and management following meningioma resection. Vascular Medicine. 2020;25(3):NP15-NP6. doi: 10.1177/1358863X20919191. |
| 348 | Rizzo SM, Tavakol S, Bi WL, Li S, Secemsky EA, Campia U, et al. Meningioma resection and venous thromboembolism incidence, management, and outcomes. Research and Practice in Thrombosis and Haemostasis. 2023;7(2). doi: 10.1016/j.rpth.2023.100121. |
| 349 | Rives N, Milazzo JP, Perdrix A, Bironneau A, Travers A, Macé B, et al. Feasibility and efficiency of testicular tissue preservation in pre-pubertal and post-pubertal boys: Experience at Rouen University Hospital. Human Reproduction. 2012;27. doi: 10.1093/humrep/27.s2.82. |
| 350 | Rivas AM, Nassar A, Bernet VJ, Smallridge RC. The case of a patient with anaplastic thyroid cancer: 9 years of disease-free survival, and counting. Endocrine Reviews. 2016;37(2). doi: 10.1210/endo-meetings.2016.THPTA.9.FRI-305. |
| 351 | Rivard BC, Johnson PJ, Miller AD. How signal alterations on MRI correlate to tumor size and margins in the canine brain. Veterinary Radiology and Ultrasound. 2022;63:368-9. doi: 10.1111/vru.13052. |
| 352 | Riudavets M, Arakaki N, Rushing E, Taratuto AL, Sevlever G. Central nervous system desmoplastic small round cell tumor. A case report. Journal of Neuropathology and Experimental Neurology. 2012;71(6):593. doi: 10.1097/NEN.0b013e318299610e. |
| 353 | Rinaldo L, Brown DA, Bhargav AG, Rusheen AE, Naylor RM, Gilder HE, et al. Venous thromboembolic events in patients undergoing craniotomy for tumor resection: incidence, predictors, and review of literature. Journal of neurosurgery. 2020;132(1):10-21. |
| 354 | Ricklefs F, Ricklefs T, Maire C, Da Silva AS, Wollman K, Sauvigny T, et al. Deciphering the methylation signature of circulating extracellular vesicle dna for cns tumor classification. Neuro-oncology. 2021;23(SUPPL 6):vi14. doi: 10.1093/neuonc/noab196.050. |
| 355 | Ricklefs F, Maire C, Wollmann K, Dührsen L, Fita K, Sahm F, et al. Methylation and mutation profiles in meningioma cell-derived extracellular vesicle dna reflect epigenetic and genomic alterations in original tumors. Neuro-oncology. 2021;23(SUPPL 6):vi19. doi: 10.1093/neuonc/noab196.070. |
| 356 | Ricklefs F, Maire C, Kolbe K, Holz M, Westphal M, Schuller U, et al. Genome-wide methylation profiling of glioblastoma extracellular vesicle DNA allows tumor classification. Neuro-oncology. 2019;21:vi102. doi: 10.1093/neuonc/noz175.424. |
| 357 | Ricklefs F, Maire C, Fita K, Fritzsche F, Kammler G, Westphal M, et al. PEDIATRIC TUMOR CLASSIFICATION THROUGH GENOME-WIDE METHYLATION PROFILING OF EXTRACELLULAR VESICLE DNA. Neuro-oncology. 2022;24:i184. doi: 10.1093/neuonc/noac079.689. |
| 358 | Rhines LD, Sampath P, Dolan ME, Tyler BM, Brem H, Weingart J. O6-benzylguanine potentiates the antitumor effect of locally delivered carmustine against an intracranial rat glioma. Cancer research. 2000;60(22):6307-10. Epub 2000/12/05. PubMed PMID: 11103789. |
| 359 | Rey JW, Heister P, Wirges U, Nadalin S, Breuer R, Niehues T. Organ donor with unclear primary brain tumor, a contraindication for transplantation? Case report of a one year old child. Klinische Padiatrie. 2009;221(6):390-2. Epub 2009/11/06. doi: 10.1055/s-0029-1239528. PubMed PMID: 19890795. |
| 360 | Reschke F, Knoefler R, Smitka M, Huebner A. Combination of Octreotide and Dexamphetamine to Control Weight Gain in Pediatric Patients with Hypothalamic Obesity. Journal of the Endocrine Society. 2019;3. doi: 10.1210/js.2019-SAT-254. |
| 361 | Remsen LG, Pagel MA, McCormick CI, Fiamengo SA, Sexton G, Neuwelt EA. The influence of anesthetic choice, PaCO2, and other factors on osmotic blood-brain barrier disruption in rats with brain tumor xenografts. Anesthesia and analgesia. 1999;88(3):559-67. |
| 362 | Remes TM, Arikoski PM, Lähteenmäki PM, Arola MO, Pokka TM, Riikonen VP, et al. Bone mineral density is compromised in very long-term survivors of irradiated childhood brain tumor. Acta oncologica (Stockholm, Sweden). 2018;57(5):665-74. |
| 363 | Remes T, Harila-Saari A, Suo-Palosaari M, Arikoski P, Riikonen P, Sutela A, et al. Late-complications in irradiated childhood brain tumor survivors. Neuro-oncology. 2012;14:i128. doi: 10.1093/neuonc/nos106. |
| 364 | Rehani M. Radiation hazards: Skin injuries, cataract and neoplasms. CardioVascular and Interventional Radiology. 2014;37(2):S194-S6. |
| 365 | Regelson W. RU 486: how abortion politics have impacted on a potentially useful drug of broad medical application. Perspectives in biology and medicine. 1992;35(3):330-8. Epub 1992/01/01. doi: 10.1353/pbm.1992.0026. PubMed PMID: 1501996. |
| 366 | Redgate ES, Boggs S, Grudziak A, Deutsch M. Polyamines in brain tumor therapy. Journal of neuro-oncology. 1995;25(2):167-79. Epub 1995/01/01. doi: 10.1007/bf01057761. PubMed PMID: 8543973. |
| 367 | Reddy Panyala A, Manickam B. Generative adversarial network for Multimodal Contrastive Domain Sharing based on efficient invariant feature-centric growth analysis improved brain tumor classification. Electromagnetic biology and medicine. 2024:1-15. doi: 10.1080/15368378.2024.2375266. |
| 368 | Reasoner BD, Boes AD, Geerling JC. Sustained, Effortless Weight Loss after Damage to the Left Frontoinsular Cortex: A Case Report. Case Reports in Neurology. 2023;15(1):63-8. doi: 10.1159/000529533. |
| 369 | Raygor KP, Than KD, Chou D, Mummaneni PV. Comparison of minimally invasive transspinous and open approaches for thoracolumbar intradural-extramedullary spinal tumors. Neurosurgical focus. 2015;39(2):E12. Epub 2015/08/04. doi: 10.3171/2015.5.Focus15187. PubMed PMID: 26235010. |
| 370 | Ravichandran T, Kumar R, Taywade SK. Correlation of various quantitative parameters on FDG PET-CT in primary brain tumors with histopathological grades. Indian Journal of Nuclear Medicine. 2022;37(5):S44. doi: 10.4103/0972-3919.363006. |
| 371 | Rauf A, Khan HU, Hussain M. Outcome of sellar and suprasellar brain tumors with retractorless modified subfrontal approach. Pakistan Journal of Medical and Health Sciences. 2021;15(7):1544-6. doi: 10.53350/pjmhs211571544. |
| 372 | Ratnasinghe LD, Graubard BI, Kahle L, Tangrea JA, Taylor PR, Hawk E. Aspirin use and mortality from cancer in a prospective cohort study. Anticancer research. 2004;24(5b):3177-84. Epub 2004/10/30. PubMed PMID: 15510608. |
| 373 | Rath SR, Long TM, Bear NL, Miles GCP, Bullock AM, Gottardo NG, et al. Metabolic and Psychological Impact of a Pragmatic Exercise Intervention Program in Adolescent and Young Adult Survivors of Pediatric Cancer-Related Cerebral Insult. Journal of adolescent and young adult oncology. 2018;7(3):349-57. |
| 374 | Rao MG, Ladner TR, Shuman WH, Feng R, Fifi JT, Leacy RAD, et al. Safety and efficacy of preoperative embolization of meningioma in patients with preoperative peritumoral edema. Journal of Neurological Surgery Part B: Skull Base. 2021;82(SUPPL 2). doi: 10.1055/s-0041-1725490. |
| 375 | Rao MG, Ladner TR, Shuman WH, Feng R, Fifi JT, Leacy RA, et al. Safety and efficacy of preoperative embolization of meningioma with a balloon catheter. Journal of Neurological Surgery Part B: Skull Base. 2021;82(SUPPL 2). doi: 10.1055/s-0041-1725491. |
| 376 | Ranjbarzadeh R, Zarbakhsh P, Caputo A, Tirkolaee EB, Bendechache M. Brain tumor segmentation based on optimized convolutional neural network and improved chimp optimization algorithm. Computers in biology and medicine. 2024;168:107723. |
| 377 | Ranjan S, Leung D, Ghiaseddin AP, Taylor JW, Lobbous M, Dhawan A, et al. Practical guidance for direct oral anticoagulant use in the treatment of venous thromboembolism in primary and metastatic brain tumor patients. Cancer. 2024;130(9):1577-89. |
| 378 | Ramezan NW, Hussain S, Mohamad N, Yaacob NM. Endocrine Disorders in Childhood Brain Tumour Survivors: A Single-Centre Study. Journal of the ASEAN Federation of Endocrine Societies. 2024;39(1):12-7. |
| 379 | Ramanauskienė E, Labanauskas L, Verkauskienė R, Sileikienė R. Early development of endocrine and metabolic consequences after treatment of central nervous system tumors in children. Medicina (Kaunas, Lithuania). 2014;50(5):275-80. |
| 380 | Rakhshani N, Jeffery AS, Schulte F, Barrera M, Atenafu EG, Hamilton JK. Evaluation of a comprehensive care clinic model for children with brain tumor and risk for hypothalamic obesity. Obesity (Silver Spring, Md). 2010;18(9):1768-74. |
| 381 | Rajaraman P. Hunting for the causes of meningioma-obesity is a suspect. Cancer Prevention Research. 2011;4(9):1353-5. doi: 10.1158/1940-6207.CAPR-11-0360. |
| 382 | Rahimi-Rad MH, Ilkhanizadeh B, Rezaei MK. Tumor-to-tumor metastases from lung carcinoma to soft tissue fibrous histiocytoma. Tanaffos. 2008;7(3):73-5. |
| 383 | Raghavan A, Borghei-Razavi H, Roxbury C, Sindwani R, Woodard T, Kshettry V, et al. Salvage endoscopic endonasal approach for CSF leak repair after craniotomy. Journal of Neurological Surgery, Part B: Skull Base. 2019;80. doi: 10.1055/s-0039-1679686. |
| 384 | Rafati M, Zamanfar D, Shiadeh SNR, Faramarzi F, Aarabi M, Damavandi HR. Serum vitamin D levels in girls with central precocious puberty. Pharmaceutical and Biomedical Research. 2018;4(1):8-12. doi: 10.3390/d10030101. |
| 385 | Rabinovich E, Shrotriya S, McCrae KR, Bartholomew JR, Silver BJ, Adams K, et al. Improving outcomes and reducing costs for cancer associated thrombosis using a centralized service: The cleveland clinic experience. Blood. 2015;126(23):1122. |
| 386 | Quigg TC, Haddad NG, Buchsbaum JC, Shih CS. Hypothalamic obesity syndrome: rare presentation of CNS+ B-cell lymphoblastic lymphoma. Pediatric blood & cancer. 2012;59(5):930-3. Epub 2012/01/04. doi: 10.1002/pbc.24058. PubMed PMID: 22213612; PubMed Central PMCID: PMC4020003. |
| 387 | Quentin C, Charbonneau S, Moumdjian R, Lallo A, Bouthilier A, Fournier-Gosselin MP, et al. A comparison of two doses of mannitol on brain relaxation during supratentorial brain tumor craniotomy: a randomized trial. Anesthesia and analgesia. 2013;116(4):862-8. |
| 388 | Quan J, Ma C. DNMT1-mediated regulating on FBXO32 promotes the progression of glioma cells through the regulation of SKP1 activity. Environmental toxicology. 2024;39(2):783-93. Epub 2023/10/02. doi: 10.1002/tox.23976. PubMed PMID: 37782699. |
| 389 | Purewal T, Lesniak C, Ravin A, Cheng J. A Rare Case of a Toxic Thyroid Nodule Found in a Hypothyroid Patient on Levothyroxine Therapy. Journal of the Endocrine Society. 2021;5:A915. doi: 10.1210/jendso/bvab048.1869. |
| 390 | Pulikeyil A, Arcilla C, Plotskaya N. A CASE OF ROSAI -DORFMAN DISEASE MANIFESTING AS INTRACRANIAL DURALBASED TUMORS. Journal of Hospital Medicine. 2023;18:S445. doi: 10.1002/jhm.13090. |
| 391 | Pugh MJV, Knoefel JE, Mortensen EM, Amuan ME, Berlowitz DR, Van Cott AC. New-onset epilepsy risk factors in older veterans. Journal of the American Geriatrics Society. 2009;57(2):237-42. doi: 10.1111/j.1532-5415.2008.02124.x. |
| 392 | Puget S, Beccaria K, Blauwblomme T, Zerah M, Polack M, Zenaty D, et al. Childhood craniopharyngioma: Results of a prospective study of riskadapted strategies to spare hypothalamus structures. Child's Nervous System. 2016;32(10):1973-4. doi: 10.1007/s00381-016-3209-9. |
| 393 | Pruitt AA. Treatment of medical complications in patients with brain tumors. Current Treatment Options in Neurology. 2005;7(4):323-36. doi: 10.1007/s11940-005-0042-y. |
| 394 | Prisco D, Tufano A, Cenci C, Pignatelli P, Santilli F, Di Minno G, et al. Position paper of the Italian Society of Internal Medicine (SIMI) on prophylaxis and treatment of venous thromboembolism in patients with cancer. Internal and emergency medicine. 2019;14(1):21-38. |
| 395 | Priester JI, Chang F, Rutledge J, Hsieh-Wong J. Progressive bilateral quadriceps weakness in a healthy young man: An unusual presentation of diffuse-large B-cell lymphoma. Journal of Investigative Medicine. 2020;68(1):A212. doi: 10.1136/jim-2019-WMRC.492. |
| 396 | Preusser M, Plumer S, Dirnberger E, Hainfellner JA, Mannhalter C. Fixation of brain tumor biopsy specimens with RCL2 results in well-preserved histomorphology, immunohistochemistry and nucleic acids. Brain pathology (Zurich, Switzerland). 2010;20(6):1010-20. |
| 397 | Preston-Martin S, Monroe K, Lee PJ, Bernstein L, Kelsey J, Henderson S, et al. Spinal meningiomas in women in Los Angeles County: investigation of an etiological hypothesis. Cancer epidemiology, biomarkers & prevention : a publication of the American Association for Cancer Research, cosponsored by the American Society of Preventive Oncology. 1995;4(4):333-9. |
| 398 | Prasad M, Arora B, Chinnaswamy G, Vora T, Narula G, Banavali S, et al. Nutritional status in survivors of childhood cancer: Experience from Tata Memorial Hospital, Mumbai. Indian journal of cancer. 2015;52(2):219-23. Epub 2016/02/09. doi: 10.4103/0019-509x.175814. PubMed PMID: 26853412. |
| 399 | Prah MA, Al-Gizawiy MM, Mueller WM, Cochran EJ, Hoffmann RG, Connelly JM, et al. Spatial discrimination of glioblastoma and treatment effect with histologically-validated perfusion and diffusion magnetic resonance imaging metrics. Journal of neuro-oncology. 2018;136(1):13-21. |
| 400 | Portnow J, Badie B, Liu X, Frankel P, Mi S, Chen M, et al. A pilot microdialysis study in brain tumor patients to assess changes in intracerebral cytokine levels after craniotomy and in response to treatment with a targeted anti-cancer agent. Journal of neuro-oncology. 2014;118(1):169-77. |
| 401 | Portnow J, Badie B, Liu X, Frankel P, Chen M, Synold TW. A pilot study of intracerebral microdialysis (ICMD) to assess changes in cytokine and chemokine levels in the brain before and after treatment with a targeted agent in patients with primary or metastatic brain tumors. Neuro-oncology. 2010;12:iv55. doi: 10.1093/neuonc/noq116. |
| 402 | Popovic V, Pekic S, Golubicic I, Doknic M, Dieguez C, Casanueva FF. The impact of cranial irradiation on GH responsiveness to GHRH plus GH-releasing peptide-6. The Journal of clinical endocrinology and metabolism. 2002;87(5):2095-9. |
| 403 | Poole N, Schwab J, Hageman JR. A 9-year-old male with hypertension. Pediatric annals. 2015;44(1):30. doi: 10.3928/00904481-20151226-09. |
| 404 | Pons-Escoda A, Garcia-Ruiz A, Naval-Baudin P, Grussu F, Fernandez JJS, Simo AC, et al. Voxel-level analysis of normalized DSC-PWI time-intensity curves: a potential generalizable approach and its proof of concept in discriminating glioblastoma and metastasis. European radiology. 2022;32(6):3705-15. |
| 405 | Pompili A, Cacciani L, Cattani F, Caroli F, Crecco M, Masterostefano R, et al. Intracranial meningiomas in the elderly. Minerva medica. 1997;88(6):229-36. |
| 406 | Polat M, Güzel Y, Oktem Ö. Other non-reproductive endocrine abnormalities in the survivors of childhood and adolescent cancers: Review. Turkiye Klinikleri Jinekoloji Obstetrik. 2014;24(3):179-83. |
| 407 | Pluimakers VG, van Atteveld JE, de Winter DTC, Bolier M, Fiocco M, Nievelstein RJAJ, et al. Prevalence, risk factors, and optimal way to determine overweight, obesity, and morbid obesity in the first Dutch cohort of 2338 long-term survivors of childhood cancer: a DCCSS-LATER study. European journal of endocrinology. 2023;189(5):495-507. doi: 10.1093/ejendo/lvad139. |
| 408 | Pluijm S, DenHoed M, Wilson C, Neggers S, Pieters R, Van Den Heuvel-Eibrink M. Bone mineral density deficit in adult survivors of childhood cancer: A systematic literature review. Pediatric Blood and Cancer. 2015;62:S257. doi: 10.1002/pbc.25715. |
| 409 | Plotkin S, Kumthekar P, Wen P, Barker F, Beauchamp R, Gerstner E, et al. A single arm phase ii study of the dual MTORC1/ MTORC2 inhibitor vistusertib provided for sporadic patients with grade ii-iii meningiomas that recur or progress after surgery and radiation. Neuro-oncology. 2021;23(SUPPL 6):vi72. |
| 410 | Pisapia J, Macyszyn L, Akbari H, Da X, Attiah M, Bi Y, et al. Non-invasive prediction of molecular subtype in glioblastoma using multi-parametric magnetic resonance imaging pattern analysis and machine learning. Cancer research. 2015;75(15). doi: 10.1158/1538-7445.AM2015-1494. |
| 411 | Pinheiro PS, Callahan KE, Stern MC, de Vries E. Migration from Mexico to the United States: A high-speed cancer transition. International journal of cancer. 2018;142(3):477-88. Epub 2017/09/25. doi: 10.1002/ijc.31068. PubMed PMID: 28940515; PubMed Central PMCID: PMC5754218. |
| 412 | Pietilä S, Mäkipernaa A, Sievänen H, Koivisto AM, Wigren T, Lenko HL. Obesity and metabolic changes are common in young childhood brain tumor survivors. Pediatric blood & cancer. 2009;52(7):853-9. Epub 2009/01/24. doi: 10.1002/pbc.21936. PubMed PMID: 19165891. |
| 413 | Pietilä S, Mäkipernaa A, Koivisto AM, Lenko HL. Growth and pubertal development in pediatric brain tumor survivors. Pediatric Blood and Cancer. 2015;62:S281. doi: 10.1002/pbc.25715. |
| 414 | Piaskowski S, Rieske P, Szybka M, Wozniak K, Bednarek A, Płuciennik E, et al. GADD45A and EPB41 as tumor suppressor genes in meningioma pathogenesis. Cancer genetics and cytogenetics. 2005;162(1):63-7. Epub 2005/09/15. doi: 10.1016/j.cancergencyto.2005.02.009. PubMed PMID: 16157202. |
| 415 | Philipova T, Baryawno N, Hartmann W, Pietsch T, Druid H, Johnsen JI, et al. Differential forms of p53 in medulloblastoma primary tumors, cell lines and xenografts. International journal of oncology. 2011;38(3):843-9. Epub 2010/12/25. doi: 10.3892/ijo.2010.884. PubMed PMID: 21184030. |
| 416 | Pham HN, Goldberg RJ, Pham LQ, Nguyen HL, Pham DA, Mai LTT, et al. Maternal and Perinatal Factors Associated With Childhood Brain Tumors: A Case-Control Study in Vietnam. Cancer control : journal of the Moffitt Cancer Center. 2024;31:10732748241258602. |
| 417 | Peseski AM, Ortel TL, Broadwater G, Bradbury C, Johnson MO. Treatment Complications of Venous Thromboembolism in Patients with Central Nervous System Tumors. Blood. 2023;142:4028. doi: 10.1182/blood-2023-188818. |
| 418 | Perri SR, Nalbantoglu J, Annabi B, Koty Z, Lejeune L, François M, et al. Plasminogen kringle 5-engineered glioma cells block migration of tumor-associated macrophages and suppress tumor vascularization and progression. Cancer research. 2005;65(18):8359-65. doi: 10.1158/0008-5472.CAN-05-0508. |
| 419 | Perisetti A, McGrann P, Bande D. Cerebellar gangliocytoma and facial papules with PTEN mutation: A rare association. American Journal of Gastroenterology. 2013;108:S596. doi: 10.1038/ajg.2013.271. |
| 420 | Peris R, Lilo T, Gurusinghe N. Predicting the growth of CP angle meningiomas: potential application of advances in technology. British Journal of Neurosurgery. 2023;37(3):542-3. doi: 10.1080/02688697.2022.2150378. |
| 421 | Peng Y, Sun J. The multimodal MRI brain tumor segmentation based on AD-Net. Biomedical Signal Processing and Control. 2023;80. doi: 10.1016/j.bspc.2022.104336. |
| 422 | Peng J, Boekhoff S, Eveslage M, Bison B, Sowithayasakul P, Müller HL. Nuchal skinfold thickness in pediatric brain tumor patients. Hormone research in paediatrics. 2021;94(SUPPL 1):353-4. doi: 10.1159/000518849. |
| 423 | Peng J, Boekhoff S, Eveslage M, Bison B, Sowithayasakul P, Müller HL. Body composition and nuchal skinfold thickness in pediatric brain tumor patients. Neuro-oncology. 2020;22(SUPPL 3):iii455. doi: 10.1093/neuonc/noaa222. |
| 424 | Peng B, Zhao X, Yang MS, Li LL. Intracellular transglutaminase-catalyzed polymerization and assembly for bioimaging of hypoxic neuroblastoma cells. Journal of materials chemistry B. 2019;7(37):5626-32. Epub 2019/08/31. doi: 10.1039/c9tb01227c. PubMed PMID: 31469375. |
| 425 | Pedron S, Sarkaria JN, Harley BAC. Engineered glioblastoma tumor models reveal extracellular matrix signals influence the efficacy of targeted inhibitors. Cancer research. 2021;81(5 SUPPL). doi: 10.1158/1538-7445.TME21-PO035. |
| 426 | Pedron S. Bioengineered tissue mimetic hydrogels to study brain tumor biology and screen therapeutics. Cancer research. 2021;81(13 SUPPL). doi: 10.1158/1538-7445.AM2021-NG09. |
| 427 | Pedro MT, Eissler A, Scheuerle A, Schmidberger J, Kratzer W, Wirtz CR, et al. Sodium Fluorescein as Intraoperative Visualization Tool During Peripheral Nerve Biopsies. World neurosurgery. 2020;133:e513-e21. Epub 2019/09/25. doi: 10.1016/j.wneu.2019.09.081. PubMed PMID: 31550541. |
| 428 | Pedersen ML, Aagaard P, Sørensen AS, Rasmussen G, Holsgaard-Larsen A. The impact of robot assisted modulated body weight support on overground gait kinematics in young adults with neurological disorders. Gait and Posture. 2023;106:S161-S2. doi: 10.1016/j.gaitpost.2023.07.194. |
| 429 | Pedachenko EG, Velibekov RA. [Radiation-induced intracranial meningiomas]. Likars'ka sprava. 1998(4):118-20. Epub 1998/10/24. PubMed PMID: 9784723. |
| 430 | Payen JF, Faillot T, Audibert G, Vergnes MC, Bosson JL, Lestienne B, et al. [Thromboprophylaxis in neurosurgery and head trauma]. Annales francaises d'anesthesie et de reanimation. 2005;24(8):921-7. Epub 2005/07/12. doi: 10.1016/j.annfar.2005.05.012. PubMed PMID: 16006086. |
| 431 | Pavelic M, Specht C, Timik D, Liao J, Kanekar S, Sogge S, et al. Distinguishing grade 1 meningioma from higher grade meningiomas without biopsy. International Journal of Radiation Oncology Biology Physics. 2013;87(2):S157-S8. doi: 10.1016/j.ijrobp.2013.06.406. |
| 432 | Patron LA, Yeoman H, Wilson S, Gokhale V, Suzuki T. Brain penetrant small molecule for the treatment of glioblastoma. Cancer research. 2024;84(5). doi: 10.1158/1538-7445.BRAIN23-A023. |
| 433 | Patni N, Alves C, von Schnurbein J, Wabitsch M, Tannin G, Rakheja D, et al. A Novel Syndrome of Generalized Lipodystrophy Associated With Pilocytic Astrocytoma. The Journal of clinical endocrinology and metabolism. 2015;100(10):3603-6. |
| 434 | Patil R, Inoue S, Portilla-Arias J, Konda B, Espinoza A, Ding H, et al. Multifunctional nano-bioconjugate based on poly(β-L-malic acid) for temozolomide delivery for brain tumor treatment. Cancer research. 2011;71(8). doi: 10.1158/1538-7445.AM2011-3221. |
| 435 | Patel SI, Obeid H, Matti L, Ramakrishna H, Shamoun FE. Cerebral Venous Thrombosis: Current and Newer Anticoagulant Treatment Options. The neurologist. 2015;20(5):80-8. Epub 2015/11/14. doi: 10.1097/nrl.0000000000000049. PubMed PMID: 26566039. |
| 436 | Patel NM, John ST, Murphy RK, Rosenberg CH. Venous thromboembolism after meningioma resection: A case report and literature review. PM and R. 2011;3(10):S181. doi: 10.1016/j.pmrj.2011.08.071. |
| 437 | Patel AK, Patel KK, Patel D. HCV treatment outcome in HIV coinfected patient in Western India: An observational study. Indian Journal of Gastroenterology. 2013;32(1):A134. doi: 10.1007/s12664-013-0416-0. |
| 438 | Park I, von Morze C, Lupo JM, Ardenkjaer-Larsen JH, Kadambi A, Vigneron DB, et al. Investigating tumor perfusion by hyperpolarized (13) C MRI with comparison to conventional gadolinium contrast-enhanced MRI and pathology in orthotopic human GBM xenografts. Magnetic resonance in medicine. 2017;77(2):841-7. |
| 439 | Park I, Lupo JM, Nelson SJ. Correlation of Tumor Perfusion Between Carbon-13 Imaging with Hyperpolarized Pyruvate and Dynamic Susceptibility Contrast MRI in Pre-Clinical Model of Glioblastoma. Molecular imaging and biology. 2019;21(4):626-32. |
| 440 | Parizel PM, Degryse HR, Gheuens J, Martin JJ, Van Vyve M, De La Porte C, et al. Gadolinium-DOTA enhanced MR imaging of intracranial lesions. Journal of computer assisted tomography. 1989;13(3):378-85. Epub 1989/05/01. doi: 10.1097/00004728-198905000-00002. PubMed PMID: 2723166. |
| 441 | Pareja-Peña F, Burgos-Molina AM, Sendra-Portero F, Ruiz-Gómez MJ. Evidences of the (400 MHz–3 GHz) radiofrequency electromagnetic field influence on brain tumor induction. International journal of environmental health research. 2022;32(1):121-30. doi: 10.1080/09603123.2020.1738352. |
| 442 | Paraiso WKD, Garcia-Chica J, Ariza X, Zagmutt S, Fukushima S, Garcia J, et al. Poly-ion complex micelles effectively deliver CoA-conjugated CPT1A inhibitors to modulate lipid metabolism in brain cells. Biomaterials Science. 2021;9(21):7076-91. doi: 10.1039/d1bm00689d. |
| 443 | Papalia H, Rochette E, Pereira B, Merlin E, Kanold J, Duché P. Metabolic response to exercise in childhood brain tumor survivors: A pilot controlled study. Pediatric blood & cancer. 2020;67(2):e28053. Epub 2019/10/19. doi: 10.1002/pbc.28053. PubMed PMID: 31625676. |
| 444 | Panikkath R, Nugent K, Yik Lim S, Panikkath D. A hand not under control of the mind - The alien limb. Journal of Investigative Medicine. 2014;62(2):538. doi: 10.231/JIM.0000000000000055. |
| 445 | Pang HH, Umaru B, Kumar SS, Drissi R. Determining the nuclear and cytoplasmic function of BMI-1 in diffuse intrinsic pontine glioma during M phase. Cancer research. 2024;84(6). doi: 10.1158/1538-7445.AM2024-508. |
| 446 | Panagopoulou P, Sattar S, Aquilina K, Jan W, Jacques T, Slater O. Challenges in the Diagnosis of Medulloblastoma Recurrence at an Unusual Site in a Patient With Prader-Willi Syndrome. Journal of pediatric hematology/oncology. 2020;42(5):e381-e4. |
| 447 | Panagopoulos D, Karydakis P, Noutsos G, Themistocleous M. Venous Thromboembolism Risk and Thromboprophylaxis in Pediatric Neurosurgery and Spinal Injury: Current Trends and Literature Review. Seminars in thrombosis and hemostasis. 2022;48(3):318-22. |
| 448 | Palmer JD, Francis JL, Pickard JD, Iannotti F. The efficacy and safety of aprotinin for hemostasis during intracranial surgery. Journal of neurosurgery. 2003;98(6):1208-16. Epub 2003/06/21. doi: 10.3171/jns.2003.98.6.1208. PubMed PMID: 12816266. |
| 449 | Pal S, Singh RP, Kumar A. Analysis of Hybrid Feature Optimization Techniques Based on the Classification Accuracy of Brain Tumor Regions Using Machine Learning and Further Evaluation Based on the Institute Test Data. Journal of Medical Physics. 2024;49(1):22-32. doi: 10.4103/jmp.jmp_77_23. |
| 450 | Pajecki D, Santo MA, Joaquim HD, Morita F, Riccioppo D, de Cleva R, et al. BARIATRIC SURGERY IN THE ELDERLY: RESULTS OF A MEAN FOLLOW-UP OF FIVE YEARS. Arquivos brasileiros de cirurgia digestiva : ABCD = Brazilian archives of digestive surgery. 2015;28 Suppl 1(Suppl 1):15-8. |
| 451 | Paez W, Gheewala R, McClelland S, Jaboin JJ, Thomas CR, Mitin T, et al. A multidisciplinary central nervous system clinic model for radiation oncology and neurosurgery (Radians): Three-year experience with brain and skull base lesions in a community hospital setting. Journal of Neurological Surgery, Part B Skull Base. 2020;81. doi: 10.1055/s-0040-1702380. |
| 452 | Pacione D, Lieberman S. Use of delayed staged combined transcranial and endoscopic endonasal resection of anterior skull base meningioma to reduce risk of cerebrospinal fluid leak. Journal of Neurological Surgery, Part B: Skull Base. 2019;80. doi: 10.1055/s-0039-1679673. |
| 453 | Pachow D, Andrae N, Kliese N, Angenstein F, Stork O, Wilisch-Neumann A, et al. mTORC1 inhibitors suppress meningioma growth in mouse models. Clinical cancer research : an official journal of the American Association for Cancer Research. 2013;19(5):1180-9. . |
| 454 | Ozkaya E, Moy F, Oktay K. Letrozole plus gonadotropin is superior to gonadotropin alone in fertility preservation cycles. Fertility and Sterility. 2012;98(3):S9-S10. doi: 10.1016/j.fertnstert.2012.07.034. |
| 455 | Ozen Y, Ugur M, Ozbek IC, Yalcinkaya E. Early-term outcomes of the pulmonary embolism response team. Pakistan Journal of Medical Sciences. 2022;38(8). doi: 10.12669/pjms.38.8.6541. |
| 456 | Ouyang J, Jiang Y, Deng C, Zhong Z, Lan Q. Doxorubicin Delivered via ApoE-Directed Reduction-Sensitive Polymersomes Potently Inhibit Orthotopic Human Glioblastoma Xenografts in Nude Mice. International journal of nanomedicine. 2021;16:4105-15. |
| 457 | Ouafik L, Sauze S, Boudouresque F, Chinot O, Delfino C, Fina F, et al. Neutralization of adrenomedullin inhibits the growth of human glioblastoma cell lines in vitro and suppresses tumor xenograft growth in vivo. The American journal of pathology. 2002;160(4):1279-92. |
| 458 | Ouafik L, Berenguer C, Cayol M, Astorgues-Xerri L, Bekradda M, Odore E, et al. OTX015, a novel BET-bromodomain (BET-BRD) inhibitor, is a promising anticancer agent for human glioblastoma. European Journal of Cancer. 2014;50:153. |
| 459 | Oswald E, Lenhard D, Lashuk K, Pfister S, Stancato L, Schueler J. Pharmacological characterization of pediatric brain tumor PDX models in a single mouse trial format. Cancer research. 2023;83(7). doi: 10.1158/1538-7445.AM2023-3524. |
| 460 | Oswald E, Lashuk K, Gojo J, Lenhard D, Mack N, Krausert S, et al. Establishment and characterization of pediatric brain tumor models in an orthotopic mouse model. Cancer research. 2022;82(12). doi: 10.1158/1538-7445.AM2022-1673. |
| 461 | Orthmann A, Zeisig R, Fichtner I. Design and development of nanocarrier for efficient drug delivery into the brain in vitro and in vivo. European Journal of Medical Research. 2010;15:174. |
| 462 | Onken J, Goerling U, Heinrich M, Pleissner S, Krex D, Vajkoczy P, et al. Patient Reported Outcome (PRO) Among High-Grade Glioma Patients Receiving TTFields Treatment: A Two Center Observational Study. Frontiers in Neurology. 2019;10. doi: 10.3389/fneur.2019.01026. |
| 463 | Ong Q, Hochberg FH, Cima MJ. Depot delivery of dexamethasone and cediranib for the treatment of brain tumor associated edema in an intracranial rat glioma model. Journal of Controlled Release. 2015;217:183-90. doi: 10.1016/j.jconrel.2015.08.028. PubMed Central PMCID: Selleck(United States). |
| 464 | Omura Y. High frequency vibration of worst ear lobule induced short period of rapid inhibition of any cancer activity including advanced terminal cancers, most malignant brain tumor glioblastoma as well as cancer of lung, breast & gastrointestinal cancers particularly pancreatic cancer. Acupuncture and Electro-Therapeutics Research. 2020;44(3-4):241-9. |
| 465 | Olivi A, Grossman SA, Tatter S, Barker F, Judy K, Olsen J, et al. Dose escalation of carmustine in surgically implanted polymers in patients with recurrent malignant glioma: a New Approaches to Brain Tumor Therapy CNS Consortium trial. Journal of clinical oncology : official journal of the American Society of Clinical Oncology. 2003;21(9):1845-9. |
| 466 | Olivan-Sasot P, Sopena-Novales P, Martinez-Sanchis B, Torres-Espallardo I, Yepes-Agudelo A, Agudelo-Cifuentes M, et al. Preliminary results of activity-time curves analysis in PET/CT 18F-choline in treated cerebral tumors. European Journal of Nuclear Medicine and Molecular Imaging. 2018;45:S357-S8. doi: 10.1007/s00259-018-4148-3. |
| 467 | Olbing H. [DIENCEPHALIC WEIGHT LOSS SYNDROME IN EARLY INFANCY]. Archiv fur Kinderheilkunde. 1964;170:268-74. Epub 1964/06/01. PubMed PMID: 14239598. |
| 468 | Okamura T, Ali-osman F. GSTP1 inhibition of HSP27 phosphorylation contributes to glioma drug resistance. Cancer research. 2011;71(8). doi: 10.1158/1538-7445.AM2011-703. |
| 469 | Okamura T. Management of cancer-associated thrombosis. Annals of Oncology. 2016;27:vii68. doi: 10.1093/annonc/mdw513. |
| 470 | Oishi T, Sameshima T, Totsuka T, Yamasaki T, Koizumi S, Namba H. Cosmetic and Neuroprotective Placement of Custom-Made Ultra-High-Molecular-Weight Polyethylene Cranial Plate (SKULPIO) in Single-Step Surgery: Technical Note and Case Report. World neurosurgery. 2019;130:187-91. |
| 471 | Ohnishi T, Sher PB, Posner JB, Shapiro WR. Capillary permeability factor secreted by malignant brain tumor. Role in peritumoral brain edema and possible mechanism for anti-edema effect of glucocorticoids. Journal of neurosurgery. 1990;72(2):245-51. |
| 472 | Ohara AE. Utilizing high intensity gait training (HIGT) to improve ambulation following grade iv pineoblastoma resection. Rehabilitation Oncology. 2021;39(2):E18-E9. doi: 10.1097/01.REO.0000000000000254. |
| 473 | Ogata A, Tashiro K, Abumiya T, Abe H, Matsuno Y, Nakamura K, et al. [Glioblastoma multiforme with extracranial metastases without previous surgery: demonstration of extracranial metastases by peroxidase antiperoxidase staining and clinicopathological study]. No to shinkei = Brain and nerve. 1987;39(7):679-85. |
| 474 | Oga M, Takenaga K, Sato Y, Nakajima H, Koshikawa N, Osato K, et al. Inhibition of metastatic brain tumor growth by intramuscular administration of the endostatin gene. International journal of oncology. 2003;23(1):73-9. Epub 2003/06/07. PubMed PMID: 12792778. |
| 475 | Oettel M, Mukhopadhyay AK. Progesterone: the forgotten hormone in men? The aging male : the official journal of the International Society for the Study of the Aging Male. 2004;7(3):236-57. Epub 2005/01/27. doi: 10.1080/13685530400004199. PubMed PMID: 15669543. |
| 476 | Obacz J, Avril T, Le Reste PJ, Urra H, Quillien V, Hetz C, et al. Endoplasmic reticulum proteostasis in glioblastoma-From molecular mechanisms to therapeutic perspectives. Science signaling. 2017;10(470). Epub 2017/03/16. doi: 10.1126/scisignal.aal2323. PubMed PMID: 28292956. |
| 477 | O'Rorke MA, Bannon FJ, Murray LJ, Hughes CM, Cantwell MM, Gavin AT, et al. Nonsteroidal anti-inflammatory drug use and brain tumour risk: A nested case-control study in the general practice research database. Pharmacoepidemiology and Drug Safety. 2012;21:180-1. doi: 10.1002/pds.3324. |
| 478 | O'Boyle K, Golpanian D, Arya M. A Case of Partial Small Bowel Obstruction due to a Rare Type of Intestinal T-Cell Lymphoma. American Journal of Gastroenterology. 2022;117(10):S2206. doi: 10.14309/01.ajg.0000870688.61925.43. |
| 479 | Nuñez-delMoral A, Brocos-Mosquera I, Vialou V, Callado LF, Erdozain AM. Characterization of Hevin (SPARCL1) Immunoreactivity in Postmortem Human Brain Homogenates. Neuroscience. 2021;467:91-109. Epub 2021/05/26. doi: 10.1016/j.neuroscience.2021.05.017. PubMed PMID: 34033869. |
| 480 | Nukolova NV, Baklaushev VP, Khalansky AS, Yusubalieva GM, Sandalova TO, Gubskiy IL, et al. Treatment of brain tumor by targeted cisplatin-loaded nanogels in rats. FEBS Journal. 2013;280:621. doi: 10.1111/febs.12341. |
| 481 | Norman S, Ramos A, Giantini Larsen AM, Bander E, Goldberg J, Parker W, et al. Impact of the COVID-19 pandemic on neuro-oncology outcomes. Journal of neuro-oncology. 2021;154(3):375-81. |
| 482 | Nolan VG, Gapstur R, Gross CR, Desain LA, Neglia JP, Gajjar A, et al. Sleep disturbances in adult survivors of childhood brain tumors. Quality of life research : an international journal of quality of life aspects of treatment, care and rehabilitation. 2013;22(4):781-9. |
| 483 | Noda SE, El-Jawahri A, Patel D, Lautenschlaeger T, Siedow M, Chakravarti A. Molecular advances of brain tumors in radiation oncology. Seminars in radiation oncology. 2009;19(3):171-8. Epub 2009/05/26. doi: 10.1016/j.semradonc.2009.02.005. PubMed PMID: 19464632. |
| 484 | Nitta T, Sato K, Okumura K. Transforming growth factor (TGF)-beta like activity of intracranial meningioma and its effect on cell growth. Journal of the neurological sciences. 1991;101(1):19-23. Epub 1991/01/01. doi: 10.1016/0022-510x(91)90014-x. PubMed PMID: 2027025. |
| 485 | Nishikawa JK, Nimmagadda P, Abraham-Philip N. Path to diagnosis of pseudotumor cerebri in a young female. American Journal of the Medical Sciences. 2024;367:S65-S6. doi: 10.1016/S0002-9629(24)00168-X. |
| 486 | Nishihara R, VanderWeele TJ, Shibuya K, Mittleman MA, Wang M, Field AE, et al. Molecular pathological epidemiology gives clues to paradoxical findings. European journal of epidemiology. 2015;30(10):1129-35. |
| 487 | Nishiguchi T, Iwakiri T, Hayasaki K, Ohsawa M, Yoneda T, Mitsuhashi Y, et al. Post-embolisation susceptibility changes in giant meningiomas: multiparametric histogram analysis using non-contrast-enhanced susceptibility-weighted PRESTO, diffusion-weighted and perfusion-weighted imaging. European radiology. 2013;23(2):551-61. |
| 488 | Nimma S, Desmond M, Greene Z, Rabai F, Seubert C. Perioperative management of awake craniotomy in third trimester of pregnancy. Journal of neurosurgical anesthesiology. 2017;29(4):536. doi: 10.1097/ANA.0000000000000452. |
| 489 | Nikita, Zuckerman S, Stannard B, Sussman E, Hadjipanayis C, Cheng J. Utility of the modified frailty index in the risk stratification of patients undergoing brain tumor resection. Journal of neurosurgery. 2019;131(1):111-2. doi: 10.3171/2019.7.JNS.AANS2019abstracts. |
| 490 | Nikishina VB, Lazarenko VA, Petrash EA, Akhmetzyanova AI. Impairments to Body Image in Meningioma of the Parietal-Occipital Area. Neuroscience and Behavioral Physiology. 2018;48(4):399-403. doi: 10.1007/s11055-018-0577-5. |
| 491 | Nikishina VB, Lazarenko VA, Petrash EA, Ahmetzyanova AI. [Disturbance of body image in patients with meningiomas of parieto-occipital localization]. Zhurnal nevrologii i psikhiatrii imeni SS Korsakova. 2016;116(12):20-4. |
| 492 | Nicol C, Jacquot J, Chebane L, Combret S, Pecquet PE, Massy N, et al. Bariatric surgery and drugs: Review of the literature and Adverse Drug Reactions analysis in French National Pharmacovigilance Database. Therapies. 2024. doi: 10.1016/j.therap.2024.02.003. |
| 493 | Nia AM, Branch DW, Maynard K, Frank T, Zavlin D, Patterson JT, et al. Metabolic Syndrome Associated with Increased Rates of Medical Complications After Intracranial Tumor Resection. World neurosurgery. 2019;126:e1055-e62. |
| 494 | Nguyen TQH, Luu NT, Nguyen TV. Evaluating the effectiveness of standard tube feeding product on neurology surgery patients at Choray hospital. Clinical nutrition ESPEN. 2020;40:609. doi: 10.1016/j.clnesp.2020.09.608. |
| 495 | Nguyen MP, Morshed RA, Cheung SW, Theodosopoulos PV, McDermott MW. Postoperative Complications and Neurological Deficits After Petroclival Region Meningioma Resection: A Case Series. Operative neurosurgery (Hagerstown, Md). 2023;25(3):251-9. |
| 496 | Neves ER, Mueller J, Anand A, Selting KA, Harley BA, Pedron-Haba S. Targeting the tumor extracellular matrix to enhance therapeutic interventions in glioblastoma. Cancer research. 2023;83(7). doi: 10.1158/1538-7445.AM2023-1191. |
| 497 | Neves ER, Mueller J, Anand A, Selting K, Xu H, Remy R, et al. Disruption of hyaluronan metabolism alters glioma cell proliferation by ligand dependent and independent mechanisms. Cancer research. 2024;84(6). doi: 10.1158/1538-7445.AM2024-4272. |
| 498 | Neves ER, Harley BA, Pedron S. Tumor - driven extracellular matrix remodeling influences therapeutic response. Cancer research. 2022;82(12). doi: 10.1158/1538-7445.AM2022-179. |
| 499 | Neuwelt EA, Specht HD, Hill SA. Permeability of human brain tumor to 99mTc-glucoheptonate and 99mTc-albumin: Implications for monoclonal antibody therapy. Journal of neurosurgery. 1986;65(2):194-8. doi: 10.3171/jns.1986.65.2.0194. |
| 500 | Neuwelt EA, Specht HD, Barnett PA, Dahlborg SA, Miley A, Larson SM, et al. Increased delivery of tumor-specific monoclonal antibodies to brain after osmotic blood-brain barrier modification in patients with melanoma metastatic to the central nervous system. Neurosurgery. 1987;20(6):885-95. |
| 501 | Neuwelt EA, Barnett PA, McCormick CI, Remsen LG, Kroll RA, Sexton G. Differential permeability of a human brain tumor xenograft in the nude rat: impact of tumor size and method of administration on optimizing delivery of biologically diverse agents. Clinical cancer research : an official journal of the American Association for Cancer Research. 1998;4(6):1549-55. |
| 502 | Nedergaard MK, Kristoffersen K, Poulsen HS, Stockhausen MT, Lassen U, Kjaer A. Longitudinal 18F-FET micropet imaging to evaluate treatment response in human glioblastoma multiforme xenografts. Neuro-oncology. 2013;15:iii237. doi: 10.1093/neuonc/not193. |
| 503 | Neal JH, Cotman CW. Human brain tumor cyst fluid is mitogenic for primary astrocytes in culture. Neurosurgery. 1993;32(1):95-8; discussion 8-9. |
| 504 | Nazari S, Mollaei Tavana P, Khalili M. Cerebral infiltrative lesion and chronic clinical course of the rosai-dorfman disease. Iranian Journal of Child Neurology. 2021;15(2):77-85. doi: 10.22037/ijcn.v15i4.30629. |
| 505 | Nayeri A, Chotai S, Prablek MA, Brinson PR, Douleh DG, Weaver KD, et al. Type 2 diabetes is an independent negative prognostic factor in patients undergoing surgical resection of a WHO grade I meningioma. Clinical neurology and neurosurgery. 2016;149:6-10. |
| 506 | Nayeri A, Chotai S, Brinson P, Prablek M, Chambless L. Increased long-term mortality in type 2 diabetics undergoing surgical resection of a who grade I meningioma. Journal of Neurological Surgery, Part B: Skull Base. 2016;77. doi: 10.1055/s-0036-1579929. |
| 507 | Nayeri A, Brinson P, Prablek M, Douleh D, Chambless L. Type 2 diabetes mellitus is an independent risk factor for perioperative complications in patients surgically treated for meningioma. Neuro-oncology. 2015;17:v133. doi: 10.1093/neuonc/nov220.14. |
| 508 | Natarajan A, Kumarasamy S. Efficient Segmentation of Brain Tumor Using FL-SNM with a Metaheuristic Approach to Optimization. Journal of medical systems. 2019;43(2):25. Epub 2019/01/04. doi: 10.1007/s10916-018-1135-y. PubMed PMID: 30604101. |
| 509 | Naseer A, Yasir T, Azhar A, Shakeel T, Zafar K. Computer-Aided Brain Tumor Diagnosis: Performance Evaluation of Deep Learner CNN Using Augmented Brain MRI. International Journal of Biomedical Imaging. 2021;2021. doi: 10.1155/2021/5513500. |
| 510 | Nakhla M, Vengerovich G, Zhang H, Bergsneider M, Kim W, Lee J, et al. Risk Factors and Predictors of Intraoperative Cerebrospinal Fluid Leak after Endoscopic Transnasal Transsphenoidal Surgery for Tumor Resection: A 1,088 Patients' Prospectively Collected Series1. Journal of Neurological Surgery, Part B Skull Base. 2022;83(SUPPL 1). doi: 10.1055/s-0042-1743625. |
| 511 | Nakano F, Matsubara T, Ishigaki T, Hatazaki S, Mouri G, Nakatsuka Y, et al. Incidence and risk factor of deep venous thrombosis in patients undergoing craniotomy for brain tumors: A Japanese single-center, retrospective study. Thrombosis research. 2018;165:95-100. |
| 512 | Nakamura Y, Shimizu T, Ohigashi Y, Itou N, Ishikawa Y. Meningioma arising in Werner syndrome confirmed by mutation analysis. Journal of clinical neuroscience : official journal of the Neurosurgical Society of Australasia. 2005;12(4):503-6 |
| 513 | Nakamura O, Shitara N, Matsutani M, Takakura K, Machida H. Phase I-II trials of poly(ICLC) in malignant brain tumor patients. Journal of interferon research. 1982;2(1):1-4. Epub 1982/01/01. doi: 10.1089/jir.1982.2.1. PubMed PMID: 6180095. |
| 514 | Nakagomi T, Takakura K. Dynamic computed tomography of brain tumors. Brain and Nerve. 1984;36(10):1031-40. |
| 515 | Nakagomi T, Segawa H, Tanaka H. Dynamic computed tomography of the brain. Neurosurgical review. 1985;8(1):15-25. Epub 1985/01/01. doi: 10.1007/bf01744874. PubMed PMID: 4022396. |
| 516 | Nagahama A, Yashiro M, Miki Y, Morisako H, Uda T, Goto T, et al. P53 expression is a useful predictive marker for recurrence of meningioma. Cancer research. 2019;79(13). doi: 10.1158/1538-7445.SABCS18-4901. |
| 517 | Naderi N, Moriyama D, Lin J, Sazgar M, Sen-Gupta I, Mnatsakanyan L. Predictors of in-hospital mortality in status epilepticus; data from the nationwide inpatient sample database, 2008-2012. Neurology. 2015;84. |
| 518 | Muster V, Raggam RB, Urbanic-Purkart T, Posch F, Gütl K, Rabensteiner J, et al. LOWER-EXTREMITY DEEP VEIN THROMBOSIS AND BLEEDING COMPLICATIONS IN PRIMARY BRAIN TUMOR PATIENTS: A PROSPECTIVE STUDY. Neuro-oncology. 2022;24:ii54-ii5. |
| 519 | Murakami M, Ushio Y, Morino Y, Ohta T, Matsukado Y. Immunohistochemical localization of apolipoprotein E in human glial neoplasms. The Journal of clinical investigation. 1988;82(1):177-88. Epub 1988/07/01. doi: 10.1172/jci113568. |
| 520 | Mundada R, Shiffrin E. A rare cause of hypopituitarism in a down syndrome patient. Endocrine Practice. 2020;26(SUPPL 2):234. |
| 521 | Mumoli N, Barco S, Cei M, Giorgi-Pierfranceschi M, Campanini M, Fontanella A, et al. Prevention and treatment of venous thromboembolism in patients with solid brain neoplasms: results of a survey among Italian physicians. Internal and emergency medicine. 2017;12(4):437-43. |
| 522 | Mulrooney DA, Yeazel MW, Kawashima T, Mertens AC, Mitby P, Stovall M, et al. Cardiac outcomes in a cohort of adult survivors of childhood and adolescent cancer: retrospective analysis of the Childhood Cancer Survivor Study cohort. BMJ (Clinical research ed). 2009;339:b4606. |
| 523 | Muhlestein WE, Akagi DS, Kallos JA, Morone PJ, Weaver KD, Thompson RC, et al. Using a Guided Machine Learning Ensemble Model to Predict Discharge Disposition following Meningioma Resection. Journal of Neurological Surgery, Part B: Skull Base. 2018;79(2):123-30. |
| 524 | Muhlestein WE, Akagi DS, Davies JM, Chambless LB. Predicting Inpatient Length of Stay After Brain Tumor Surgery: Developing Machine Learning Ensembles to Improve Predictive Performance. Neurosurgery. 2019;85(3):384-93. Epub 2018/08/17. doi: 10.1093/neuros/nyy343. |
| 525 | Mueller BA, Chow EJ, Kamineni A, Daling JR, Fraser A, Wiggins CL, et al. Pregnancy outcomes in female childhood and adolescent cancer survivors: a linked cancer-birth registry analysis. Archives of pediatrics & adolescent medicine. 2009;163(10):879-86. |
| 526 | Moussa WMM, Mohamed MAA. Prophylactic use of anticoagulation and hemodilution for the prevention of venous thromboembolic events following meningioma surgery. Clinical neurology and neurosurgery. 2016;144:1-6. doi: 10.1016/j.clineuro.2016.02.040. |
| 527 | Mousavi SM, Shayanfar M, Rigi S, Mohammad-Shirazi M, Sharifi G, Esmaillzadeh A. Adherence to the Mediterranean dietary pattern in relation to glioma: A case-control study. Clinical nutrition (Edinburgh, Scotland). 2021;40(1):313-9. |
| 528 | Mousavi SM, Rigi S, Shayanfar M, Mohammad-Shirazi M, Sharifi G, Esmaillzadeh A. Refined grains consumption is associated with a greater odds of glioma. Nutritional neuroscience. 2022;25(3):432-40. Epub 2020/05/08. doi: 10.1080/1028415x.2020.1758889. PubMed PMID: 32378475. |
| 529 | Mott JD, Thomas CL, Rosenbach MT, Takahara K, Greenspan DS, Banda MJ. Post-translational proteolytic processing of procollagen C-terminal proteinase enhancer releases a metalloproteinase inhibitor. The Journal of biological chemistry. 2000;275(2):1384-90. |
| 530 | Motomura K, Sumita K, Chalise L, Nishikawa T, Tanahashi K, Ohka F, et al. Characterization of Intraoperative Motor Evoked Potential Monitoring for Surgery of the Pediatric Population with Brain Tumors. World neurosurgery. 2018;119:e1052-e9. |
| 531 | Motkoski JW, Yang FW, Lwu SH, Sutherland GR. Toward robot-assisted neurosurgical lasers. IEEE transactions on bio-medical engineering. 2013;60(4):892-8. Epub 2012/10/11. doi: 10.1109/tbme.2012.2218655. PubMed PMID: 23047855. |
| 532 | Moseeva MB, Azizova TV, Bannikova MV. Risk of central nervous system tumour incidence in a cohort of workers chronically exposed to ionising radiation. Radiation and environmental biophysics. 2024;63(1):17-26. |
| 533 | Moschovi M, Pavlopoulos A, Zampogiannis A, Lagos A, Stathaki D. Anthropometric data in childhood brain tumors. Pediatric Blood and Cancer. 2018;65:S443. doi: 10.1002/pbc.27455. |
| 534 | Morrison LC, Tatari N, Werbowetski-Ogilvie TE. Embryonic Stem Cell Models of Human Brain Tumors. 2019. p. 127-42. |
| 535 | Morris GM, Micca PL, Coderre JA. The effect of dexamethasone on the uptake of p-boronophenylalanine in the rat brain and intracranial 9L gliosarcoma. Applied radiation and isotopes : including data, instrumentation and methods for use in agriculture, industry and medicine. 2004;61(5):917-21. |
| 536 | Morone PJ, Stewart TG, Zuckermen SL, Dewan MC, Mistry A, Agazzi S, et al. A novel risk stratification tool to predict hospital length of stay after surgery for meningioma. Journal of Neurological Surgery, Part B: Skull Base. 2018;79. doi: 10.1055/s-0038-1633425. |
| 537 | Moriuchi S, Shimizu K, Miyao Y, Hayakawa T. Characterisation of a new mouse monoclonal antibody (ONS-M21) reactive with both medulloblastomas and gliomas. British journal of cancer. 1993;68(5):831-7. |
| 538 | Morgan ER, Mason WP, Maurice C. A critical balance: managing coagulation in patients with glioma. Expert review of neurotherapeutics. 2016;16(7):803-14. Epub 2016/04/22. doi: 10.1080/14737175.2016.1181542. PubMed PMID: 27101362. |
| 539 | Morales-Santiago AB, Toro DH, Vazquez EG, Rosa S. Unfortunate presentation of a common but forgotten parasite infection in the caribbean. American Journal of Gastroenterology. 2017;112:S779-S80. doi: 10.1038/ajg.2017.312. |
| 540 | Morales JS, Valenzuela PL, Herrera-Olivares AM, Rincón-Castanedo C, Martín-Ruiz A, Castillo-García A, et al. What are the effects of exercise training in childhood cancer survivors? A systematic review. Cancer metastasis reviews. 2020;39(1):115-25. |
| 541 | Molloy K, Odedra S, Vydianath B, Chaganti S, Yoo J, Scarisbrick J. Oral mycosis fungoides: a rare clinical entity. British Journal of Dermatology. 2021;185:18. doi: 10.1111/bjd.19953. |
| 542 | Mohr SB, Gorham ED, Garland CF, Grant WB, Garland FC. Low ultraviolet B and increased risk of brain cancer: an ecological study of 175 countries. Neuroepidemiology. 2010;35(4):281-90. |
| 543 | Modzelewska P, Chludzińska S, Lewko J, Reszeć J. The influence of leptin on the process of carcinogenesis. Wspolczesna Onkologia. 2019;23(2):63-8. doi: 10.5114/wo.2019.85877. |
| 544 | Mobarak NA, Alotabi F, Balbaid AAO, Al Shakweer W, Alrasheed R, Al Malki STA, et al. DURABLE RESPONSE TO IMMUNOTHERAPY IN AN ADOLESCENT PATEINT WITH CONSTITUTIONAL MISMATCH REPAIR DEFICIENCY CMMRD AND SYNCHRONOUS THREE PRIMARY MALIGNANCIES. Neuro-oncology. 2024;26:iv84. doi: 10.1093/neuonc/NOAE064.285. |
| 545 | Mizobuchi Y, Nakajima K, Fujihara T, Azumi M, Takagi Y. Development of a Navigation-guided Fence-post Catheter for Brain Tumor Resection. The journal of medical investigation : JMI. 2022;69(1.2):117-9. Epub 2022/04/26. doi: 10.2152/jmi.69.117. PubMed PMID: 35466132. |
| 546 | Miyatani S, Kuchiki M, Hattori M, Kyoya T, Doshida M, Toya M, et al. Successful pregnancies following assisted reproductive technology (ART) for cancer survivors. Human Reproduction. 2014;29:i255. doi: 10.1093/humrep/29.Supplement_1.1. |
| 547 | Mittal R, Malik V, Singla G, Kaur A, Singh M, Mittal A. 3D reconstruction of brain tumors from 2D MRI scans: An improved marching cube algorithm. Biomedical Signal Processing and Control. 2024;91. doi: 10.1016/j.bspc.2023.105901. |
| 548 | Mittal BR, Agrawal K, Shukla J, Vatsa R, Bhattacharya A, Singh B. Ga-68 DOTATATE PET/CT in identification of primary site in patients with carcinoma of unknown primary-neuroendocrine tumors. European Journal of Nuclear Medicine and Molecular Imaging. 2013;40:S376-S7. |
| 549 | Mirzaei MR, Arababadi MK, Asadi MH, Mowla SJ. Altered expression of high molecular weight heat shock proteins after OCT4B1 suppression in human tumor cell lines. Cell Journal. 2016;17(4):608-16. |
| 550 | Miller J, Shoemaker A, Abuzzahab MJ, Gottschalk M, Yuan G, Malhotra S, et al. Impact of Setmelanotide Treatment on Weight- and Body Composition-Related Outcomes in Pediatric and Adult Patients With Hypothalamic Obesity. Hormone research in paediatrics. 2023;96:262-3. doi: 10.1159/000531602. |
| 551 | Miller AB, Green LM. Electric and magnetic fields at power frequencies. Chronic diseases in Canada. 2010;29 Suppl 1:69-83. Epub 2011/01/14. PubMed PMID: 21199600. |
| 552 | Michaelides S, Bablekos G, Kourtelessi E, Alexopoulos A, Ionas G, Paraskevopoulos K, et al. Distribution of dyspnea etiologies among patients with either acute or chronic breathlessness and normal cardiorespiratory function. European Respiratory Journal. 2012;40. |
| 553 | Miccoli L, Poirson-Bichat F, Sureau F, Bras Gonçalves R, Bourgeois Y, Dutrillaux B, et al. Potentiation of lonidamine and diazepam, two agents acting on mitochondria, in human glioblastoma treatment. Journal of the National Cancer Institute. 1998;90(18):1400-6. |
| 554 | Metrus N, Matsuoka CK, Johnson J. Paroxysmal singultus as the result of brainstem histoplasmosis. Neurology. 2018;90(15). |
| 555 | Merrell R, Castillo-Saavedra L, Yuen C, Wilmington A, Vogel MTJ, Walker M, et al. Estrogen hormone replacement therapy in incidental meningioma-a growth rate analysis. Neurology. 2020;94(15). |
| 556 | Menon LG, Armant M, Badr C, Bakhos TA, Yang H, Pratt J, et al. A local cell-based delivery system for human gliomas using human bone marrow-derived mesenchymal stromal cells. Neuro-oncology. 2009;11(5):589. doi: 10.1215/15228517-2009-034. |
| 557 | Menezes JC, Troster EJ, Dichtchekenian V. Digoxin antibody decreases natriuresis and diuresis in cerebral hemorrhage. Intensive care medicine. 2003;29(12):2291-6. Epub 2003/09/05. doi: 10.1007/s00134-003-1955-0. PubMed PMID: 12955184. |
| 558 | Menezes AH. Craniovertebral junction neoplasms in the pediatric population. Child's nervous system : ChNS : official journal of the International Society for Pediatric Neurosurgery. 2008;24(10):1173-86. Epub 2008/04/11. doi: 10.1007/s00381-008-0598-4. PubMed PMID: 18401564. |
| 559 | Mehta M, Khan AJ, Sabaawy HE, Haffty BG. The effect of BMI-1 inhibition on brain cancer stem cells. Journal of Clinical Oncology. 2012;30(15). |
| 560 | Mehrvar A, Tashvighi M, Nadrei A, Nikfarjam H, Mehrvar N, Otoukesh A, et al. Infantile malignancies incidence and survival under 6 months. Iranian Journal of Blood and Cancer. 2017;9(3):22. |
| 561 | Medina RT, Ferreira FCS, Pires ARC, Arêas ALBG. Primary pulmonary meningioma: A case report. European Journal of Surgical Oncology. 2015;41:S164. |
| 562 | McNally BB, Sivakumar A, Haddad N, Suchartlikitwong S, Muna Aguon P, Fung BM, et al. Vedolizumab treatment of refractory immune checkpoint inhibitor colitis. American Journal of Gastroenterology. 2021;116(SUPPL):S756. doi: 10.14309/01.ajg.0000780316.76780.1f. |
| 563 | McKone EL, Breen W, Foster NR, Bogan AW, Alstat RA, Boyce S, et al. Memantine for Pediatric Patients Receiving Cranial Irradiation: A Pilot Study. International Journal of Radiation Oncology Biology Physics. 2023;117(2):S134-S5. doi: 10.1016/j.ijrobp.2023.06.537. |
| 564 | McKee S, Yang A, Del Signore A, Bederson J, Iloreta A, Shrivastava R. Excess charges in intracranial meningioma surgery: The influence of patient and hospital characteristics. Journal of Neurological Surgery, Part B: Skull Base. 2018;79. doi: 10.1055/s-0038-1633422. |
| 565 | McIver BA, Davis TS, Reinhart K, Vera E, Choi A, Kunst T, et al. Evaluating clinical and sociodemographic risk for symptom-related functional interference in the primary brain tumor population. Cancer research. 2024;84(6). doi: 10.1158/1538-7445.AM2024-6305. |
| 566 | McHayle A, Pertsch NJ, Toms SA, Weil RJ. Operative duration and early outcomes in patients having a supratentorial craniotomy for brain tumor: A propensity matched analysis. Journal of clinical neuroscience : official journal of the Neurosurgical Society of Australasia. 2021;92:207-14. |
| 567 | McDonald KL, Sevim H, Ha W, Chung L, Baxter R. MIF/CD74 guided therapeutic strategy for relapsed glioblastoma patients. Cancer research. 2014;74(19). doi: 10.1158/1538-7445.AM2014-2844. |
| 568 | McDonagh D, Ouyang M, Phillips-Bute B, James M, Gan T. Does midline-shift predict postoperative nausea in brain tumor patients undergoing awake craniotomy? A retrospective study. Journal of neurosurgical anesthesiology. 2012;24(4):461. |
| 569 | McDonagh D, Mingwen O, Phillips-Bute B, James M, Gan T. Postoperative nausea in patients with benign and malignant brain tumors undergoing awake craniotomy: A retrospective study. Journal of neurosurgical anesthesiology. 2012;24(4):498. |
| 570 | McCutcheon IE, Flyvbjerg A, Hill H, Li J, Bennett WF, Scarlett JA, et al. Antitumor activity of the growth hormone receptor antagonist pegvisomant against human meningiomas in nude mice. Journal of neurosurgery. 2001;94(3):487-92. |
| 571 | Mazzatenta D, Zoli M, Guaraldi F, Ambrosi F, Faustini Fustini M, Pasquini E, et al. Outcome of Endoscopic Endonasal Surgery in Pediatric Craniopharyngiomas. World neurosurgery. 2020;134:e277-e88. Epub 2019/10/21. doi: 10.1016/j.wneu.2019.10.039. PubMed PMID: 31629927. |
| 572 | Maturi N, Tan EJ, Xie Y, Sundström A, Bergström T, Casar-Borota O, et al. Serum established cancer stem cell cultures represent unique models of glioblastoma intertumor heterogeneity. Neuro-oncology. 2019;21:vi264. doi: 10.1093/neuonc/noz175.1107. |
| 573 | Matrasova I, Busek P, Balaziova E, Sedo A. Heterogeneity of molecular forms of dipeptidyl peptidase-IV and fibroblast activation protein in human glioblastomas. Biomedical papers of the Medical Faculty of the University Palacky, Olomouc, Czechoslovakia. 2017;161(3):252-60. |
| 574 | Mathew M, Salahuddin A, Mathew NR, Nandhagopal R. Idiopathic intracranial hypertension presenting as postpartum headache. Neurosciences (Riyadh, Saudi Arabia). 2016;21(1):52-5. |
| 575 | Mathe D, Horvath I, Szigeti K, Janoki GA, Horváth Á, Jánoki G, et al. Quantitative SPECT/CT imaging of glioma burden in nude rats with 99mTc-GJ30, a novel Sstr2a-ligand formulation. Molecular imaging and biology. 2012;14:S322. doi: 10.1007/s11307-012-0543-5. |
| 576 | Mashiko T, Oguma H, Konno T, Gomi A, Yamaguchi T, Nagayama R, et al. Training of Intra-Axial Brain Tumor Resection Using a Self-Made Simple Device with Agar and Gelatin. World neurosurgery. 2018;109:e298-e304. Epub 2017/10/11. doi: 10.1016/j.wneu.2017.09.162. PubMed PMID: 28987833. |
| 577 | Maschio M, Sperati F, Dinapoli L, Vidiri A, Fabi A, Pace A, et al. Weight of epilepsy in brain tumor patients. Journal of neuro-oncology. 2014;118(2):385-93. Epub 2014/05/03. doi: 10.1007/s11060-014-1449-7. PubMed PMID: 24789254. |
| 578 | Marx M, Langer T, Beck JD, Dörr HG. A review of endocrine late effects in children after brain tumor therapy. Strahlentherapie und Onkologie. 1999;175(7):305-8. doi: 10.1007/PL00002297. |
| 579 | Marx M, Langer T, Beck JD, Dörr HG. [Disorders of endocrine function after brain tumor therapy in childhood]. Strahlentherapie und Onkologie : Organ der Deutschen Rontgengesellschaft [et al]. 1999;175(7):305-8. Epub 1999/08/05. doi: 10.1007/pl00002297. PubMed PMID: 10432990. |
| 580 | Martuscello RT, Deleyrolle LP, Louviere CD, McCarthy DJ, Skinner CL, Jundi MA, et al. Diminished progression of glioblastoma with a supplemented high-fat low-carbohydrate diet. Neuro-oncology. 2014;16:v215. doi: 10.1093/neuonc/nou278.12. |
| 581 | Martucci M, Gaudino S, Schiarelli C, Colantonio R, Costantini A, Tartaglione T, et al. Brain tumor perfusion: Comparison of dynamic susceptibility contrast-enhanced mr imaging values in infratentorial and supratentorial lesions. Neuroradiology. 2011;53:S55. doi: 10.1007/s00234-011-0914-7. |
| 582 | Marsland M, Dowdell A, Faulkner S, Gedye C, Lynam J, Griffin CP, et al. The Membrane Protein Sortilin Is a Potential Biomarker and Target for Glioblastoma. Cancers. 2023;15(9). doi: 10.3390/cancers15092514. |
| 583 | Marosi C. Complications of chemotherapy in neuro-oncology. 2012. p. 873-85. |
| 584 | Markowitz D, Aamodt WW, Hamedani AG. Social Determinants of Health in Idiopathic Intracranial Hypertension. Journal of neuro-ophthalmology : the official journal of the North American Neuro-Ophthalmology Society. 2024;44(3):346-9. |
| 585 | Marinelli JP, Modzeski MC, Lane JI, Van Gompel JJ, Stokken JK, Thanarajasingam G, et al. Primary Skull Base Lymphoma: Manifestations and Clinical Outcomes of a Great Imitator. Otolaryngology--head and neck surgery : official journal of American Academy of Otolaryngology-Head and Neck Surgery. 2018;159(4):643-9. |
| 586 | Marinău LD, Singer CE, Meşină C, Niculescu EC, Puiu I, Petrescu IO, et al. Two girl patients with medulloblastoma. Case reports. Romanian journal of morphology and embryology = Revue roumaine de morphologie et embryologie. 2017;58(3):1103-8. |
| 587 | Mariappan R, Venkatraghavan L. Is serum lactate a potential biomarker for malignant primary brain tumors? Retrospective chart review. Journal of neurosurgical anesthesiology. 2013;25(4):494-5. doi: 10.1097/ANA.0b013e3182a4d5ff. |
| 588 | Mareninov S, Kay A, Sanchez D, Mirsadraei L, Lou J, Shafa B, et al. Lyophilization as a brain tumor preservation method for long-term storage at ambient temperature. Journal of Neuropathology and Experimental Neurology. 2011;70(6):523. doi: 10.1097/NEN.0b013e3182227917. |
| 589 | Mareninov S, De Jesus J, Sanchez DE, Kay AB, Wilson RW, Babic I, et al. Lyophilized brain tumor specimens can be used for histologic, nucleic acid, and protein analyses after 1 year of room temperature storage. Journal of neuro-oncology. 2013;113(3):365-73. |
| 590 | Marcus CL, Trescher WH, Halbower AC, Lutz J. Secondary narcolepsy in children with brain tumors. Sleep. 2002;25(4):435-9. Epub 2002/06/20. PubMed PMID: 12071545. |
| 591 | Marchand L, Leconte M, Jeanneau R, Chazaud J. [Meningioma, ocular melanoma & liver, kidney, lung & adrenal metastases, uterine leiomyoma & adenomatous hyperplasia of the pituitary in a manic obese female]. Annales medico-psychologiques. 1957;115(5):924-30. |
| 592 | Maravilla KR, Smith MP, Vymazal J, Goyal M, Herman M, Baima JJ, et al. Are there differences between macrocyclic gadolinium contrast agents for brain tumor imaging? Results of a multicenter intraindividual crossover comparison of gadobutrol with gadoteridol (the TRUTH study). AJNR American journal of neuroradiology. 2015;36(1):14-23. |
| 593 | Manuela P, Elio R, Erika C, Editta B. Integrative approaches of diet and complementary medicine for the treatment of chemotherapy and hormone therapy side effects in patients with solid tumour: Experience in the hospital of Lucca (Italy). European Journal of Integrative Medicine. 2012;4:15-6. |
| 594 | Mantilla E, Maalouf S, Olurinde O, Singh V. Low serum 25 (OH) vitamin D level is associated with increased risk of primary CNS malignancy: A retrospective cohort study in a veteran population. Neuro-oncology. 2018;20:vi283. doi: 10.1093/neuonc/noy148. |
| 595 | Mantia C, Zwicker JI. Anticoagulation in the setting of primary and metastatic brain tumors. 2019. p. 179-89. |
| 596 | Mansell M, Hill R, Johnson D, Hamby T, Hines A. Favorable Parental Perception of Proactive Nutrition Intervention in High-risk Pediatric Brain Tumor Population and Impact on Nutrition Outcomes. Journal of pediatric hematology/oncology. 2023;45(1):e103-e8. |
| 597 | Manjunath C, Mahurkar N. In vitro cytotoxicity of cardamom oil, lemon oil, and jasmine oil on human skin, gastric, and brain cancer cell line. Journal of cancer research and therapeutics. 2021;17(1):62-8. Epub 2021/03/17. doi: 10.4103/jcrt.JCRT_915_17. PubMed PMID: 33723134. |
| 598 | Manam A, Lakhoo K, Trivedi I. Incidentally Diagnosed Triple Cancers Within 2 Years: A Novel Case of Rare Metachronous Cancers, Renal Cell Carcinoma, Cholangiocarcinoma, and Squamous Cell Carcinoma of the Gastroesophageal Junction. American Journal of Gastroenterology. 2022;117(10):S1571. |
| 599 | Man Hon T, Goodman C, Moseley H, Eljamel S. Protoporphyrin-IX fluorescence in skull base meningiomas' resection. Journal of Neurological Surgery, Part B: Skull Base. 2013;74. doi: 10.1055/s-0033-1336196. |
| 600 | Maltby S, Brewer G, Ellerman J, Krishnan A. Sarcoidosis: The Great Malignancy Mimic. Diagnosis. 2024;11(2):A81-A2. doi: 10.1515/dx-2023-0177. |
| 601 | Malmir H, Shayanfar M, Mohammad-Shirazi M, Tabibi H, Sharifi G, Esmaillzadeh A. Patterns of nutrients intakes in relation to glioma: A case-control study. Clinical nutrition (Edinburgh, Scotland). 2019;38(3):1406-13. Epub 2018/07/02. doi: 10.1016/j.clnu.2018.06.961. PubMed PMID: 29960808. |
| 602 | Malmir H, Shayanfar M, Mohammad-Shirazi M, Tabibi H, Sharifi G, Esmaillzadeh A. Tea and coffee consumption in relation to glioma: a case-control study. European journal of nutrition. 2019;58(1):103-11. Epub 2017/11/11. doi: 10.1007/s00394-017-1575-z. PubMed PMID: 29124385. |
| 603 | Maliniak ML, Gapstur SM, McCullough LE, Rees-Punia E, Gaudet MM, Um CY, et al. Joint associations of physical activity and body mass index and the risk of excess body fatness-related cancer. Cancer research. 2019;79(13). doi: 10.1158/1538-7445.am2019-961. |
| 604 | Maliniak ML, Gapstur SM, McCullough LE, Rees-Punia E, Gaudet MM, Um CY, et al. Abstract 961: Joint associations of physical activity and body mass index and the risk of excess body fatness-related cancer. Cancer research. 2019;79(13). doi: 10.1158/1538-7445.SABCS18-961. |
| 605 | Maldar A, Prathap HJ, Pawar S, Bhandary R. Prognostic role of F-18 FDG uptake in brain in comparison to primary tumor in various malignancies at staging. Indian Journal of Nuclear Medicine. 2019;34(5):S39. doi: 10.4103/0972-3919.271608. |
| 606 | Malczewska A, Vlavianos P, Faiz O, Gabe S, Abraham R, Goldin R, et al. MEN1-related glucagonomatosis incidentally revealed during management of recurrent intestinal obstruction following surgery for crohn's disease. Neuroendocrinology. 2016;103:111. doi: 10.1159/000448725. |
| 607 | Malczewska A, Vlavianos P, Faiz O, Gabe S, Abraham R, Al-Nahhas A, et al. MEN1-associated glucagonomatosis - Case report. Endokrynologia Polska. 2016;67:A50-A1. |
| 608 | Mahmoud BS, Alamri AH, McConville C. Polymeric nanoparticles for the treatment of malignant gliomas. Cancers. 2020;12(1). doi: 10.3390/cancers12010175. |
| 609 | Magni R, Verderio P, Liotta L, Spreafico F, Massimino M, Luchini A, et al. Investigation of the cerebrospinal fluid proteome from central nervous system pediatric tumors using bait loaded hydrogel nanoparticles and mass spectrometry. Cancer research. 2014;74(19). doi: 10.1158/1538-7445.AM2014-1874. |
| 610 | Magerman C, Boros E, Preziosi M, Lhoir S, Gilis N, De Witte O, et al. Childhood craniopharyngioma: a retrospective study of children followed in Hôpital Universitaire de Bruxelles. Frontiers in endocrinology. 2024;15:1297132. |
| 611 | Madhusoodhanan S, Kesavadas C, Paul JS. SWI processing using a local phase difference modulated venous enhancement filter with noise compensation. Magnetic resonance imaging. 2019;59:17-30. Epub 2019/03/01. doi: 10.1016/j.mri.2019.02.012. PubMed PMID: 30817962. |
| 612 | Macias JJ, Lee YC, Finkelberg T, Zdioruk M, Berger G, Nowicki M, et al. Assessment of brain penetrance, biodistribution, and efficacy of platinum (iv)-conjugated fluorinated macrocyclic cellpenetrating peptides in a murine glioblastoma model. Neuro-oncology. 2021;23(SUPPL 6):vi84-vi5.. |
| 613 | Macartney G, Stacey D, Harrison MB, VanDenKerkhof E. Symptoms, coping, and quality of life in pediatric brain tumor survivors: a qualitative study. Oncology nursing forum. 2014;41(4):390-8. Epub 2014/06/28. doi: 10.1188/14.Onf.390-398. PubMed PMID: 24969249. |
| 614 | Ma M, Wieder MS, Kranitzky B. A vertebral lytic lesion without an obvious source: Colorectal cancer presenting solely with a bony lytic lesion. American Journal of Gastroenterology. 2017;112:S845. doi: 10.1038/ajg.2017.312. |
| 615 | Lv Y, Mao X, Deng Y, Yu L, Chu J, Hao S, et al. Surgical site infections after elective craniotomy for brain tumor: a study on potential risk factors and related treatments. Chinese Neurosurgical Journal. 2023;9(1). doi: 10.1186/s41016-023-00336-1. |
| 616 | Lv PC, Roy J, Putt KS, Low PS. Evaluation of a Carbonic Anhydrase IX-Targeted Near-Infrared Dye for Fluorescence-Guided Surgery of Hypoxic Tumors. Molecular pharmaceutics. 2016;13(5):1618-25. |
| 617 | Lv H, Zhang X, Sharma J, Reddy MVR, Reddy EP, Gallo JM. Integrated pharmacokinetic-driven approach to screen candidate anticancer drugs for brain tumor chemotherapy. AAPS Journal. 2013;15(1):250-7. doi: 10.1208/s12248-012-9428-4. PubMed Central PMCID: Reddy. |
| 618 | Lv H, Wang F, Ramana Reddy MV, Zhou Q, Zhang X, Reddy EP, et al. Screening candidate anticancer drugs for brain tumor chemotherapy: Pharmacokinetic-driven approach for a series of (E)-N-(substituted aryl)-3-(substituted phenyl)propenamide analogues. Investigational new drugs. 2012;30(6):2263-73. doi: 10.1007/s10637-012-9806-x. |
| 619 | Lustig RH, Post SR, Srivannaboon K, Rose SR, Danish RK, Burghen GA, et al. Risk factors for the development of obesity in children surviving brain tumors. The Journal of clinical endocrinology and metabolism. 2003;88(2):611-6. |
| 620 | Luo Z, Jia Z, Yuan Z, Peng J. HDC-Net: Hierarchical Decoupled Convolution Network for Brain Tumor Segmentation. IEEE journal of biomedical and health informatics. 2021;25(3):737-45. Epub 2020/08/06. doi: 10.1109/jbhi.2020.2998146. PubMed PMID: 32750914. |
| 621 | Lum MA, Martin AJ, Alexander MD, McCoy DB, Cooke DL, Lillaney P, et al. Intra-Arterial MR Perfusion Imaging of Meningiomas: Comparison to Digital Subtraction Angiography and Intravenous MR Perfusion Imaging. PloS one. 2016;11(11):e0163554. |
| 622 | Luis GA, Hernández CM. Validation of the perioperative nutritional screen (PONS) for prediction of postoperative outcomes in brain tumor patients. Clinical nutrition ESPEN. 2023;58:572. doi: 10.1016/j.clnesp.2023.09.431. |
| 623 | Lueangarun S, Auewarakul CU. Diffuse large B cell lymphoma presenting as Horner's syndrome in a patient diagnosed with neurofibromatosis type 1: A case report and review of the literature. Journal of medical case reports. 2012;6. doi: 10.1186/1752-1947-6-8. |
| 624 | Lucas JT, LeVine D, Ismael Y, Hsu CY, Darrow KR, Faught AM, et al. Vasculopathy in Pediatric Craniopharyngioma Patients Treated with Surgery and Proton Radiotherapy. International Journal of Radiation Oncology Biology Physics. 2020;108(3):S37-S8. doi: 10.1016/j.ijrobp.2020.07.2142. |
| 625 | Low CA, Jakicic JM, Marsland A, Donovan HS, Weimer J, Sherwood PR. Objective physical activity in neuro-oncology family caregivers: Associations with inflammatory markers. Psychosomatic Medicine. 2015;77(3):A42. |
| 626 | Losonczy H, Nagy Á, Tar A. Actual questions about the prevention of venous thromboembolism in hospitalized and ambulatory cancer patients receiving chemotherapy. Orvosi hetilap. 2016;157(6):203-11. doi: 10.1556/650.2016.30357. |
| 627 | López-Olmos J. HRT with tibolone during the menopause. Clinica e Investigacion en Ginecologia y Obstetricia. 2005;32(3):106-15. doi: 10.1016/s0210-573x(05)73485-7. |
| 628 | Lönnemark O, Ryttlefors M, Sundblom J. Cranioplasty in Brain Tumor Surgery: A Single-Center Retrospective Study Investigating Cranioplasty Failure and Tumor Recurrence. World neurosurgery. 2023;170:e313-e23. Epub 2022/11/11. doi: 10.1016/j.wneu.2022.11.010. PubMed PMID: 36356841. |
| 629 | Longo M, Gelfand Y, De La Garza-Ramos R, Agarwal V. A retrospective analysis of surgical meningioma patients in an underserved population. Journal of Neurological Surgery, Part B: Skull Base. 2019;80. doi: 10.1055/s-0039-1679453. |
| 630 | Long TM, Rath SR, Maroni TD, Wallman KE, Atkinson HC, Gottardo NG, et al. Fitness, body composition and vascular health in adolescent and young adult survivors of paediatric brain cancer and cranial radiotherapy. International Journal of Adolescent Medicine and Health. 2021;31(5). |
| 631 | Loiseau H, Huchet A, Rué M, Cowppli-Bony A, Baldi I. [Epidemiology of primary brain tumor]. Revue neurologique. 2009;165(8-9):650-70. Epub 2009/05/19. doi: 10.1016/j.neurol.2009.04.002. PubMed PMID: 19446856. |
| 632 | Lockwood J, Harris K, Aslam R, Amenta P, Jackson N. Middle fossa meningioma presenting with external auditory canal mass, conductive hearing loss, and facial weakness. Journal of Neurological Surgery, Part B Skull Base. 2020;81. doi: 10.1055/s-0040-1702675. |
| 633 | Lladó V, López DJ, Ibarguren M, Alonso M, Soriano JB, Escribá PV, et al. Regulation of the cancer cell membrane lipid composition by NaCHOleate: effects on cell signaling and therapeutical relevance in glioma. Biochimica et biophysica acta. 2014;1838(6):1619-27. |
| 634 | Liu ZG, Zhao XM, Baxter P, Tao YL, Su J, Adesina A, et al. Radiation resistance of orthotopic glioblastoma xenografts is mediated by increasing CD133+ cell fraction, mobilizing G0 cells into G1 phase, and activating self-renewal and mesenchymal genes. Cancer research. 2011;71(8). doi: 10.1158/1538-7445.AM2011-570. |
| 635 | Liu Y, Hu H, Han Y, Li L, Li Z, Zhang L, et al. Body Mass Index Has a Nonlinear Association With Postoperative 30-Day Mortality in Patients Undergoing Craniotomy for Tumors in Men: An Analysis of Data From the ACS NSQIP Database. Frontiers in endocrinology. 2022;13:868968. |
| 636 | Liu Y, Carpenter A, Yuan H, Zhou Z, Zalutsky M, Vaidyanathan G, et al. Gold nanostar as theranostic probe for brain tumor sensitive PET-optical imaging and imageguided specific photothermal therapy. Cancer research. 2016;76(14). doi: 10.1158/1538-7445.AM2016-4213. |
| 637 | Liu X, Xu Y, Han L, Yi Y. Reassessing the potential of MYB-targeted anti-cancer therapy. Journal of Cancer. 2018;9(7):1259-66. doi: 10.7150/jca.23992. |
| 638 | Liu TF, Cohen KA, Willingham MC, Tatter SB, Puri RK, Frankel AE. Combination fusion protein therapy of refractory brain tumors: demonstration of efficacy in cell culture. Journal of neuro-oncology. 2003;65(1):77-85. Epub 2003/12/03. doi: 10.1023/a:1026286214901. PubMed PMID: 14649887. |
| 639 | Liu H, Liu N, Cheng Y, Jin W, Zhang P, Wang X, et al. Hexokinase 2 (HK2), the tumor promoter in glioma, is downregulated by miR-218/Bmi1 pathway. PloS one. 2017;12(12):e0189353. |
| 640 | Liu F, Duan C, Han Y. Circular RNA hsa_circ_0000285 regulates the microRNA-599/G-protein subunit gamma 12 (miR-599/GNG12) axis to promote glioma progression. Journal of clinical laboratory analysis. 2022;36(3):e24207. |
| 641 | Line S, Sajesh BV, Schwinghamer K, Yathindranath V, Tsoli M, Ziegler D, et al. BLOOD BRAIN BARRIER (BBB) MODULATION USING CADHERIN PEPTIDES IN THE TREATMENT OF DIFFUSE MIDLINE GLIOMA OF THE PONS (DMG-P). Neuro-oncology. 2022;24:i32. |
| 642 | Lin MF, Kawachi MH, Stallcup MR, Grunberg SM, Lin FF. Growth inhibition of androgen-insensitive human prostate carcinoma cells by a 19-norsteroid derivative agent, mifepristone. The Prostate. 1995;26(4):194-204. Epub 1995/04/01. doi: 10.1002/pros.2990260405. PubMed PMID: 7716084. |
| 643 | Lin L, Liao SC, Lee YJ, Tseng MC, Lee MB. Brain tumor presenting as anorexia nervosa in a 19-year-old man. Journal of the Formosan Medical Association = Taiwan yi zhi. 2003;102(10):737-40. Epub 2003/12/24. PubMed PMID: 14691602. |
| 644 | Lin KW, Liao A, Qutub AA. Simulation predicts IGFBP2-HIF1α interaction drives glioblastoma growth. PLoS computational biology. 2015;11(4):e1004169. Epub 2015/04/18. doi: 10.1371/journal.pcbi.1004169. PubMed PMID: 25884993; PubMed Central PMCID: PMC4401766. |
| 645 | Lim U, Subar AF, Mouw T, Hartge P, Morton LM, Stolzenberg-Solomon R, et al. Consumption of aspartame-containing beverages and incidence of hematopoietic and brain malignancies. Cancer epidemiology, biomarkers & prevention : a publication of the American Association for Cancer Research, cosponsored by the American Society of Preventive Oncology. 2006;15(9):1654-9. |
| 646 | Likar R, Nahler G. The use of cannabis in supportive care and treatment of brain tumor. Neuro-Oncology Practice. 2017;4(3):151-60. doi: 10.1093/nop/npw027. |
| 647 | Libermann TA, Razon N, Bartal AD, Yarden Y, Schlessinger J, Soreq H. Expression of epidermal growth factor receptors in human brain tumors. Cancer research. 1984;44(2):753-60. Epub 1984/02/01. PubMed PMID: 6318976. |
| 648 | Liao YM, Yeh CJ, Shu HL, Lin PC, Chang TT, Chiou SS. Successful large-volume leukapheresis for hematopoietic stem cell collection in a very-low-weight brain tumor infant with coagulopathy. Pediatrics and neonatology. 2013;54(3):211-3. |
| 649 | Liao YH, Li B, Li Y, Luo BN, Li Z. Radiological and clinicopathological analysis of intraspinal primary Rosai-Dorfman disease. Chinese Journal of Contemporary Neurology and Neurosurgery. 2014;14(4):322-8. doi: 10.3969/j.issn.1672-6731.2014.04.011. |
| 650 | Liao CH, Wang JT, Lin CF, Chen SC, Lin CJ, Hsu SPC, et al. Pretemporal trans-Meckel's cave transtentorial approach for large petroclival meningiomas. Neurosurgical focus. 2018;44(4):E10. Epub 2018/04/03. doi: 10.3171/2018.1.Focus17733. PubMed PMID: 29606050. |
| 651 | Liang B, Shetty SR, Omay SB, Almeida JP, Ni S, Chen YN, et al. Predictors and incidence of orthostatic headache associated with lumbar drain placement following endoscopic endonasal skull base surgery. Acta neurochirurgica. 2017;159(8):1379-85. |
| 652 | Li XL, Ren J, Niu RN, Jiang X, Xu GH, Zhou P, et al. Primary intracranial leiomyosarcoma in an immunocompetent patient: Case report with emphasis on imaging features. Medicine. 2019;98(17):e15269. |
| 653 | Li X, Fang X, Yang G, Su S, Zhu L, Yu Z. TransU²-Net: An Effective Medical Image Segmentation Framework Based on Transformer and U²-Net. IEEE journal of translational engineering in health and medicine. 2023;11:441-50. |
| 654 | Li W, Wu C, Yao Y, Dong B, Wei Z, Lv X, et al. MUC4 modulates human glioblastoma cell proliferation and invasion by upregulating EGFR expression. Neuroscience letters. 2014;566:82-7. Epub 2014/03/04. doi: 10.1016/j.neulet.2014.02.033. PubMed PMID: 24582898. |
| 655 | Li W, Liu J, Fu W, Zheng X, Ren L, Liu S, et al. 3-O-acetyl-11-keto-β-boswellic acid exerts anti-tumor effects in glioblastoma by arresting cell cycle at G2/M phase. Journal of experimental & clinical cancer research : CR. 2018;37(1):132. |
| 656 | Li S, Chen LN, Zhong L. A 7-year-old boy with recurrent cyanosis and tachypnea: A case report. World Journal of Clinical Cases. 2022;10(20):6974-80. doi: 10.12998/wjcc.v10.i20.6974. |
| 657 | Li Q, Wang J, Zhou Y. Data augmentation method based on the Gaussian kernel density for glioma diagnosis with Raman spectroscopy. Analytical methods : advancing methods and applications. 2023;15(15):1861-9.. |
| 658 | Li PC, Chen SY, Xiangfei D, Mao C, Wu CH, Shih JC. PAMs inhibits monoamine oxidase a activity and reduces glioma tumor growth, a potential adjuvant treatment for glioma. BMC complementary medicine and therapies. 2020;20(1):252. |
| 659 | Li H, Liang Q, Wang L. Icaritin inhibits glioblastoma cell viability and glycolysis by blocking the IL-6/Stat3 pathway. Journal of cellular biochemistry. 2019;120(5):7257-64. doi: 10.1002/jcb.28000. PubMed Central PMCID: winherb medical science(China). |
| 660 | Li B, Gao B, Zhu HJ, Luwor RB, Lu J, Zhang L, et al. The Prognostic Value of Preoperative Inflammatory Markers for Pathological Grading of Glioma Patients. Technology in cancer research & treatment. 2024;23:15330338241273160. |
| 661 | Lewandowski M, Gwoździński K. Photoprotective and radioprotective properties of nitroxides and their application in magnetic resonance imaging. Postepy higieny i medycyny doswiadczalnej (Online). 2016;70(0):1101-11.. |
| 662 | Levy ML, Granville RC, Hart D, Meltzer H. Deep venous thrombosis in children and adolescents. Journal of neurosurgery. 2004;101(1 Suppl):32-7. Epub 2005/10/07. doi: 10.3171/ped.2004.101.2.0032. PubMed PMID: 16206969. |
| 663 | Leung E, Sheikh M, Benzaquen M, Cranston-D'Amato H. A case of Moya Moya disease in a 47 year old female presenting with ischemic stroke. American Journal of Respiratory and Critical Care Medicine. 2015;191. |
| 664 | Leopold A, Yusubalieva GM, Korchagina AA, Melnikov P, Baklaushev VP. Single chain antibody against connexin: A new tool for targeting the peritumoral region of glial tumors. European Neuropsychopharmacology. 2014;24:S234. |
| 665 | Leong CK, Jeyapalan S. Cranio-cervical junction intramural extramedullary meningothelial meningioma in pregnancy: A case report. Medical Journal of Malaysia. 2023;78:52. |
| 666 | Lensu S, Waselius T, Mäkinen E, Kettunen H, Virtanen A, Tiirola M, et al. Irradiation of the head reduces adult hippocampal neurogenesis and impairs spatial memory, but leaves overall health intact in rats. The European journal of neuroscience. 2021;53(6):1885-904. |
| 667 | Lemm D, De Oliveira FH, Bernays RL, Kockro RA, Kollias S, Fischer I, et al. Rare suprasellar glioblastoma: Report of two cases and review of the literature. Brain Tumor Pathology. 2012;29(4):216-20. doi: 10.1007/s10014-012-0086-0. |
| 668 | Lei S, Zheng R, Zhang S, Huang Y, Qiao L, Song B, et al. Years lived with disability of cancer in China: findings from disability weights measurement with a focus on the effect of disease burden. Science bulletin. 2023;68(13):1430-8. |
| 669 | Lei Q, Wang X, Wu C, Bi J, Zhang L. Superior mesenteric artery syndrome caused by surgery and radiation therapy for a brain tumor: A case report. Experimental and Therapeutic Medicine. 2015;10(4):1578-80. doi: 10.3892/etm.2015.2691. |
| 670 | Lehman RA, Krishnamurthy S, Berlin CM. Weight and height deficits in children with brain stem tumors. Clinical pediatrics. 2002;41(5):315-21. Epub 2002/06/28. doi: 10.1177/000992280204100504. PubMed PMID: 12086197. |
| 671 | Lefranc F, Rynkowski M, DeWitte O, Kiss R. Present and potential future adjuvant issues in high-grade astrocytic glioma treatment. Advances and technical standards in neurosurgery. 2009;34:3-35. Epub 2009/04/17. doi: 10.1007/978-3-211-78741-0_1. PubMed PMID: 19368079. |
| 672 | Lee V, Smith TW, Arikan MÇ, Zhang L, Çataltepe O, Çataltepe S. Fatty Acid-binding Protein 4 Expression in Tumor Cells as a Potential Marker for Anaplastic Meningiomas. Applied Immunohistochemistry and Molecular Morphology. 2021;29(2):e10-e6. doi: 10.1097/PAI.0000000000000848. |
| 673 | Lee SY, Slagle-Webb B, Rizk E, Miller P, Zhang X, Ponnuru P, et al. Development of antitumor compounds for temozolomide-resistant tumors. Neuro-oncology. 2009;11(5):595. doi: 10.1215/15228517-2009-034. |
| 674 | Lee M, Hoyt C, Rodgers GM. ADAMTS13 expressed in glioblastoma and astrocytoma cells can modulate endothelial cell migration. Blood. 2013;122(21). |
| 675 | Lee JS, Lee JY, Kim KH, Park SH, Koh EJ, Kim SK, et al. The Role of Early and Delayed Surgery for Infants with Congenital Brain Tumors. Cancer research and treatment. 2024;56(3):909-19. |
| 676 | Lee EJ, Kim JH, Park ES, Kim YH, Lee JK, Hong SH, et al. A novel weighted scoring system for estimating the risk of rapid growth in untreated intracranial meningiomas. Journal of neurosurgery. 2017;127(5):971-80. Epub 2017/01/14. doi: 10.3171/2016.9.Jns161669. PubMed PMID: 28084908. |
| 677 | Lee C, Fotovati A, Verraeult M, Wakimoto H, Reynolds B, Dunham C, et al. Targeting polo-like kinase for the treatment of brain tumors and its unique capacity to eliminate cancer stem cells through Sox-2 inhibition. Neuro-oncology. 2011;13:i9. doi: 10.1093/neuonc/nor018. |
| 678 | Lee AG, Wall M. Papilledema: are we any nearer to a consensus on pathogenesis and treatment? Current neurology and neuroscience reports. 2012;12(3):334-9. Epub 2012/02/23. doi: 10.1007/s11910-012-0257-8. PubMed PMID: 22354546. |
| 679 | Lee A, Oley F, Jr., Lo M, Fong R, McGann M, Saunders I, et al. Direct oral anticoagulants or low-molecular-weight heparins for venous thromboembolism in patients with brain tumors. Thrombosis research. 2021;208:148-55. |
| 680 | Lee A, Oley F, Lo M, Fong R, McGann M, Saunders I, et al. Direct Oral Anticoagulants (DOACs) Vs. Low Molecular Weight Heparins (LMWH) for Venous Thromboembolism (VTE) in Patients with Primary Brain Tumors or Secondary Brain Metastases. Blood. 2020;136:6. . |
| 681 | Lebrun L, Allard-Demoustiez S, Gilis N, Van Campenhout C, Rodesch M, Roman C, et al. Clinicopathological and molecular characterization of a case classified by DNA‑methylation profiling as "CNS embryonal tumor with BRD4-LEUTX fusion". Acta neuropathologica communications. 2023;11(1):46. |
| 682 | LeBlanc Z, Watts R. Complex pathophysiology of hyponatremiawith brain tumor chemotherapy, an illustrative case presentation. Journal of Investigative Medicine. 2013;61(2):467. doi: 10.231/JIM.0b013e3182820c55. |
| 683 | Lebbink CA, Ringers TP, Schouten-van Meeteren AYN, Van Iersel L, Clement SC, Boot AM, et al. Prevalence and Risk Factors of Hypothalamic- Pituitary Dysfunction in Infant and Toddler Brain Tumor Survivors. Journal of the Endocrine Society. 2021;5:A719. doi: 10.1210/jendso/bvab048.1463. |
| 684 | Lebbink CA, Ringers TP, Schouten-van Meeteren AYN, van Iersel L, Clement SC, Boot AM, et al. Prevalence and risk factors of hypothalamic-pituitary dysfunction in infant and toddler childhood brain tumor survivors. European journal of endocrinology. 2021;185(4):597-606. |
| 685 | Leader A, Hamulyák EN, Carney BJ, Avrahami M, Knip JJ, Rozenblatt S, et al. Intracranial hemorrhage with direct oral anticoagulants in patients with brain metastases. Research and Practice in Thrombosis and Haemostasis. 2020;4(SUPPL 1):1054-5. doi: 10.1002/rth2.12393. |
| 686 | Leader A, Hamulyák E, Wang T, Baumann Kreuziger L, Carney B, Hoeben A, et al. Multinational Cohort Analysis of Intracranial Hemorrhage Outcomes in Patients with Brain Tumors Receiving Anticoagulation (ABC Study). Research and Practice in Thrombosis and Haemostasis. 2023;7. doi: 10.1016/j.rpth.2023.100611. |
| 687 | Le Roux E, Menesguen F, Tejedor I, Popelier M, Halbron M, Faucher P, et al. Studying the Care and Social Pathway of Young Adults With Endocrine and Metabolic Diseases During Transition: The Transend Cohort. Journal of the Endocrine Society. 2020;4:A1078-A9. doi: 10.1210/jendso/bvaa046.2138. |
| 688 | Lasky JL, Patel R, Liau LM. Immunotherapeutic targeting of brain tumor stem cells. Neuro-oncology. 2009;11(5):619. doi: 10.1215/15228517-2009-034. |
| 689 | Lasica N, Djilvesi D, Papic V, Karan M, Jelaca B, Golubovic J, et al. Venous thromboembolic and hemorrhagic events after meningioma surgery: A single-center retrospective cohort study of risk factors. PloS one. 2022;17(8):e0273189. |
| 690 | Larsson C, Kleppestø M, Grothe I, Vardal J, Bjørnerud A. T1 in high-grade glioma and the influence of different measurement strategies on parameter estimations in DCE-MRI. Journal of magnetic resonance imaging : JMRI. 2015;42(1):97-104. |
| 691 | Laprie A, Noel G, Chaltiel L, Truc G, Sunyach M, Charissoux M, et al. Dose-painting multicenter phase III trial in newly diagnosed glioblastoma: the SPECTRO-GLIO trial. Radiotherapy and Oncology. 2021;161:S246-S7. doi: 10.1016/S0167-8140(21)06866-3. |
| 692 | Lapierre V, Mahé C, Aupérin A, Stambouli F, Oubouzar N, Tramalloni D, et al. Platelet transfusion containing ABO-incompatible plasma and hepatic veno-occlusive disease after hematopoietic transplantation in young children. Transplantation. 2005;80(3):314-9. |
| 693 | Langlands JM, Kanekal S, Steino A, Mendez A, Karman Z, Toth BM, et al. Dianhydrogalactitol (VAL-083) for the treatment of glioblastoma multiforme (GBM): Impact of glucose transporters for crossing the blood brain barrier (BBB). Cancer research. 2022;82(12). |
| 694 | Landrigan PJ, Raps H, Cropper M, Bald C, Brunner M, Canonizado EM, et al. The Minderoo-Monaco Commission on Plastics and Human Health. Annals of global health. 2023;89(1):23. Epub 2023/03/28. doi: 10.5334/aogh.4056. PubMed PMID: 36969097; PubMed Central PMCID: PMC10038118. |
| 695 | Landau E, Boop FA, Merchant TE. Supratentorial ependymoma: Disease control and functional outcomes after irradiation. International Journal of Radiation Oncology Biology Physics. 2011;81(2):S657. |
| 696 | Lakshmi A, Alagarsamy M, Anbarasa Pandian A, Paramathi Mani D. Evolutionary gravitational neocognitron neural network optimized with marine predators optimization algorithm for MRI brain tumor classification. Electromagnetic biology and medicine. 2024;43(1-2):1-18. |
| 697 | Lakomkin N, Stannard B, Hadjipanayis C. The safety and feasibility of brain tumor resection in the outpatient setting-a pilot study. Journal of neurosurgery. 2020;132(4):15. doi: 10.3171/2020.4.JNS.AANS2020abstracts. |
| 698 | Lakomkin N, Hadjipanayis CG. Non-routine discharge disposition is associated with post-discharge complications and 30-day readmissions following craniotomy for brain tumor resection. Journal of neuro-oncology. 2018;136(3):595-604. |
| 699 | Lake J, Donson A, Witt D, Foreman N, Hoffman L. A low-grade glioma histologically diagnosed as an ependymoma: How molecular data is shaping the way we diagnosis and treat brain tumors. Pediatric Blood and Cancer. 2018;65:S22-S3. doi: 10.1002/pbc.27057. |
| 700 | Ladha H, Pawar T, Gilbert MR, Armstrong TS. Wound healing complications in brain tumor patients on bevacizumab. Neuro-oncology. 2014;16:ii73. doi: 10.1093/neuonc/nou174. |
| 701 | Ladas E, Buck C, Arbit N. A cross-sectional survey exploring behaviors and psychosocial determinants of physical activity and diet in children with brain tumors (BT). Neuro-oncology. 2014;16:i113. doi: 10.1093/neuonc/nou079. |
| 702 | Kwon OI, Lee MB, Jahng GH. High frequency conductivity decomposition by solving physically constraint underdetermined inverse problem in human brain. Scientific reports. 2023;13(1):3273. |
| 703 | Kuzior A, Hernandez-Lazaro A, De Leon-Durango R, Rios-Gomez C, Arnas-Leon C, Acosta-Calero C, et al. DOES GENDER-AFFIRMING HORMONAL THERAPY CAUSE A SIGNIFICANT RISE OF BLOOD PRESSURE IN YOUNG TRANSGENDER PEOPLE? Journal of hypertension. 2022;40:e204. |
| 704 | Kusuda K, Okamoto J, Horise Y, Tamura M, Kobayashi E, Muragaki Y, et al. Clinical Information Analyzer system to support surgery toward realization of AI surgery. International journal of computer assisted radiology and surgery. 2019;14:S129-S30. doi: 10.1007/s11548-019-01969-3. |
| 705 | Kuruppu D, Bhere D, Farrar CT, Kill K, Zhu A, Shah K, et al. Novel oncolytic HSV-1 therapeutics for breast cancer meningeal metastases. Molecular imaging and biology. 2015;17(1). doi: 10.1007/s11307-014-0809-1. |
| 706 | Kuroiwa T, Ohki J, Tabata H. Peritumoral atrophy and neuronal deformation of meningioma. Neuro-oncology. 2017;19:iii35. doi: 10.1093/neuonc/nox036. |
| 707 | Kurmasheva R, Kolb EA, Smith MA, Teicher BA, Erickson SW, Maris JM, et al. Initial testing of m276-PBD CD276 antibody-drug conjugate in preclinical models of pediatric cancers by the Pediatric Preclinical Testing Consortium (PPTC). Molecular cancer therapeutics. 2019;18(12 SUPPL 1). doi: 10.1158/1535-7163.TARG-19-C003. |
| 708 | Kurita N, Kawaguchi M, Nakahashi K, Sakamoto N, Horiuchi T, Takahashi M, et al. [Retrospective analysis of postoperative nausea and vomiting after craniotomy]. Masui The Japanese journal of anesthesiology. 2004;53(2):150-5. |
| 709 | Kuon E, Birkel J, Schmitt M, Dahm JB. Radiation exposure benefit of a lead cap in invasive cardiology. Heart (British Cardiac Society). 2003;89(10):1205-10. Epub 2003/09/17. doi: 10.1136/heart.89.10.1205. PubMed PMID: 12975420; PubMed Central PMCID: PMC1767881. |
| 710 | Kuo YH, Edgar MA, Luther N, Schwartz TH. Novel low-grade glioneuronal neoplasm presenting in an octogenarian: case report and review of the literature. Clinical neurology and neurosurgery. 2006;108(4):426-32. Epub 2006/06/08. doi: 10.1016/j.clineuro.2005.05.006. PubMed PMID: 16758540. |
| 711 | Kuo YC, Chao IW. Conjugation of melanotransferrin antibody on solid lipid nanoparticles for mediating brain cancer malignancy. Biotechnology progress. 2016;32(2):480-90. Epub 2015/12/25. doi: 10.1002/btpr.2214. PubMed PMID: 26701338. |
| 712 | Kumar SS, Sengupta S, Zhu X, Mishra DK, Phoenix T, Dyer L, et al. Diffuse intrinsic pontine glioma cells are vulnerable to mitotic abnormalities associated with BMI-1 modulation. Molecular Cancer Research. 2020;18(11):1711-23. doi: 10.1158/1541-7786.MCR-20-0099. |
| 713 | Kumar SS, Sengupta S, Zhu X, Mishra DK, Fuller C, Fouladi M, et al. Induction of mitotic abnormalities and BMI-1 modulation to treat diffuse intrinsic pontine glioma. Neuro-oncology. 2020;22(SUPPL 3):iii291. doi: 10.1093/neuonc/noaa222. |
| 714 | Kumar SS, Sengupta S, Weetall M, Baird J, Drissi R. BMI-1 modulation impairs DNA damage response and sensitizes DIPG cells to ionizing radiation. Neuro-oncology. 2019;21:AA116. doi: 10.1093/neuonc/noz036.218. |
| 715 | Kumar SS, Sengupta S, Lee K, Hura N, Fuller C, DeWire M, et al. BMI-1 is a potential therapeutic target in diffuse intrinsic pontine glioma. Oncotarget. 2017;8(38):62962-75. doi: 10.18632/oncotarget.18002. |
| 716 | Kumar SS, Sengupta S, Lee K, Hura N, Drissi R. Validation of BMI-1 as a therapeutic target in diffuse intrinsic pontine glioma. Neuro-oncology. 2017;19:iv8. doi: 10.1093/neuonc/nox083. |
| 717 | Kumar SS, Mishra D, McHugh T, Drissi R. INDUCED MITOTIC ABNORMALITIES ASSOCIATED WITH BMI-1 MODULATION SENSITIZE DIFFUSE INTRINSIC PONTINE GLIOMA CELLS TO IONIZING RADIATION. Neuro-oncology. 2022;24:i20. doi: 10.1093/neuonc/noac079.069. |
| 718 | Kumar R, Giri PJ, Jaiswal A, Agarwal T, Pal L. Intracranial Rosai-Dorfman syndrome mimicking meningioma. Pan Arab Journal of Neurosurgery. 2008;12(1):70-2+126+5. |
| 719 | Kumar P, Garg N. Flow Cytometry Approaches to Obtain Medulloblastoma Stem Cells from Bulk Cultures. Methods in molecular biology (Clifton, NJ). 2022;2423:87-94. Epub 2022/01/04. doi: 10.1007/978-1-0716-1952-0_8. PubMed PMID: 34978690. |
| 720 | Kumar KS, Brunner C, Schuster M, Zerbe O, Grotzer M, Schneider G, et al. Small molecule targeting of oncogenic FGF2-FGFR signaling in brain tumors. Neuro-oncology. 2020;22(SUPPL 3):iii413-iii4. doi: 10.1093/neuonc/noaa222. |
| 721 | Küker W, Friese S, Krapf H, Ernemann U. Chronic intracranial hypertension due to a tumor of the sinus wall: MRI and angiography. Klinische Neuroradiologie. 2000;10(1):23-8. |
| 722 | Kuijten RR, Bunin GR, Nass CC, Meadows AT. Gestational and familial risk factors for childhood astrocytoma: results of a case-control study. Cancer research. 1990;50(9):2608-12. Epub 1990/05/01. PubMed PMID: 2328486. |
| 723 | Kryzhanovskaya I. A fortuitous find in the work-up of a chronic cough. Journal of general internal medicine. 2015;30:S308-S9. |
| 724 | Krysiak R, Marek B, Okopień B. [Central precocious puberty]. Endokrynologia Polska. 2008;59(6):530-40. Epub 2008/01/01. PubMed PMID: 19347819. |
| 725 | Krona C, Dyberg C, Sandén E, Darabi A, Wickström M, Northcott P, et al. Stereotactic injections of primary medulloblastomas as a representative model of the different subgroups for preclinical drug screening. Cancer research. 2014;74(20). doi: 10.1158/1538-7445.PEDCAN-A84. |
| 726 | Krishna KB, Tas E, Popovic J. Dysembryoplastic neuroepithelial tumor (DNET) in a prepubertal boy with noonan syndrome(NS) receiving growth hormone (GH) therapy. Should brain MRI be routinely performed in children with NS prior to initiating GH therapy? Endocrine Reviews. 2014;35. |
| 727 | Kremer P, Mahmoudreza F, Ding R, Pritsch M, Zoubaa S, Frei E. Intraoperative fluorescence staining of malignant brain tumors using 5-aminofluorescein-labeled albumin. Neurosurgery. 2009;64(3 SUPPL.):ons53-ons60. |
| 728 | Kreitschmann-Andermahr I, Siegel S, Thissen A, Rosenkranz E, Piroth MD, Mertens R, et al. Endocrine sequelae, sleep and psychosocial impairment in adult patients after multimodal treatment for brain tumors or leukemia. Endocrine Reviews. 2012;33(3). |
| 729 | Koyee M, Mohana Rupa K, Bhavana U, Greeshma, Ganesh MV. Parietal meningioma excision under hypotensive anaesthesia in a patient with a prosthetic mitral valve. Journal of Clinical and Diagnostic Research. 2011;5(8):1677-8. |
| 730 | Koutnik AP, Poff AM, Ward NP, Ramirez M, D'Agostino DP. Establishing that the VM-M3 model of metastatic cancer exhibits cancer cachexia. FASEB Journal. 2016;30. |
| 731 | Kong Y, Ai C, Dong F, Xia X, Zhao X, Yang C, et al. Targeting of BMI-1 with PTC-209 inhibits glioblastoma development. Cell cycle (Georgetown, Tex). 2018;17(10):1199-211. Epub 2018/06/12. doi: 10.1080/15384101.2018.1469872. PubMed PMID: 29886801; PubMed Central PMCID: PMC6110607. |
| 732 | Komolafe MA, Sunmonu TA, Oke O. Stroke-like syndrome in a middle aged Nigerian woman with metastatic brain cancer. West African journal of medicine. 2009;28(4):266-9. Epub 2010/04/29. PubMed PMID: 20425745. |
| 733 | Komarowska M, Chrzanowski R, Tylicka M, Rutkowski R, Mariak Z, Zelazowska-Rutkowska B, et al. Plasma concentration of Bisphenol A and leptin in patients with meningioma and glioma: A pilot study. Advances in medical sciences. 2022;67(2):229-33. |
| 734 | Koloušková S, Zemková D, Cháňová M, Mališ J, Šnajderová M. Retrospective analysis of growth in tumors of hypothalamus-hypophysis region. Cesko-Slovenska Pediatrie. 2005;60(6):345-8. |
| 735 | Kolakshyapati M, Ikawa F, Abiko M, Mitsuhara T, Kinoshita Y, Takeda M, et al. Multivariate risk factor analysis and literature review of postoperative deterioration in Karnofsky Performance Scale score in elderly patients with skull base meningioma. Neurosurgical focus. 2018;44(4):E14. |
| 736 | Kokkinakis DM, Schold SC, Jr., Hori H, Nobori T. Effect of long-term depletion of plasma methionine on the growth and survival of human brain tumor xenografts in athymic mice. Nutrition and cancer. 1997;29(3):195-204. Epub 1997/01/01. doi: 10.1080/01635589709514624. PubMed PMID: 9457739. |
| 737 | Koeppen S, Hense J. Rechallenge with bevacizumab in a long-term survivor with glioblastoma. Neuro-oncology. 2016;18:iv51-iv2. |
| 738 | Kobayashi K, Suehiro M, Maihara T, Usami I, Kageyama Y, Okazaki S, et al. Cerebral Sinovenous Thrombosis and Subdural Hematoma as Treatment-Related Complications in Suprasellar Germ Cell Tumor Associated with Adipsic Diabetes Insipidus. Pediatric neurosurgery. 2019;54(4):288-92. |
| 739 | Kobata T, Maeda Y, Monden T, Morimoto M, Oomori K. [Investigation of Radioactivity Concentration of the Normal Brain Region for the Phantom Experiment in Brain Tumor PET Imaging]. Nihon Hoshasen Gijutsu Gakkai zasshi. 2019;75(8):771-6. |
| 740 | Ko CC, Lim SW, Chen TY, Chen JH, Li CF. Prediction of early progression in skull base meningiomas: Additional benefits of diffusion-weighted MRI maging. Neuroradiology. 2018;60(1):308-10. |
| 741 | Knovich MA, Lesser GJ. The management of thromboembolic disease in patients with central nervous system malignancies. Current treatment options in oncology. 2004;5(6):511-7. Epub 2004/10/29. doi: 10.1007/s11864-004-0039-x. PubMed PMID: 15509484. |
| 742 | Kline C, Morimoto L, Wiemels J, Metayer C, Walsh K. Pre-and perinatal factors associated with development of pediatric ependymoma: Results of case-control analysis. Neuro-oncology. 2017;19:iv17. doi: 10.1093/neuonc/nox083. |
| 743 | Kleinschmidt-De Masters B, Serbenou D, Turin C. Plasmacytoma: Yet Another Rare Sellar Region Mass. Journal of Neuropathology and Experimental Neurology. 2024;83(6):538. doi: 10.1093/jnen/nlae036. |
| 744 | Klauschie J, Rose SR. Incidence of short stature in children with hydrocephalus. Journal of pediatric endocrinology & metabolism : JPEM. 1996;9(2):181-7. Epub 1996/03/01. PubMed PMID: 8887141. |
| 745 | Klap BC, Te Winkel ML, Den Hoed M, Van Waas M, Neggers SJCMM, Boot AM, et al. Prediction of impaired bone mineral density in long-term adult survivors of childhood cancer. Pediatric Blood and Cancer. 2013;60:163. doi: 10.1002/pbc.24719. |
| 746 | Kitahara CM, Gamborg M, Rajaraman P, Sørensen TI, Baker JL. A prospective study of height and body mass index in childhood, birth weight, and risk of adult glioma over 40 years of follow-up. American journal of epidemiology. 2014;180(8):821-9. |
| 747 | Kirman CR, Gargas ML, Marsh GM, Strother DE, Klaunig JE, Collins JJ, et al. Cancer dose-response assessment for acrylonitrile based upon rodent brain tumor incidence: Use of epidemiologic, mechanistic, and pharmacokinetic support for nonlinearity. Regulatory Toxicology and Pharmacology. 2005;43(1):85-103. |
| 748 | Kimura H, Nakagawa K, Sakaki S, Matsuoka K. [Intracranial meningioma of an infant: a case report]. No shinkei geka Neurological surgery. 1987;15(6):663-8. Epub 1987/06/01. PubMed PMID: 3313088. |
| 749 | Kim Y, Kumar S. CD44-mediated adhesion to hyaluronic acid contributes to mechanosensing and invasive motility. Molecular cancer research : MCR. 2014;12(10):1416-29. Epub 2014/06/26. doi: 10.1158/1541-7786.Mcr-13-0629. PubMed PMID: 24962319; PubMed Central PMCID: PMC4201971. |
| 750 | Kim Y, Kang H, Powathil G, Kim H, Trucu D, Lee W, et al. Role of extracellular matrix and microenvironment in regulation of tumor growth and LAR-mediated invasion in glioblastoma. PloS one. 2018;13(10):e0204865. |
| 751 | Kim KM, Shim JK, Chang JH, Lee JH, Kim SH, Choi J, et al. Failure of a patient-derived xenograft for brain tumor model prepared by implantation of tissue fragments. Cancer Cell International. 2016;16(1). doi: 10.1186/s12935-016-0319-0. |
| 752 | Kim KH, Seo Y, Kim SH. Efficacy of Hydroxyapatite Using Skull Base Reconstruction Performed by Neurosurgeons in the Learning Curve. Journal of Neurological Surgery, Part B Skull Base. 2023;84. doi: 10.1055/s-0043-1762431. |
| 753 | Kim K, Cho C, Bang MS, Shin HI, Phi JH, Kim SK. Intraoperative neurophysiological monitoring: A review of techniques used for brain tumor surgery in children. Journal of Korean Neurosurgical Society. 2018;61(3):363-75. doi: 10.3340/jkns.2018.0078. |
| 754 | Kim JH, Yoon HK, Lee HC, Park HP, Park CK, Dho YS, et al. Preoperative 5-aminolevulinic acid administration for brain tumor surgery is associated with an increase in postoperative liver enzymes: a retrospective cohort study. Acta neurochirurgica. 2019;161(11):2289-98. |
| 755 | Kim CJ, Song KH, Kang CK, Choe PG, Bae JY, Choi HJ, et al. Predicting central nervous system complications in staphylococcus aureus bacteremia using clinical scoring system. Open Forum Infectious Diseases. 2018;5:S315-S6. doi: 10.1093/ofid/ofy210.893. |
| 756 | Kim BR, Chun MH, Han EY. The assessment and rehabilitation outcomes on fatigue in patients with brain tumors. PM and R. 2010;2(9):S19-S20. |
| 757 | Kim A, Moon JS, Yang HR, Chang JY, Ko JS, Seo JK. Diencephalic syndrome: A frequently neglected cause of failure to thrive in infants. Korean Journal of Pediatrics. 2015;58(1):28-32. doi: 10.3345/kjp.2015.58.1.28. |
| 758 | Kilinc F, Setzer M, Prinz V, Jussen D, Marquardt G, Gessler F, et al. The Beneficial Effect of Preoperative Exercise on Postoperative Clinical Outcome, Quality of Life and Return to Work after Microsurgical Resection of Spinal Meningiomas. Journal of Clinical Medicine. 2023;12(8). doi: 10.3390/jcm12082804. |
| 759 | Kilinc F, Setzer M, Marquardt G, Keil F, Dubinski D, Bruder M, et al. Functional outcome and morbidity after microsurgical resection of spinal meningiomas. Neurosurgical focus. 2021;50(5):E20. Epub 2021/05/02. doi: 10.3171/2021.2.Focus201116. PubMed PMID: 33932928. |
| 760 | Khouri T, Sidhar K. Foot Drop in the Young Runner. Clinical Journal of Sport Medicine. 2024;34(2):e24. doi: 10.1097/JSM.0000000000001210. |
| 761 | Khatiwala R, Peris-Celda M, Kenning T, Pinheiro-Neto C. Endoscopic endonasal reconstruction of high-flow cerebrospinal fluid leak with fascia lata “button” graft and nasoseptal flap: Surgical technique and case series. Journal of Neurological Surgery, Part B: Skull Base. 2018;79. doi: 10.1055/s-0038-1633625. |
| 762 | Khan S, Siddiqui K, Bakhshi SK, Shamim MS. Venous thromboembolism after meningioma resection. JPMA The Journal of the Pakistan Medical Association. 2021;71(10):2477-8. Epub 2022/01/03. PubMed PMID: 34974600. |
| 763 | Khan RB, Merchant TE, Sadighi ZS, Crabtree VM, Bello M, Sykes A, et al. Risk factors for hypersomnia and narcolepsy and response to therapy in survivors of childhood brain tumors. Neuro-oncology. 2017;19:iii4. doi: 10.1093/neuonc/nox036. |
| 764 | Khan R, Bano M, Mirman I, Wang F, Haitao P, Smith J, et al. Height, weight and cardiovascular effects of stimulants on children with brain tumor. Neuro-oncology. 2020;22(SUPPL 3):iii440. doi: 10.1093/neuonc/noaa222. |
| 765 | Khan KJ, Gevorgyan AG, Sahai-Srivastava S, Richeimer S. Indomethacin responsive postdural puncture headache with a paroxysmal hemicrania phenotype. Headache. 2016;56:76-8. doi: 10.1111/head.12832. |
| 766 | Khan AM, Chiasakul T, Redd R, Patell R, McCarthy EP, Neuberg D, et al. Survival outcomes with warfarin compared with direct oral anticoagulants in cancer-associated venous thromboembolism in the United States: A population-based cohort study. PLoS medicine. 2022;19(5):e1004012. |
| 767 | Khan A, Winkfield B, Khan S, Zafar R, Amer I. HEMORRHAGIC CYSTIC BRAIN LESIONS AS INITIAL PRESENTATION OF SMALL CELL LUNG CANCER. Chest. 2020;158(4):A1426. doi: 10.1016/j.chest.2020.08.1291. |
| 768 | Khammad VA, Otero JJ, Guerra FJG, Izquierdo YC, Becker AP, Kharchenko NV, et al. Machine learning applications in R for the differentiation of primary CNS tumors. FASEB Journal. 2020;34(SUPPL 1). doi: 10.1096/fasebj.2020.34.s1.01778. |
| 769 | Kessous R, Wainstock T, Sheiner E. Pre-pregnancy obesity and childhood malignancies: A population-based cohort study. Pediatric blood & cancer. 2020;67(6):e28269. Epub 2020/03/21. doi: 10.1002/pbc.28269. PubMed PMID: 32196946. |
| 770 | Kayali H, Onguru O, Erdogan E, Sirin S, Timurkaynak E. Isolated intracranial Rosai-Dorfman disease mimicking meningioma. Clinical neuropathology. 2004;23(5):204-8. Epub 2004/12/08. PubMed PMID: 15581022. |
| 771 | Kayadjanian N, Hsu EA, Wood AM, Carson DS. Caregiver Burden and Its Relationship to Health-Related Quality of Life in Craniopharyngioma Survivors. The Journal of clinical endocrinology and metabolism. 2023;109(1):e76-e87. |
| 772 | Kawasaki S, Misawa H, Kondo T, Kondo Y, Terauchi Y. Risk factors for sleep apnea syndrome in nonobese type 2 diabetics. Diabetes. 2019;68. doi: 10.2337/db19-1619-P. |
| 773 | Kawasaki S, Misawa H, Kaneda R, Shizuku T, Tamura Y, Kondo T, et al. Type 2 diabetes is complicated by sleep apnoea syndrome: focussing in comorbidities. Diabetologia. 2018;61:S514-S5. doi: 10.1007/s00125-018-4693-0. |
| 774 | Kawasaki S, Misawa H, Kaneda R, Kondo T, Kondo Y, Terauchi Y. Risk factors for sleep apnea syndrome in diabetic patients with a focus on comorbidities. Diabetes. 2018;67:A435. |
| 775 | Kato Y, Holm DA, Okollie B, Artemov D. Noninvasive detection of temozolomide in brain tumor xenografts by magnetic resonance spectroscopy. Neuro-oncology. 2010;12(1):71-9. Epub 2010/02/13. doi: 10.1093/neuonc/nop006. PubMed PMID: 20150369; PubMed Central PMCID: PMC2940553. |
| 776 | Karri M, Annavarapu CSR, Acharya UR. Explainable multi-module semantic guided attention based network for medical image segmentation. Computers in biology and medicine. 2022;151. doi: 10.1016/j.compbiomed.2022.106231. |
| 777 | Karhade AV, Fandino L, Gupta S, Cote DJ, Iorgulescu JB, Broekman ML, et al. Impact of operative length on post-operative complications in meningioma surgery: a NSQIP analysis. Journal of neuro-oncology. 2017;131(1):59-67. |
| 778 | Karayegen G, Aksahin MF. Brain tumor prediction on MR images with semantic segmentation by using deep learning network and 3D imaging of tumor region. Biomedical Signal Processing and Control. 2021;66. doi: 10.1016/j.bspc.2021.102458. |
| 779 | Kapoor-Narula U, Lenka N. Elucidating the Anti-Tumorigenic Efficacy of Oltipraz, a Dithiolethione, in Glioblastoma. Cells. 2022;11(19). Epub 2022/10/15. doi: 10.3390/cells11193057. PubMed PMID: 36231019; PubMed Central PMCID: PMC9562012. |
| 780 | Kanno H, Kuwabara T, Yasumitsu H, Umeda M. Transforming growth factors in urine from patients with primary brain tumors. Journal of neurosurgery. 1988;68(5):775-80. Epub 1988/05/01. doi: 10.3171/jns.1988.68.5.0775. PubMed PMID: 3258633. |
| 781 | Kamson D, Yuen C, Castillo-Saavedra L, Wilmington A, Walker M, Merrell R. Patient parameters associated with tumor growth in incidental meningioma. Neuro-oncology. 2018;20:vi148. doi: 10.1093/neuonc/noy148. |
| 782 | Kamoun WS, Ley CD, Farrar CT, Duyverman AM, Lahdenranta J, Lacorre DA, et al. Edema control by cediranib, a vascular endothelial growth factor receptor-targeted kinase inhibitor, prolongs survival despite persistent brain tumor growth in mice. Journal of clinical oncology : official journal of the American Society of Clinical Oncology. 2009;27(15):2542-52. |
| 783 | Kamath D, Bell D, Lorenzana A. Rosai dorfman and chronic myelomonocytic leukemia: Secondary neoplasms post medulloblastoma treatment. Pediatric Blood and Cancer. 2020;67(SUPPL 2). doi: 10.1002/pbc.28321. |
| 784 | Kamarudin MN, Wan Hitam WH, Tajuddin LSA. Foster kennedy syndrome secondary to sphenoid wing meningioma. Neuro-Ophthalmology. 2012;36:26-7. doi: 10.3109/01658107.2012.690640. |
| 785 | Kamal S, Abbassi MM, Abouelnaga S, Agha AM. Pilot study: Valproic acid effectiveness in minimizing incidence of seizures in postoperative pediatric brain tumor patients. Pharmacotherapy. 2012;32(10):e223. doi: 10.1002/j.1875-9114.2012.01219. |
| 786 | Kalakoti P, Edwards A, Ferrier C, Sharma K, Huynh T, Ledbetter C, et al. Biomarkers of Seizure Activity in Patients With Intracranial Metastases and Gliomas: A Wide Range Study of Correlated Regions of Interest. Frontiers in Neurology. 2020;11. doi: 10.3389/fneur.2020.00444. |
| 787 | Kala R, Deepa P. Segmentation of brain magnetic resonance images using deep learning classification and multi-modal composition. Current Signal Transduction Therapy. 2020;15(2):94-108. doi: 10.2174/1574362414666181220105908. |
| 788 | Jussila MP, Remes T, Anttonen J, Harila-Saari A, Niinimäki J, Pokka T, et al. Late vertebral side effects in long-term survivors of irradiated childhood brain tumor. PloS one. 2018;13(12):e0209193. |
| 789 | Jung IH, Yoo J, Roh TH, Park HH, Hong CK. Importance of Sufficient Petrosectomy in an Anterior Petrosal Approach: Relightening of the Kawase Pyramid. World neurosurgery. 2021;153:e11-e9. Epub 2021/05/19. doi: 10.1016/j.wneu.2021.05.017. PubMed PMID: 34004357. |
| 790 | Jugl S, Chen S, Antonelli PJ, Winterstein AG. Risk of intracranial meningioma requiring surgical intervention among recent hormonal contraceptives users. Pharmacoepidemiology and Drug Safety. 2021;30(SUPPL 1):329. doi: 10.1002/pds.5305. |
| 791 | Joó GJ, Reiniger L, Papp C, Csaba Á, Komáromy H, Rigó J. Intrauterine diagnosis and pathology of fetal choroid plexus carcinoma - A case study. 2014;210(12):1156-9. doi: 10.1016/j.prp.2014.01.009. |
| 792 | Jones LW, Guill B, Keir ST, Carter K, Friedman HS, Bigner DD, et al. Using the theory of planned behavior to understand the determinants of exercise intention in patients diagnosed with primary brain cancer. Psycho-oncology. 2007;16(3):232-40. |
| 793 | Johnson MD, Woodard A, Okediji EJ, Toms SA, Allen GS. Lovastatin is a potent inhibitor of meningioma cell proliferation: evidence for inhibition of a mitogen associated protein kinase. Journal of neuro-oncology. 2002;56(2):133-42. |
| 794 | Johnson KJ, Zoellner NL, Gutmann DH. Peri-gestational risk factors for pediatric brain tumors in Neurofibromatosis Type 1. Cancer epidemiology. 2016;42:53-9. Epub 2016/03/29. doi: 10.1016/j.canep.2016.03.005. PubMed PMID: 27018750; PubMed Central PMCID: PMC4899111. |
| 795 | Johnson K. Uterine fibroids, BMI may flag postmenopausal meningioma risk. Oncology Report. 2011(JANUARY):13. |
| 796 | Johnson AH, Rodgers Phillips S, Rice M. Abnormal weight gain with fatigue and stress in early survivorship after childhood brain tumor diagnosis. Journal for specialists in pediatric nursing : JSPN. 2020;25(3):e12288. Epub 2020/02/18. doi: 10.1111/jspn.12288. PubMed PMID: 32065725. |
| 797 | Jo JT, Schiff D, Perry JR. Thrombosis in brain tumors. Seminars in thrombosis and hemostasis. 2014;40(3):325-31. Epub 2014/03/07. doi: 10.1055/s-0034-1370791. PubMed PMID: 24599439. |
| 798 | Jin R, Accortt NA, Sandschafer D, Lawrence T, Loaiza-Bonilla A. Initial experience of patientstreated in a real-world clinicalsetting with bevacizumab-awwb:The first FDA-approved biosimilarto bevacizumab. Journal of Clinical Oncology. 2021;39(3 SUPPL). doi: 10.1200/JCO.2021.39.3_suppl.81. |
| 799 | Jiang M, Zhai F, Kong J. A novel deep learning model DDU-net using edge features to enhance brain tumor segmentation on MR images. Artificial intelligence in medicine. 2021;121:102180. Epub 2021/11/13. doi: 10.1016/j.artmed.2021.102180. PubMed PMID: 34763802. |
| 800 | Jhalani A, Gupta MK, Sharma N, Agrawal D, Khinchi MP. Brain tumor stem cell: A emerging tool for investigation and inhibition of tumor genesis in brain. Research Journal of Pharmaceutical, Biological and Chemical Sciences. 2010;1(3):680-90. |
| 801 | Jeong YI, Song JG, Kang SS, Ryu HH, Lee YH, Choi C, et al. Preparation of poly(DL-lactide-co-glycolide) microspheres encapsulating all-trans retinoic acid. International journal of pharmaceutics. 2003;259(1-2):79-91. Epub 2003/06/06. doi: 10.1016/s0378-5173(03)00207-2. PubMed PMID: 12787638. |
| 802 | Jeon S, Han S, Lee KM, Park AK, Ahn HS, Shin HY, et al. SNPs in apoptosis and cell cycle control related genes and brain tumor risk among children in Korea. Cancer research. 2010;70(8). doi: 10.1158/1538-7445.AM10-2846. |
| 803 | Jensen RL, Wurster RD. Calcium channel antagonists inhibit growth of subcutaneous xenograft meningiomas in nude mice. Surgical neurology. 2001;55(5):275-83. Epub 2001/08/23. doi: 10.1016/s0090-3019(01)00444-x. PubMed PMID: 11516467. |
| 804 | Jean-Pierre P, Grandner M, Jean-Pierre A, Garland SN, Richards E, Maciorowski G. Clinical correlates of memory problems in middle-aged adult-onset cancer survivors in the United States: A national cross-sectional study. Journal of Clinical Oncology. 2014;32(15). |
| 805 | Jasper BW, Conklin HM, Lawford J, Morris EB, Howard SC, Wu S, et al. Growth effects of methylphenidate among childhood cancer survivors: a 12-month case-matched open-label study. Pediatric blood & cancer. 2009;52(1):39-43. |
| 806 | Jarmula J, Merrill T, Woodard T, Kshettry V, Recinos P, Sindwani R. Analysis of Unplanned Surgery following Endoscopic Skull Base Surgery. Journal of Neurological Surgery, Part B: Skull Base. 2024;85. doi: 10.1055/s-0044-1780086. |
| 807 | Jansson S, Khorram-Manesh A, Nilsson O, Kölby L, Tisell LE, Wängberg B, et al. Treatment of bilateral pheochromocytoma and adrenal medullary hyperplasia. Annals of the New York Academy of Sciences. 2006;1073:429-35. Epub 2006/11/15. doi: 10.1196/annals.1353.046. PubMed PMID: 17102111. |
| 808 | Jaga S, Rama Devi K. Brain tumor classification utilizing Triple Memristor Hopfield Neural Network optimized with Northern Goshawk Optimization for MRI image. Biomedical Signal Processing and Control. 2024;95. doi: 10.1016/j.bspc.2024.106450. |
| 809 | Jabeen S, Rehman A, Sultana N, Nazir S, Riaz N, Mahmood Z. Frequency of Endometriosis in Women Undergoing Diagnostic Laparoscopy for Subfertility at Sheikh Zayed Hospital Rahim Yar Khan. Pakistan Journal of Medical and Health Sciences. 2022;16(9):195-7. doi: 10.53350/pjmhs22169195. |
| 810 | Jaan M, Saeed A, Munwar A, Yasmin S, Zaman N, Zafar A. Elevated Serum Ferritin Levels in Gestational Diabetes Mellitus (GDM) at a Tertiary Care Hospital. Pakistan Journal of Medical and Health Sciences. 2023;17(2):637-9. doi: 10.53350/pjmhs2023172637. |
| 811 | Izumoto S, Arita N, Ushio Y, Hayakawa T, Ohnishi T, Taki T, et al. [Effect of MX-2, a morpholino anthracycline derivative, against human and rat glioma cells and experimental leptomeningeal tumors in rats]. Gan to kagaku ryoho Cancer & chemotherapy. 1988;15(5):1765-9. |
| 812 | Iyengar V, Patell R, Zwicker J. Challenges in anticoagulation for patients with brain tumors. Best practice & research Clinical haematology. 2022;35(1):101350. Epub 2022/08/28. doi: 10.1016/j.beha.2022.101350. PubMed PMID: 36030073. |
| 813 | Iyengar V, Agrawal S, Chiasakul T, Tehranchi K, McNichol M, Carney BJ, et al. Comparison of direct oral anticoagulants versus low-molecular-weight heparin in primary and metastatic brain cancers: a meta-analysis and systematic review. Journal of thrombosis and haemostasis : JTH. 2024;22(2):423-9. |
| 814 | Ivan ME, Bryan Iorgulescu J, El-Sayed I, McDermott MW, Parsa AT, Pletcher SD, et al. Risk factors for postoperative cerebrospinal fluid leak and meningitis after expanded endoscopic endonasal surgery. Journal of Clinical Neuroscience. 2015;22(1):48-54. doi: 10.1016/j.jocn.2014.08.009. |
| 815 | Iurlova OV, Dolgikh VV. Perinatal complications in newborns with symptomatic forms of congenital generalized viral infections. European Journal of Paediatric Neurology. 2015;19:S84. |
| 816 | Isobe T, Matsumura A, Anno I, Nagatomo Y, Yoshizawa T, Itai Y, et al. [Changes in 1H-MRS in glioma patients before and after irradiation: the significance of quantitative analysis of choline-containing compounds]. No shinkei geka Neurological surgery. 2003;31(2):167-72. |
| 817 | Isobe N, Ikawa F, Tominaga A, Kuroki K, Sadatomo T, Mizoue T, et al. Factors Related to Frailty Associated with Clinical Deterioration After Meningioma Surgery in the Elderly. World neurosurgery. 2018;119:e167-e73. Epub 2018/07/22. doi: 10.1016/j.wneu.2018.07.080. PubMed PMID: 30031190. |
| 818 | Ishikawa T, Takeuchi K, Nagatani T, Aimi Y, Tanemura E, Tambara M, et al. Quality of Life Changes Before and After Transsphenoidal Surgery for Sellar and Parasellar Lesions. World neurosurgery. 2019;122:e1202-e10. Epub 2018/11/18. doi: 10.1016/j.wneu.2018.11.017. PubMed PMID: 30447458. |
| 819 | Ishihara T, Nogami K, Takeshita Y, Ochi S, Shima M. Fibrinolytic abnormality associated with progression of pediatric solid tumor. Pediatrics international : official journal of the Japan Pediatric Society. 2018;60(6):540-6. Epub 2018/03/06. doi: 10.1111/ped.13546. PubMed PMID: 29505702. |
| 820 | Ioannoni E, Grande G, Olivi A, Antonelli M, Caricato A, Montano N. Factors affecting serum lactate in patients with intracranial tumors - A report of our series and review of the literature. Surgical Neurology International. 2020;11(39). doi: 10.25259/SNI_552_2019. |
| 821 | Inoue T, Ikezaki K, Sato Y. Ultrasonic surgical system (SONOPET) for microsurgical removal of brain tumors. Neurological research. 2000;22(5):490-4. Epub 2000/08/10. doi: 10.1080/01616412.2000.11740706. PubMed PMID: 10935222. |
| 822 | Inaba S, Kudo M, Kamano H, Ohishi Y, Kiyasu J, Watari T. Lymphomatosis cerebri caused by adult T cell leukemia/lymphoma: a differential diagnosis for depression: a case report. Journal of medical case reports. 2024;18(1):348. |
| 823 | Ilievski B, Noveska Petrovska B, Kocmanovska Petreska S, Stojkovski V, Zdravkovski P, Mircevski V. Metastatic ductal breast carcinoma in meningioma: A case report. Virchows Archiv. 2017;471(1):S222-S3. doi: 10.1007/s00428-017-2205-0. |
| 824 | Ikeyama Y, Abiko S, Kurokawa Y, Okamura T, Watanabe K, Inoue S, et al. [Radiation-induced cerebrovasculopathy: a case report and review of the literature]. No shinkei geka Neurological surgery. 1993;21(8):751-7. Epub 1993/08/01. PubMed PMID: 8361576. |
| 825 | Ikeda Y, Sakemi T, Shouno Y, Uchida M, Nagano Y, Kou T, et al. [A case of polyarteritis nodosa who developed rapidly progressive glomerulonephritis and presented with a tumor-like shadow on head CT in the remission stage]. Nihon Jinzo Gakkai shi. 1994;36(9):1052-6. |
| 826 | Ifteni P, Correll CU, Burtea V, Kane JM, Manu P. Sudden unexpected death in schizophrenia: autopsy findings in psychiatric inpatients. Schizophrenia research. 2014;155(1-3):72-6. Epub 2014/04/08. doi: 10.1016/j.schres.2014.03.011. PubMed PMID: 24704220. |
| 827 | Ideta MML, De Oliveira Paschoalino MC, Dos Santos AL, De Farias SR, Nascimento TL, Dos Santos Soares ML, et al. Intracranial extra-axial ependymoma: A case report. Brazilian Neurosurgery. 2018;37. doi: 10.1055/s-0038-1672890. |
| 828 | Ida S. [Evaluation and treatment of gastroesophageal reflux in infants and children]. Nihon rinsho Japanese journal of clinical medicine. 2004;62(8):1553-8. Epub 2004/09/04. PubMed PMID: 15344550. |
| 829 | Huth H, Merchant T. INDEPENDENCE AND SOCIAL ATTAINMENT IN IRRADIATED LONG-TERM SURVIVORS OF CHILDHOOD CRANIOPHARYNGIOMA. Pediatric Blood and Cancer. 2022;69(SUPPL 2):S102-S3. doi: 10.1002/pbc.29735. |
| 830 | Huq S, Khalafallah AM, Ruiz-Cardozo MA, Botros D, Oliveira LAP, Dux H, et al. A novel radiographic marker of sarcopenia with prognostic value in glioblastoma. Clinical neurology and neurosurgery. 2021;207:106782. Epub 2021/06/30. doi: 10.1016/j.clineuro.2021.106782. PubMed PMID: 34186275. |
| 831 | Huang YJ. Intracranial Immature teratoma diagnosed in a fetus- case report. Ultrasound in Medicine and Biology. 2019;45:S95. doi: 10.1016/j.ultrasmedbio.2019.07.317. |
| 832 | Huang HW, Yan LM, Yang YL, He X, Sun XM, Wang YM, et al. Bi-frontal pneumocephalus is an independent risk factor for early postoperative agitation in adult patients admitted to intensive care unit after elective craniotomy for brain tumor: A prospective cohort study. PloS one. 2018;13(7):e0201064. |
| 833 | Huang CB, Dikas E, Vedala K. Acute hemorrhagic stroke in a patient with metastatic carcinoma after anticoagulation use. Annals of Neurology. 2019;86:S191-S2. |
| 834 | Hua C, Wu S, Chemaitilly W, Lukose RC, Merchant TE. Predicting the probability of abnormal stimulated growth hormone response in children after radiotherapy for brain tumors. International journal of radiation oncology, biology, physics. 2012;84(4):990-5. |
| 835 | Hu W, Ye J, Wang J, Ma X, Zhang Z. Use of kilovoltage X-ray volume imaging in patient dose calculation for head-and-neck and partial brain radiation therapy. Radiation oncology (London, England). 2010;5:29. |
| 836 | Hu Q, Zhao J, Xu J, Zhou X, Shui Y, Shen L, et al. Long-Term Relief of Cerebral Radiation Necrosis Treated with Low-Dose Bevacizumab - a Report of 2 Cases. Oncology research and treatment. 2017;40(3):133-7. Epub 2017/02/24. doi: 10.1159/000455868. PubMed PMID: 28231593. |
| 837 | Hu MX, Liu J, Chen XB, Xu AQ, Shu SR, Wang CH, et al. Primary culture of human malignant meningioma cells and its intracranial orthotopic transplantation in nude mice. Nan fang yi ke da xue xue bao = Journal of Southern Medical University. 2018;38(3):340-5. |
| 838 | Hu J, Ljubimova JY, Inoue S, Konda B, Patil R, Ding H, et al. Phosphodiesterase type 5 inhibitors increase Herceptin transport and treatment efficacy in mouse metastatic brain tumor models. PloS one. 2010;5(4):e10108. |
| 839 | Hu J, Ljubimova JY, Inoue S, Konda B, Patil R, Ding H, et al. PDE5 inhibitors increase trastuzumab transport for treatment of HER2-positive metastatic brain tumors. Cancer research. 2010;70(8). doi: 10.1158/1538-7445.AM10-1447. |
| 840 | Howell JC, Rose SR. Pituitary disease in pediatric brain tumor survivors. Expert review of endocrinology & metabolism. 2019;14(4):283-91. Epub 2019/05/28. doi: 10.1080/17446651.2019.1620599. PubMed PMID: 31131647. |
| 841 | Hovsepyan G, Lehman A, Thomson CA, Iyengar NM, Beebe-Dimmer JL, Barac A, et al. Lipid levels and survival after obesity-related cancer in the Women's Health Initiative CVD biomarker cohort. Journal of Clinical Oncology. 2020;38(15). doi: 10.1200/JCO.2020.38.15_suppl.e13618. |
| 842 | Hossain JA, Ystaas LR, Mrdalj J, Välk K, Riecken K, Fehse B, et al. Lentiviral HSV-Tk.007-mediated suicide gene therapy is not toxic for normal brain cells. The journal of gene medicine. 2016;18(9):234-43. Epub 2016/08/05. doi: 10.1002/jgm.2895. PubMed PMID: 27490042. |
| 843 | Hornikx M, Uyttebroeck A, Vanrusselt D, Sleurs C, Verschueren S, Gewillig M. Exercise tolerance and physical activity short after intensive treatment in patients with childhood cancer. Cardiology in the Young. 2022;32:S101. doi: 10.1017/S1047951122001950. |
| 844 | Hornikx M, Uyttebroeck A, Vanrusselt D, Sleurs C, Gewillig M, Verschueren S. EXERCISE TOLERANCE AND PHYSICAL ACTIVITY SHORT AFTER INTENSIVE TREATMENT IN PATIENTS WITH CHILDHOOD CANCER. Pediatric Blood and Cancer. 2022;69:S503. doi: 10.1002/pbc.29952. |
| 845 | Horne VE, Bielamowicz K, Nguyen J, Hilsenbeck S, Lindsay H, Sonabend R, et al. Methylphenidate improves weight control in childhood brain tumor survivors with hypothalamic obesity. Pediatric blood & cancer. 2020;67(7):e28379. Epub 2020/05/10. doi: 10.1002/pbc.28379. PubMed PMID: 32383818. |
| 846 | Horne V, Bingham N, Inge T, Black J, Jenkins T, R Rose S. Hypothalamic obesity: 4 years of the international registry of hypothalamic obesity disorders. Hormone research in paediatrics. 2017;88:327-8. doi: 10.1159/000481424. |
| 847 | Hongell K, Kurki S, Sumelahti ML, Soilu-Hänninen M. Risk of cancer among Finnish multiple sclerosis patients. Multiple sclerosis and related disorders. 2019;35:221-7. Epub 2019/08/14. doi: 10.1016/j.msard.2019.08.005. PubMed PMID: 31404761. |
| 848 | Hong C, Manes RP, Vining EM, Omay SB. Utility of Intraoperative MRI Assessing Nasoseptal Flap Vascularity in Predicting Postoperative CSF Leak After Endoscopic Endonasal Approaches to Anterior Skull Base Lesions. Journal of Neurological Surgery, Part B Skull Base. 2022;83(SUPPL 1). doi: 10.1055/s-0042-1743720. |
| 849 | Holmes GE, Holmes FF, Baker AB, Hassanein RS. Childhood cancer survivors: attained adult heights. Compared with sibling controls. Clinical pediatrics. 1990;29(5):268-72. Epub 1990/05/01. doi: 10.1177/000992289002900503. PubMed PMID: 2340689. |
| 850 | Hoffman LM, Levy JM, Kilburn L, Billups C, Stokes V, McCourt E, et al. PEDIATRIC BRAIN TUMOR CONSORTIUM (PBTC)-055: A PHASE I STUDY OF TRAMETINIB AND HYDROXYCHLOROQUINE (HCQ) FOR BRAF-FUSION OR NEUROFIBROMATOSIS TYPE-1 (NF1)-ASSOCIATED PEDIATRIC GLIOMAS. Neuro-oncology. 2022;24:i35. doi: 10.1093/neuonc/noac079.129. |
| 851 | Hoefnagel D, Kwee LE, van Putten EH, Kros JM, Dirven CM, Dammers R. The incidence of postoperative thromboembolic complications following surgical resection of intracranial meningioma. A retrospective study of a large single center patient cohort. Clinical neurology and neurosurgery. 2014;123:150-4. |
| 852 | Hochberg I, Hochberg Z. Hypothalamic obesity. 2010. p. 185-96. |
| 853 | Hitchcock MH, Hollinshead AC, Chretien P, Rizzoli HV. Soluble membrane antigens of brain tumors. I. Controlled testing for cell-mediated immune responses in a long surviving glioblastoma multiforme patient. Cancer. 1977;40(2):660-6. |
| 854 | Hisada Y, Geddings JE, Ay C, Mackman N. Venous thrombosis and cancer: from mouse models to clinical trials. Journal of thrombosis and haemostasis : JTH. 2015;13(8):1372-82. Epub 2015/05/20. doi: 10.1111/jth.13009. PubMed PMID: 25988873; PubMed Central PMCID: PMC4773200. |
| 855 | Hirao M, Oka N, Hirashima Y, Horie Y, Takaku A. [Deep sylvian meningioma: case report and review of the literature]. No shinkei geka Neurological surgery. 1986;14(12):1471-8. Epub 1986/11/01. PubMed PMID: 3808209. |
| 856 | Hippolyte H, De Carli E, Pellier I, Rialland X, Coutant R. Risk of Long-Term Endocrine Sequelae in Survivors of Progressing Childhood Optic Pathway Glioma Treated by Upfront Chemotherapy: Preliminary Analyses of 102 Subjects from the French Multicentric BB-SFOP Registry. Journal of the Endocrine Society. 2020;4:A297. |
| 857 | Heuch JM, Heuch I, Akslen LA, Kvåle G. Risk of primary childhood brain tumors related to birth characteristics: a Norwegian prospective study. International journal of cancer. 1998;77(4):498-503. |
| 858 | Hervey-Jumper SL, Li J, Lau D, Molinaro AM, Perry DW, Meng L, et al. Awake craniotomy to maximize glioma resection: methods and technical nuances over a 27-year period. Journal of neurosurgery. 2015;123(2):325-39. Epub 2015/04/25. doi: 10.3171/2014.10.Jns141520. PubMed PMID: 25909573. |
| 859 | Hervey-Jumper SL, Berger M, Lau D. Awake craniotomy to maximize glioma resection: Methods and technical nuances with 561 patients. Journal of neurosurgery. 2015;122(6):A1544. doi: 10.3171/2015.6.JNS.AANS2014abstracts. |
| 860 | Hernandez-Lazaro A, De Leon-Durango R, Rios-Gomez C, Santana-Ojeda B, Perez-Rivero JM, Fernandez-Trujillo-Comenge PM, et al. INCIDENCE OF CLINICAL HYPERTENSION IN YOUNG TRANSGENDER PEOPLE AFTER A FIVEYEAR FOLLOW-UP: ASSOCIATION WITH GENDERAFFIRMING HORMONAL THERAPY. Journal of hypertension. 2023;41:e322. |
| 861 | Heo J, Heft N, Patel V, Kantor D. Differentiating tumefactive multiple sclerosis (MS) from tumor in a patient with comorbid MS and ovarian cancer. Annals of Neurology. 2018;84:S125. |
| 862 | Henegar L, Chang H, Walker CJ, Kashyap T, Maloof M, Wang F, et al. Synergistic anti-cancer effects of selinexor and bevacizumab in mouse xenograft models of glioblastoma. Cancer research. 2022;82(12). doi: 10.1158/1538-7445.AM2022-6022. |
| 863 | Helligsoe ASL, Weile KS, Kenborg L, Henriksen LT, Lassen-Ramshad Y, Amidi A, et al. Systematic Review: Sleep Disorders Based on Objective Data in Children and Adolescents Treated for a Brain Tumor. Frontiers in Neuroscience. 2022;16. doi: 10.3389/fnins.2022.808398. |
| 864 | Held P, Fellner C, Fellner F, Seitz J, Graf S, Hilbert M, et al. MRI of inner ear and facial nerve pathology using 3D MP-RAGE and 3D CISS sequences. The British journal of radiology. 1997;70(834):558-66. Epub 1997/06/01. doi: 10.1259/bjr.70.834.9227246. PubMed PMID: 9227246. |
| 865 | Heikens J, Ubbink MC, Van Der Pal HPJ, Bakker PJM, Fliers E, Smilde TJ, et al. Long term survivors of childhood brain cancer have an increased risk for cardiovascular disease. Cancer. 2000;88(9):2116-21. doi: 10.1002/(SICI)1097-0142(20000501)88:9<2116::AID-CNCR18>3.0.CO;2-U. |
| 866 | Heckel D, Comtesse N, Brass N, Blin N, Zang KD, Meese E. Novel immunogenic antigen homologous to hyaluronidase in meningioma. Human molecular genetics. 1998;7(12):1859-72. Epub 1998/11/13. doi: 10.1093/hmg/7.12.1859. PubMed PMID: 9811929. |
| 867 | He J, Jia L, Zhang Y, Tian Y, Hao P, Li T, et al. Obesity Paradox for Postoperative Mortality in Young Chinese Patients Undergoing Craniotomy for Brain Tumor Resection. Journal of neurosurgical anesthesiology. 2023. doi: 10.1097/ANA.0000000000000932. |
| 868 | Hazany S, Hesselink JR, Healy JF, Imbesi SG. Utilization of glutamate/creatine ratios for proton spectroscopic diagnosis of meningiomas. Neuroradiology. 2007;49(2):121-7. Epub 2006/11/07. doi: 10.1007/s00234-006-0167-z. PubMed PMID: 17086406. |
| 869 | Hayashi M, Kobayashi H, Handa Y, Kawano H, Kabuto M, Ishii H, et al. [Note on the mechanism of producing the plateau waves]. No to shinkei = Brain and nerve. 1984;36(11):1127-33. Epub 1984/11/01. PubMed PMID: 6335401. |
| 870 | Hayakawa M, Higuma Y, Hirata A, Ogawa M, Ikemura R, Yamashiro S, et al. A Surgical Case of Lipomatous Hypertrophy of the Atrial Septum. Heart Surgery Forum. 2024;27(7):E711-E7. doi: 10.59958/hsf.7055. |
| 871 | Hawighorst H, Debus J, Schreiber W, Knopp MV, Engenhart-Cabillic R, Essig M, et al. Contrast-enhanced magnetization transfer imaging: improvement of brain tumor conspicuity and delineation for radiosurgical target volume definition. Radiotherapy and oncology : journal of the European Society for Therapeutic Radiology and Oncology. 1997;43(3):261-7. |
| 872 | Hawasli AH, Rubin JB, Tran DD, Adkins DR, Waheed S, Hullar TE, et al. Anti-angiogenic agents for non-malignant brain tumors. Journal of Neurological Surgery, Part B: Skull Base. 2013;74. doi: 10.1055/s-0033-1336173. |
| 873 | Hatzoglou V, Ulaner GA, Zhang Z, Beal K, Holodny AI, Young RJ. Comparison of the effectiveness of MRI perfusion and fluorine-18 FDG PET-CT for differentiating radiation injury from viable brain tumor: a preliminary retrospective analysis with pathologic correlation in all patients. Clinical imaging. 2013;37(3):451-7. |
| 874 | Hatlen RR, Rajagopalan P. Investigating Trans-differentiation of Glioblastoma Cells in an In Vitro 3D Model of the Perivascular Niche. ACS biomaterials science & engineering. 2023;9(6):3445-61. Epub 2023/05/02. doi: 10.1021/acsbiomaterials.2c01310. PubMed PMID: 37129167. |
| 875 | Hartnett KP, Ward KC, Kramer MR, Lash TL, Mertens AC, Spencer JB, et al. The risk of preterm birth and growth restriction in pregnancy after cancer. International journal of cancer. 2017;141(11):2187-96. |
| 876 | Hartmann A, Saindane A, Bruce B, Newman N, Biousse V. Papilledema from Compression of Intracranial Venous Sinuses by Dural Meningiomas. Neurology. 2017;88(16). |
| 877 | Harland TA, Wang M, Gunaydin D, Fringuello A, Freeman J, Hosokawa PW, et al. Frailty as a Predictor of Neurosurgical Outcomes in Brain Tumor Patients. World neurosurgery. 2020;133:e813-e8. Epub 2019/10/13. doi: 10.1016/j.wneu.2019.10.010. PubMed PMID: 31605842. |
| 878 | Hardiansyah D, Riana A, Beer A, Glatting G. Model selection in single-time-point dosimetry using non-linear mixed-effects modeling. Journal of Nuclear Medicine. 2023;64. |
| 879 | Hardesty DA, Chen T, Brigeman ST, Lasker GF, Porter RW, Spetzler RF. Bleeding risk versus thromboembolic risk in patients undergoing posterior fossa skull base surgery in the modern ERA: Results of an aggressive chemical VTE prophylaxis paradigm. Journal of Neurological Surgery, Part B: Skull Base. 2016;77. |
| 880 | Harder T, Plagemann A, Harder A. Birth weight and subsequent risk of childhood primary brain tumors: a meta-analysis. American journal of epidemiology. 2008;168(4):366-73. Epub 2008/06/27. doi: 10.1093/aje/kwn144. PubMed PMID: 18579539. |
| 881 | Hansen JA, Stancel HH, Klesges LM, Tyc VL, Hinds PS, Wu S, et al. Eating behavior and BMI in adolescent survivors of brain tumor and acute lymphoblastic leukemia. Journal of pediatric oncology nursing : official journal of the Association of Pediatric Oncology Nurses. 2014;31(1):41-50. |
| 882 | Handisurya A, Gessl A, Marculecsu R, Dieckmann K, Hainfellner J, Woehrer A, et al. IGF-1 is not elevated in patients with HGG treated with radiochemotherapy. Neuro-oncology. 2015;17:v174. doi: 10.1093/neuonc/nov226.9. |
| 883 | Han MJ, Kim SC, Joo CU, Kim SJ. Cerebral salt-wasting syndrome in a child with Wernicke encephalopathy treated with fludrocortisone therapy: A case report. Medicine. 2016;95(36):e4393. |
| 884 | Han K, Peyret T, Marchand M, Quartino A, Gosselin NH, Girish S, et al. Population pharmacokinetics of bevacizumab in cancer patients with external validation. Cancer chemotherapy and pharmacology. 2016;78(2):341-51. |
| 885 | Hamilton MG, Hull RD, Pineo GF. Venous thromboembolism in neurosurgery and neurology patients: a review. Neurosurgery. 1994;34(2):280-96; discussion 96. Epub 1994/02/01. doi: 10.1227/00006123-199402000-00012. PubMed PMID: 8177390. |
| 886 | Hamilton MG, Hull RD, Pineo GF. Prophylaxis of venous thromboembolism in brain tumor patients. Journal of neuro-oncology. 1994;22(2):111-26. Epub 1994/01/01. doi: 10.1007/bf01052887. PubMed PMID: 7745464. |
| 887 | Hajnžić TF, Kaštelan M, Lukač J, Hajnžić T. Immunocompetent cells and lymphocyte reactivity to mitogens in levamisole-treated brain tumor children. Pediatric hematology and oncology. 1999;16(4):335-40. doi: 10.1080/088800199277164. |
| 888 | Hajnžić TF. Brain tumors in childhood (growth and development of treated patients). Paediatria Croatica, Supplement. 2006;50(1):209-13. |
| 889 | Haj-Hosseini N, Richter JCO, Hallbeck M, Wårdell K. Low dose 5-aminolevulinic acid: Implications in spectroscopic measurements during brain tumor surgery. Photodiagnosis and photodynamic therapy. 2015;12(2):209-14. doi: 10.1016/j.pdpdt.2015.03.004. PubMed Central PMCID: Medac(Germany). |
| 890 | Hahn BM, Schrell UM, Sauer R, Fahlbusch R, Ganslandt O, Grabenbauer GG. Prolonged oral hydroxyurea and concurrent 3d-conformal radiation in patients with progressive or recurrent meningioma: results of a pilot study. Journal of neuro-oncology. 2005;74(2):157-65. |
| 891 | Gurney JG, Ness KK, Stovall M, Wolden S, Punyko JA, Neglia JP, et al. Final height and body mass index among adult survivors of childhood brain cancer: childhood cancer survivor study. The Journal of clinical endocrinology and metabolism. 2003;88(10):4731-9. |
| 892 | Guo P, Ma J, Li S, Guo Z, Adams AL, Gallo JM. Targeted delivery of a peripheral benzodiazepine receptor ligand-gemcitabine conjugate to brain tumors in a xenograft model. Cancer chemotherapy and pharmacology. 2001;48(2):169-76. |
| 893 | Guo A, Chen X, Guo F, Shashikiran T, Song L. Clinical characteristic of 12 misdiagnosed cases with rare intracranial infection. Neurosurgery Quarterly. 2016;26(2):129-35. doi: 10.1097/WNQ.0000000000000150. |
| 894 | Guntner AS, Peyrl A, Mayr L, Englinger B, Berger W, Slavc I, et al. Cerebrospinal fluid penetration of targeted therapeutics in pediatric brain tumor patients. Acta neuropathologica communications. 2020;8(1):78. |
| 895 | Gump W. Use of Tranexamic Acid in Brain Tumor Surgery. Journal of Neurosurgery Pediatrics. 2023;31(3):67. doi: 10.3171/2023.3.PEDS.ASPN2023Abstracts. |
| 896 | Gulati S, Chakrabarty B. Dietary therapy in childhood epilepsy, an overview. International Journal of Epilepsy. 2014;1(1):27-35. doi: 10.1016/j.ijep.2014.03.002. |
| 897 | Guemes M, Fuente L, Caballero FJ, Martos-Moreno G, Munoz-Calvo MT, Argente J. Endocrinological effects in survivors of central nervous system tumours (CNST) after a 5 year follow-up. Hormone research in paediatrics. 2012;78:224. doi: 10.1159/000343183. |
| 898 | Gubernatorova E, Pavlova MG, Kaznacheeva T, Tselovalnikova T. Aseptic necrosis of femur in combination with hypocorticism and hypogonadism in childhood medulloblastoma survivor. Osteoporosis International. 2016;27(SUPPL 1):S408. doi: 10.1007/s00198-016-3530-x. |
| 899 | Gu J, Zhang Y, Shi M, Zhang M, Cheng L, Sun H, et al. Regional and systemic distribution of temozolomide from a polymer implant in rat brain. Drug Metabolism Reviews. 2014;45:94. doi: 10.3109/03602532.2013.868114. |
| 900 | Grzywotz A, Li Y, Unger N, Kiewert C, Chmielewski WX, Sure U, et al. Pituitary enlargement in patients with cerebrospinal fluid drainage due to ventricular shunt insertion: know the condition and do not mistake for adenoma. Pituitary. 2023;26(1):164-70. |
| 901 | Grzmil M, Hemmings BA. Deregulated signalling networks in human brain tumours. Biochimica et biophysica acta. 2010;1804(3):476-83. Epub 2009/11/03. doi: 10.1016/j.bbapap.2009.10.018. PubMed PMID: 19879382. |
| 902 | Grunberg SM, Weiss MH. Lack of efficacy of megestrol acetate in the treatment of unresectable meningioma. Journal of neuro-oncology. 1990;8(1):61-5. Epub 1990/02/01. doi: 10.1007/bf00182088. PubMed PMID: 2319292. |
| 903 | Groszberg D, Blumenthal IJ. Subcortical fibroblastoma of the brain. American Journal of Pathology. 1947;23(5):741-54. |
| 904 | Grosse H. [Coincidence of liver cirrhosis, diabetes mellitus, hypertension, obesity, cholecystopathy and meningioma in elderly women (author's transl)]. Zentralblatt fur allgemeine Pathologie u pathologische Anatomie. 1981;125(5):410-3. Epub 1981/01/01. PubMed PMID: 7331562. |
| 905 | Grossbach AJ, Mahaney KB, Menezes AH. Pediatric meningiomas: 65-year experience at a single institution. Journal of neurosurgery Pediatrics. 2017;20(1):42-50. Epub 2017/05/06. doi: 10.3171/2017.2.Peds16497. PubMed PMID: 28474981. |
| 906 | Groothuis DR, Lippitz BE, Fekete I, Schlageter KE, Molnar P, Colvin OM, et al. The effect of an amino acid-lowering diet on the rate of melphalan entry into brain and xenotransplanted glioma. Cancer research. 1992;52(20):5590-6. Epub 1992/10/15. PubMed PMID: 1394182. |
| 907 | Gribsholt SB, Fenton DC, Veres K, Farkas DK, Ording AG, Richelsen B, et al. The impact of hospital-diagnosed overweight or obesity on cancer risk: A nationwide danish cohort study. Pharmacoepidemiology and Drug Safety. 2017;26:224. doi: 10.1002/pds.4275. |
| 908 | Green JB, Oldewurtel HA, Forster FM. Glutamic oxalacetic transaminase (GOT) and lactic dehydrogenase (LDH) activities. Study in the cerebrospinal fluid of brain tumor patients, in normal human brain, and in brain tumor homogenates. Neurology. 1959;9(8):540-4. |
| 909 | Gravina GL, Mancini A, Colapietro A, Vitale F, Vetuschi A, Pompili S, et al. The novel CXCR4 antagonist, PRX177561, reduces tumor cell proliferation and accelerates cancer stem cell differentiation in glioblastoma preclinical models. Tumour biology : the journal of the International Society for Oncodevelopmental Biology and Medicine. 2017;39(6):1010428317695528. |
| 910 | Granbichler CA, Oberaigner W, Kuchukhidze G, Bauer G, Ndayisaba JP, Seppi K, et al. Cause-specific mortality in adult epilepsy patients from Tyrol, Austria: hospital-based study. Journal of neurology. 2015;262(1):126-33. |
| 911 | Gramanzini M, Gargiulo S, Greco A, Feliciello A, Lignitto L, Brunetti A, et al. 18F[FLT]PET and imaging post-processing for non -invasive assessment of siRNA treatment efficacy in experimental glioblastom. European Journal of Nuclear Medicine and Molecular Imaging. 2013;40:S283. doi: 10.1007/s00259-013-2535-3. |
| 912 | Graham R, Coven S, Stanek J, Folta A, Hollingsworth E, Kumar R. Risk of venous thromboembolism (VTE) in children with central nervous system (CNS) tumors-findings from a multi-center cohort study using the pediatric health information systems (PHIS) database. Haemophilia. 2019;25:30. doi: 10.1111/hae.13786. |
| 913 | Grafals-Ruiz N, Sánchez-Álvarez AO, Santana-Rivera Y, Lozada-Delgado EL, Rabelo-Fernandez RJ, Rios-Vicil CI, et al. MicroRNA-92b targets tumor suppressor gene FBXW7 in glioblastoma. Frontiers in Oncology. 2023;13. doi: 10.3389/fonc.2023.1249649. |
| 914 | Grafals-Ruiz N, Lozada-Delgado E, Santana-Rivera Y, Rivera-Diaz M, Valiyeva F, Vivas-Mejía P. MicroRNA-92b is a potential therapeutic target for Glioblastoma treatment. FASEB Journal. 2019;33(SUPPL 1):778.3. doi: 10.1096/fasebj.2019.33.1_supplement.778.3. |
| 915 | Goyal A, Kumari A, Solanki K, Verma A, Agrawal N. Exploring the healing powers of Saikosaponin A: A review of current perspectives. Pharmacological Research - Modern Chinese Medicine. 2024;12. doi: 10.1016/j.prmcm.2024.100500. |
| 916 | Gottmann L, Berchtold L, Mair M, Starzer AM, Fürst JH, Berger JM, et al. 2115P Pre-cachexia incidence in patients with solid cancer: A cross-sectional study. Annals of Oncology. 2023;34:S1107. doi: 10.1016/j.annonc.2023.09.897. |
| 917 | Goto T, Tanioka Y, Sakai T, Matsumoto I, Kakinoki K, Tanaka T, et al. Successful islet transplantation from a single pancreas harvested from a young, low-BMI, non-heart-beating cadaver. Transplantation proceedings. 2005;37(8):3430-2. |
| 918 | Gorenstein L, Shrot S, Ben-Ami M, Stern E, Yalon M, Hoffmann C, et al. Predictive factors for radiation-induced pituitary damage in pediatric patients with brain tumors. Radiotherapy and oncology : journal of the European Society for Therapeutic Radiology and Oncology. 2024;196:110268. |
| 919 | González-Briceño LG, Samara-Boustani D, Beltrand J, Grill J, Puget S, Dufour C, et al. Endocrine transition of care from pediatric to adult medicine in adolescents and young adult survivors of childhood brain tumour. experience at hopital universitaire necker-enfants malades and hopital universitaire la pitie-salpetriere-a follow-up study of the 2010-2015 cohort. Hormone research in paediatrics. 2019;91:466. doi: 10.1159/000501868. |
| 920 | Gonzalez-Aponte MF, Damato AR, Trebucq LL, Simon T, Cárdenas-García SP, Cho K, et al. Circadian regulation of MGMT expression and promoter methylation underlies daily rhythms in TMZ sensitivity in glioblastoma. Journal of neuro-oncology. 2024;166(3):419-30. |
| 921 | González Briceño LG, Kariyawasam D, Samara-Boustani D, Giani E, Beltrand J, Bolle S, et al. High Prevalence of Early Endocrine Disorders After Childhood Brain Tumors in a Large Cohort. The Journal of clinical endocrinology and metabolism. 2022;107(5):e2156-e66. |
| 922 | Goldman S, Pollack I, Kun L, Billups C, Broniscer A, Robinson G, et al. Phase II study of peginterferon alfa-2b (pegintron)/sylatron for pediatric patients with progressive or recurrent craniopharyngioma following radiotherapy: A pediatric brain tumor consortium study (PBTC-039). Neuro-oncology. 2018;20:i40-i1. |
| 923 | Góis CMS, de Araújo MCNV, de Silva KC, Araújo ATC. Evaluation of the knowledge about pusher syndrome in neurofunctional physiotherapists. Revista Neurociencias. 2011;19(4):595-601. doi: 10.34024/rnc.2011.v19.8325. |
| 924 | Gohar Y, Beshay U, Daba A, Hafez E. Bioactive compounds from Streptomyces nasri and its mutants with special reference to proteopolysaccharides. Polish journal of microbiology. 2006;55(3):179-87. Epub 2007/03/07. PubMed PMID: 17338270. |
| 925 | Goh KYC, Poon WS, Chan DTM, Chui PI. Tissue plasminogen activator expression in meningiomas and glioblastomas. Clinical neurology and neurosurgery. 2005;107(4):296-300. doi: 10.1016/j.clineuro.2004.09.010. |
| 926 | Gleeson HK, Stoeter R, Ogilvy-Stuart AL, Gattamaneni HR, Brennan BM, Shalet SM. Improvements in final height over 25 years in growth hormone (GH)-deficient childhood survivors of brain tumors receiving GH replacement. The Journal of clinical endocrinology and metabolism. 2003;88(8):3682-9. |
| 927 | Giustozzi M, Proietti G, Becattini C, Roila F, Agnelli G, Mandalà M. ICH in primary or metastatic brain cancer patients with or without anticoagulant treatment: a systematic review and meta-analysis. Blood advances. 2022;6(16):4873-83. Epub 2022/07/01. doi: 10.1182/bloodadvances.2022008086. |
| 928 | Giménez Jiménez I, García Claudio N, Montero Sánchez FI, Pérez Caballero P, Loro Represa JM, Argente Navarro MP. Anesthetic management of awake craniotomy for brain tumors: Our hospitals' experience. Journal of neurosurgical anesthesiology. 2016;28(2):S15-S6. |
| 929 | Gillet B, Jeanpierre E, Mameri A, Lambert T, Lavenu-Bombled C, Maynadié H, et al. Diagnosis and management of unexpected coexistence of severe haemophilia A and acquired von Willebrand disease related to mitral regurgitation. Haemophilia. 2020;26:59. doi: 10.1111/hae.13911. |
| 930 | Giljevic JS, Lenicek- Krleza J, Jakovljevic G, Bonevski A, Cvrlje FJ. Nutritional status in children with high-risk brain tumor-diagnostic and therapeutic possibilities. Pediatric Blood and Cancer. 2012;59(6):1086. doi: 10.1002/pbc.24295. |
| 931 | Gildea GC, Sandler CX, Eliadis P, Walker D, Pickersgill D, Shevill M, et al. Evaluating exercise for patients with brain cancer: The BRACE study. Asia-Pacific Journal of Clinical Oncology. 2021;17(SUPPL 9):183. doi: 10.1111/ajco.13716. |
| 932 | Ghesquieres H, Ferlay C, Richioud B, Isnardi V, Nicolas-Virlizier E, Domnisoru II, et al. Usefulness of positron emission tomography using 18F-FDG performed at diagnosis and during initial chemotherapy for imaging primary central nervous system lymphoma: A 24 immunocompetent patient's study. Blood. 2011;118(21). |
| 933 | Ghassemi N, Shoeibi A, Rouhani M. Deep neural network with generative adversarial networks pre-training for brain tumor classification based on MR images. Biomedical Signal Processing and Control. 2020;57. doi: 10.1016/j.bspc.2019.101678. |
| 934 | Gezer E, Cantürk Z, Selek A, Çetinarslan B, Tarkun İ, Sözen M, et al. Cushing's disease due to a pituitary adenoma as a component of collision tumor: A case report and review of the literature. Journal of medical case reports. 2020;14(1):59. |
| 935 | Georgakopoulou VE, Mathioudakis N, Papalexis P, Aravantinou-Fatorou A, Tarantinos K, Sklapani P, et al. Factors related to morbidity and mortality of meningiomas resection-associated venous thromboembolism (Review). Molecular and Clinical Oncology. 2023;19(3). doi: 10.3892/mco.2023.2666. |
| 936 | Georgakis MK, Dessypris N, Papadakis V, Tragiannidis A, Bouka E, Hatzipantelis E, et al. Perinatal and early life risk factors for childhood brain tumors: Is instrument-assisted delivery associated with higher risk? Cancer epidemiology. 2019;59:178-84. |
| 937 | Gelbart M, Nahum E, Gelbart M, Kaplan E, Kadmon G, Kershenovich A, et al. Hyperlactatemia in children following brain tumor resection: prevalence, associated factors, and clinical significance. Child's nervous system : ChNS : official journal of the International Society for Pediatric Neurosurgery. 2022;38(4):739-45. |
| 938 | Gaudino S, Martucci M, Botto A, Tartaglione T, Di Lella GM, Calandrelli R, et al. Dynamic susceptibility-weighted contrast-enhanced (DSC) perfusion MR imaging in pediatric neurooncology, first step: A clinical feasibility study. Neuroradiology. 2015;57(1):S124. doi: 10.1007/s00234-015-1557-x. |
| 939 | Gatson NT, Manikowski J, Bross S, Hinger B, Mauer T, Garcia T, et al. Prognostic features of malignant meningioma: Evaluation of aggressive meningiomas within a large rural hospital system database and first report of a metastatic chordoid meningioma to the lung. Neuro-oncology. 2019;21:vi142. |
| 940 | Garg A, Patel P, Lignelli A, Baron E, Kazim M. Coincidental Optic Nerve Meningioma and Thyroid Eye Disease. Ophthalmic plastic and reconstructive surgery. 2015;31(4):e94-5. Epub 2014/05/17. doi: 10.1097/iop.0000000000000113. PubMed PMID: 24833445. |
| 941 | García-Escobar I, Beato-Zambrano C, Muñoz Langa J, Brozos Vázquez E, Obispo Portero B, Gutiérrez-Abad D, et al. Pleiotropic effects of heparins: does anticoagulant treatment increase survival in cancer patients? Clinical & translational oncology : official publication of the Federation of Spanish Oncology Societies and of the National Cancer Institute of Mexico. 2018;20(9):1097-108. |
| 942 | Garavaglia MM, Mak TH, Cusimano MD, Rigamonti A, Crescini C, McCredy VA, et al. Body Mass Index as a risk factor for increased serum lactate during craniotomy. Minerva anestesiologica. 2013;79(10):1132-9. |
| 943 | Garavaglia M, Timothy MH, Cusimano M, Rigamonti A, Crescini C, McCredie V, et al. Hyperlactatemia during craniotomy: Evidence of muscle hypoperfusion? Canadian Journal of Anesthesia. 2013;60(1):S68. doi: 10.1007/s12630-013-0063-z. |
| 944 | Garavaglia M, Rigamonti A, Cusimano M, Crescini C, Mazer D, Hare G, et al. Dexmedetomidine for complex patients undergoing awake craniotomy. Anesthesia and analgesia. 2013;116. |
| 945 | Garavaglia M, Rigamonti A, Cusimano M, Crescini C, Mazer D, Hare G, et al. Dexmedetomidine for awake craniotomy in complex patients. Canadian Journal of Anesthesia. 2013;60(1):S67. doi: 10.1007/s12630-013-0063-z. |
| 946 | Garavaglia M, McCredie V, Mak T, Rigamonti A, Cusimano M, Baker A, et al. Serum lactate: A potential marker of tissue ischemia or metabolic dysfunction during craniotomy for brain tumor resection. Journal of neurosurgical anesthesiology. 2012;24(4):502-3. |
| 947 | Gans MS, Byrne SF, Glaser JS. Standardized A-scan echography in optic nerve disease. Archives of ophthalmology (Chicago, Ill : 1960). 1987;105(9):1232-6. Epub 1987/09/01. doi: 10.1001/archopht.1987.01060090090035. PubMed PMID: 3307721. |
| 948 | Gallus S, Foschi R, Talamini R, Altieri A, Negri E, Franceschi S, et al. Risk factors for prostate cancer in men aged less than 60 years: a case-control study from Italy. Urology. 2007;70(6):1121-6. Epub 2007/12/26. doi: 10.1016/j.urology.2007.07.020. PubMed PMID: 18158031. |
| 949 | Gallizia A, Mauro V, Crocco M, Allegri AEM, Napoli F, Garrè ML, et al. Bone mass and fracture prevalence in childhood brain cancer survivors 2, 5 or 7 years after off therapy. Hormone research in paediatrics. 2019;91:73. doi: 10.1159/000501868. |
| 950 | Gaillard PJ, Gladdines W, Appeldoorn CCM, Rip J, Boogerd WJ, Beijnen JH, et al. Development of glutathione pegylated liposomal doxorubicin (2B3-101) for the treatment of brain cancer. Cancer research. 2012;72(8). doi: 10.1158/1538-7445.AM2012-5687. |
| 951 | Gadoue SM, Toomeh D, Schultze BE, Schulte RW. A dose–volume constraint (DVC) projection-based algorithm for IMPT inverse planning optimization. Medical physics. 2022;49(4):2699-708. doi: 10.1002/mp.15504. |
| 952 | Furuhata H, Araki K, Ogawa T. Financial Estimation of the Uncertainty in Medicine Using Present Value of Medical Fees and a Mortality Risk Prediction Model: a Retrospective Cohort Study. Journal of medical systems. 2021;45(11):98. |
| 953 | Fukui T, Suehiro S, Shibata T, Hirai H, Aoyama T. Concomitant pulmonary embolectomy and excision of a left atrial myxoma. Journal of cardiac surgery. 2001;17(5):408-9. doi: 10.1111/j.1540-8191.2001.tb01169.x. |
| 954 | Fu W, Li Y, Liu R, Li J. Incidence and Risk Factors of Delirium Following Brain Tumor Resection: A Retrospective National Inpatient Sample Database Study. World neurosurgery. 2024. doi: 10.1016/j.wneu.2024.06.108. |
| 955 | Fròsina G, Profumo A, Marubbi D, Marcello D, Ravetti JL, Daga A. ATR kinase inhibitors NVP-BEZ235 and AZD6738 effectively penetrate the brain after systemic administration. Radiation oncology (London, England). 2018;13(1):76. |
| 956 | Frere C, Pernod G, Idbaih A, Mahe I. Direct Oral Anticoagulants versus Low Molecular Weight Heparins in Patients with Brain Tumor Receiving Therapeutic Anticoagulation: A Systematic Review and Meta-Analysis. Research and Practice in Thrombosis and Haemostasis. 2023;7. |
| 957 | Frati A, Pesce A, Toccaceli G, Fraschetti F, Caruso R, Raco A. Spinal Meningiomas Prognostic Evaluation Score (SPES): predicting the neurological outcomes in spinal meningioma surgery. Neurosurgical review. 2019;42(1):115-25. |
| 958 | Fouse SD, Steino A, Butowski N, Bacha JA, Kanekal S, Santos ND, et al. Dianhydrogalactitol inhibits the growth of glioma stem and non-stem cultures, including temozolomide-resistant cell lines, in vitro and in vivo. Cancer research. 2015;75(15). doi: 10.1158/1538-7445.AM2015-2562. |
| 959 | Fouse SD, Steino A, Butowski N, Bacha JA, Kanekal S, Dos Santos N, et al. Dianhydrogalactitol inhibits the growth of glioblastoma multiforme stem and non-stem cultures, in vitro and in vivo. Cancer research. 2015;75(23). doi: 10.1158/1538-7445.BRAIN15-B28. |
| 960 | Fourniols T, Randolph LD, Staub A, Vanvarenberg K, Leprince JG, Préat V, et al. Temozolomide-loaded photopolymerizable PEG-DMA-based hydrogel for the treatment of glioblastoma. Journal of controlled release : official journal of the Controlled Release Society. 2015;210:95-104. |
| 961 | Fotovati A, Abu-Ali S, Wang PS, Deleyrolle L, Lee C, Triscott J, et al. YB-1 bridges neural stem cells and brain tumor-initiating cells via its roles in differentiation and cell growth. Neuro-oncology. 2012;14:i14-i5. doi: 10.1093/neuonc/nos095. |
| 962 | Forsgren L, Nyström L. An incident case-referent study of epileptic seizures in adults. Epilepsy research. 1990;6(1):66-81. Epub 1990/05/01. doi: 10.1016/0920-1211(90)90010-s. PubMed PMID: 2357957. |
| 963 | Forsell P, Hellers G. The Swedish Adjustable Gastric Banding (SAGB) for morbid obesity: 9 year experience and a 4-year follow-up of patients operated with a new adjustable band. Obesity surgery. 1997;7(4):345-51. Epub 1997/08/01. doi: 10.1381/096089297765555601. PubMed PMID: 9730522. |
| 964 | Fonseca BGA, Segredo MP, Novak G, Molinari B, Sakamoto AP, Ferriani VPL, et al. Malignancy in childhood onset-systemic lupus erythematosus: Real-life data from a nationwide series. Advances in Rheumatology. 2018;58. doi: 10.1186/s42358-018-0019-7. |
| 965 | Fluss R, Kobets AJ, Inocencio JF, Hamad M, Feigen C, Altschul DJ, et al. The incidence of venous thromboembolism following surgical resection of intracranial and intraspinal meningioma. A systematic review and retrospective study. Clinical neurology and neurosurgery. 2021;201:106460. |
| 966 | Florenzi C, Rutili S, Fintoni T, Para O, Blasi E, Bacci F, et al. An unusual swelling on the scalp. Italian Journal of Medicine. 2018;12(2):58. doi: 10.4081/itjm.2018.s2. |
| 967 | Fleming CA, Viani K, Murphy AJ, Mosby TT, Arora B, Schoeman J, et al. The development, testing, and preliminary feasibility of an adaptable pediatric oncology nutrition algorithm for low-middle income countries. Indian journal of cancer. 2015;52(2):225-8. |
| 968 | Fleming A, Wang KW, Scheinemann K, Valencia M, Duckworth J, Banfield L, et al. The effectiveness of lifestyle intervention, pharmacotherapy or bariatric surgery on lowering BMI z-score in obese survivors of childhood brain tumors: A systematic review. Pediatric Blood and Cancer. 2015;62:S282. |
| 969 | Fisusi FA, Alzharani A, Summers I, Morger J, Okubanjo O, Wai Chooi K, et al. Lomustine nanoparticles are effective brain cancer treatments. Cancer research. 2013;73(8). doi: 10.1158/1538-7445.AM2013-4519. |
| 970 | Fisher RS, Rausch JR, Ferrante AC, Prussien KV, Olshefski RS, Vannatta KA, et al. Trajectories of health behaviors across early childhood cancer survivorship. Psycho-oncology. 2019;28(1):68-75. |
| 971 | Fisahn C, Cambier Z, Kobata K, Schmidt C, Yilmaz E, Lim P, et al. Functional rehabilitation using the hybrid assistive limb exoskeleton: A first experience in the united states. Global Spine Journal. 2018;8(1):145S-6S. doi: 10.1177/2192568218771030. |
| 972 | Firkins J, Ambady P, Neuwelt E. Comorbidities in survival of adults with a glioblastoma. Neuro-oncology. 2017;19:vi112. doi: 10.1093/neuonc/nox168. |
| 973 | Ferrulli A, Parrino C, Premoli C, Macrì C, Massarini S, Luzi L. Deep transcranial magnetic stimulation in patients with obesity: Italian safety data. Obesity facts. 2019;12:11. doi: 10.1159/000489691. |
| 974 | Ferronato MJ, Alonso EN, Salomón DG, Fermento ME, Gandini NA, Quevedo MA, et al. Antitumoral effects of the alkynylphosphonate analogue of calcitriol EM1 on glioblastoma multiforme cells. The Journal of steroid biochemistry and molecular biology. 2018;178:22-35. |
| 975 | Ferro JC, Teixeira JP, Próspero I, Barbosa D, Silva DG, Fontão De Castro S, et al. Not always a zebra - Clear Cell Renal Cell Carcinoma Metastasis detected in [68Ga]Ga-DOTA-NOC PET/CT. European Journal of Nuclear Medicine and Molecular Imaging. 2023;50:S251-S2. |
| 976 | Ferreira MJ, Castedo JL, Costa MM, Carvalho DM. Unilateral Adrenalectomy Can Induce Control in ARMC5 Mutations Patients. Journal of the Endocrine Society. 2020;4:A471-A2. doi: 10.1210/jendso/bvaa046.929. |
| 977 | Fernandes EV, Rodrigues F, Pardal C, Carvalho L, Jardim Pena D. Meningioma during pregnancy-how to prevent complications during labor-case report. Journal of Perinatal Medicine. 2013;41. doi: 10.1515/jpm-2013-2003. |
| 978 | Ferdinande K, Harlet L, Decaestecker J, De Wulf D. Unexpected outcome of a sigmoid lesion believed to be malignant. Acta gastro-enterologica Belgica. 2023;86(2):383-4. Epub 2023/07/10. doi: 10.51821/86.2.11927. PubMed PMID: 37428178. |
| 979 | Feng FY, Li Q, Wang ZJ. Effect of uroacitides in improving quality of life of advanced cancer patients. Chinese Journal of Clinical Rehabilitation. 2004;8(2):384-5. |
| 980 | Felicetti F, D'Ascenzo F, Brignardello E, Moretti C, Gaita F. Prevalence of cardiovascular risk factors in long-term survivors of childhood cancer. European Heart Journal. 2013;34:477. |
| 981 | Fantozzi P, Fisher MJ, Herrington JD, Li Y, McCormack SE, Parish-Morris J, et al. FATIGUE AS A RISK FACTOR FOR REDUCED SOCIAL CONNECTEDNESS IN PEDIATRIC BRAIN TUMOR SURVIVORS. Neuro-oncology. 2024;26:iv181. doi: 10.1093/neuonc/noae064.627. |
| 982 | Fangusaro J, Onar-Thomas A, Young Poussaint T, Lensing S, Wu S, Ligon AH, et al. SELUMETINIB IN PEDIATRIC PATIENTS WITH NON-NEUROFIBROMATOSIS TYPE 1-ASSOCIATED, NONOPTIC PATHWAY (OPG) AND NON-PILOCYTIC RECURRENT/ PROGRESSIVE LOW-GRADE GLIOMA HARBORING BRAFV600E MUTATION OR BRAF-KIAA1549 FUSION: A MULTICENTER PROSPECTIVE PEDIATRIC BRAIN TUMOR CONSORTIUM (PBTC) PHASE 2 TRIAL. Neuro-oncology. 2022;24:i88. doi: 10.1093/neuonc/noac079.322. |
| 983 | Fan Y, Zha R, Sano T, Zhao X, Liu S, Woollam MD, et al. Mechanical tibial loading remotely suppresses brain tumors by dopamine-mediated downregulation of CCN4. Bone Research. 2021;9(1). doi: 10.1038/s41413-021-00144-2. PubMed Central PMCID: Sigma(United States) |
| 984 | Fan Y, Sun Y, Chang W, Zhang X, Tang J, Zhang L, et al. Bioluminescence imaging and two-photon microscopy guided laser ablation of GBM decreases tumor burden. Theranostics. 2018;8(15):4072-85. doi: 10.7150/thno.25357. |
| 985 | Fan CH, Wang TW, Hsieh YK, Wang CF, Gao Z, Kim A, et al. Enhancing Boron Uptake in Brain Glioma by a Boron-Polymer/Microbubble Complex with Focused Ultrasound. ACS applied materials & interfaces. 2019;11(12):11144-56. |
| 986 | Falanga A, Russo L. Epidemiology, risk and outcomes of venous thromboembolism in cancer. Hamostaseologie. 2012;32(2):115-25. Epub 2011/10/06. doi: 10.5482/ha-1170. PubMed PMID: 21971578. |
| 987 | Faienza MF, Delvecchio M, Indrio F, Francavilla R, Acquafredda A, Cavallo L. Acute pancreatitis in a girl with panhypopituitarism due to craniopharyngioma on growth hormone treatment. A combination of risk factors. Hormone research. 2009;71(6):372-5. |
| 988 | Fahey FH, Kinahan PE, Doot RK, Kocak M, Thurston H, Poussaint TY. Variability in PET quantitation within a multicenter consortium. Medical physics. 2010;37(7):3660-6. Epub 2010/09/14. doi: 10.1118/1.3455705. PubMed PMID: 20831073; PubMed Central PMCID: PMC2905446. |
| 989 | Faccini R, Collamati F, Morganti S, Patera V, Russomando A, Sciubba A, et al. A novel radioguided surgery technique exploiting-b decays. Radiotherapy and Oncology. 2014;110:S33-S4. |
| 990 | Evernden B, Law V, Kenchappa R, Puskas J, Ryzhova E, Fedorenko I, et al. Detection, molecular profiling and culture of CSF-CTCS in leptomeningeal disease (LMDZ) in melanoma. Neuro-oncology. 2017;19:vi46. doi: 10.1093/neuonc/nox168. |
| 991 | Evans A, Nelson MB, Britt B, Margol A, Robison N, Dhall G, et al. Boswellia as an alternative to dexamethasone in children with brain tumors. Neuro-oncology. 2014;16:i115-i6. doi: 10.1093/neuonc/nou079. |
| 992 | Etame AB, Smith CA, Chan WC, Rutka JT. Design and potential application of PEGylated gold nanoparticles with size-dependent permeation through brain microvasculature. Nanomedicine : nanotechnology, biology, and medicine. 2011;7(6):992-1000. |
| 993 | Essock-Burns E, Li Y, Lupo J, Polley MY, Butowski N, Cha S, et al. Comparison of DSC-derived perfusion parameters in patients newly diagnosed with GBM treated with either RT + TMZ or RT + TMZ + anti-angiogenic therapy. Neuro-oncology. 2010;12:iv118. doi: 10.1093/neuonc/noq116. |
| 994 | Espinosa I, D'Angelo E, Corominas M, Gonzalez A, Prat J. Mixed endometrial carcinomas with a “low-grade serous”–like component: a clinicopathologic, immunohistochemical, and molecular genetic study. Human pathology. 2018;71:65-73. doi: 10.1016/j.humpath.2017.10.016. |
| 995 | Eslick GD, Seex K. A 49-year-old woman with a history of head trauma. Brain Pathology. 2008;18(1):98-9. doi: 10.1111/j.1750-3639.2007.00115_2.x. |
| 996 | Erol S, Ayöz S, Çiftçi F, Özçinar E, Kaya AG, Çiledaǧ A, et al. Does the presence of right ventricular hypo/akinesia on echocardiography in patients with submassive pulmonary embolism increase the risk of hemodynamic deterioration and mortality? Turkish Thoracic Journal. 2019;20:S59. |
| 997 | Erdreich-Epstein A, Robison N, Ren X, Zhou H, Ji L, Margo A, et al. PID1 (NYGGF4), a new tumor suppressor-like gene in pediatric and adult brain tumors. Neuro-oncology. 2013;15:i11. doi: 10.1093/neuonc/not047. |
| 998 | Erdlenbruch B, Jendrossek V, Marx M, Hunold A, Eibl H, Lakomek M. Antitumor effects of erucylphosphocholine on brain tumor cells in vitro and in vivo. Anticancer research. 1998;18(4a):2551-7. Epub 1998/08/15. PubMed PMID: 9703909. |
| 999 | Engler D, Shen Y, Gusella J, Betensky RA. Comparison of clinical subgroup aCGH profiles through pseudolikelihood ratio tests. Statistical Applications in Genetics and Molecular Biology. 2011;10(1). doi: 10.2202/1544-6115.1407. |
| 1000 | Emfietzoglou R, Spyrou N, Mantzoros CS, Dalamaga M. Could the endocrine disruptor bisphenol-A be implicated in the pathogenesis of oral and oropharyngeal cancer? Metabolic considerations and future directions. Metabolism: clinical and experimental. 2019;91:61-9. |
| 1001 | Elowe-Gruau E, Beltrand J, Brauner R, Pinto G, Samara-Boustani D, Thalassinos C, et al. Childhood craniopharyngioma: hypothalamus-sparing surgery decreases the risk of obesity. The Journal of clinical endocrinology and metabolism. 2013;98(6):2376-82. |
| 1002 | Elder JA. Survival and Cancer in Laboratory Mammals Exposed to Radiofrequency Energy. Bioelectromagnetics. 2003;24(SUPPL. 6):S101-S6. doi: 10.1002/bem.10175. |
[truncated: 83,014 more chars]
