# Supplementary material for: Association between anthropometric factors and meningioma risk: A systematic review and meta-analysis
Source: PLoS One. 2025 May 13;20(5):e0323461. doi: 10.1371/journal.pone.0323461 (PMC12074524; doi:10.1371/journal.pone.0323461)
Supplement: S3 Table — (DOCX) [file pone.0323461.s003.docx]

**S3 Table Additional basic characteristic of included studies.**

| Study | Exposure | Exposure level | Risk estimate and 95% CI | Matching or adjustment |
| --- | --- | --- | --- | --- |
| Jhawar et al. 2003 [5] | BMI (kg/m^2^) | <22 | 1.0 (referent) | Age , menopausal status, and PMH use |
|  |  | 22-24.9 | 1.10(0.61-1.97) |  |
|  |  | ≥25 | 1.61(0.96-2.70） |  |
| Benson et al. 2008 [6] | BMI (kg/m^2^) | <25 | 1.0 (referent) | Age, height, strenuous exercise, socioeconomic level, smoking, alcohol intake, parity, age at first birth, OC. |
|  |  | 25-29.9 | 1.01(0.79-1.29) |  |
|  |  | ≥30 | 1.40(1.08-1.87) |  |
|  | Height (cm) | <160 | 1.0(referent) | Age, strenuous exercise, socioeconomic level, smoking, alcohol intake, parity, age at first birth, OC, and BMI. |
|  |  | 160-164.9 | 1.05(0.81-1.37） |  |
|  |  | 165+ | 1.25(0.98-1.60) |  |
| Johnson et al. 2011 [7] | BMI (kg/m^2^) | 18.5-24.9 | 1.0 (referent) | Age |
|  |  | 25-29.9 | 0.92(0.59-1.44) |  |
|  |  | 30.0-34.9 | 2.14(1.36-3.36) |  |
|  |  | ≥35 | 1.99(1.06-3.71) |  |
|  | Height (inches) | ≤62 | 1.0 (referent) | Age |
|  |  | 63-64 | 1.21(0.74-1.97) |  |
|  |  | 65-66 | 1.24(0.74-2.07) |  |
|  |  | >66 | 1.65(0.97-2.82) |  |
|  | Waist circumference | ≤30.25 | 1.0 (referent) | Age |
|  |  | 30.26–33.50 | 0.92(0.50-1.69) |  |
|  |  | 33.51–37.75 | 1.56(0.92-2.67) |  |
|  |  | >37.75 | 2.13(1.28-3.56) |  |
|  | WHR | ≤0.77 | 1.0 (referent) | Age |
|  |  | 0.78–0.83 | 1.07 (0.63–1.81) |  |
|  |  | 0.84–0.89 | 1.04 (0.61–1.76) |  |
|  |  | >0.89 | 1.56 (0.96–2.54) |  |
| Michaud et al. 2011 [8] | BMI (kg/m^2^) | 20–24.9 | 1.0 (referent) | Age, country, sex, education, physical activity. |
|  |  | <20 | 1.00 (0.46–2.19) |  |
|  |  | 25-30 | 1.34 (0.97–1.86) |  |
|  |  | ≥30 | 1.48 (0.98–2.23) |  |
|  | Height (cm) | Q1 | 1.0 (referent) | Age, country, sex, education, weight, waist circumference, and WHR |
|  |  | Q2 | 1.17 (0.78-1.78) |  |
|  |  | Q3 | 0.88 (0.57-1.38) |  |
|  |  | Q4 | 0.88 (0.57-1.38) |  |
|  | Waist circumference | Q1 | 1.0 (referent) | Age, country, sex, education |
|  |  | Q2 | 1.18 (0.73–1.88) |  |
|  |  | Q3 | 1.06 (0.65–1.72) |  |
|  |  | Q4 | 1.71 (1.08–2.73) |  |
|  | WHR | Q1 | 1.0 (referent) | Age, country, sex, education |
|  |  | Q2 | 1.04 (0.66–1.65) |  |
|  |  | Q3 | 0.99 (0.62–1.56) |  |
|  |  | Q4 | 1.27 (0.81–1.99) |  |
| Edlinger et al. 2012 [9] | BMI (kg/m^2^) | 21 | 1.0 (referent) | Age, sex, birth year (in decades), and smoking status |
|  |  | 23 | 0.87(0.58-1.32) |  |
|  |  | 25 | 1.01(0.68-1.49) |  |
|  |  | 27 | 1.05(0.71-1.56) |  |
|  |  | 31 | 1.39(0.96-2.03) |  |
| Wiedmann et al. 2013 [10] | BMI (kg/m^2^) | 20-24.9 | 1.0 (referent) | Age, sex |
|  |  | <20 | 0.82 (0.35–1.92) |  |
|  |  | 25-29.9 | 1.22 (0.83–1.80) |  |
|  |  | ≥30 | 1.48 (0.89–2.45) |  |
|  | Height (cm) | Q1 | 1.0 (referent) | Age, sex |
|  |  | Q2 | 0.65 (0.43–0.99) |  |
|  |  | Q3 | 0.69 (0.29–1.22) |  |
|  |  | Q4 | 0.52 (0.22–1.20) |  |
| Wiedmann et al. 2017 [11] | BMI (kg/m^2^) | 20-24.9 | 1.0 (referent) | Age, sex, heigh, and birth year cohort |
|  |  | 25-29.9 | 1.08 (0.99–1.17) |  |
|  |  | ≥30 | 1.12 (0.98–1.27) |  |
|  | Height (cm) in men | Q1 | 1.0 (referent) | Age BM, and birth year cohort |
|  |  | Q2 | 1.11 (0.90–1.35) |  |
|  |  | Q3 | 1.23 (1.00–1.51) |  |
|  |  | Q4 | 1.32 (1.08–1.61) |  |
|  | Height (cm) in women | Q1 | 1.0 (referent) | Age BM, and birth year cohort |
|  |  | Q2 | 1.12 (0.99–1.27) |  |
|  |  | Q3 | 1.21 (1.08–1.37) |  |
|  |  | Q4 | 1.25 (1.11–1.41) |  |
| Muskens et al. 2019 [12] | BMI (kg/m^2^) | <25 | 1.0 (referent) | Exact birth year, ethnicity, study area, alcohol, smoking status, diabetes, hypertension, and education |
|  |  | 25-29.9 | 1.21 (1.02–1.44) |  |
|  |  | ≥30 | 1.22 (0.99–1.50) |  |
|  | Height (cm) | Q1 | 1.0 (referent) | Exact birth year, ethnicity, study area, alcohol, smoking status, diabetes, hypertension, and education |
|  |  | Q2 | 0.93 (0.73–1.18) |  |
|  |  | Q3 | 1.05 (0.83–1.33) |  |
|  |  | Q4 | 0.92 (0.70–1.22) |  |
| Ogawa et al. 2020 [13] | BMI (kg/m^2^) | ≥18.5, <23 | 1.0 (referent) | Age, sex, smoking, alcohol intake, coffee, green tea, past history of diabetes mellitus, and allergy. |
|  |  | ≥23, <25 | 0.98 (0.45-2.16) |  |
|  |  | ≥25, <27.5 | 1.88 (0.91-3.85) |  |
|  |  | 27.5-40 | 1.98 (0.84-4.67) |  |
|  | Height (cm) | <150 (women) or <162 (men) | 1.0 (referent) | Age, sex, smoking, alcohol intake, coffee, green tea, past history of diabetes mellitus, and allergy. |
|  |  | ≥150, <154 (women) or ≥162, <167 (men) | 0.74 (0.37-1.48) |  |
|  |  | >154 (women) or >167 (men) | 0.70 (0.34-1.44) |  |
