# Supplementary material for: Association between anthropometric factors and meningioma risk: A systematic review and meta-analysis
Source: PLoS One. 2025 May 13;20(5):e0323461. doi: 10.1371/journal.pone.0323461 (PMC12074524; doi:10.1371/journal.pone.0323461)
Supplement: S4 Table — (DOCX) [file pone.0323461.s004.docx]

S4 Table Risk bias of included studies based on the Newcastle–Ottawa Scale

| Reference | Selection | Comparability | Outcome | Total |
| --- | --- | --- | --- | --- |
| Jhawar et al. 2003 [5] | 2 | 1 | 2 | 5 |
| Benson et al. 2008 [6] | 3 | 1 | 3 | 7 |
| Johnson et al. 2011 [7] | 3 | 1 | 3 | 7 |
| Michaud et al. 2011 [8] | 4 | 1 | 3 | 8 |
| Edlinger et al. 2012 [9] | 4 | 1 | 2 | 7 |
| Wiedmann et al. 2013 [10] | 4 | 1 | 3 | 8 |
| Wiedmann et al. 2017 [11] | 4 | 1 | 2 | 7 |
| Muskens et al. 2019 [12] | 3 | 1 | 3 | 7 |
| Ogawa et al. 2020 [13] | 3 | 1 | 3 | 7 |
